# Development of Cathepsin B‑Activatable Cell-Penetrating Peptides for Tumor Targeting

**DOI:** 10.1021/acsptsci.5c00746

**Published:** 2026-02-18

**Authors:** Konstantin Kuhne, Lydia Strohbach, Christin Neuber, Robert Wodtke, Gloria Ruiz-Gómez, Birgit Belter, Florian Brandt, Lara Gluhacevic von Krüchten, Max Keller, M. Teresa Pisabarro, Klaus Kopka, Jens Pietzsch, Reik Löser

**Affiliations:** † Institute of Radiopharmaceutical Cancer Research, 39063 Helmholtz-Zentrum Dresden-Rossendorf, Bautzner Landstraße 400, Dresden 01328, Germany; § BIOTEC, 9169 Technische Universität Dresden, Tatzberg 47-49, Dresden 01307, Germany; ∥ Institute of Pharmacy, Faculty of Chemistry and Pharmacy, 9147 Universität Regensburg, Universitätsstraße 31, Regensburg 93053, Germany; ‡ Faculty of Chemistry and Food Chemistry, School of Science, Technische Universität Dresden, Dresden 01069, Germany

**Keywords:** kinetic hysteresis, PET imaging, pharmacokinetics, protease substrates, radiometal-based labeling, theranostics

## Abstract

Extracellular cathepsin B is a driver of tumor progression and metastasis, and its potential as a diagnostic and prognostic marker is increasingly recognized. To harness its activity for triggering the uptake of activatable cell-penetrating peptides (ACPPs) *in vivo*, kinetically suitable and stable endopeptidase substrates for this cysteine protease, which mainly acts as a carboxydipeptidase, are required. This challenge was tackled by C-terminal elongation of the previously identified GIVRAK sequence to octapeptides and systematic structural variation, which has revealed that the endopeptidase activity of cathepsin B is associated with kinetic hysteresis and the P4′ residue plays a key role in this regard, as further investigated by enzyme–substrate docking in silico. By replacing the N-terminal motif with GFLG and focused *N*-methylation of the backbone, the substrate serum half-life was extended from 3.7 min to 23.4 h. Integrating this sequence into the fluorophore-conjugated ACPP and fluorescence microscopy in U87MG cells confirmed cathepsin B-mediated uptake on the basis of selective inhibitors and control probes. PET imaging and biodistribution studies *ex vivo* with a NODAGA-conjugated ACPP analogue radiolabeled with copper-64 in a murine U87MG-derived xenograft model together with radiopharmacological investigations in normal Wistar rats demonstrated more favorable pharmacokinetics compared to the corresponding CPP. Although tumor-associated proteolytic activation *in vivo* is indicated, this does not contribute to tumor retention as judged from control experiments under pharmacological blockade of cathepsin B and with nonfunctional analogues. The obtained results are discussed in the context of previous data for radiolabeled ACPPs, and limitations for the general use of ACPPs for radiotheranostic approaches are highlighted.

## Graphical abstract

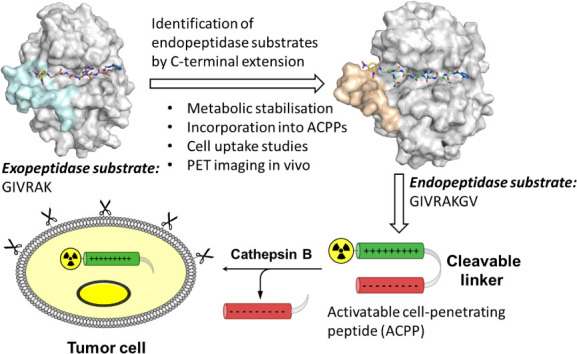

Initiation and progression of cancer strongly correlate with increased proteolytic activity. Hence, addressing tumor-associated proteases by various molecular vectors offers great potential toward therapy and diagnosis of neoplastic diseases.^1–5^ Besides inhibition of proteases as an option for therapeutic intervention, their enzymatic activity can be conversely exploited for tumor-directed delivery of therapeutic payloads such as cytostatic agents or reporter groups such as radionuclides and paramagnetic metal ions for nuclear and MR-based imaging, respectively.^6–14^ Apart from various protease-responsive vectors such as polymeric and nanoparticle-based materials,^15,16^ activatable cell-penetrating peptides (ACPPs) stand out because of their well-defined molecular composition. They can be prepared by modular solid-phase peptide synthesis (SPPS), which allows for precise tailoring with reporter groups, payloads, and modulating entities. Although activation of latent cell-penetrating peptides can also occur by nonenzymatic stimuli such as pH, reactive oxygen species, and light, the prototypical ACPP consists of an arginine-rich C-terminal sequence whose cell-penetrating capability is electrostatically attenuated by a polyanionic glutamate-rich sequence at the N-terminus, linked by a peptide chain that is recognized as a substrate by a specific protease.^17^ This type of ACPP was independently developed by the groups of Paul Wender and Roger Tsien.^18^ Tsien and coworkers used the sequence PLGLAG as a linker connecting hexa-d-glutamate and nona-d-arginine, which is hydrolytically cleaved between the residues glycine and leucine by matrix metalloproteinase-2 (MMP-2).^19^ Equipped with the fluorophore Cy5 as a reporter, its suitability for optical imaging of neoplastic tissue *in vivo* has been demonstrated for a HT-1080-derived murine xenograft model of human fibrosarcoma.^20^ Worth of note, this vector proved to be suitable for tumor drug delivery as its conjugation with the DNA damage checkpoint inhibitor AZD7762 resulted in effective radiosensitization in tumor-bearing mice, which was reflected by a significantly longer survival time compared to treatment with ionizing radiation alone. Moreover, attachment of the radiosensitizer to ACPP proved to be advantageous over antibody conjugation.^21^ The prominent function of the gelatinases MMP-2 and -9 in various diseases such as cancer, atherosclerosis, and cardiovascular disease has led van Duijnhoven et al. to label this construct with lutetium-177 at its C-terminus to enable SPECT imaging in the HT-1080 tumor model^22^ and in a mouse model of myocardial infarction.^23,24^ To allow for a more precise monitoring of proteolytic activation, the SPECT probe was additionally radiolabeled with iodine-125 at its N-terminus. Radiopharmacological evaluation of this probe in the HT-1080 fibrosarcoma model indicated that proteolytic MMP-2/-9-dependent activation in the vasculature preceded uptake of the probe in the tumor tissue, which was confirmed by comparative investigations in a mamma carcinoma model with low expression levels of the target enzymes.^25^ MMP-14, a further tumor-associated MMP of the transmembrane type (MT1-MMP) capable of activating pro-MMP-2, was targeted using a ^99m^Tc-labeled ACPP with SGRIGFLRTA as the substrate sequence by applying the design principle of Wender’s group, but no data on its *in vivo* behavior were reported.^26^

These findings suggest to exploit proteases other than MMP-2/-9, whose activity is more restricted to neoplastic tissue, for a more tumor-selective targeting with these promising vector molecules. A proteolytic enzyme whose elevated activity strongly correlates with tumor progression and metastasis is the cysteine protease cathepsin B.^27^ Under physiological conditions, this enzyme is located mainly in lysosomes, whereas its extracellular secretion is associated with disorders such as arthritis, fibrotic lung disease, neurodegeneration, and especially cancer.^28,29^ In particular, in addition to sorting into the lysosomes, procathepsin B can be released via Golgi-derived vesicles to the surface of tumor cells through the default secretory pathway, where it binds to the annexin II tetramer, which facilitates the processing of extracellular proteins including tissue-type plasminogen activator and type I collagen.^30–32^ Caveolin-1 in caveolae was identified as a further membrane-bound interaction partner of cathepsin B, which was found to be crucial for the degradation of the basement type IV collagen in the context of cell invasion.^33^ The secretion of cathepsin B was reported for several cancer cell lines^34^ as well as tumor-associated macrophages^35^ and its localization at the cell surface was demonstrated for lung tumor cells by both light^36^ and electron microscopy.^37^

In addition to cathepsin B, other cysteine cathepsins, such as cathepsins L, S, and K, whose substrate specificity partially overlaps with that of this enzyme, can be secreted by tumor cells and tumor-associated benign cells, even though to a minor extent.^35,38–40^

Theranostic targeting of tumor microenvironment-derived cathepsin B was demonstrated for a Cy5.5-conjugated designed ankyrin repeat protein (DARPin) recognizing both the pro and mature forms of the enzyme by optical imaging in a murine mammacarcinoma model based on the 4T1 cell line.^41^ Therefore, ACPPs containing cathepsin B-sensitive linkers hold great promise as imaging probes or vectors for cathepsin B-mediated delivery. In this regard, it should be pointed out that targeting of cathepsin B by substrates is challenged by the fact that this protease can act both as an exopeptidase, particularly a carboxydipeptidase, and as an endopeptidase. This unique feature of cathepsin B can be attributed to the presence of the occluding loop, comprising residues 102–128, which sterically restricts the access of endopeptidase substrates to the active site cleft and recognizes the terminal carboxylate of carboxydipeptidase substrates through charge-supported hydrogen bonds with His 110 and His 111. The commonly accepted view is that the carboxydipeptidase activity is favored at lower pH with the optimum at pH 4.5, while the endopeptidase activity of cathepsin B is predominantly observable at pH > 5.5 with the optimum around neutrality. This inverse relation can be rationalized by considering that the pH determines the protonation state of the vicinal histidine residues and therefore their capability to act as hydrogen bond donors.^42^ However, a recent study has revealed that the dual proteolytic behavior of cathepsin B is strongly influenced by the amino acid sequence of the particular substrate with no clear correlation between pH and its exo- and endopeptidase activity, and its action as a carboxydipeptidase predominates under both acidic and neutral conditions.^43,44^

The prevailing carboxydipeptidase activity makes the design of cell-penetrating peptides to be activated by cathepsin B-catalyzed cleavage challenging. Nevertheless, a variety of proteins has been identified to act as physiological endopeptidase substrates of cathepsin B,^45–47^ even though the structural requirements of peptidic substrates that favor endopeptidase activity remain largely unexplored. This applies particularly to the influence of the cathepsin B-substrate relationships beyond the P2′–S2′ interaction (standard protease substrate notation according to Schechter and Berger^48^), which should be critical for endopeptidase activity. Short peptide sequences that can be employed as linkers for cathepsin B-mediated delivery of reporter groups or therapeutic payloads conjugated to tumor-homing protein ligands were recently reviewed.^49–53^ Even though these linkers are efficient substrates of cathepsin B, their tumor selectivity relies largely on the protein ligand as exclusive targeting of the enzyme is mostly not achieved because of lacking substrate selectivity. Since these substrate linkers lack a terminal carboxylic group, their cleavage probably does not reflect carboxydipeptidase activity. However, as they do not extend beyond the S2′ region, they probably do not represent “true” endopeptidase substrates of cathepsin B either.

Regarding artificial carboxydipeptidase substrates, the hexapeptide GIVRAK was found to undergo highly efficient hydrolytic cleavage by cathepsin B as identified from a combinatorial library.^54^ Hence, the five N-terminal amino acid residues of this peptide can be considered as optimal P4–P1′ sites for cathepsin B’s carboxydipeptidase activity. We hypothesized that it might be possible to obtain efficient endopeptidase substrates of cathepsin B by C-terminal elongation and systematic variation of the terminal amino acid residue. With regard to their application, the substrate peptides were optimized for balanced proteolytic stability in human serum and efficient cathepsin B-catalyzed conversion. The most favorable sequence was inserted as a cathepsin B-responsive linker into the ACPPs by SPPS, which allowed for furnishing fluorophore- and copper-64-labeled conjugates that were suitable for molecular imaging at the cellular level by fluorescence microscopy and small-animal PET imaging, respectively. The integrated process of substrate identification and optimization, probe design, and characterization in cathepsin B-expressing human U87MG glioblastoma cells and a derived murine tumor xenograft model is reported herein.

## Results and Discussion

### Design, Synthesis, and Kinetic Characterization of Endopeptidase Substrates for Cathepsin B: Endopeptidase Activity Is Associated with Kinetic Hysteresis

The internally quenched hexapeptide Abz-GIVRAK­(Dnp)–OH (**1**), equipped with a 2-aminobenzoyl (Abz) group as a fluorescence donor and a 2,4-dinitrophenyl (Dnp) group as an acceptor (Figure 1), which was reported by Cotrin et al. as the optimal carboxydipeptidase substrate of cathepsin B,^54^ was selected as the starting point for the development of endopeptidase substrates. Therefore, its synthesis was established based on microwave-assisted SPPS at Wang-linker-functionalized polystyrene resin, which was loaded with commercially available Fmoc-Lys­(Dnp)–OH. Terminal 2-aminobenzoylation of the hexapeptide sequence was done with isatoic anhydride with good results. However, during the further course of this study, quinazoline-2,4-dione formation was occasionally observed during this step,^55^ which was avoided by using Boc-Abz–OH for terminal capping. In order to establish the Förster resonance energy transfer (FRET)-based cathepsin B assay, the N- and C-terminal cleavage products were also synthetically accessed. The synthesis of Abz-GIVR–OH (**1a**) was performed on the 2-chlorotrityl chloride resin, which facilitated the loading of Fmoc-Arg­(Pbf)–OH compared to the Wang resin. All other substrate peptides (Figure 1) were synthesized as C-terminal amides using the Rink amide resin, considering their prospective incorporation into ACPPSs. All peptides were predominantly obtained in moderate to good yields (5–91%) and in good chemical purity (≥90%). With the exception of compounds **4** and **63**, whose purities were 92% and 90.7%, respectively, the purity of the other final products was >95%.

**Figure 1:**
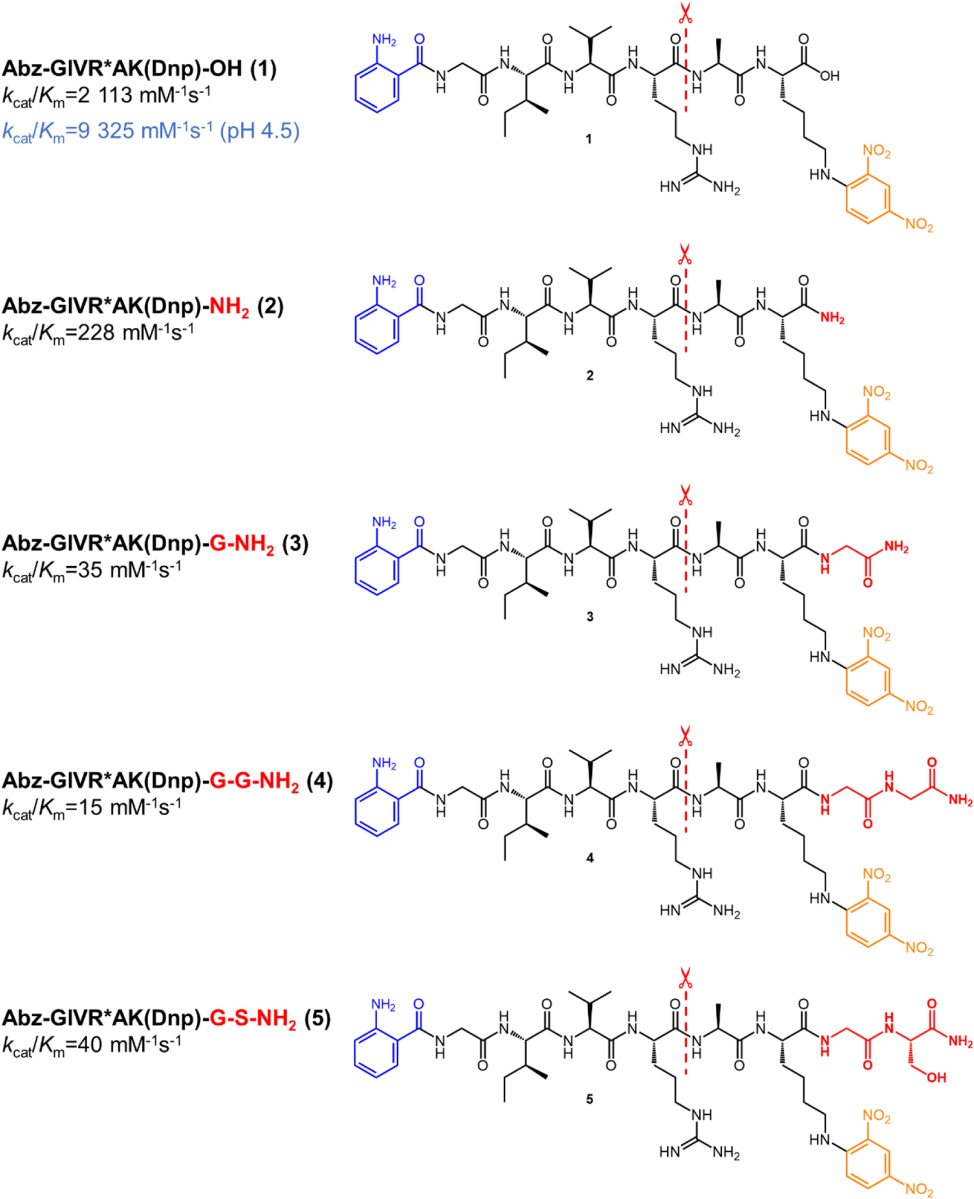
Design of cathepsin B endopeptidase substrates by C-terminal extension of the hexapeptide carboxydipeptidase substrate Abz-GIVRAK­(Dnp)–OH by P3′ and P4′ residues. The fluorescence donor Abz is highlighted in blue, the quenching Dnp moiety is shown in ocher, and the C-terminal extension beyond P2′ is shown in red. The cleavage site of the P1–P1′ peptide bond is each indicated by the dashed line. Unless otherwise indicated, performance constants are given for cathepsin B-catalyzed hydrolysis at pH 6.0.

For setting up the FRET-based assay for continuous monitoring of the cathepsin B-catalyzed cleavage of the peptidic substrates, the hexapeptide Abz-GIVRAK­(Dnp)–OH (**1**) and the cleavage products were spectrophotometrically characterized for absorption and emission spectra. Compared to hexapeptide **1**, the fluorescence intensity of tetrapeptide **1a** is 77-fold higher for excitation and emission at 320 and 416 nm, respectively, which corresponds to a FRET efficiency of 0.987.^56,57^ No difference in fluorescence intensity can be detected for isolated **1a** and the equimolar mixture of **1a** and **1b**, which indicates complete dequenching upon proteolytic cleavage. For accurate correlation between the concentration of the released product and the increase of fluorescence intensity, the inner filter effect needs to be considered for FRET-based substrates.^58^ To this end, the product fluorescence at a concentration of 5 μM for **1a**, which is a representative product concentration reached during the time range of the kinetic assay, was measured in the presence of unconverted substrate **1** in a concentration range up to 150 μM (see Figure S1 in Supporting Information). To exclude interference of potential substrate precipitation with the fluorescence measurements, the solubility range of each substrate compound was determined by recording UV/vis spectra at various concentrations, as a concentration-dependent increase at wavelengths around 600 nm indicates precipitation. Cathepsin B-catalyzed substrate conversion was measured for different substrate concentrations in a range restricted by the solubility limit of each peptide. Stability of the enzyme over the time of the measurement was confirmed on the basis of the Selwyn test.^59^ Based on the determined conversion rates at different substrate concentrations, the parameters *k*_cat_ and *K*_m_ were determined, from which the specificity constant *k*_cat_/*K*_m_ was calculated for each substrate, which herein will be referred to as the performance constant, in accordance to Koshland’s recommendation.^60^

For lead substrate **1** (Figure 1), a performance constant of 9325 mM^–1^ s^–1^ was determined at pH 4.5, which is close to the value of 7288 mM^–1^ s^–1^ reported by Cotrin et al.^54^ Increasing the pH to 6.0 resulted in an approximately 5-fold less efficient cathepsin B-catalyzed conversion of **1** (*k*_cat_/*K*_m_ = 2113 mM^–1^ s^–1^). Amidation of the C-terminus of **1** to **2** resulted in a *k*_cat_/*K*_m_ of 285 mM^–1^ s^–1^ at pH 4.5, which is about 30-fold lower compared to the free carboxylic acid. These structure/pH-kinetics relations reflect the importance of the putative salt bridge between the terminal carboxylate and the protonated imidazole rings of the adjacent His residue of the occluding loop. Notably, for the proteolytic cleavage of **2** the change in pH from 4.5 to 6.0 results in a significantly weaker attenuation in the performance constant by only a factor of 1.25 (data not shown). Similar relationships as obtained for hexapeptide **1** and the corresponding primary amide **2** were reported by Yoon et al. for analogous pairs of peptides.^43^ Extension of **2** by a C-terminal Gly residue to heptapeptide **3** resulted in an approximately 6-fold lower performance constant of 35 mM^–1^ s^–1^ at pH 6.0. This amino acid was selected because Gly occurs with high abundance in the P3′ position of cathepsin B-catalyzed cleavage products in a proteomic peptide library from HEK293 cell lysates, as shown in the study by Biniossek et al., who attributed this finding to a sterically restricted S3′ binding pocket and/or the requirement for conformational flexibility to minimize steric clash with the closed occluding loop.^46^ Further C-terminal extension by the identical amino acid residue to octapeptide **4** was not of significant influence on the catalytic performance with *k*_cat_/*K*_m_ = 15 mM^–1^ s^–1^. To improve the aqueous solubility of the octapeptidic substrate, the C-terminal Gly residue was replaced by Ser (compound **5**, Figure 1), which allows for introducing a hydrophilic hydroxy group with minimal steric demand of the side chain. Surprisingly, the introduction of this amino acid changed the kinetic behavior from a linear time course toward concavely curved graphs for product release, which means that the rates of cathepsin B-catalyzed conversion of **5** are increasing over time. This is indicative of hysteretic behavior, so the progress curves were analyzed according to the equation for enzyme hysteresis (eq 1) as derived by Frieden:

1 $${[P]}_{t}=v_{s}\times t-(\left(v_{s}-v_{0}\right)\times \frac{1-e^{-k_{obs}\cdot t}}{k_{obs}})+d$$

where [*P*]_*t*_ is the product concentration present at time *t*, *v*_s_ and *v*_0_ are the final steady-state and initial velocities, respectively, *k*_obs_ is the pseudo-first-order rate constant for reaching the steady state, and *d* is the background offset.^61^ The final steady state velocities *v*_s_ obtained in this way were analyzed for their dependence on substrate concentration, which allowed for calculating a performance constant of 40 mM^–1^ s^–1^, which is moderately higher than for substrate **3** bearing Gly in P4′. To obtain further information on the importance of the P4′ residue for the cathepsin B-substrate interaction, the amino acid residue in this position was systematically varied with all 20 proteinogenic amino acids for the octapeptidic scaffold containing the aminobenzoylated glycine residue (Table 1). Remarkably, all amino acid residues incorporated into the P4′ position different from glycine resulted in concave substrate conversion curves, as exemplarily shown for compound **11** (P4′ = Val) in comparison to compound **4** (P4′ = Gly) in Figure 2, indicating enzyme hysteresis. The most efficient conversion was observed for valine occupying the P4′ position with *k*_cat_/*K*_m_ = 245 mM^–1^ s^–1^. To further explore the scope and limitations of the obviously prominent P4′–S4′ interaction, nonproteinogenic amino acids representing structural analogues of valine were incorporated in the C-terminal position and the kinetic substrate properties of the corresponding octapeptides toward cathepsin B were investigated. The results are included in Table 1. The structure–activity relationships have revealed that valine in P4′ seems to be optimal for cathepsin B-catalyzed cleavage as slightly larger residues such as *tert*-leucine and cyclobutylglycine in this position led to significantly diminished performance constants (Figure 3A). A comparable effect was observed when one of valine’s methyl groups was omitted by incorporating 2-aminobutyric acid as the C-terminal residue. Considering the ratio of the performance constants of the alanine and valine derivatives **6** and **11**, respectively, the difference in the free substrate binding enthalpies can be calculated on the basis of a linear free-energy relationship, which yields a value of 1.32 kJ/mol. This value might reflect the energetic contribution of the two methyl groups, each providing 0.66 kJ/mol, which is in the reported range of 0.4–4 kJ/mol for the incremental binding energy per sp^3^-hybridized carbon atom.^62^ Furthermore, there seems to be a correlation between the substrate properties and the van der Waals surface areas for the aliphatic substituents in P4′ (Figure 3B). In particular, the performance constants increase starting from alanine over cyclopropylglycine (Cprg) to valine, while increasing the P4′ substituent beyond the isopropyl group decreases the *k*_cat_/*K*_m_ value. This finding suggests that the P4′–S4′ interaction is mainly determined by van der Waals contacts, which are most favorable for the valine side chain, while sterically more demanding residues will probably lead to clashes in the binding pocket. Noteworthy, the data point obtained for the octapeptide amide containing glycine in P4′ (compound **4**) is clearly lying out of the correlation, which points toward an alternate mode of substrate binding in this case and is in accordance with the missing hysteresis.

**Table 1:** Overview of Sequences for Peptides of Type Abz-G-P3-P2-P1-P1′-Lys­(Dnp)-P3′-P4′-CONH_2_, with the Exception of Compound **1** (Abz-GIVRAK­(Dnp)–OH), together with Their Substrate Properties (at pH 6.0) toward Cathepsin B and for Selected Peptides toward Cathepsins S, L, and K as Well as Half-Lives in Human Serum^a^

							*k*_cat_/*K*_m_ (mM^–1^ s^–1^)				
cpd	P3	P2	P1	P1′	P3′	P4′	Cat B	Cat S	Cat L	Cat K	*T*_1/2_ (min)
**1**	I	V	R	A	-	-	2113	n.d.	n.d.	n.d.	n.d.
**2**	I	V	R	A	-	-	228	n.d.	n.d.	n.d.	n.d.
**3**	I	V	R	A	G	-	35	n.d.	n.d.	n.d.	n.d.
**Abz-GIVRAK(Dnp)-G-P4′-CONH**_**2**_											
**4**	I	V	R	A	G	G	15	n.d.	n.d.	n.d.	n.d.
**5**	I	V	R	A	G	S	40	n.d.	n.d.	n.d.	3.7
**6**	I	V	R	A	G	A	152	n.d.	n.d.	n.d.	n.d.
**7**	I	V	R	A	G	2Abu	109	n.d.	n.d.	n.d.	n.d.
**8**	I	V	R	A	G	Nva	122	n.d.	n.d.	n.d.	n.d.
**9**	I	V	R	A	G	P	10	n.d.	n.d.	n.d.	n.d.
**10**	I	V	R	A	G	Nle	175	n.d.	n.d.	n.d.	n.d.
**11**	I	V	R	A	G	V	245	n.d.	n.d.	n.d.	n.d.
**12**	I	V	R	A	G	I	149	n.d.	n.d.	n.d.	n.d.
**13**	I	V	R	A	G	Tle	158	n.d.	n.d.	n.d.	n.d.
**14**	I	V	R	A	G	L	150	n.d.	n.d.	n.d.	n.d.
**15**	I	V	R	A	G	Cprg	231	n.d.	n.d.	n.d.	n.d.
**16**	I	V	R	A	G	Cbg	147	n.d.	n.d.	n.d.	n.d.
**17**	I	V	R	A	G	Cpeg	167	n.d.	n.d.	n.d.	n.d.
**18**	I	V	R	A	G	Phg	64	n.d.	n.d.	n.d.	n.d.
**19**	I	V	R	A	G	F	151	n.d.	n.d.	n.d.	n.d.
**20**	I	V	R	A	G	Y	126	n.d.	n.d.	n.d.	n.d.
**21**	I	V	R	A	G	H	71	n.d.	n.d.	n.d.	n.d.
**22**	I	V	R	A	G	W	78	n.d.	n.d.	n.d.	n.d.
**23**	I	V	R	A	G	T	93	n.d.	n.d.	n.d.	n.d.
**24**	I	V	R	A	G	Hse	79	n.d.	n.d.	n.d.	n.d.
**25**	I	V	R	A	G	C	135	n.d.	n.d.	n.d.	n.d.
**26**	I	V	R	A	G	M	181	n.d.	n.d.	n.d.	n.d.
**27**	I	V	R	A	G	D	19	n.d.	n.d.	n.d.	n.d.
**28**	I	V	R	A	G	E	23	n.d.	n.d.	n.d.	n.d.
**29**	I	V	R	A	G	N	104	n.d.	n.d.	n.d.	n.d.
**30**	I	V	R	A	G	Q	70	n.d.	n.d.	n.d.	n.d.
**31**	I	V	R	A	G	Orn	12	n.d.	n.d.	n.d.	n.d.
**32**	I	V	R	A	G	K	36	n.d.	n.d.	n.d.	n.d.
**33**	I	V	R	A	G	R	24	n.d.	n.d.	n.d.	n.d.
**Abz-GIV-P1-RAK(Dnp)-GS-CONH**_**2**_											
**34**	I	V	hArg	A	G	S	127	n.d.	n.d.	n.d.	2.9
**35**	I	V	nArg	A	G	S	9.7	n.d.	n.d.	n.d.	5.5
**36**	I	V	r	A	G	S	n.c.	n.d.	n.d.	n.d.	18
**37**	I	V	Cit	A	G	S	8.7	n.d.	n.d.	n.d.	8.9
**Abz-GFLGAK(Dnp)GS-CONH**_**2**_											
**38**	F	L	G	A	G	S	0.7	n.d.	n.d.	n.d.	16
**Abz-G-P3-P2-P1-P1′-Lys(Dnp)-Sar-P4′-CONH**_**2**_											
**39**	I	V	R	A	Sar	S	1.7	n.d.	n.d.	n.d.	39
**40**	I	V	R(ec)	A	Sar	S	1.4	n.d.	n.d.	n.d.	880
**41**	F	L	G	A	Sar	S	2.2	n.d.	n.d.	n.d.	1500
**42**	F	L	G	A	Sar	V	5.8	n.d.	n.d.	n.d.	1400
**43**	F	L	G	A	Sar	G	3.36	n.d.	n.d.	n.d.	n.d.
**44**	F	L	G	A	Sar	A	2.13	n.d.	n.d.	n.d.	n.d.
**45**	F	L	G	A	Sar	L	1.40	n.d.	n.d.	n.d.	n.d.
**46**	F	L	G	A	Sar	F	5.79	n.d.	n.d.	n.d.	n.d.
**47**	F	L	G	A	Sar	Y	1.60	n.d.	n.d.	n.d.	n.d.
**48**	F	L	G	A	Sar	E	2.02	n.d.	n.d.	n.d.	n.d.
**49**	F	L	G	A	Sar	Q	2.41	n.d.	n.d.	n.d.	n.d.
**50**	F	L	G	A	Sar	Orn	1.87	n.d.	n.d.	n.d.	n.d.
**51**	F	L	G	A	Sar	K	0.68	n.d.	n.d.	n.d.	n.d.
**52**	F	ACBC	G	A	Sar	V	n.c.	n.d.	n.d.	n.d.	n.d.
**53**	L	F	G	A	Sar	V	2.6^†^	8.9	112	920	n.d.
**54**	L	F(3-Me)	G	A	Sar	V	1.8^†^	4.4^†^	131^†^	180	n.d.
**55**	L	F(3-I)	G	A	Sar	V	1.9^†^	1.8^†^	107	34^†^	n.d.
**56**	F	L	Cit	A	Sar	V	7.1^†^	42^†^	46	394^†^	n.d.
**57**	F	F	Cit	A	Sar	V	2.1^†^	n.d.	n.d.	n.d.	n.d.
**58**	F	V	Cit	A	Sar	V	3.1^†^	n.d.	n.d.	n.d.	n.d.
**59**	F	ACBC	Cit	A	Sar	V	0.6	n.d.	n.d.	n.d.	n.d.
**60**	L	F(3-Me)	Cit	A	Sar	V	4.8	12^†^	26	395	n.d.
**61**	I	V	Cit	A	Sar	V	1.22^†^	n.d.	n.d.	n.d.	n.d.
**62**	I	ACBC	Cit	A	Sar	V	n.c.	n.d.	n.d.	n.d.	n.d.
**63**	I	V	R(ec)	A	Sar	V	3.4^†^	16^†^	21	68	n.d.
**64**	I	F(3-Me)	R(ec)	A	Sar	V	3.1	1.4^†^	75	23^†^	n.d.
**65**	I	F(3-I)	R(ec)	A	Sar	V	0.5	n.c.	2^†^	n.c.	n.d.
**66**	F	L	G	F	Sar	V	3.0	6.7	50	283	n.d.
**67**	F	L	Cit	F	Sar	V	n.c.	n.d.	n.d.	n.d.	n.d.

a †Indicates values derived from Hanes–Woolf linearization. Nonstandard AA-abbreviations: 2-Abu, l-2-aminobutyric acid; ACBC, 1-aminocyclobutanecarboxylic acid; Arg­(ec)/R­(ec), *N* ^ω^-(ethylcarbamoyl)-l-arginine; Cbg, l-cyclobutylglycine; Cpeg, l-cyclopentylglycine; Cprg, l-cyclopropylglycine; Hse, l-homoserine; Nle, l-norleucine; Nva, l-norvaline; Orn, ornithine; Phg, l-phenylglycine; Sar, sarcosine; Tle, *tert*-leucine; n.c., no cleavage; n.d., not determined. The concentration ranges for all substrates are included in Table S1 and the determination of the solubility range is exemplarily shown for compounds **5**, **11** and **37** in Figure S2, each in Supporting Information.

**Figure 2:**
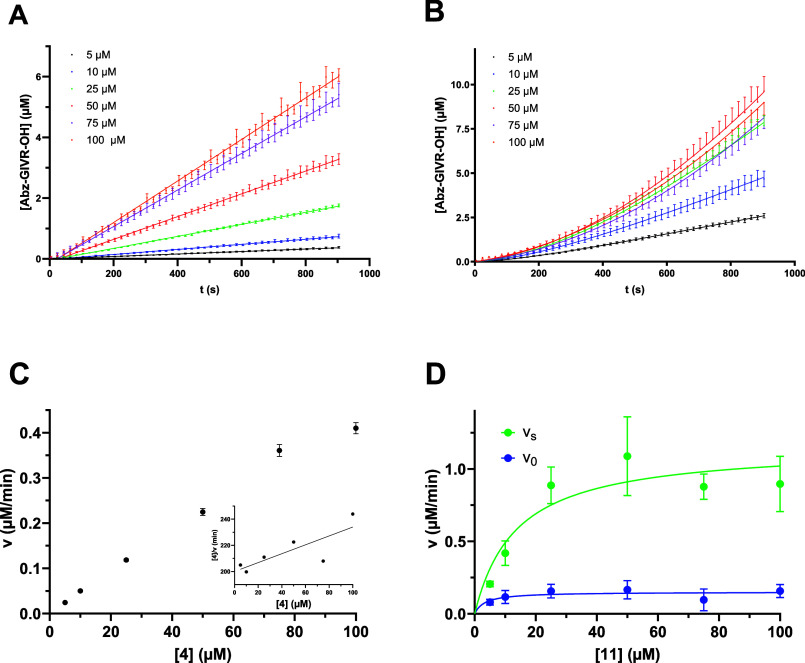
Amino acid residues different from Gly in the P4′ position lead to kinetic hysteresis as exemplarily illustrated by fluorimetric monitoring of cathepsin B-catalyzed hydrolytic conversion of Abz-GIVRAK­(Dnp)­GG-NH_2_ (**4**; **A**) and Abz-GIVRAK­(Dnp)­GV-NH_2_ (**11**; **B**) each to fluorescent Abz-GIVR–OH at 30 °C and pH 6.0. Data sets were averaged from three independent experiments (*n* = 3) with duplicate measurements for each. Data are plotted with SEM. Fluorescence intensities were corrected for the inner filter effect, the background fluorescence intensities at the start of the reaction were subtracted, and values were transformed into product concentrations. (**C**) Replot of the steady-state rates (*v*) against the concentration of substrate **4**. As substrate saturation was not reached within the investigated concentration range, nonlinear regression was omitted, and *V*_max_ and *K*_m_ were determined by linear regression in the Hanes–Woolf plot (inset). (**D**) Replot of the initial (*v*_0_) and final steady-state rates (*v*_s_) against the concentration of substrate **11** and fitted graphs for determination of *V*_max_ and *K*_m_.

**Figure 3:**
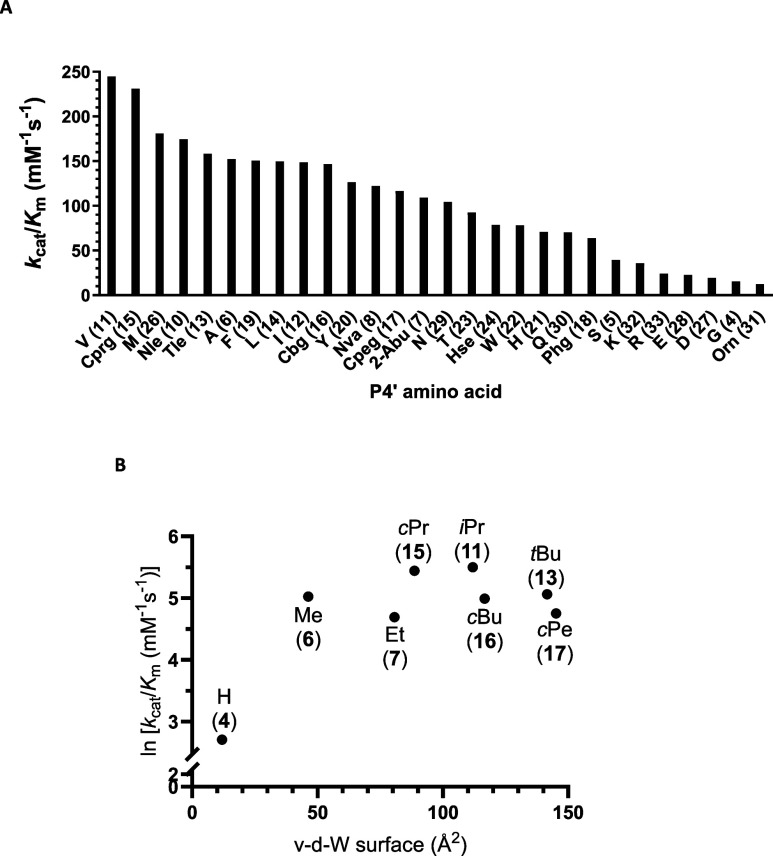
(**A**) Order of octapeptides of type Abz-GIVRAK­(Dnp)­GX-NH_2_ from high (left) to low (right) performance constants for the proteolytic cleavage by cathepsin B. X indicates the varied P4′ position. See the caption of Table 1 for abbreviations of nonproteinogenic amino acids. (**B**) Correlation of performance constants with the van der Waals (v-d-W) surface of the P4′ side chain for selected aliphatic and cycloaliphatic residues. Acronyms for the substituents at each data point denote the alkyl side chain each attached to the C_α_ of residue X.

To the best of our knowledge, hysteretic behavior for cathepsin B-catalyzed hydrolysis, apart from its time-dependent reductive activation by DTT,^63^ has not been reported so far. In general, hysteresis in the context of enzyme catalysis refers to a slow response toward the exposure of substrates or other ligands, which manifests in either a “lag” or “burst” in the rate of the catalyzed reaction.^61,64^ As pointed out by Neet and Ainslie, the slow transition reflects “a conformational change (isomerization), a dissociation-association reaction of the enzyme, or a direct displacement of a bound ligand by a second ligand”.^65,66^ Considering the well-known importance of the occluding loop in the regulation of the endopeptidase activity of cathepsin B,^44^ the observed lag phases in the conversion of the octapeptides with P4′ ≠ Gly might reflect the conformational transition (isomerization) between the “open” and “closed” states of the occluding loop. Kinetic information on the observed slow transition in cathepsin B-catalyzed substrate conversion can be obtained by analyzing the obtained data of the progression curves according to eq 1 which allows for obtaining the pseudo-first-order rate constants *k*_obs_. The formalism for the mathematical description of substrate conversion in the presence of enzyme isomerization from a catalytically inactive state into the fully active state was, to the best of our knowledge, exclusively provided by Antonio Baici within his seminal monograph “Kinetics of enzyme-modifier interactions” (Scheme 1). The meaning of the *k*_obs_ values in the context of this kinetic mechanism and their relation to substrate concentration and elementary rate constants is given in eq 2. The further relationships used for data evaluation are included in eqs 3 and 4.^67^ The meanings of the rate constants are explained in Scheme 1.

**Scheme 1:**
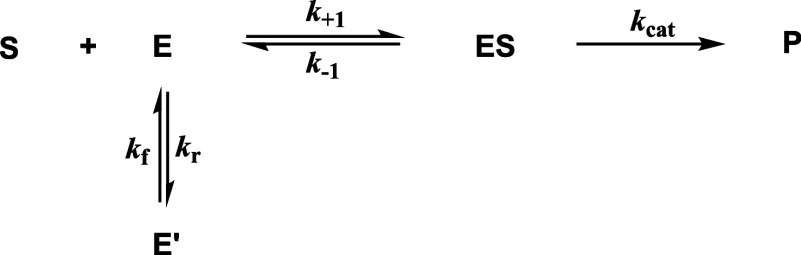
Kinetic Chart for Enzyme-Catalyzed Substrate Conversion in the Presence of Slow Transition between an Inactive (*E*’) and Fully Active Enzyme Isoform (*E*)


2 $$k_{obs}=\frac{k_{r}}{1+\frac{\left[S\right]}{K_{m}^{\prime }}}+k_{f}$$

*k*_obs_: pseudo-first-order rate constant for reaching the steady state included in eq 1;

*k*_f_: first-order rate constant for the isomerization of the enzyme from the inactive to the active state (Scheme 1);

*k*_r_: first-order rate constant for the isomerization of the enzyme in the reverse direction;

[*S*]: substrate concentration;

*K*_m_′: Michaelis constant for the initial enzyme equilibrium.

3 $$K_{eq}=\frac{\left[E^{\prime }\right]}{\left[E\right]}=\frac{k_{r}}{k_{f}}$$

*K*_eq_: equilibrium constant for enzyme isomerization;

[*E*′]: equilibrium concentration of the inactive enzyme form;

[*E*]: equilibrium concentration of the active enzyme form;

*k*_r_, *k*_f_: see eq 2.

4 $$\frac{V_{max\left(s\right)}}{V_{max\left(0\right)}}=1+K_{eq}$$

*V*_max(s)_: maximum velocity for final steady-state rates;

*V*_max(0):_ maximum velocity for initial rates;

*K*_eq_: see eq 3.

The *k*_obs_ values are included in the progress curves of substrate conversion and can be obtained by nonlinear regression according to eq 1. However, this is challenging because the virtual steady states are attained at time points beyond the optimized measurement times, particularly at higher substrate concentrations. Nevertheless, for some octapeptidic substrates with P4′ ≠ Gly, the dependence of the *k*_obs_ values on the substrate concentration could be computationally analyzed by robust nonlinear regression. In particular, data from 7 compounds were evaluated (compounds **7**, **11**, **21**, **23**, **24**, **29**, and **30**; P4′ = 2-Abu, Val, His, Thr, Hse, Asn, and Gln, respectively). All *k*_obs_ values declined hyperbolically with increasing substrate concentrations and approached asymptotically a minimal limit representing *k*_f_ (see Figure S3A in Supporting Information). Computational analysis by nonlinear regression according to eq 2 using the *K*_m_′ value determined from analysis of the determined initial velocities as a constraint yielded *k*_r_ values ranging between 0.0015 s^–1^ and 0.0059 s^–1^ with a mean value of 0.0029 s^–1^ (±0.0006 s^–1^, SEM, *n* = 7). As expected, a correlation between the distinct obtained *k*_r_ values and the kind of the varying P4′ side chain, which included aliphatic, aromatic, as well as polar residues, was not obvious, and the distinct rate constants for each compound varied within the error margins (see Figure S3B in Supporting Information). The *k*_f_ values obtained in this way ranged between 0.0003 and 0.0015 s^–1^ with a mean value of 0.0009 s^–1^ (±0.0002 s^–1^, SEM, *n* = 7). The distinct values for *k*_f_ were somewhat more error-prone than the *k*_r_ values. Therefore, the *k*_f_ values were alternatively calculated from the division of *k*_r_ by the corresponding equilibrium constant *K*_eq_ defined by eq 3. This parameter is included in the ratio of the maximum velocities for the initial and steady rates according to eq 4.^67^ The values obtained for *V*_max(s)_/*V*_max(0)_ ranged between 3.75 and 7.83 (see Figure S3C in Supporting Information) with a mean value of 5.33 (±0.51, SEM, *n* = 7), which yields *K*_eq_ = 4.33 (±0.51, SEM, *n* = 7) equaling the concentration ratio between the inactive and active cathepsin B species. This allows for computation of *k*_f_ according to eq 3 to 0.0007 s^–1^. These first-order rate constants for isomerization of cathepsin B provide unprecedented insight toward the conformational dynamics of its occluding loop, as mean life times of 23.8 and 5.7 min for the closed and open states of the occluding loop can be derived from the reciprocal values of the rate constants, respectively. The determined rate constants are in the range of the time scale observed for the movement of large domains in multidomain enzymes such as the conformational change from the compact closed to the extended open state in human transglutaminase 2.^68^ However, the conformational transitions of lid elements that regulate the substrate access to the binding site in other monomeric enzymes similar to cathepsin B’s occluding loop, typically operate at considerably smaller time scales.^69^ For example, the movement of the lid subdomain in adenylate kinase, which with 43 residues is about twice as large as the occluding loop in cathepsin B, occurs in the range of milliseconds.^70^

### Molecular Docking of Selected Substrates to Cathepsin B: Residues Larger Than Gly in P4′ Require an Open Occluding Loop

To obtain clues about the structural basis of both kinetic hysteresis and structure-velocity relationships for varying P4′ residues, for which a clear preference for relatively small hydrophobic side chains was observed, the 3D structures of octapeptides **4** and **11** were modeled, and their binding to cathepsin B was investigated by molecular docking. For this purpose, available crystallographic structures of cathepsin B representing different conformational states of the occluding loop (i.e., closed (PDB ID 1GMY, 1.9 Å)^71^ and open (PDB ID 3PBH, 2.5 Å)^72^) were used (see Methods). The structure corresponding to the propeptide residues 39-KRLCGTFL-46 of procathepsin B (PDB ID 3PBH), which occupy the substrate-binding site in reverse orientation, was used as a template to model the substrate compounds. This molecular template was manually reoriented on the cathepsin B structure along the *y*-axis toward the substrate-binding site. The modeling of the substrates’ secondary structures included dihedral angle scans as a first approximation to accomplish maximum alignment of the substrate residue side chains, especially at positions P2 and P4′, to the cathepsin B propeptide residues Thr^P^44 (S2 site) and Leu^P^41 (S2′), respectively, while also avoiding steric clashes with the protein (see Methods). In the aligned arrangement of substrate and propeptide, the spatial deviations of corresponding side-chain atoms in the respective residues (C_β_ of Val at P2 in modeled substrates (**4** and **11**) and C_β_ of Thr^P^44, and C_γ_ of Val at P4′ in substrate **11** and C_δ_ Leu^P^41) were 0.7 and 0.9 Å, respectively (Figure S4A in Supporting Information). This resulted in a starting structure in extended conformation from residues P2 to P2′. Interestingly, modeling of the substrate **4** using AlphaFold3,^73^ which does not take into account the functionalizations of Lys­(*N* ^ε^) at P2′ as well as those at the N- and C-termini, predicted a similar extended structure from residues P2 to P3′ with high confidence (Figure S4B,C in Supporting Information). While both molecules **4** and **11**, as expected, can bind to the open-loop conformation of cathepsin B (Figure 4A and B), there are crucial differences with regard to the number of intermolecular contacts in the respective complexes. In the top 10-ranked modeled interactions of compounds **4** and **11** with the open-loop conformation of cathepsin B (Figure 4A and B, see Methods), compound **11** was predicted to establish three H-bonds with the backbone of cathepsin B residues Gly24, Gly74, and Met196 (Figure 4A), whereas for compound **4** a single H-bond was predicted between the *ortho* nitro group of Lys (Dnp) and His111 at the occluding loop of cathepsin B (Figure 4B). Interestingly, compared to molecule **4**, where a Gly residue occupies position P4′, molecule **11** showed larger van der Waals contacts between the Val side chain at this position and Trp221 (Figure 4A). Based on these observations, this residue might be postulated as a key structural element in the recognition of the substrate’s P4′ side chain. The side chain of Trp221 is conformationally flexible to a certain extent, which might allow for the accommodation of different alkyl residues, which is in agreement with the experimental results discussed above in the context of Figure 3B. Furthermore, the spatial orientation of the isopropyl chain against the indole ring indicates that the enzyme–substrate recognition at this site relies mainly on CH−π interactions, whose strong dispersive character^74,75^ is in line with the observed incremental energy contributions mentioned in the previous section.

**Figure 4:**
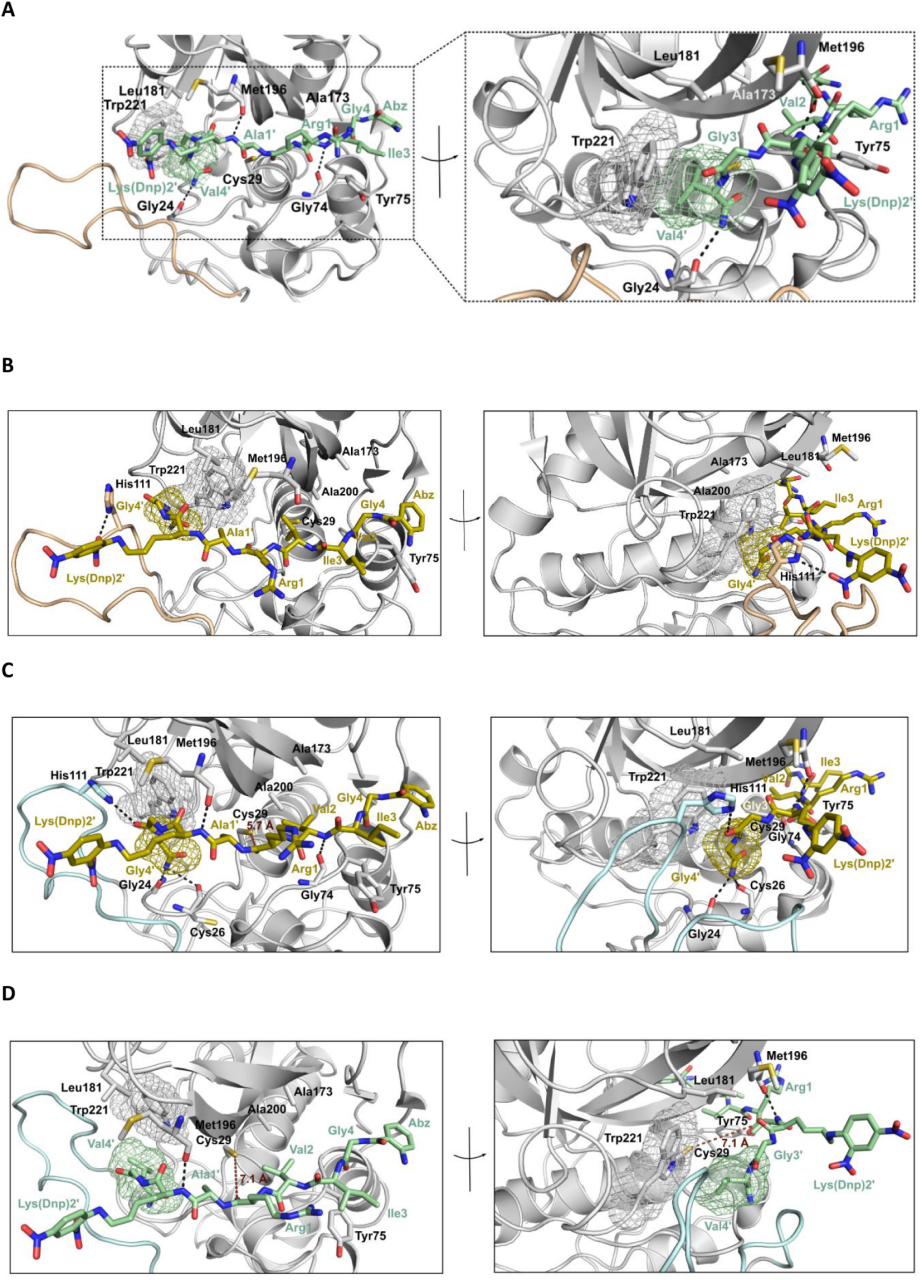
Modeling of cathepsin B in complex with substrates **11** (**A** and **C**) and **4** (**B** and **D**). Cathepsin B (occluding loop in open conformation (**A** and **B**), PDB ID 3PBH, 2.5 Å and closed conformation (**C** and **D**), PDB ID 1GMY, 1.9 Å) is shown in gray cartoon, and the occluding loop in open and closed orientation is each highlighted in pale and cyan, respectively. Interacting residues are highlighted in sticks, colored by atom type and labeled. Substrates **11** and **4** are shown in green and olive sticks, respectively, colored by atom type and labeled. The surface mesh denotes van der Waals contacts between Trp221 of cathepsin B and substrate residues at P4′ (Val4′ and Gly4′, respectively). H-bonds are depicted with black dashed lines. Figure created with PyMOL v.2.4.1.

Valuable conclusions regarding substrate recognition can furthermore be drawn from the crystal structure of procathepsin B (PDB ID 3PBH). In this structural assembly, the section Leu^p^41 to Phe^p^45 of the propeptide adopts an extended β-strand-like conformation, and the corresponding side chains are accommodated each by the binding sites S2′ to S3. Leu^P^41 is occupying the S2′ binding pocket, which is defined by the residues Trp221, Trp225, and Phe180 (distance to Trp221: 3.4 Å between C_γ_ of Leu^p^41 and indole nitrogen).^72^ A two-turn helical segment is located upstream of Leu^p^41. Considering the topological relationships between the β-strand and α-helical conformation, according to which the C_α_ atoms of the residues *i*, *i*+2 and *i*, *i*+3/4, respectively, are approximately equidistant,^76^ Leu^p^38 should correspond to the substrate’s P4′ residue. However, the side chain of Leu^p^38 appears to be partially solvent-exposed rather than accommodated by a certain binding site. Considering this fact in combination with the modeled **11**–cathepsin B complex suggests that the substrate’s P4′ Val residue occupies the S2′ binding site. Therefore, molecular recognition in the S2′ subsite seems to converge on the isopropyl groups of Leu^P^41 and Val in P4′ of the propeptide and substrate **11**, respectively, and the model of the **11**–cathepsin B complex (Figure 4B) suggests a plausible rationale for the experimental findings. The P4′–S2′ positional shift is likely a consequence of the long side chain of the P2′ Lys­(Dnp) side chain in **11** and its bulky dinitrophenyl group, which cannot be adopted by cathepsin B’s corresponding S2′ binding pocket, and, therefore, forces the residue to turn out of the binding cleft so that the P4′ Val residue can bend into the S2′ pocket supported by the conformationally flexible Gly residue in P3′. This interpretation is in accordance with the generally accepted view that subsites beyond S2′ in papain-like cysteine proteases do not exist.^77,78^ However, experimental data on cathepsin B–substrate interactions, which are compiled and curated in the MEROPS database, suggest that the enzyme’s specificity clearly covers the positions P3′ and P4′,^79^ which might indicate the general significance of the concluded residue-subsite shift.

Modeling of the recognition of substrates **4** and **11** by cathepsin B with the occluding loop in a closed conformation revealed that both octapeptides could, in principle, bind to the enzyme’s substrate-binding cleft (Figure 4C and D). However, compound **4** containing Gly in P4′ is predicted to bind substantially deeper into the cleft compared to substrate **11** bearing Val in this position. Notably, the modeling results suggested that the P3′ carbonyl oxygen in **4** is hydrogen-bonded to the imidazole NH of His111, the primary amide in P4′ acts as a hydrogen bond donor toward the carbonyl oxygen atoms of Gly24 and Cys26, and the P2 amide nitrogen is hydrogen-bonded to the carbonyl oxygen of Gly74 (Figure 4C). Conversely, for compound **11**, only the backbone amide nitrogen in P2′ was predicted to be hydrogen-bonded to the carbonyl oxygen of Met196 (Figure 4D). The distance between the sulfur atom of the active-site Cys29 and the P1 carbonyl carbon atom is more than 1 Å greater for **11** (5.7 Å for **4** vs 7.1 Å for **11**; Figure 4C and D). In this context, it is worth mentioning that for the ion–dipole complex along the reaction path between methanethiolate and *N*-methylacetamide—used as a model system for cysteine-dependent proteolysis—a sulfur–carbon distance of 4.33 Å was calculated on the basis of a QM/MM study.^80^ It could therefore be argued that the binding of compound **11** to the closed-loop conformer is not productive enough to enable sufficiently close contacts for the formation of the covalent complex with cathepsin B to cleave the P1–P1′ peptide bond. In addition, the predicted binding poses provide insight into why Gly in P3′ is pivotally preferred by cathepsin B, as observed by Biniossek et al.^46^ On the basis of their results, it remained open whether this is due to steric restriction in the S3′ pocket or the conferred conformational flexibility by Gly, which allows for bending away from the closed occluding loop. The modeling studies herein suggest that this particular binding site is completely occupied by the side chains of Leu181 and Met196, which, independently of the occluding loop orientation, do not allow for the accommodation of substituents beyond the small hydrogen atom (see Figure S5 in Supporting Information).

The results from the docking studies suggest that cathepsin B with a closed occluding loop is endopeptidase-active if two adjacent Gly residues are located in P3′ and P4′. The observed hysteretic conversion of substrates P4′ ≠ Gly was explained in the previous section by assuming that the closed occluding loop conformation of cathepsin B cannot process these substrates and a conformational transition must therefore precede catalysis. The modeling studies support this hypothesis by establishing a plausible structural basis for the differential binding modes of octapeptides **4** and **11.** Furthermore, the rather simplistic view that the closed occluding loop confers exclusive carboxydipeptidase activity to cathepsin B because the access of residues beyond P2′ is sterically blocked should perhaps be reconsidered. Together with the observed kinetic hysteresis for true endopeptidase activity of cathepsin B, the previous conclusion that the endopeptidase activity of this protease is an evolutionary remnant resulting from the conformational flexibility of the occluding loop could be questioned.^81^ Our results suggest that this unique structural element may have rather evolved for structural and kinetic fine-tuning of substrate proteolysis that might be of (patho)­physiological relevance.

### Stabilization of Cathepsin B Endopeptidase Substrates against Proteolytic Cleavage in Human Serum: The P1–P1′ and P2′–P3′ Bonds Are the Major Sites for Metabolic Degradation

The intended application of the identified endopeptidase substrates as linkers for targeted delivery requires sufficient stability against premature proteolytical cleavage in the circulation. For this purpose, octapeptide **5**, which shows favorable kinetic properties with regard to endopeptidolytic cleavage by cathepsin B, was incubated in human blood serum, and its degradation was monitored by LC/ESI-MS at different time points, using the absorbance of the dinitrophenyl group at 365 nm to calculate the proportion of intact octapeptide and the *m*/*z* ratio to identify the cleavage products (shown exemplarily for compounds **5**, **35**, and **36** in Figures S7–S9 in Supporting Information). As shown in Table 1, compound **5** undergoes a rapid degradation in human serum with *t*_1/2_ = 3.72 min (see Figure S6 in Supporting Information), which was mainly attributed to hydrolytic cleavage of the P1–P1′ peptide bond after the Arg residue. This finding does not surprise in respect to the abundance of serine proteases in blood serum with trypsin-like substrate specificity such as plasmin, thrombin and kallikrein.^82,83^ To attenuate the cleavage of this peptide bond, analogues of arginine were introduced including derivatives with inverted configuration and extended or shortened chain length, such as homoarginine and norarginine (compounds **34** and **35**, Table 1). Exchanging arginine against the former analogue resulted in a 4-fold extension of the half-life in human plasma for a 31mer derivative of calcitonin gene-related peptide (CGRP)^84^ Similarly, substituting arginine against the latter analogue with shortened side chain in a factor XIIa-inhibiting bicyclic peptide increased its plasma half-life from 4 to 22 h.^85^ Replacement of l-arginine by its d-configurated analogue, as exerted in peptide **36**, is a proven strategy for improving the metabolic stability of peptides.^86^ Furthermore, the uncharged Arg analogue citrulline, which was extensively employed in cathepsin B-responsive linkers,^52,87,88^ was introduced in P1 with the intent of stabilization. The sequentially more distant P1–P4 motif GFLG was chosen on the basis of results obtained for cathepsin B-catalyzed cleavage of chromogenic peptides attached to *N*-(2-hydroxypropyl)­methacrylamide (HPMA)-based copolymers.^89^ This sequence was also employed for tumor targeting of PEG-Doxorubicin conjugates,^90^ in the design of enzymatically cleavable radioimmunoconjugates^91^ and antisense oligonucleotides^92^ and furthermore in probes for cathepsin B-mediated optical imaging and photodynamic therapy.^93^ Although the replacement of the Arg residue in P1 can significantly prolong the half-life in serum, e.g., to 18 min for the d-Arg-containing analogue **36**, no sufficient stability for application *in vivo* was achieved by this approach. Analysis of degradation products by LC-MS indicated proteolysis after the P3′ Gly residue as an additional cleavage site besides the P1–P1′ peptide bond. Therefore, *N*-methylation of the P3′ Gly residue was attempted, which resulted in a serum half-life of >24 h when combined with Ser­(BnCOOH) in P1 or GFLG as nonprimed site tetrapeptide sequence, which can be classified as highly stable considering the acyclic nature of the peptide.^94^ In contrast, the half-life was only 39 min for the *N*-methylated octapeptide bearing the GIVR N-terminal moiety. As expected, substituting Arg by different residues in P1 diminished or even abolished (compound **36**) the substrate potential toward cathepsin B with the exception of hArg, as compound **34** exhibits a performance constant of 127 mM^–1^ s^–1^, compared to 40 mM^–1^ s^–1^ for the reference substrate **5**. Changing the N-terminal substrate moiety to GFLG (compound **38**) significantly reduced the substrate potential, which, surprisingly, was somewhat improved upon combination with the *N*-methylated P_2_′–P_3_′ peptide bond (compound **41**). In the case of GIVR as a nonprimed sequence, this modification was clearly detrimental (compound **39**), as expected considering the importance of hydrogen bond networks in protease-substrate interactions.^95^ In combination with *N*-methylation, the arginine residue in the IVR motif was also *N* ^ω^-ethylcarbamylated (Arg­(ec), compound **40**). For this purpose, the building block Fmoc-Arg­(ec,Boc)–OH was synthesized and employed for SPPS (see Scheme S1 and associated text in Supporting Information for details). Carbamoylation of the guanidino group was found to be beneficial in arginine-containing peptidic receptor ligands.^96^ In the present case, ethylcarbamoylation resulted in a *k*_cat_/*K*_m_ for the cathepsin B-catalyzed conversion that was similar to that for the peptide with unsubstituted Arg, while this modification increased the half-life in human serum by a factor of >20 (Table 1, compare **39** and **40**).

Structure–stability relationships in comparison to performance as cathepsin B substrates for selected octapeptide amides are summarized in Figure 5. Variation of the P4′ residues by selected proteinogenic amino acids beyond Ser and Val in the metabolically stabilized octapeptide containing the GFLG motif largely confirmed the SAR trend observed in the GIVR-derived substrates regarding the kinetic efficiencies (see Table 1 and Figure S10 in Supporting Information).

**Figure 5:**
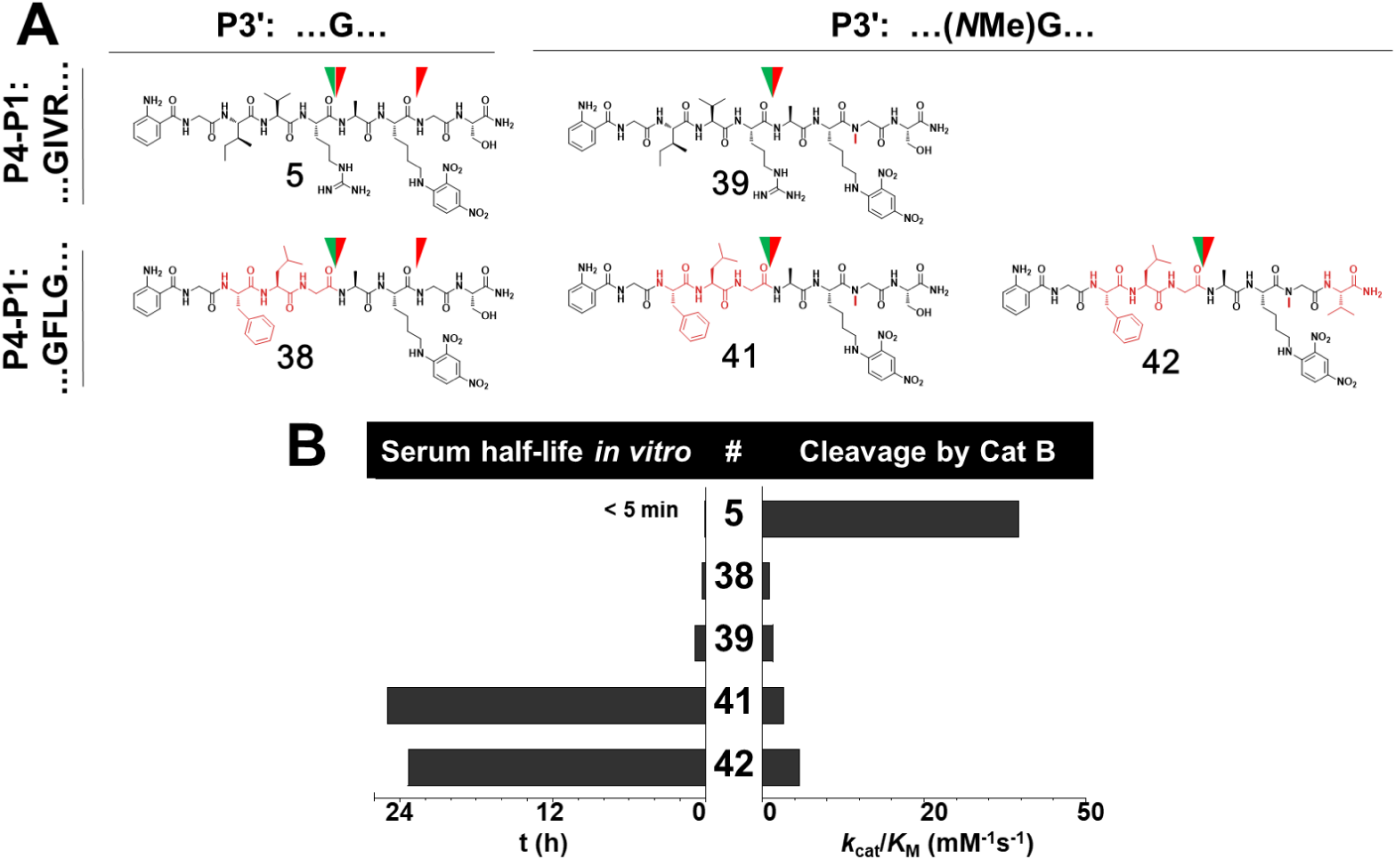
Summary of structure–stability/activity relationships between stability in human serum and cathepsin B-catalyzed conversion. (**A**) Structures of selected octapeptides. Green half-arrowheads indicate the cathepsin B cleavage site, and red half-arrowheads indicate the unspecific cleavage sites of human serum proteases identified by LC-ESI-MS fragment analysis. (**B**) Histogram plots for half-life in human blood serum versus performance constants for cathepsin B-catalyzed conversion.

### Substrate Selectivity against Cathepsins S, L, and K: Octapeptides Optimized for Cathepsin B and Stability Are Also Cleaved by Other Tumor-Associated Cysteine Cathepsins

Considering the broad and overlapping substrate specificity of the papain-like mammalian cathepsins, as confirmed in a recent study,^47^ the conversion of the octapeptidic cathepsin B substrate **44** catalyzed by the human cysteine cathepsins S, L, and K was investigated (Table 1). As is obvious, cleavage by the considered cathepsins occurs more efficiently in comparison to cathepsin B. To explore whether selectivity toward conversion by cathepsin B can be achieved, the P3–P1′ residues of the octapeptidic scaffold were varied in a partially iterative manner, while the N-terminal Gly residue and the C-terminal P2′–P4′ moiety were kept constant to maintain proteolytic serum stability. The non-natural residues introduced into the P2 position were selected based on their beneficial performance in dipeptide alkyne-derived inhibitors (*meta*-methyl- and *meta*-iodophenylalanine, compounds **54**, **55**, **60**, **64**, and **65**, Table 1) and in a cathepsin B-responsive linker for antibody-drug conjugation (1-aminocyclobutane carboxylic acid (ACBC), compounds **52**, **59**, and **62**),^59,87,97^ respectively. Considering the presence of citrulline in multiple cathepsin B-cleavable linkers, Gly in P1 of compound **44** was replaced by this residue (compound **56**) and the combinations with selected P2 residues different from Leu (compounds **57**-**59**) were tested. Citrulline in P1 was also tried as a replacement in the IVR motif (compounds **61** and **62**). The arginine residue in this tripeptide sequence was also replaced by Arg­(ec) (compounds **63**-**65**). The GFLG motif was furthermore combined with Phe in P1′ (compound **66**), also together with citrulline in P1 (compound **67**), as aromatic residues in this position were reported as favorable for cathepsin B-catalyzed conversion.^77,98^ Leu and Ile besides Phe in P3 and Val in P2 were taken into focus because these residues in the particular positions were found to have a beneficial influence in oligopeptidic carboxydipeptidase substrates.^98^

The kinetic performance of the particular octapeptides is included in Table 1. In almost all combinations of octapeptides and cathepsins, the cleavage by cathepsin B appears to be less efficient in comparison to cathepsins L, S, and K. This general trend can be explained by the occluding loop of cathepsin B, which sterically restricts the access to the substrate binding site also in the case of open orientation.^81,98^ The fastest reaction was mostly observed with cathepsin K with performance constants in the three-digit range (mM^–1^ s^–1^) followed by cathepsin l-catalyzed cleavage, apart from a few exceptions. The extent of conversion by cathepsin S was more comparable to that of cathepsin B but was also mostly faster than with the target enzyme, except for the substrates **55**, **65**, and **66** (Table 1). Switching the positions of Phe and Leu in the GFLG motif (compound **53**) attenuated cleavage by cathepsin B. Unexpectedly, the introduction of ACBC into P2 abolished (compounds **52** and **62**) or strongly diminished (compound **59**) substrate conversion by cathepsin B. The change from Gly in P1 to Cit in the GFLG motif slightly slowed down the conversion by the other cathepsins but did not significantly improve cleavage by cathepsin B (compare **44** and **56**). Further combinations of P2 and P3 residues with Cit in P1 did neither accelerate the conversion by cathepsin B nor improve the selectivity profile (compounds **57**-**62**). Remarkably, the introduction of *N* ^ω^-ethylcarbamoylated Arg into the P1 position of the GIVR motif (compound **63**) strongly attenuated cleavage by cathepsin K and in combination with Phe­(3-I) in P2 even abolished the conversions catalyzed by cathepsins S and K. However, the conversion by cathepsin B was either impeded upon the introduction of this residue. Changing Ala in P1′ to Phe (compound **66**) attenuated the cathepsin K-catalyzed conversion without major improvement in the selectivity profile. Unexpectedly, the combination of this residue with Cit in P1 as realized in octapeptide **67** abolished the conversion by cathepsin B, in contrast to the observation made with substrate **56**. Such nonadditive behavior was also observed for other cathepsin substrates and indicates that the contribution of distinct residues to the enzyme–substrate interaction is influenced by the sequential context within the peptidic substrate.^98^

The efficient cleavage of the majority of compounds **44** and **56**-**67** (Table 1) by cathepsin K can be partly explained by the fact that both Leu and Gly are the most preferred P2 and P1 residues for this protease, respectively.^47,99,100^ Leu in P2 is furthermore the distinctively preferred P2 residue for the cathepsins S and L, which explains the lack of selective cathepsin B-catalyzed conversion. The missing selectivity of the octapeptide substrates toward the target enzyme cathepsin B does not need to be considered as impediment toward the intended application in activatable cell-penetrating peptides, because their secretion by tumor cells is less predominant compared to cathepsin B. The fact that also cathepsin L can be secreted by certain tumor cell lines^101,102^ and cathepsin S is secreted by tumor-associated immune cells^103^ should even enhance tumor uptake of activatable cell-penetrating peptides as cleavage can be conferred by multiple cysteine cathepsins. This conclusion is further supported when considering the partially compensatory functions of the cathepsins B, L, and S in the context of tumor progression.^104^

### Incorporation of the Optimized Stabilized Substrate into the Activatable Cell-Penetrating Peptide

Following the generic design introduced by Tsien et al., the octapeptide sequence GFLGAK­(Dnp)­(*N*Me)­GV, which was identified as a favorable substrate of cathepsin B with concomitant proteolytic stability in human serum, was integrated into ACPPs by placing it in between the N-terminal (d-Glu)_9_ attenuation sequence and the C-terminal (d-Arg)_9_ cell penetration sequence. In difference to the ACPP composition chosen by Tsien et al. and van Duijnhoven et al., who incorporated only one 6-aminohexanoic spacer N-terminal to the substrate linker, an additional 6-aminohexanoic spacer was introduced between the substrate sequence and the C-terminal penetration sequence to ensure sufficient flexibility. Following the design of previously reported ACPPs, a further 6-aminohexanoic spacer was placed after the (d-Arg)_9_ section followed by an amidated lysine residue as the ultimate C-terminal residue. The ε-amino group of this residue served as the attachment point of either the TAMRA fluorophore (compound **68**) or the NODAGA chelating moiety to enable radiolabeling with copper-64 (compound **71**, Figure 6). The NOTA macrocyclic chelator contained in NODAGA is aptly suited for the complexation of radioisotopes of both copper and gallium.^105^

**Figure 6:**
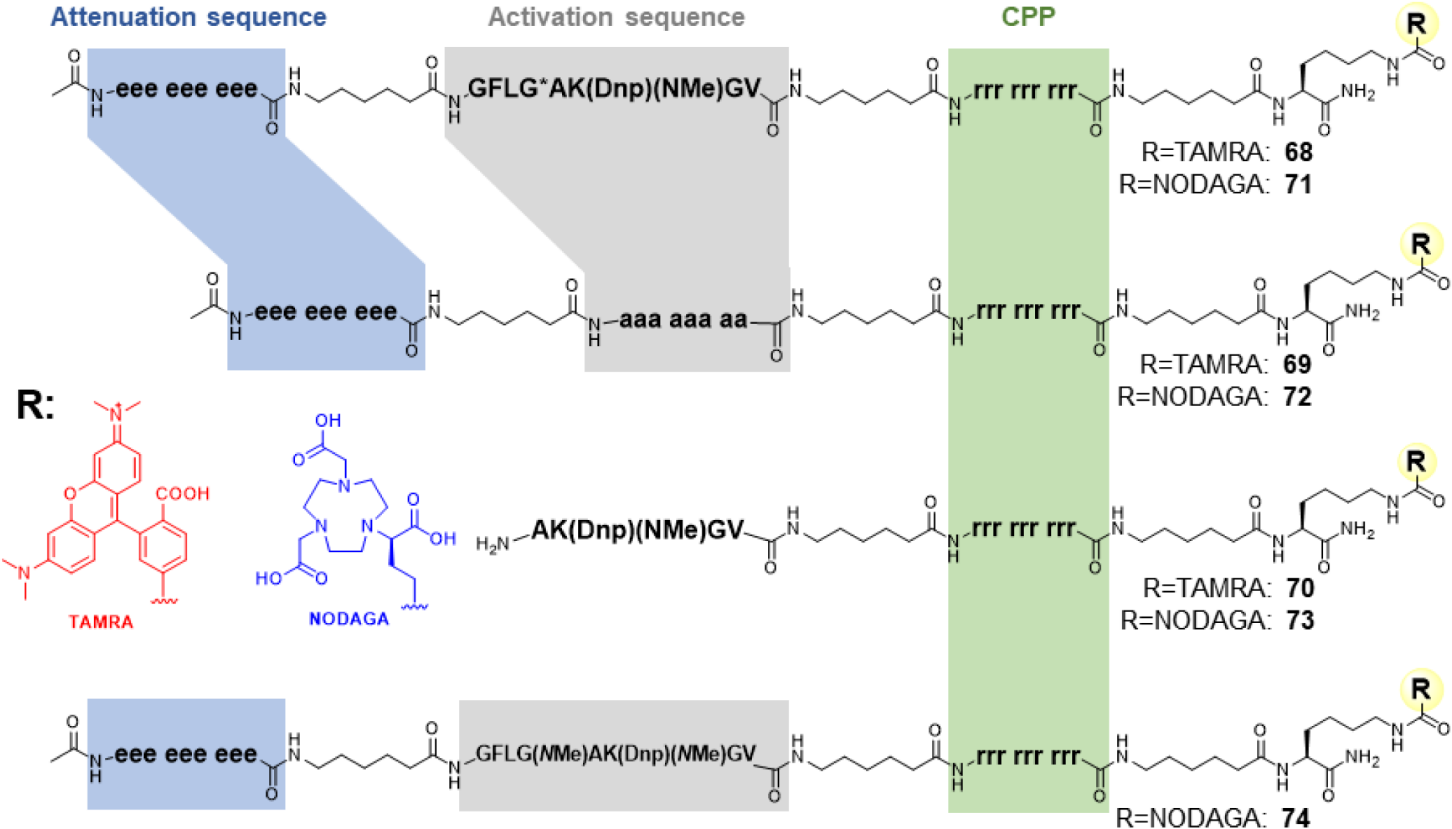
Schematic structure of 6-TAMRA- and NODAGA-conjugated peptidic probes and control compounds. TAMRA-ACPP (**68**) and NODAGA-ACPP (**71**): In the ACPP, the cathepsin B substrate **42** is inserted as an activation sequence between a nona-d-Glu attenuation sequence and a nona-d-Arg CPP sequence. The 6-TAMRA label is located C-terminally of the CPP as a side-chain modification of the C-terminal l-Lys residue. The peptide is C-terminally amidated to suppress potential exopeptidase activity. Individual sequence segments are linked by 6-aminohexanoic acid spacers to confer conformational flexibility to the construct. TAMRA-nACPP­(ala_8_) (**69**) and NODAGA-nACPP­(ala_8_) (**72**): In the nonactivatable cell-penetrating peptide, the activation sequence is substituted completely with d-Ala residues to prevent proteolytic cleavage and thereby cellular uptake. TAMRA-CPP (**70**) and NODAGA-CPP (**73**): In the preactivated peptide, the substrate activation sequence is truncated to the primed residues and conjugated to the CPP, thereby enabling cellular uptake without proteolytic activation. NODAGA-nACPP­(^NMe^Ala) (**74**): An alternative nonactivatable cell-penetrating peptide was designed by *N*-methylation of the Ala residue in the cleavage site.

The C-terminal cell-penetrating moiety containing the four residues of the C-terminal half of the substrate linker followed by the aminohexanoic spacer was synthesized as a constitutively membrane-permeating control vector in both fluorophore- and chelator-conjugated versions (compounds **70** and **73**, Figure 6).

In order to differentiate between the influence of proteolytic activation and the intrinsic pharmacokinetic properties of the probe molecules, further control vectors with impaired susceptibility to proteolysis were designed. To abolish proteolytic activation completely, the substrate sequence was replaced by octa-d-alanine, both for fluorophore- and chelator-conjugated versions (compounds **69** and **72**, respectively), which should render the linker moiety completely stable against degradation by all mammalian proteases.^106^ In the further course of the work, the requirement for suppressing cathepsin B-catalyzed cleavage by means of a structurally less divergent linker arose (see section Radiopharmacological Studies *In Vivo*). To this end, the P1–P1′ peptide bond between Gly and Ala in the substrate sequence was *N*-methylated, which led to NODAGA-conjugated compound **74** (Figure 6).

As for the isolated substrate peptides, the synthesis of all probe compounds was accomplished by sequential microwave-assisted SPPS. In difference to the octapeptide syntheses, each coupling step was repeated under omission of microwave irradiation and with an extended reaction time for each second step in order to ensure efficient peptide bond formation for the multiple d-Arg residues. Double coupling was reported as beneficial for the incorporation of Arg residues using coupling of Fmoc-Arg­(Pbf)–OH for which δ-lactamization competes with intramolecular peptide bond formation.^107–109^ Furthermore, the reaction time for acidic cleavage and side-chain deprotection was extended from 1 to 2 h to ensure complete removal of all Pbf groups from the side chains of the (d-Arg)_9_ sequence. To achieve the conjugation with the fluorophore or the metal chelator, the C-terminal Lys residue was attached to the Rink amide resin by coupling Aloc-Lys­(Fmoc)–OH, followed by Fmoc cleavage at the *N* ^ε^-amino group and coupling of 6-TAMRA or NODA-GA­(^*t*^Bu)_3_. Subsequently, Aloc was removed from the α-amino group and SPPS was continued (see Experimental Section). This protecting group strategy was pursued instead of using Fmoc-Lys­(Aloc)–OH because removal of the Aloc group at the ε-amino group is not possible without compromising the Fmoc-protected α-amino groups.^110^ Alternatively, selective functionalization of the side chain at the C-terminal Lys residue can be accomplished by assembling the Fmoc-Ahx-Lys­(Boc) dipeptide at the Rink resin and subsequent Boc removal by treatment with 10% H_2_SO_4_/dioxane for 30 min at room temperature, which did not result in significant cleavage from the resin.^111^ This strategy was followed in the synthesis of the peptidic probe compound **74**.

### Cellular Uptake Studies of TAMRA-Conjugated ACPP

#### Selection of Cell Lines: U87MG Cells Express and Secrete Cathepsin B

To identify suitable cathepsin B-expressing tumor cell lines, the glioblastoma cell lines U87MG and U251MG^112^ as well as the colorectal neoplastic cell lines SW403, SW480,^113^ and SW620 ^113^ were selected for more detailed expression analysis by Western blot based on an initial screening of cell lines selected in orientation to literature reports. The latter three cell lines are each derived from human colorectal adenocarcinoma. While SW403 and SW480 represent tumor cell lines of primary origin, the SW620 cell line is the lymph node metastatic variant of SW480.^114,115^ With regard to a negative control with negligible cathepsin B expression, the epidermoid carcinoma cell line A431 and the melanoma cell line Mel Juso, each derived from human primary tumors, were examined. Compared to isolated cells, expression levels of proteins can potentially vary in tumor tissue. Therefore, murine subcutaneous xenograft models of tumors were established on the basis of the mentioned cancer cell lines and the derived tissue lysates were subjected to Western blot analysis. Besides detection of cathepsin B, the expression profiles of the other oncologically most relevant cysteine cathepsins S, L, and K were also determined (see Figures S13–S15 in Supporting Information). Furthermore, as the activity of cysteine cathepsins *in cellulae* and *in vivo* is regulated by endogenous macromolecular inhibitors such as cystatin B and C, which is particularly well studied for human glioblastoma,^116^ Western blot-based analysis was extended to the detection of the latter two proteins, as shown in Figures S16 and S17 in Supporting Informatiion.

The Western blot analysis for the detection of cathepsin B in the different cell lines is shown in Figure S12 in Supporting Information. Depending on the posttranslational processing of cathepsin B, the mature enzyme can be present as a single-chain (27.6 kDa) or double-chain form (22.2 kDa for the heavy chain and 5.2 kDa for the light chain),^42,117^ which does not influence its catalytic activity.^118^ The U87MG cells show a prominent band at around 31 kDa, which can be assigned to the single-chain form of cathepsin B. In contrast, in U251MG, the double-chain form predominates and its large chain is present in the Western blot at around 21 kDa. The short chain is faintly recognizable at about 10 kDa. The double-chain form can also be detected primarily for SW480. These three cell lines are obviously promising as cathepsin B-positive cell lines. However, further investigations have shown that the U251MG cells show low cathepsin B activity on living cells in contrast to the high activity of the cell lysate. The inverse relation was found for U87MG cells, which was attributed to the expression of the intracellular proteinaceous cysteine cathepsin inhibitor cystatin B in these cells (see Figure S16 in Supporting Information).^59^ The SW480 cells significantly express the related endogenous inhibitor cystatin C (see Figure S17 in Supporting Information), which is largely secreted^119–121^ and will therefore attenuate extracellular cathepsin B activity. Consequently, U87MG was selected as the cathepsin B-positive tumor cell line. The SW403 cells show only a weak band, while no cathepsin B is detectable for SW620. In contrast, the A431 cells considered as a cathepsin B-negative cell line show a weak band for the double-chain heavy fragment, which is in accordance with recently published results.^122^ However, the SW620 cell line was excluded as a negative control cell line, as these cells were reported to secrete cathepsin B with a low intracellular concentration of the enzyme.^113^ Because the Mel Juso cells show distinctly no band for cathepsin B, this cell line was selected as the negative control cell line. Using kit-based subcellular fractionation, the localization of cathepsins B, S, L, and K in the lysosomal and membrane fraction as well as in the supernatant of the cultured U87MG cells and the other cell lines was investigated by Western blot analysis (Figure S12B in Supporting Information). Consistent with the cell-associated cathepsin B activity observed by Hulkower et al. and us,^59,123^ cathepsin B is detectable both in the membrane fraction and in the supernatant of U87MG cells, which is not the case for the other considered cell lines (Figure S12B in Supporting Information). Surprisingly, the Mel Juso cells, deemed to be cathepsin B-negative, seem to secrete the enzyme. However, considering the concomitant expression of cystatin C in these cells, they can still be considered negative with regard to the enzymatic activity of this cathepsin. In addition to cathepsin B, various species of cathepsin L are detectable in the membrane fraction (procathepsin L, single-chain mature, and heavy chain for double-chain mature cathepsin L^124^) while obviously only procathepsin L is secreted into the supernatant of the U87MG cells. Membrane retention of cathepsin L has been occasionally reported but the structural mechanism behind this subcellular localization remains elusive.^125,126^ Cathepsin S is not detectable in U87MG cells, and only faint bands of cell-associated cathepsin K are detectable for this line while its secretion into the supernatant does not seem to occur (Figure S12B in Supporting Information). Even though the expression levels vary between cultured cells and tumor tissue, the expression of the target enzyme in U87MG-derived xenograft tissue was confirmed as shown in Figure S12A in Supporting Information.

Immunohistochemical staining of cathepsin B, L, and S largely corroborated the Western blot results obtained with tissue homogenates (see Figures S18–S20 in Supporting Information). Cathepsin B is expressed at the edges (invasive fronts) of tumor tissue in all five tumor xenograft models, while cathepsin L is more uniformly distributed and cathepsin S seems to be largely absent in all tumors. The observed histochemical distribution in the tumor tissue, particularly the concentration of cathepsin B at the invasive front, is in agreement with literature reports.^116,127^

#### Uptake Studies by Fluorescence Microscopy: Cathepsin B-Mediated ACPP Activation by U87MG Cells

The time-dependent uptake of 6-TAMRA-ACPP (**68**) at a concentration of 5 μM was investigated by fluorescence microscopy for the U87MG glioblastoma cell line with confirmed cathepsin B expression and activity^59^ in comparison to both control compounds **69** and **70** (Figure 6) and Mel Juso as a control cell line of missing cathepsin B expression. The TAMRA-derived fluorescence emission was put into ratio to the signal of the nucleus-targeted fluorescent dye Hoechst 33342 to correct for fluctuations in cell number. The time course of uptake was followed for 30 min each, and the final ratio obtained for ACPP **68** in U87MG cells at 37 °C in the presence of DTT was set to 100%. The results are shown in Figure 7. Notably, the TAMRA-based emission signal concentrates in the subnuclear compartment that can be assigned to the nucleolus (Figure 7A). Enrichment in this suborganelle, which is electrostatically driven by the interaction with polyanionic rRNA, is a characteristic of oligoarginine-based CPPs, and this enrichment is even enhanced in the case of d-Arg derivatives.^128,129^ An increase of cellular uptake over the time was observed for the U87MG cells at 37 °C in the presence of DTT (0.5 mM), which was significantly diminished in the absence of DTT resulting in final uptake values that were approximately half as high compared to the presence of the reductant. At 4 °C, the uptake was even more strongly attenuated to approximately 10% in relation to 37 °C in the presence of DTT. These results suggest that the cellular uptake of **68** is an enzyme-initiated process. In particular, the beneficial influence of DTT on its uptake suggests the involvement of cysteine proteases because this reagent protects the active-site thiol group from oxidation and thus maintains enzymatic activity.^63,130,131^ In contrast, the uptake of the TAMRA-CPP control probe **70** exceeds that of ACPP **68** at every time point and reaches final values that are almost 5-fold as high as for **68**. Similar differences between the uptakes of ACPP and CPP were observed for an r_8_-based PSA-activatable cell-penetrating peptide with a disulfide-linked (DGG)_3_D as an attenuating domain in PC3M prostate cancer cells.^132^ Furthermore, the uptake of **70** at the same concentration proceeds significantly faster and is independent of temperature and the presence of DTT (Figure 7B). Noteworthy, the virtually identical uptake kinetics of **70** at 37 and 4 °C indicates that its cell entry proceeds through direct translocation through the membrane, which is in agreement to results from previous studies on the uptake mechanisms of oligoarginine-based cell-penetrating peptides.^18,133–136^ Cellular binding of the nonactivatable probe nACPP containing the (d-Ala)_8_ linker (compound **69**) was substantially lower than that of ACPP **68**.

**Figure 7:**
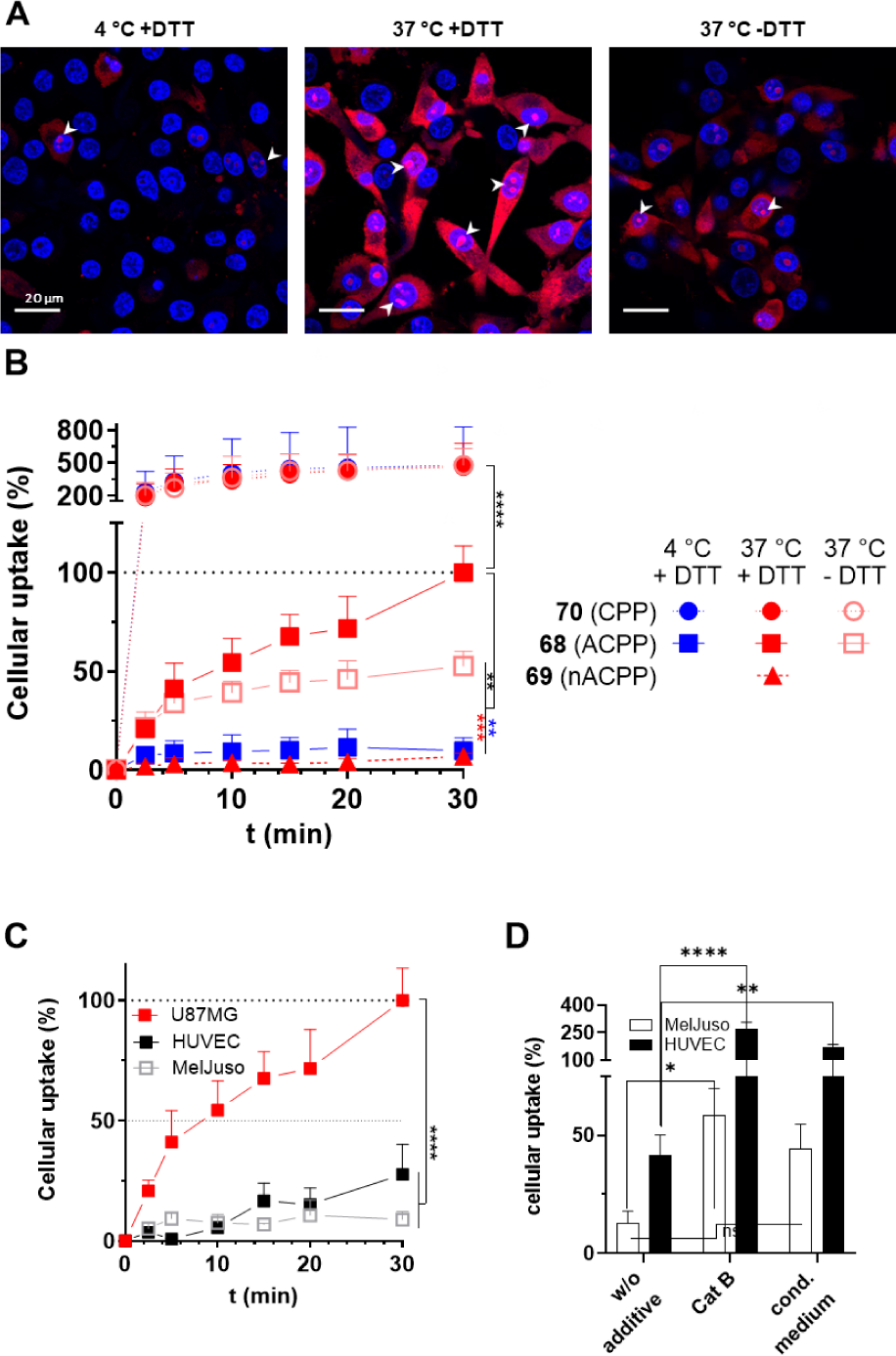
Time course of the cellular uptake of TAMRA-ACPP (**68**). (**A**) Fluorescence microscopic imaging of the cellular uptake of compound **68** in U87MG cells. Nucleolar accumulation of the released CPP fragment is highlighted by white arrowheads. Agent for nuclear staining: Hoechst 33342. (**B**) Dependence of cellular uptake on assay conditions. Cellular uptake of **68**, **69**, and **70** in U87MG cells was studied at different temperatures (4 °C vs 37 °C) and in the absence and presence of DTT (0.5 mM). *n* = 4–10. (**C**) Dependence of cellular uptake on cell line. Cellular uptake of TAMRA-ACPP (**68**) was studied in cells with high (U87MG) and low (HUVEC, Mel Juso) cathepsin B expression levels. *n* = 4–12. (**D**) Activity transfer assay. Induction of uptake of **68** by addition of 0.25 μg/mL cathepsin B to the incubation medium or addition of medium that was conditioned for 30 min on U87MG cells, *n* = 4–12. Uptake values are each normalized to the value of uptake of TAMRA-ACPP (**68**) in U87MG cells after 30 min at 37 °C in the presence of 0.5 mM DTT (100%) and plotted with SEM. Statistical analysis: Ordinary one-way ANOVA with Dunnett’s posttest; (**B**) vs **68**, 30 min, +DTT, 37 °C; (**C**) vs U87MG; (**D**) each vs w/o additive; ****p < 0.0001, ***p < 0.001, **p < 0.01, *p < 0.1, ns: not significant.

The uptake of ACPP **68** in the Mel Juso melanoma cells at 37 °C, which was selected as the control cell line of negligible cathepsin B expression, resembles that in U87MG cells at 4 °C, which suggests that proteolytic activation largely does not occur in this cell line. A further control experiment for the uptake of **68** was performed with the benign endothelial cell line HUVEC, which does not secrete cathepsin B, but potentially other proteases such as MMPs.^137^ Uptake of **68** in the HUVEC cells was somewhat higher compared to the Mel Juso cells but reached final values that were only 28% compared to the U87MG cells at 37 °C in the presence of DTT (Figure 7D). Cell association of TAMRA-ACPP (**68**) was strongly enhanced in the presence of exogenously added cathepsin B (0.25 μg/mL) for Mel Juso and HUVEC, which led to a 5-fold increase in the uptake for both cell lines (Figure 7D). This result provided evidence toward the cathepsin B-mediated uptake of **68**. A similar effect was observed when U87MG cell-conditioned medium was transferred to the Mel Juso and HUVEC cells, which increased the uptake to a similar extent as did the addition of the isolated enzyme (Figure 7D).

Further evidence toward cathepsin B-mediated cellular uptake was obtained by studying the cell association of TAMRA-ACPP (**68**) under the administration of various protease inhibitors at concentrations of 10 and 100 μM to the U87MG cells. Information for all applied inhibitor compounds is compiled in Table S4 in Supporting Information. As it is obvious from the microscopic images, the presence of 10 μM CA074, E64, and leupeptin strongly attenuated the cell-associated red fluorescence (Figure S21A in Supporting Information). In contrast, the other protease inhibitors pepstatin, ilomastat, and the neutral endopeptidase inhibitor LBQ657 at the same concentration were obviously not effective in this regard. The epoxysuccinyl peptides E64 and CA074 are broad-band cysteine cathepsin and cathepsin B-selective irreversible inhibitors, respectively.^138^ However, CA074 was reported to inhibit cathepsin S at higher concentrations^139^ and to inhibit cathepsin L under reducing conditions.^140^ The latter result was apparently not confirmed by Yoon et al., but these contradictory results are not discussed by the authors.^139^ Pepstatin A is a broad-band inhibitor of aspartic proteases including the oncologically relevant cathepsin D^141^ and the peptide-derived hydroxamic acid ilomastat is a potent inhibitor of metalloproteases including the MMPs.^142^ Influence of CA074, E64, and leupeptin was also investigated by applying concentrations of 100 μM. The inhibitory effect of both concentrations was quantified as shown Figure S21B in Supporting Information. Considering that at a concentration of 10 μM only CA074, E64, and leupeptin exerted a statistically significant effect on the uptake of **70**, the involvement of secreted cysteine cathepsins is highly evident. Furthermore, the recently confirmed selectivity of inhibitor CA074 for cathepsin B over cathepsin L^139^ and the fact that among the four most relevant cysteine cathepsins, only cathepsin B and procathepsin L were detectable in the cell supernatant by Western blot, as described above, suggest that the former enzyme is the major protease mediating the uptake of **68** into U87MG cells. This conclusion is in line with the observation that cathepsin B and U87MG-conditioned medium are capable of inducing the uptake in Mel Juso and HUVEC cells to a similar extent. However, the contribution of other proteases to a minor extent is likely, because neither CA074 nor E64 nor leupeptin at 100 μM can block the probe uptake completely. Distinct to the uptake of **68**, the uptake of TAMRA-CPP (**70**) was not inhibited by the presence of the cysteine cathepsin inhibitors CA074, E64, and leupeptin (Figure S21C in Supporting Information), which provides compelling evidence that the uptake of **68** is initiated by proteolytic cleavage.

To obtain insight into the mechanism of cell permeation, the uptake of **68** was investigated in the presence of various modulators that influence the subcellular dynamics of transport processes. In particular, colchicine, cytochalasin D, and vacuolin-1 were investigated as potential uptake inhibitors at concentrations of 100 μM each (Figure S21D in Supporting Information). The involvement of the cytoskeleton, which mainly consists of supramolecular filaments formed by actin and tubulin monomers, in endocytotic processes is known for a long time.^143^ While colchicine acts as an inhibitor of microtubule assembly by suppressing the polymerization of tubulin subunits, cytochalasin D blocks the assembly of monomeric actin into filaments^144,145^ and, therefore, inhibits multiple endocytotic pathways, predominantly macropinocytosis.^146^ The presence of cytochalasin D did not show a significant effect on the uptake of **68**, which is in accordance to reports that the uptake of oligoarginines proceeds mainly independently of endocytosis.^133^ However, colchicine at an identical concentration led to a reduction in uptake to 57%. The different effect of colchicine compared to that of cytochalasin D can be rationalized in the context of the results of Allolio et al., who elaborated that nona-l-arginine (R_9_) enters HeLa cells via the induction of multilamellarity of the membrane lipid bilayer followed by membrane fusion and subsequent pore formation.^134^ Independently, it has been recently shown that similar processes of membrane remodeling are supported by microtubules without the involvement of motor proteins.^147^ The inhibitory effect of vacuolin-1, which can inhibit endosomal trafficking and the fusion of cytotic vesicles and lysosomes,^148^ mainly via inhibition of phosphatidylinositol-3-phosphate-5 kinase (PIKfyve),^149^ was statistically nonsignificant. This result suggests that the transport of **68** and its cleavage products largely does not occur within endosomal-lysosomal compartments, which is in line with its nonendocytotic uptake concluded from the missing inhibitory effect of cytochalasin D.

### Radiopharmacological Studies *In Vivo*

As the cathepsin B-mediated uptake in U87MG cells was confirmed, a murine xenograft model was established on the basis of this cell line and the distribution of copper-64-labeled NODAGA-ACPP ([^64^Cu]­Cu-NODAGA-ACPP; [^64^Cu]­Cu-**71**) in comparison to the corresponding CPP ([^64^Cu]­Cu-NODAGA-CPP; [^64^Cu]­Cu-**73**) and nACPPs ([^64^Cu]­Cu-NODAGA-nACPP­(ala_8_); [^64^Cu]­Cu-**72** and [^64^Cu]­Cu-NODAGA-nACPP­(^NMe^Ala); [^64^Cu]­Cu-**74**, see Figure 6 for structures) was studied by small animal PET imaging in the tumor-bearing mice complemented by *ex vivo* biodistribution studies. Furthermore, radiometric *ex vivo* blood analysis was performed in healthy Wistar rats for [^64^Cu]­Cu-**71** and [^64^Cu]­Cu-**73** to obtain solid pharmacokinetic parameters and insight into the metabolic stability of the polyionic peptides, which will be discussed first within the next section.

#### Stability Studies and Investigation of Basic Pharmacokinetics in Wistar Rats: ACPPs Show More Favorable Behavior Than CPPs

Blood sampling and analysis revealed that, within the circulation of rats, in addition to degradation products resulting from proteolytic processing, a significant portion of intact [^64^Cu]­Cu-**71** is detectable in the blood plasma at 60 min p.i. Worth of note, original [^64^Cu]­Cu-**71** is also present in the urine at the same time point. In contrast, *ex vivo* analysis after administration of the authentic cell-penetrating cleavage product [^64^Cu]­Cu-**73** revealed that it is largely degraded and rapidly eliminated from the blood circulation as virtually no original tracer compound is detectable in the blood plasma at 60 min p.i. (Figure S22 in Supporting Information). Based on these *ex vivo* analysis data, pharmacokinetic parameters were calculated for [^64^Cu]­Cu-**71** and [^64^Cu]­Cu-**73** (Table S5 and Figure S23 in Supporting Information). In accordance to the observed rapid excretion of [^64^Cu]­Cu-**73**, the elimination half-life of [^64^Cu]­Cu-**71** is with 45 min as double as high as that of [^64^Cu]­Cu-**73** (25 min). Despite the higher blood retention time of [^64^Cu]­Cu-**71**, its specific volume of distribution is with 2561 mL/kg more favorable than that of its C-terminal cleavage product [^64^Cu]­Cu-**73** (8501 mL/kg), which should potentially account for higher contrast of the PET images. Accordingly, the clearance of [^64^Cu]­Cu-**73** is significantly higher compared to the probe compound [^64^Cu]­Cu-**71** (236 mL min kg^–1^ vs 40 mL min kg^–1^). Worth of note, elimination of [^64^Cu]­Cu-**73** occurs predominantly via the liver whereas that of [^64^Cu]­Cu-**71** seems to follow mainly the renal path, as concluded from the activity fractions (%ID) in the rat for the liver and the kidneys at 60 min p.i. (see Table S5 in Supporting Information). The observed rapid clearance and high liver uptake of [^64^Cu]­Cu-**73** from the blood circulation are in agreement with the results obtained by Sarko et al., who studied the biodistribution of ^111^In-labeled CPPs of multiple classes in tumor-bearing mice.^150,151^ For the majority of those peptides, the blood activity was <1% ID/g at 60 min p.i. and, in particular, the uptake of the [^64^Cu]­Cu-**73**-related R_9_ peptide was highest in the liver with 49.9% ID/g followed by uptake in the spleen and kidneys with 8.2% ID/g and 8.1% ID/g, respectively.^150^ Moreover, the more favorable pharmacokinetics of [^64^Cu]­Cu-**71** over [^64^Cu]­Cu-**73** in terms of less rapid clearance and attenuated liver uptake corroborates the findings of Aguilera et al., who studied the systemic distribution of an MMP-2/-9-activatable ACPP and its corresponding cell-penetrating C-terminal fragment each conjugated to the Cy5 fluorophore in HT-1080 tumor-bearing mice^152^ The high volume of distribution observed for [^64^Cu]­Cu-**73** does not necessarily reflect its capability to permeate into cells, because a similar behavior was observed for cationic ^3^H-labeled poly-l-lysine-based dendrimers, which was attributed to vascular surface binding rather than extravasation.^153^ Reversible adhesion of [^64^Cu]­Cu-**73** at vascular surfaces could be rationalized by considering that endothelial cells are coated by a glycocalyx rich in negatively charged hyaluronic acid.^154^ In line with this interpretation is the fact that the majority of the ^64^Cu activity in the blood is distributed in the plasma while <10% are associated with the erythrocytes (see Figure S23 in Supporting Information). This applies to both [^64^Cu]­Cu-**73** and [^64^Cu]­Cu-**71**. In the case of membrane permeation, this value should be higher. Notably, plasma protein binding is low for both [^64^Cu]­Cu-**73** and [^64^Cu]­Cu-**71**, as upon precipitation with trichloroacetic acid, the majority of the ^64^Cu activity was distributed in the plasma supernatant while <10% of it was found in the protein pellet.

#### Radiopharmacological Studies in U87MG Tumor-Bearing Mice: Proteolytic Activation Is Evidenced but Does Not Lead to Enhanced Tumor Retention

*Ex vivo* biodistribution patterns were determined in the U87MG xenograft mice to gain insights into the basic pharmacokinetic properties and to compare the tumor uptake of the radiolabeled probe and its derivatives for time points 1, 4, and 24 h p.i. The obtained results, in particular the inverse relations of uptake values in the liver and kidney, largely confirm the differential pharmacokinetic characteristics of [^64^Cu]­Cu-**73** and [^64^Cu]­Cu-**71** as observed in Wistar rats. Further significant uptake was noted in the lung and the spleen. Although the uptake of [^64^Cu]­Cu-**71** is attenuated compared to the active cell-penetrating peptide [^64^Cu]­Cu-**73** (Figure 8A), the difference between the probe and its C-terminal fragment was less evident in these organs. Of note, [^64^Cu]­Cu-**73** was retained to some extent in bone tissue, as reflected by increasing SUV values in the femur (Figure 8A). For comparison, the initial uptake of [^64^Cu]­Cu-**71** at 1 h p.i. was similar in this region, but values then decreased. Bone retention of [^64^Cu]­Cu-**73** can be explained by the high content of polyanionic proteoglycans in this tissue.^155^ Biodistribution results covering the comprehensive set of different organs and tissues for each compound are shown in Supporting Information (Tables S6–S8). The uptake in the tumor tissue is low for both [^64^Cu]­Cu-**73** and [^64^Cu]­Cu-NODAGA-**71** (SUV < 0.5) but higher compared to the muscle as the control organ. Regarding the tumor accumulation of [^64^Cu]­Cu-**71**, no significantly higher uptake compared to [^64^Cu]­Cu-**73** was discernible for the different time points, even though the SUV values were somewhat higher for [^64^Cu]­Cu-**71** than for [^64^Cu]­Cu-**73** for all time points (Figure 8A).

**Figure 8:**
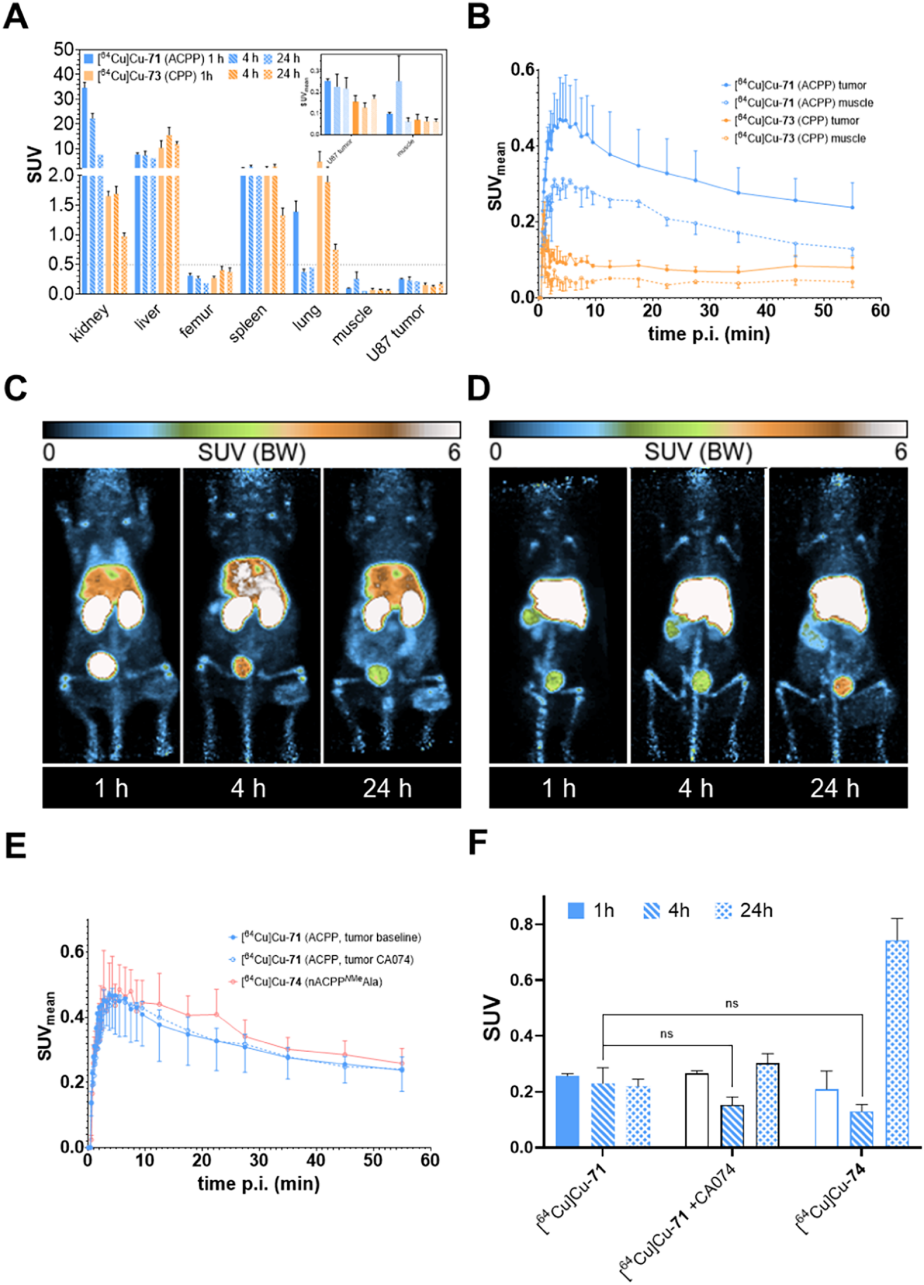
Radiopharmacological characterization of [^64^Cu]­Cu-NODAGA-ACPP ([^64^Cu]­Cu-**71**) and [^64^Cu]­Cu-NODAGA-CPP ([^64^Cu]­Cu-**73**) in U87MG-bearing mice. Data were obtained from 4 independent experiments (*n* = 4) and are plotted with SEM (bar diagrams) or SD (time-activity curves). (**A**) *Ex vivo* biodistribution of [^64^Cu]­Cu-**71** and [^64^Cu]­Cu-**73** in U87MG-bearing mice for selected organs and tumor tissue. The inset shows the uptake in the muscle and tumor tissue on an adapted y scale. Uptake values (SUV) for all organs and both compounds are included in Tables S6 and S7 in Supporting Information. (**B**) PET-derived time-activity curves [^64^Cu]­Cu-**71** and [^64^Cu]­Cu-**73** in the tumor and muscle region. (**C**) and (**D**) PET images for [^64^Cu]­Cu-**71** (**C**) and [^64^Cu]­Cu-**73** (**D**). **E** and **F**: Investigations toward the cathepsin B-mediated tumor uptake of [^64^Cu]­Cu-NODAGA-ACPP ([^64^Cu]­Cu-**71**) by administration of CA074 and the nonfunctional probe [^64^Cu]­Cu-NODAGA-nACPP­(^NMe^Ala) ([^64^Cu]­Cu-**74**). (**E**) PET-derived time-activity curves. (**F**) *Ex vivo* biodistribution. Statistical analysis: Ordinary one-way ANOVA with Dunnett’, ns: not significant.

More detailed information on the different tumor uptake behaviors of the two radiotracers was obtained from time-resolved small animal PET imaging. The time-activity curves retrieved from the PET data for the tumor region are shown in Figure 8B. The uptake of the cell-penetrating peptide [^64^Cu]­Cu-**73** is low (SUV_mean_ = 0.22), with a narrow maximum reached as early as 1 min p.i. followed by a rapid washout. In contrast, the uptake of [^64^Cu]­Cu-**71** in the tumor tissue reached levels more than double as high (SUV_mean_ = 0.47) and its elimination is protracted, which results in strongly enhanced integral tumor accumulation compared to the cell-penetrating C-terminal fragment [^64^Cu]­Cu-**73** (AUC_0–60 min_ = 4.48 SUV·min vs 17.42 SUV·min, respectively). Accordingly, the tumor tissue delineates with enhanced contrast in the static PET images for [^64^Cu]­Cu-**71** compared to [^64^Cu]­Cu-**73** (Figure 8C and D, respectively). The ratio of 2.14 between the SUV_mean_ of [^64^Cu]­Cu-**71** and [^64^Cu]­Cu-**73** is similar to the value observed for a fluorescent MMP-2/9-cleavable ACPP relative to its cell-penetrating fragment determined *ex vivo*.^20^ These results contrast to those obtained by van Duijnhoven et al., who found that for MMP-2/9-targeted ACPPs, uptake in the tumor region is higher for the cell-penetrating C-terminal fragment than for the activatable probes.^22^ As reported in the experimental section there and not discussed by the authors, the DOTA-conjugated ^177^Lu-labeled ACPP probe was administered at a dose of 60 nmol, which is about 500 times higher than in the present study (see Table S10 in Supporting Information). The discussion of this aspect will be taken up below.

In order to prove the cathepsin B-mediated character of the enhanced tumor uptake of [^64^Cu]­Cu-**71** compared to [^64^Cu]­Cu-**73**, two different approaches were pursued. On the one hand, the cathepsin B-specific inhibitor CA074 was applied at a dose of 10 mg/kg^157^ to attenuate the tumor-associated activity of this enzyme. Data for *ex vivo* biodistribution of [^64^Cu]­Cu-**71** in the presence of CA074 for all and selected organs are shown in Table S6 and Figure S24 in Supporting Information, respectively. Neither in the PET-derived nor in the *ex vivo* biodistribution data was a significant inhibition of the tumor uptake of [^64^Cu]­Cu-**71** discernible (Figure 8E and F), which suggests that its uptake in the tumor tissues is largely independent of the proteolytic activity of cathepsin B. On the other hand, analogues of [^64^Cu]­Cu-**71** bearing a modified octapeptide linker with varying susceptibility toward proteolytic cleavage were synthesized and investigated to further obtain insight into the influence of protease activity onto the tumor uptake. First, nACPP­(ala_8_), which in its TAMRA-conjugated version (**69**, Figure 6) was successfully employed as a control probe in the fluorescence-based cell uptake studies, was equipped with NODAGA. However, PET imaging experiments with the resulting [^64^Cu]­Cu-NODAGA-nACPP­(ala_8_) ([^64^Cu]­Cu-**72**) have shown significantly higher uptake in the lungs compared to [^64^Cu]­Cu-**71** and [^64^Cu]­Cu-**73**, which might indicate aggregation (see Figure S25 and Table S8 in Supporting Information). Furthermore, [^64^Cu]­Cu-**72** was obtained in insufficient radiochemical purity <80% (see Figures S33–S36 in Supporting Information). Therefore, this compound was deemed unsuitable for proving cathepsin B-mediated uptake *in vivo*. In an alternative approach to modify the proteolytic susceptibility of the octapeptide linker accordingly, its alanine residue in P1′ position was *N*-methylated, as this kind of modification of the peptidic backbone strongly attenuates protease-catalyzed cleavage in general.^156,158–160^ Cleavage of the resulting [^64^Cu]­Cu-NODAGA-nACPP­(^NMe^Ala) ([^64^Cu]­Cu-**74**, Figure 6) by isolated cathepsin B was investigated in comparison to that of the corresponding ACPP [^64^Cu]­Cu-**71** (see Figure S26 in Supporting Information). Even though *N*-methylation did not abolish the cleavage, the cathepsin B-catalyzed conversion of [^64^Cu]­Cu-**74** was determined to be 2.4-fold slower compared to [^64^Cu]­Cu-**71** (9.4 mM^–1^ s^–1^ vs 22.6 mM^–1^ s^–1^ for *k*_obs_/[*E*]). The more efficient cleavage of [^64^Cu]­Cu-**71** compared to the octapeptide **42** (*k*_cat_/*K*_m_ = 5.8 mM^–1^ s^–1^) might result from the higher temperature of 37 °C, which was 30 °C for the fluorimetric assay. Furthermore, the absence of the bulky Abz group in [^64^Cu]­Cu-**71** could potentially contribute to its faster cathepsin B-catalyzed conversion. Worth of note, the *k*_obs_/[*E*] value determined for [^64^Cu]­Cu-**71** is of a similar magnitude as that of other radiotracers equipped with a protease cleavage site whose enzymatic conversion influences their biodistribution.^161^ The lung uptake of [^64^Cu]­Cu-**74** was in the range as that observed for [^64^Cu]­Cu-**71** and [^64^Cu]­Cu-**73** (see Figure S28 and Table S9 in Supporting Information). In line with the missing effect of CA074, *N*-methylation did not significantly change the tumor uptake as the PET-derived time-activity curves for the tumor region are very similar for [^64^Cu]­Cu-**71** and [^64^Cu]­Cu-**74** (Figure 8E), despite the conversion of the latter by cathepsin B is significantly slower. This result indicates that cathepsin B-catalyzed cleavage does not obviously contribute to the tumor uptake of [^64^Cu]­Cu-**71** in the U87MG xenograft model.

To obtain information regarding tumor-associated proteolytic cleavage, the tumor and other tissues of the U87MG-xenografted mice were extracted at dedicated time points after injection of either [^64^Cu]­Cu-**71** or [^64^Cu]­Cu-**73** and tissue homogenates were analyzed by radio-HPLC (see Figure S29 in Supporting Information). While at 60 min p.i. mainly original [^64^Cu]­Cu-**71** was detectable in the blood, a mixture of [^64^Cu]­Cu-**71** and [^64^Cu]­Cu-**73** was present in the tumor tissue. At 18 h p.i., only [^64^Cu]­Cu-**73** was detectable besides degradation products of lower retention time in the tumor tissue. Worth of note, neither [^64^Cu]­Cu-**71** nor [^64^Cu]­Cu-**73** was detectable in the excretory organs at both time points, but [^64^Cu]­Cu-**73** appeared in the urine at 60 min p.i. These findings indicate that [^64^Cu]­Cu-**71** appears to be specifically converted to [^64^Cu]­Cu-**73** in the tumor tissue. However, as discussed above, this proteolytic event does obviously not contribute to tumor uptake as treatment with CA074 did not have an influence in this regard and there was no significant difference between the time course of [^64^Cu]­Cu-**71** and [^64^Cu]­Cu-**74** in the U87MG xenograft tissue.

The results obtained for radiolabeled [^64^Cu]­Cu-NODAGA-ACPP ([^64^Cu]­Cu-**71**) *in vivo* are somewhat contradictory to the observations made with TAMRA-ACPP (**68**) and the corresponding fluorescent control compounds **69** and **70** by microscopic studies at the cellular level, which unequivocally demonstrated that cathepsin B-catalyzed activation of the probe translates into cell permeation. To support the comparison of the results from fluorescence microscopy of cultured cells and PET imaging in animals, the uptake of [^64^Cu]­Cu-NODAGA-ACPP ([^64^Cu]­Cu-**71**), [^64^Cu]­Cu-NODAGA-CPP ([^64^Cu]­Cu-**73**), [^64^Cu]­Cu-NODAGA-nACPP­(ala_8_) ([^64^Cu]­Cu-**72**) and [^64^Cu]­Cu-NODAGA-nACPP­(^NMe^Ala) ([^64^Cu]­Cu-**74**) was investigated in U87MG and Mel Juso cells (see Figure S30 in Supporting Information). Cell binding of [^64^Cu]­Cu-**71** was considerably lower than that of [^64^Cu]­Cu-**73** and no significant difference between the cathepsin B-expressing U87MG cells and the cathepsin B-negative Mel Juso cells was evident with regard to the binding of the activatable radiolabeled probe. For unknown reasons, binding of [^64^Cu]­Cu-**73** to Mel Juso cells is higher than for U87MG cells, but the cellular uptake in general is low and quantitatively comparable to that of nona-l-arginine in different cell lines.^150^ Furthermore, the cell binding propensity of [^64^Cu]­Cu-**72** and [^64^Cu]­Cu-**74** was in a similar range as that of [^64^Cu]­Cu-**71** and comparable to that of other uncleaved ACPPs.^26^ Cleavage of the probe [^64^Cu]­Cu-**71** in the presence of U87 cells was not detectable (see Figure S31 in Supporting Information). These findings are in line with the absent cathepsin B-mediated tumor uptake *in vivo* but incongruous to the putative tumor-associated conversion of [^64^Cu]­Cu-**71** to [^64^Cu]­Cu-**73** as observed by radio-HPLC analysis *ex vivo*. This discrepancy could be explained by the fact that the area of exposure to cathepsin B is higher in the tumor tissue *in vivo* than in a layered *in vitro* culture and maybe the cathepsin B activity is higher *in vivo* due to contributions from nonmalignant cells in the tumor microenvironment. Similar observations were reported in the context of MMP-activatable ACPPs, for which cell uptake was significantly lower than for the corresponding CPP and in a comparable range to that of the corresponding non-ACPP. This relation was found for both MMP-2/9- and MMP-14-activatable CPPs each in the presence of HT-1080 fibrosarcoma cells.^22,23^ To partially mimic the microenvironment that the U87MG cells might encounter *in vivo*, the *in vitro*-cultivated tumor cells were exposed to assay buffer at pH 6.0 for 30 min, which stimulates the secretion of cathepsin B,^33^ prior to the addition of [^64^Cu]­Cu-**71**. Analysis of the supernatant after an incubation time of 15 min indicated the partial cleavage of [^64^Cu]­Cu-**71** (see Figure S32 in Supporting Information). This finding makes the tumor-associated activation of ACPP [^64^Cu]­Cu-**71** noted above even more plausible.

The missing evidence for cathepsin B-mediated uptake of [^64^Cu]­Cu-**71** is in disagreement to the results obtained for its fluorescent analogue TAMRA-ACCP (**68**) in the fluorescence microscopy studies. These contradictory observations can be reconciled by considering the amounts of probe molecules applied toward the viable cells. While **68** was applied at 5 μM, the concentration of [^64^Cu]­Cu-**71**—in accordance with a radiotracer approach—was more than 3 orders of magnitude lower. Consequently, the considerably lower rate of cathepsin B-catalyzed conversion might be insufficient to release enough [^64^Cu]­Cu-**73** for significant cell permeation. Besides the concentration-dependent rate of enzyme-catalyzed ACPP conversion, also the uptake of the released CPP depends critically on concentration. In particular, membrane passage of Arg-rich peptides by direct translocation is effective in the concentration range >1 μM,^162^ which can be explained in light of recent studies showing that positively charged CPPs can induce membrane hyperpolarization, which in turn leads to the formation of pores that allow cell entry.^163,164^ The different amounts of probe molecules might also be the key to explain the opposing results observed for ^177^Lu-labeled MMP-2/-9-activatable cell-penetrating peptides by Duijnhoven et al., for which tumor uptake was lower than that of the corresponding cell-penetrating C-terminal cleavage product when both probes were administered at approximately 500 times higher animal doses than those used here, as mentioned above (see Table S10 in Supporting Information).^22^ Moreover, ACPP animal doses of 10 nmol, which is 100 times higher compared to the dose applied herein, were chosen by Tsien’s group for Cy5-conjugated MMP-2/-9-cleavable ACPPs. The researchers estimated that the effective concentration reached in the target tissue, even if complete ACPP-CPP conversion is assumed, should not exceed the submicromolar range and therefore remain below the above-mentioned threshold for direct membrane translocation. On the other hand, the maximum dose of ACPPs is limited by the high toxicity of the released CPP.^152^ Therefore, the potential of arg*_x_-*/glu_*x*_-based ACPPs for protease-activated tumor targeting, in particular for radionuclide theranostic applications, should be reconsidered.

## Conclusions

Octapeptides which can be efficiently cleaved by cathepsin B were identified on the basis of stepwise C-terminal extension of the previously reported optimal exopeptidase substrate sequence GIVRAK. Kinetic characterization of various derivatives as cathepsin B substrates was based on internal dequenching of the Abz-Dnp FRET pair. The P4′ residue in these octapeptidic substrates was identified as an important determinant of specificity. Furthermore, kinetic hysteresis was found to be related to true endopeptidase activity associated with the open occluding loop conformation of cathepsin B. This conclusion is drawn on the basis of P4′-related structure–kinetics relationships in combination with molecular modeling studies. To achieve sufficient stability with respect to targeting of cathepsin B *in vivo*, the N-terminal moiety GIVR was replaced by GFLG and *N*-methylation of the P2′–P3′ peptide bond, which increased the half-life in human blood serum from 3.7 min to 23.4 h. The linker sequence, which was optimized in terms of both enzyme-catalyzed conversion and metabolic stability, was incorporated into the TAMRA-conjugated activatable cell-penetrating peptide. The cathepsin B-dependent cell uptake of the thereby obtained probe was detected by fluorescence microscopy in the glioblastoma cell line U87MG. In contrast, tumor uptake of the NODAGA-conjugated version labeled with copper-64 at n.c.a. level, as determined by PET imaging in a murine U87MG-derived tumor xenograft model, did not correlate with cathepsin B activity, although tumor-associated proteolytic cleavage was evidenced. This suggests to conclude that the capability of ACPPs for protease-dependent tumor delivery of radionuclides is limited.

The TAMRA-conjugated ACPP was identified as a well-suited tool for the investigation of extracellular cathepsin B activity, which can potentially be used for activity-based FACS experiments, for example. With regard to cathepsin B-mediated tumor delivery, the potential of the GFLGAK­(Dnp)­(NMe)­GV octapeptide as a cleavable linker is indicated and needs to be further explored, for example, in vector-drug conjugates. The delivery of n.c.a. radionuclides to tumor tissue via cleavage of this linker should be further explored by incorporating it into peptidic constructs that can trigger a conformational change leading to membrane insertion independent of cell penetration.^165^

## Experimental Section

### Automated Solid-Phase Peptide Synthesis

Automated peptide synthesis was performed using a BiotageTable Initiator+ Alstra automated microwave peptide synthesizer.

In the general setup, octapeptides were synthesized using Fmoc-Rink Amide-MBHA resin at a 0.1 μmol scale and 5 equiv of Fmoc-AA–OH at a concentration of 0.25 mM (in NMP) per coupling, using microwave-assisted couplings. Synthesis of ACPP, CPP, and nACPP peptides (6-TAMRA- or NODAGA-modified, compounds **69**-**74**) was performed using manually loaded and modified ChemMatrix resin, at a scale of 0.03 mmol using 5 equiv of Fmoc-AA at a concentration of 0.05 mM (in NMP) per coupling. Each amino acid was coupled in a triple coupling, with the first reaction performed at RT and the two subsequent reactions with microwave support.

Resin swelling was performed by agitating the resin in 4.5 mL of DMF for 20 min at 70 °C, after which the solution was aspirated. Automated removal of the Fmoc-protecting group was performed by adding 4.5 mL of 20% piperidine in NMP (v/v) to the resin, which was repeated once. The reaction mixture was agitated at RT for 3 min in the first deprotection step and for 10 min in the second. The deprotection solution was aspirated between reactions. The resin was washed four times with 4.5 mL of DMF after the second deprotection step.

For automated coupling reactions with microwave heating, the amino acid, HATU (0.5 M in NMP), and DIPEA (2 M in NMP, 2 equiv to AA) were added to the resin in this order. The resin was subsequently agitated under microwave heating (75 °C, max. 125 W) for 5 min, after which the reaction solution was aspirated and the resin was washed twice with 4.5 mL DMF. For the synthesis of ACPP, CPP, and nACPP, each coupling step was repeated at room temperature for 1 h with otherwise identical settings to microwave-assisted synthesis.

### Solid-Phase Synthesis with Manual Operations

#### General Procedure for N^α^-Acetylation

Acetylation was performed either to block remaining reaction sites on the resin after manual loading or as a final modification step for the ACPP and nACPP constructs (compounds **69**-**74**). After swelling the resin in 5 mL CH_2_Cl_2_, 10 equiv (corresponding to the amount of free amino groups) of acetic anhydride and 10 equiv of DIPEA were added. The reaction mixture was agitated for 15 min at RT. Subsequently, the resin was washed three times with CH_2_Cl_2_. This procedure was performed two times. After the final deprotection cycle, the resin was washed five times each with CH_2_Cl_2_, DMF, CH_3_OH, and Et_2_O and subsequently dried under reduced pressure.

#### General Procedure for Aloc-Deprotection

Aloc was used as N^α^-protecting group of Aloc-l-Lys­(Fmoc)–OH introduced as the C-terminal amino acid for reporter group conjugation in the optimized synthesis route of the ACPP, CPP, and nACPP constructs (compounds **68**-**73**). After swelling the resin in 15 mL CH_2_Cl_2_, 20 equiv of phenylsilane were added. The solution was degassed using argon. 0.2 equiv of Pd­(PPh_3_)_4_ were weighed in under an argon atmosphere and added to the solution. The reaction mixture was agitated for 20 min at RT. Subsequently, the resin was washed three times with CH_2_Cl_2_. This procedure was performed three times. After the final deprotection cycle, the resin was washed four times each with CH_2_Cl_2_ and DMF, six times with a freshly prepared solution of sodium diethyldithiocarbamate in DMF (15%, W/V), and further four times each with DMF, CH_2_Cl_2_, and Et_2_O and subsequently dried under reduced pressure. After Aloc deprotection, the resin batches were subdivided for automated SPPS (see above). Alternatively, the C-terminal Lys residue was loaded as Fmoc-Lys­(Boc)–OH (instead of Aloc-l-Lys­(Fmoc)–OH) to the Rink-amide ChemMatrix resin (200 mg, 0.5 mmol/g) for the synthesis of compound **74**. After coupling of Fmoc-Ahx–OH, the dipeptide-bearing resin was treated with dioxane (4 mL) followed by the addition of conc. H_2_SO_4_ (400 μL) and agitated for 30 min at room temperature in order to remove the Boc group. The thereby deprotected *N* ^ε^-amino group was acylated with NODA-GA­(^*t*^Bu)_3_ as described below.

#### General Procedure for Manual Coupling of Fmoc-AA–OH and Boc-2Abz–OH

For the coupling of Fmoc-AA, the resin was swollen in NMP. Five equiv of the protected AA and 5 equiv of HATU were dissolved in NMP and added to the resin. Ten equiv of DIPEA were added for *in situ* activation. The suspension was agitated at RT for 6 h. Subsequently, the resin was washed either five times each with DMF, CH_2_Cl_2_, and again DMF and the next coupling was performed immediately or, if reaction analytics were deemed necessary, the resin was washed five times each with DMF, CH_2_Cl_2_, CH_3_OH, and Et_2_O and dried. After the procedure, either a Kaiser’s test or a test cleavage was performed (see below).

#### General Procedure for Manual Coupling of Other Carboxylic Acids

Coupling of other carboxylic acids (6-TAMRA and NODA-GA­(^*t*^Bu)_3_) was performed in a similar manner to the manual Fmoc-AA coupling, using PyBOP instead of HATU as the coupling reagent and deploying only 3 equiv of carboxylic acid and PyBOP and 6 equiv of DIPEA.

#### General Procedure for Kaiser’s Test^166^

To determine the completeness of a coupling step, a small amount of resin was transferred into a reaction tube and 50 μL of each of the following solutions were added in this order: (I) 2.8 M ninhydrin in EtOH, (II) 0.04 M phenol in EtOH, (III) 0.02 mM KCN in pyridine. As references, the same mixture was prepared once without resin and once with a minute amount of glycine. The reaction mixtures were agitated for 5 min at 99 °C. A color change to blue-violet (Ruhemann’s purple) indicated the presence of free amino groups, requiring repetition of the previous coupling step.

#### General Procedures for Manual Loading of Resins

Manual loading of resins was performed in case of the synthesis of ACPPs, CPPs, and nACPPs, when a low degree of resin loading was necessary or when peptides were synthesized on Wang and 2-chlorotrityl chloride resin, as was the case for hexapeptide **1** and tetrapeptide **1a**, respectively. For manual loading of Rink Amide ChemMatrix resin (max. loading 0.7 mmol/g), the resin was swollen in NMP. After filtration, Aloc-l-Lys­(Fmoc)–OH (in NMP) was added to the resin at 1.25 equiv to the desired loading of approximately 0.1 mmol/g, as well as 2.5 equiv of PyBOP and 2.5 equiv of *N*,*N*-diisopropylethylamine (DIPEA). The suspension was agitated at RT for 48 h. Subsequently, resin loading was determined and, if the achieved degree of loading was within ±10% of 0.1 mmol/g, acetylation as described above, was performed to block remaining reaction sites. If the loading degree was too low for efficient synthesis, loading was repeated with a reduced amount of reagents. Typically, 1 g of resin was used to generate an initial batch of 100 μmol preloaded resin, which was then modified further on resin and thereafter subdivided into batches of 25 μmol for automated peptide synthesis.

For loading of Wang resin (0.7 mmol/g max. loading) in the synthesis of compound **1**, swelling was performed in CH_2_Cl_2_/DMF (9:1, v/v). After filtration, the resin was resuspended in the coupling solution consisting of 1.5 equiv Fmoc-l-Lys­(Dnp)–OH, 1.5 equiv 1-hydroxybenzotriazole, 1.5 equiv diisopropylcarbodiimide, and 0.1 equiv DMAP in DMF, and the resulting mixture was agitated at RT for 6 h. Subsequently, the resin was washed successively with DMF, CH_2_Cl_2_, and CH_3_OH (each 3 times) and dried *in vacuo*.

Loading of 2-chlorotrityl chloride resin (1.6 mmol/g) for the synthesis of compound **1a** was performed by adding a solution of Fmoc-l-Arg­(Pbf)–OH (0.7 equiv) and DIPEA (2.1 equiv) in DMF to the preswollen (CH_2_Cl_2_) resin. The resulting mixture was agitated for 3 h at RT, and the resin was washed with DMF and CH_2_Cl_2_ (4 times each). For blocking of unreacted linker sites, the resin was treated with CH_2_Cl_2_/CH_3_OH/DIPEA (v/v/v 17:1:2, 5 mL, 4 × 2 min), washed with CH_2_Cl_2_ (4 times), and dried *in vacuo*.

#### General Procedures for Peptide Cleavage from Resin and Side-Chain Deprotection

##### Test Cleavage

To determine the success of intermediate reaction steps during solid-phase synthesis, test cleavages were performed to liberate analytical amounts of peptide from the resin. To this end, a small amount of dried resin was transferred into a reaction tube and 100 μL of TFA/thioanisole/ethanedithiol (90:7:3, v/v/v) was freshly prepared *in situ* by first adding a preprepared solution of thioanisole/ethanedithiol (7:3, v/v) and then TFA. The suspension was agitated at RT for 1 h (3 h for peptides containing the polyarginine sequence), after which the crude peptide was precipitated by the addition of 1 mL ice-cold Et_2_O and subsequent incubation at −20 °C for 1 h. The precipitate was pelletized by centrifugation (1 min, 4 °C, 20,817 × *g*), washed five times with ice-cold Et_2_O and dried. Subsequently, the peptide was dissolved in 50% CH_3_CN/water (0.1% TFA) for analysis by mass spectrometry and RP-HPLC.

##### Full Cleavage

To completely cleave the peptide from the resin, the resin was treated with 3 mL of freshly prepared TFA/thioanisole/ethanedithiol (90:7:3, v/v/v) and agitated at RT for 3 h, after which the solution was collected and the resin washed three times with 1 mL of TFA. In case of the ACPP, CPP, and nACPP peptides (compounds **68**-**74**), volumes were tripled due to the significantly higher swelling capacity of the ChemMatrix resin. The crude peptide was subsequently precipitated by the addition of 40 mL of ice-cold Et_2_O and subsequent incubation at −20 °C for at least 1 h. The precipitate was pelletized by centrifugation (2 min, 4 °C, 7,197 × *g*), washed five times with ice-cold Et_2_O and dried (30 min at 65 °C). The crude yield was determined, and the crude product was analyzed by RP-HPLC to determine an appropriate gradient for subsequent purification by preparative RP-HPLC.

#### Reduction of Oxidized Methionine

After cleaving the peptide from the resin, the crude peptide containing oxidized methionine residues was dissolved in a freshly prepared solution of TFA/trimethylbromosilane/EDT (96/2.4/1.6, v/v/v) and then agitated for 30 min at RT.^167^ Subsequently, the peptide was precipitated by adding ice-cold Et_2_O and stored at −20 °C for at least 2 h. The precipitate was pelletized by centrifugation (2 min, 4 °C, 20,817 × *g*), and the pellet was redissolved in ice-cold Et_2_O. This washing process was repeated five times, after which the crude peptide was purified by RP-HPLC.

### General Procedures for Peptide Analysis and Purification

#### RP-HPLC

Depending on the availability of the systems, analytical and preparative RP-HPLC was performed on either of two systems: HPLC A (Varian Prepstar System with ProStar 325 UV–vis Detector) and HPLC B (Shimadzu Prominence System with RF-20A (fluorescence detector) and SPD-M20A (photodiode array detector)). Separation was performed by gradient elution, using a mobile phase system consisting of water (0.1% TFA, eluent A) and CH_3_CN (0.1% TFA, eluent B). Substances were dissolved in CH_3_CN/water (0.1%) at the ratio that equals the one at the start of the gradient and filtered using 0.22 μm syringe filters.

#### Analytical HPLC

Analytical RP-HPLC during synthesis was performed using a Microsorb Dynamax C_18_ column (length × diameter: 250 × 4.6 mm, particle size: 5 μm, pore size: 100 Å) on HPLC A and a Jupiter Proteo C_18_ column (250 × 4.6 mm, 4 μm, 90 Å) or Aeris Peptide 5 μm XB-C18 columns (100 Å, 250 × 4.6 mm) on HPLC B with the gradients shown in Tables S11–S14 in Supporting Information at a flow rate of 1 mL/min each.

#### Preparative HPLC

Preparative RP-HPLC was performed using a Microsorb Dynamax C_18_ column (250 × 21.4 mm, 5 μm, 100 Å) on HPLC A and a Jupiter Proteo C_18_ column (250 × 4.2 mm, 4 μm, 90 Å) on HPLC B. An appropriate gradient (10 mL/min flow rate) was chosen for each separation individually based on a previous analytical RP-HPLC using the same HPLC device and a corresponding column. A representative gradient for preparative RP-HPLC is shown in Table S15 in Supporting Information. Fractionation was performed when necessary, with fractions analyzed by the available MS instrument. After purification, purity was determined by analytical RP-HPLC and samples <95% pure were repurified. Pure products were freeze-dried and stored at −20 °C.

#### Radio-HPLC

Radio-HPLC for analysis of ^64^Cu-labeled products and detection of radiometabolites was performed using the equipment and procedures described previously.^161^

#### UPLC-ESI-ToF-MS

Samples for UPLC-ESI-ToF-MS were obtained either directly from RP-HPLC or prepared from dry substances in 25% CH_3_CN/water. Measurement and analysis were performed on a Waters Acquity I-Class UPLC with a BEH C18 column (100 × 2.1 mm, 1.7 μm, 130 Å) coupled to a Waters Xevo TQ-S ESI-ToF mass spectrometer, operated by the MassLynx V4.1 software. Separation was performed using a binary gradient of CH_3_CN/CH_3_OH (1:1, v/v, 0.1% Optima acetic acid, eluent A) and water (0.1% Optima acetic acid, eluent B) with a 0.4 mL/min flow rate, using one of the gradients listed in Tables S16–S18 in Supporting Information.

#### MALDI-ToF-MS

Samples for MALDI-ToF-MS were obtained either directly from RP-HPLC or prepared from dry substances by dissolving in CH_3_CN/water (3:7, v/v, 0.1% TFA). Samples were applied to an MTP 384 ground steel target plate by the spot-in-spot technique: 0.5 μL of a saturated solution of α-cyano-4-hydroxycinnamic acid (HCCA) in CH_3_CN/water (3:7, v/v, 0.1% TFA) was deposited first and 0.5 μL of analyte solution was subsequently applied directly into the still-wet HCCA droplet. Spots were allowed to dry at RT to form an analyte-HCCA cocrystal. Measurements were performed on a Bruker autoFlex-ToF/ToF unit operated by FlexControl V3.3, with subsequent evaluation by FlexAnalysis V3.3. Bruker’s Peptide Calibration Standard and Peptide Calibration Standard II were used for dual internal calibration and applied in the immediate vicinity of sample spots.

#### High-Resolution ESI-MS

High-resolution mass spectra of compounds **1**-**5**, **52**, **58**, **59**, **63**-**65**, and **67**-**74** were obtained on a Q-TOF MS using electrospray ionization: Agilent 1260 Infinity II HPLC (Santa Clara, California, USA; pump G7104C, autosampler G7129C, column oven G7116A, DAD detector G7117C) coupled to a γ detector Gabi Star (Raytest Isotopenmeßgeräte GmbH, Straubenhardt, Germany) followed by accurate mass Revident Q-TOF LC/Q-TOF G6575A. The measurements were performed in bypass mode using an eluent consisting of (A): CH_3_CN and (B): 0.1% formic acid in H_2_O; flow rate 0.2 mL/min. A reference mass solution containing hexakis­(1*H*,1*H*,3*H*-tetrafluoropropoxy)­phosphazene and purine was continuously coinjected via a dual AJS ESI source. The system was operated using Agilent MassHunter Workstation 3.6 LC/MS data acquisition software (Version 12.0), and data evaluation was performed using Agilent MassHunter Workstation 3.6 Qualitative Analysis software (Version 12.0 Update 1). Theoretical *m*/*z* values for different charge states were calculated using the MS prediction tool implemented in MestReNova (Version 15.1.0–38027).

### *In Vitro* Serum Stability Assay

A 10 μM solution of peptide in pooled human serum (purchased from Merck Millipore) was prepared in Protein LoBind tubes from a 10 mM DMSO stock. The solution was incubated at 37 °C and 700 rpm. Samples were taken before (*t*_0_) and at various time points during incubation (*t*_*x*_, to 72 h). Immediately after taking samples, an equal amount of an EtOH/CH_3_CN solution (1:1, v/v) was added to inactivate serum proteases and precipitate serum proteins. Samples were stored for at least 1 h at −20 °C to facilitate precipitation before centrifugation (5 min, 4 °C, 20,817 × *g*). Supernatants were then transferred into a 0.22 μm Corning Costar Spin-X centrifuge tube filter and centrifuged (5 min, 4 °C, 20,817 × *g*). The discharge was precipitated again for at least 1 h at −20 °C before further centrifugation (5 min, 4 °C, 20,817 × *g*). For analysis by UPLC, the sample solutions were diluted 1:5 in the solvent mixture that was identical to the one at the start of the gradient. Separation and detection were performed by a UPLC-ESI-ToF-MS as detailed above, using UPLC-gradient B. For quantitative comparison of peptide stability, absorbance measurements (λ = 365 nm) were analyzed and the ratio of the peptide peak area at *t*_0_ to the total peak area was defined as 100% stability. Stability of *t*_*x*_-samples was calculated as percentages of the *t*_0_-sample. All measurements were performed in triplicates.

### Methods for Cell Culture, Cell, and Tissue Lysis

The origin and cultivation of all investigated cell lines (U87MG, U251MG, A431, Mel Juso, SW403, SW480, SW620) are summarized in Table S2 in Supporting Information. In general, cultivation was carried out in the specified culture media in an incubator at 37 °C and 5% CO_2_ partial pressure. The cells were inspected every 2–3 days and passaged at a confluence of >95%, but at least after 5 days.

In particular, the human cell lines U87MG and Mel Juso were used as models for high and low expression of cathepsin B in tumor tissue, respectively, and the corresponding peptide-tissue interaction. Additionally, human vascular endothelial cells (HUVEC) were used to evaluate the potential interaction of the ACPP with endothelial tissue. U87MG and Mel Juso were cultivated using Dulbecco’s modified Eagle’s Medium with added 10% fetal bovine serum (FBS) and 1% penicillin/streptomycin, while HUVEC were cultivated using Cellovations Endothelial Cell Growth Media without further additives.

For Western blot expression analysis, the cells cultivated in cell culture flasks (75 cm^2^) were detached with 0.05% trypsin/0.02% EDTA and the enzymatic reaction was stopped with 5 mL of cell culture medium. The cells were centrifuged in an Eppendorf tube (5 min, 4 °C, 300 × *g*), and the cell pellet was washed three times with PBS. The cells were stored at −70 °C.

For lysis, the cell pellets were resuspended in 100 μL lysis buffer and incubated for 10 min at room temperature. The cells were broken down twice for 7 s using ultrasound (20%, pulsed) and cooled on ice for 5 min between lysis cycles. After centrifugation (15 min, 4 °C, 16,000 × *g*), the clear supernatant was transferred to a new Eppendorf tube and stored on ice or at −70 °C until further use.

The tissue pieces were transferred to gentleMACS M tubes for lysis and mixed with 200–500 μL of ice-cold lysis buffer. After 10 min of incubation on ice, the tubes were placed in the gentleMACS Octo Dissociator from Miltenyi Biotec and the samples were lysed using the preinstalled Protein_01 program. The samples were centrifuged (5 min, 4 °C, 4000 × *g*) and then treated with ultrasound (15 s, pulsed, 20%) to ensure complete lysis. After transfer to a 1.5 mL Eppendorf tube and recentrifugation (15 min, 4 °C, 16,000 × *g*), the clear supernatant was transferred to a new Eppendorf tube and stored at −70 °C or on ice until further use.

Protein concentration of the samples was determined using the Pierce BCA Protein Assay Kit from Thermo Fisher Scientific according to the method described by Smith et al.^168^

#### Preparation of Cell Culture Vessels for Cellular Assays

For U87MG and Mel Juso, IBIDI μ-slides and 96-well plates were treated using a coating solution of 0.1% poly-d-lysine in water (v/v). Enough coating solution was added to each well to completely cover the bottom, and the respective vessel was incubated at RT for 15 min. The solution was aspirated, and the well was washed with PBS, after which the cell culture vessel was dried at RT. The coating solution was used multiple times.

For HUVEC, T25 cell culture flasks as well as IBIDI μ-slides were treated using a solution of bovine collagen (0.1 mg/mL, w/V). The coating procedure was performed for 2 h at 37 °C, and washing and drying were performed as described before. The coating solution was not reused.

#### Passaging and Seeding for Cellular Assays

Detaching cells from the cell culture flask was necessary for passaging after cells reached full confluence and seeding in specific culture vessels for cellular experiments. Cells were washed twice with 5 mL of PBS and then incubated with 1 mL of trypsin/EDTA for 3 min at 37 °C. Gentle tapping at the side of the culture vessel facilitated the detachment. Subsequently, 11 mL of cell culture medium were added to stop trypsinization. Cells were split in desired ratios from 1:2 to 1:36 into new cell culture flasks filled with new medium for further cultivation or seeded into cell culture vessels for assays. Depending on the cell line and assays, a different number of cells was seeded: For fluorescence microscopy, 150,000 cells of either U87MG or Mel Juso, and 50,000 of HUVEC, were seeded per IBIDI μ-slide well. For enzyme kinetic measurement, 60,000 U87MG were seeded per cavity in a 96-well plate, with the outermost columns (A/B) and rows (1/12) filled only with PBS. Seeding of IBIDI μ-slides and 96-well plates was performed on the day prior to the respective experiments. Cell numbers were determined using a Neubauer Improved counting chamber.

### Western Blot and Immunohistochemistry

#### Western Blot

Western blot for assaying cellular expression of cathepsins B, K, L, and S was performed as reported.^57^ Information on the investigated cell lines and the used primary antibodies are included in Tables S2 and S3, respectively, in Supporting Information.

#### Cell fractionation for determining subcellular cathepsin localization

##### Membrane Fraction

The membrane fraction was isolated using the Pierce Cell Surface Protein Isolation Kit from Thermo Scientific as described in the kit. For each sample, four subconfluent cell culture flasks (75 cm^2^) were washed with PBS and biotinylated. After stopping the reaction with quenching solution, the cells were scraped off, transferred to a Falcon tube (50 mL), and centrifuged to obtain the pellet. The cells were lysed, and the biotinylated membrane proteins were separated using NeutrAvidin resin. Elution was performed using SDS-PAGE sample buffer with DTT. The samples obtained were analyzed by Western blot without further dilution.

##### Lysosome Fraction

The lysosome fraction was isolated using the Lysosome Enrichment Kit for Tissue and Cultured Cells from Thermo Scientific as described in the kit.

The cells were cultivated in a cell culture flask (75 cm^2^) and the cell pellet was obtained as described above (50–200 mg of cells). The pellets were lysed with the enclosed Lysosome Reagents A and B, and the lysosomes were separated by density gradient centrifugation. The lysosome fraction was removed, diluted 2-fold with PBS, and centrifuged (30 min, 4 °C, 16,100 × *g*), and the pellet was resuspended in dilution buffer and centrifuged again. The supernatant was discarded.

The lysosomes were then resuspended in 100 μL lysis buffer and, after an incubation period of 10 min on ice, broken down using ultrasound (2 × 7 s, 20%). After determining the protein concentration, the samples were diluted to 0.65 mg/mL with lysis buffer and sample buffer (4:1) and analyzed by Western blot. In total, 13 μg protein/bag was applied to the gel in each case.

##### Cell Culture Supernatant

To detect cathepsins in the cell culture supernatant, the cells were cultured in a 25 cm^2^ cell culture flask to approximately 90% confluence and then cultured for 24 h in the absence of serum. The cell medium was transferred to a 15 mL Falcon centrifuge tube and centrifuged (5 min, 4500 × *g*). The cell pellet was recovered and lysed, and the protein concentration of the lysate was determined as described above. The upper layer of the cell medium was discarded, and 5 mL of cell culture supernatant was transferred to a Vivaspin 6 tube (MWCO 10000). The samples were centrifuged for approximately 15 min (10,000 × *g*, fixed rotor), but at least until the sample was reduced to less than 200 μL. The concentrated medium was filled up to 200 μL with lysis buffer and mixed with 50 μL sample buffer. After heating in a thermomixer (5 min, 95 °C), the samples were analyzed by Western blot. The application was proportional to the protein concentrations measured in the cell lysates.

#### Immunohistochemistry

Immunohistochemical staining of tumor xenograft sections for cathepsins B, L, S, and K was performed as preciously described^169^ using the identical primary antibodies as for the Western blot reported in Table S3 in Supporting Information.

### *In Vitro* Enzyme Kinetics

Determination of the kinetic properties of all octapeptide substrates was conducted on isolated cathepsin B. Selected substrates were furthermore investigated regarding their kinetic properties toward cathepsin K, cathepsin L, and cathepsin S, as well as directly on viable U87MG cells. Substrates were always prepared from a 10 mM stock solution in DMSO. A Synergy 4 Microplate Reader (Biotek) was used for fluorescence measurement (λ_em/ex_ = 325/410 nm). The reader and all solutions were preheated to 37 °C. The tetrapeptide **1a** was used as a reference compound to regularly generate calibration measurements to enable the conversion of fluorescence values into Δ*c*-values. Solutions containing DTT were stored at −20 °C for a maximum of 3 months.

#### Enzyme Kinetics Using Isolated Enzyme

The composition of assay buffers A and B and the composition of the various enzyme buffers are listed in Tables S19 and S20 in Supporting Information, respectively.

Substrate working solutions were created by serial dilution from DMSO stock in assay buffer B, and 20 μL of substrate working solution was added per cavity of a 96-well plate, along with 170 μL of assay buffer A. The combined solution was incubated for 10 min at 37 °C to ensure complete intermixture. In parallel, the enzyme was prepared by first diluting the enzyme stock in the respective enzyme buffer to an initial concentration of ≈60 μg/mL (the exact concentration achieved in this step depended on the individual enzyme batch and was modified to achieve to easily reach the necessary 5 μg/mL in the next step). This solution was diluted further in assay buffer C into the final 5 μg/mL enzyme working solution. The enzyme working solution was preactivated at 37 °C for 5 min, before 10 μL of the enzyme working solution was added per cavity for a final enzyme concentration of 0.25 μg/mL per well. Kinetic measurement was immediately started after adding the enzyme and performed over a period of 15 min. For each substrate and enzyme, three independent measurements were performed in duplicates for six different concentrations. Data analysis was performed by either fitting the parameters of the Michaelis–Menten equation by nonlinear regression or linear regression according to the Hanes–Woolf equation, depending on substrate solubility (GraphPad Prism V6.0).

### Structure–Kinetics Correlation Analysis

Van-der Waals (v-d-W) surfaces for the P4′ residues were calculated using the Chemicalize online platform developed by ChemAxon.^170^ The value for bonded hydrogen (P4′ = Gly) was calculated from the difference of the v-d-W surfaces of methane and the half-value of the v-d-W surface area of ethane according to eq 5.

5 $$A_{\mathrm{vdW}}\left(H\right)=A_{\mathrm{vdW}}\left(CH_{4}\right)-\frac{A_{\mathrm{vdW}}\left(C_{2}H_{6}\right)}{2}=11.89{Å}^{2}$$

The v-d-W surface area of the various substituents was calculated from the values of the corresponding hydrocarbons (RH) as follows in eq 6:

6 $$A_{\mathrm{vdW}}\left(R\right)=A_{\mathrm{vdW}}\left(RH\right)-A_{\mathrm{vdW}}\left(H\right)$$

The following surface area values were used for the correlation analysis: H: 11.89 Å^2^, Me: 46.28 Å^2^, Et: 80.67 Å^2^; *i*Pr: 111.94 Å^2^, *c*Pr: 88.64 Å^2^, *t*Bu: 141.54 Å^2^, *c*Bu: 116.63 Å^2^, *c*Pe: 145.10 Å^2^.

### Evaluation of Cellular Uptake of 6-TAMRA-Modified Compounds

The complete procedure is described once in the paragraph below with subsequent sections outlining modifications of the setup for individual experiments. If not stated otherwise, solutions were at RT when added to the cells. The assays were performed under nonsterile conditions.

#### Determination of Cell Line-Dependent Uptake Kinetics (U87MG, Mel Juso, HUVEC)

The cell culture medium was aspirated, and cells were washed once with PBS. Cells were preincubated with 198 μL of serum-free OptiMEM (0.5 mM DTT) at 37 °C for 30 min. The 6-TAMRA-labeled construct of interest added was diluted from a 1 mM stock solution in DMSO to a final concentration of 5 μM. This was specifically performed by momentarily removing the 198 μL medium from the cells, adding the peptide, rapidly agitating the solution, and transferring it back to the cells to ensure homogeneous distribution. As a vehicle control, 1% DMSO in serum-free OptiMEM (0.5 mM DTT) was used. Cells were subsequently incubated for up to 30 min at 37 °C. To assess relevant features of cellular uptake, incubation was also performed without DTT and at 4 °C. The cells were then washed twice by 20 s incubation with acidic washing buffer (0.2 M glycine, 0.15 M NaCl, pH 3.0) at RT and once with PBS. Thereafter, nuclear staining was performed by 5 min incubation with 100 μg/mL Hoechst 33258 in serum-free OptiMEM, after which the staining solution was again replaced by serum-free OptiMEM. Microscopy studies were performed on an Olympus IX83 microscope (FV10-ASW V04.00.02.09), with fluorophore excitation/detection at λ_ex/em_ = 555/655 nm (6-TAMRA) and λ_ex/em_ = 425/475 nm (Hoechst 33258). Using ImageJ (V1.52e), the mean fluorescence intensity was determined for both wavelengths. Fluorescence for the peptide (6-TAMRA) was normalized to nuclei (Hoechst 33258) for each image to account for the varying number of cells per image. Subsequently, those values were normalized to the vehicle control (= 0% uptake) and uptake in U87MG after 30 min (37 °C, 0.5 mM DTT, = 100% uptake). Statistical analysis was performed using GraphPad Prism (V6.0).

#### Determination of Activation Specificity and Cathepsin B-Export Pathway by Inhibition (U87MG)

Inhibitors were added into the preincubation medium at 10 μM or 100 μM concentration, respectively, before preincubation. Incubation with the final peptide solution was performed for 15 min at 37 °C. Washing and nuclear staining were performed as described above. For reference, a vehicle control (1% DMSO) was used as the negative control (= 0% uptake) and 5 μM ACPP-(6-TAMRA) (= 100% uptake).

#### Induction of ACPP Uptake in Cathepsin B-Negative Cells (Mel Juso, HUVEC)

Induction of ACPP uptake was performed by adding either cathepsin B directly into the incubation medium or by conditioning the medium on U87MG cells directly before the assay. When directly adding cathepsin B, the enzyme was prepared as described above (section *In Vitro* Enzyme Kinetics); each well received 188 μL of serum-free OptiMEM (0.5 mM DTT), 10 μL of enzyme solution, and 2 μL of 1 mM ACPP-(6-TAMRA) in DMSO (final concentration: 5 μM). In preparation of conditioning, a T25 cell culture flask was seeded with 3.75 × 10^6^ U87MG at the same time as the respective μ-slides. For conditioning, cell culture medium was aspirated, the cells were washed once with PBS, and 5 mL of serum-free OptiMEM (0.5 mM DTT) were added. The medium was conditioned for 30 min at 37 °C, after which the medium was removed and filtered through a 0.22 μM syringe filter. Each well received 198 μL of conditioned serum-free OptiMEM (0.5 mM DTT) and 2 μL of 1 mM DMSO-stock of ACPP-(6-TAMRA). Incubation with the final peptide solution was performed for 15 min at 37 °C. Washing and nuclear staining were performed as described above. Treatment of U87MG with vehicle control (1% DMSO, = 0% uptake) and 5 μM ACPP-(6-TAMRA) in nonmodified, preincubated medium (= 100% uptake) served as positive and negative control references, respectively.

### Radiochemistry and Radiopharmacology

#### ^64^Cu-Labeling

[^64^Cu]­CuCl_2_ was produced at the Helmholtz-Zentrum Dresden-Rossendorf on the 30 MeV TR-Flex cyclotron (Advanced Cyclotron Systems Inc., ACSI, Canada) using the nuclear reaction ^64^Ni­(p,n)^64^Cu as reported previously.^171,172^ Peptides (1 μL of 2 mM DMSO stock, 2 nmol) were labeled with [^64^Cu]­CuCl_2_ (100 MBq, 98 μL) in ammonium acetate buffer (pH 5.6, 20 min, 80 °C). Accordingly, the apparent molar activity (molar activities calculated based on the applied peptide amount; no separation of nonlabeled peptide was conducted after radiolabeling) was 50 GBq/μmol. The radioligand stock solutions (0.2 M NH_4_OAc, 1 MBq/μL, 40 μM) were diluted with PBS (10 mM, pH 7.4) or 0.154 M NaCl for further experiments.

#### Cell Binding of ^64^Cu-labeled ACPP and Control Probes

Cells (U87MG and Mel Juso) were seeded in 24-well plates (freshly coated with poly-d-lysine) 4 days prior to the experiment (U87MG: 2 × 10^5^ cells/well, Mel Juso: 4 × 10^5^ cells/well). For the binding assay, OptiMEM was used as the medium for both cell types. Prior to the experiment, all cells were washed once with fresh medium. For determining binding, [^64^Cu]­Cu-**71**, [^64^Cu]­Cu-**73**, and [^64^Cu]­Cu-**74** were each diluted to 25 nM in OptiMEM and added to the cells. The plates were incubated at 37 °C for 1 h (shaken at 300 rpm). After incubation, cells were washed twice with cold PBS^+^ (PBS containing 492 μM MgCl_2_ and 603 μM CaCl_2_ for approximately 5 min). For determining internalization, one wash step with cold PBS^+^ was replaced by treatment with cold glycine buffer (50 mM, pH 2.8) for 5 min. Subsequently, the cells were lysed with 0.1% SDS in 0.1 M NaOH. The lysates were measured in a γ-counter (PerkinElmer Wizard 3″). The obtained activity values were corrected by subtraction of the background values obtained from empty wells identically treated in the absence of cells. The results were normalized to the protein content (measured at *A*_280 nm_ with the setting 1 Abs = 1 mg/mL) of the lysates determined with a NanoDrop spectrophotometer (Thermo Fisher Scientific).

#### Plasma Stability Assay

Lyophilized human plasma was obtained as previously reported^173^ and reconstituted in deionized water (351 mg of lyophilizate were dissolved in 4 mL of water). Solutions of compound [^64^Cu]­Cu-**71** or [^64^Cu]­Cu-**74** (each 20 μL, approximately 10 MBq) were added to 350 μL of plasma and incubated for 24 h at 37 °C. The plasma proteins were precipitated by the addition of double the volume of a solution named “Supersol” (20% EtOH, 0.5% Triton X-100, 0.1% saponin, 5 mM EDTA, 0.5 mM *o*-phenanthroline), and the resulting mixture was centrifuged and analyzed by radio-HPLC (see below for conditions and Figure S27 in Supporting Information for chromatograms).

### Radiopharmacological Investigations *Ex Vivo* and *In Vivo*

#### Animal Experiments in Healthy Wistar Rats

Animal experiments with healthy Wistar rats were carried out according to the guidelines of the German Regulations for Animal Welfare. The protocols were approved by the local Ethical Committee for Animal Experiments (DD24.1–5131/450/16).

Stability and biodistribution *ex vivo* of [^64^Cu]­Cu-**71** and [^64^Cu]­Cu-**73** were studied in normal Wistar rats as reported in ref 169 with slight adjustments. In brief, a desflurane-anaesthetized male rat (body weight: 481 g) was intravenously injected with 105 MBq (4.2 nmol) [^64^Cu]­Cu-**73**. At selected time points (3/30/60 min p.i.), arterial blood samples were obtained via a femoral artery catheter with subsequent liquid substitution by E153 electrolyte solution. After determination of activity concentration in blood, blood plasma was obtained by centrifugation for 3 min at 13,500 rpm (17.5 × *g*) and −3 °C. Again, the activity concentration was measured in blood plasma. Afterward, plasma proteins were precipitated by the addition of “Supersol” (composition given above) into blood plasma in a ratio of 2:1. The samples were centrifuged and analyzed by radio-HPLC as described previously.^161^

Blood activity values were analyzed for the determination of pharmacokinetic parameters (Figure S23 and Table S5 in Supporting Information) as previously described.^174^

#### Animal Experiments in U87 Tumor-Bearing Mice

All animal experiments with U87 tumor-bearing mice were carried out according to the guidelines of the German Regulations for Animal Welfare. The protocols were approved by the local Ethical Committee for Animal Experiments (DD24.1–5131/449/49). To generate tumor xenografts for PET imaging, NMRI nude mice (Rj:NMRI-*Foxn1* ^*nu/nu*^, Janvier) were subcutaneously injected with 5 × 10^6^ U87MG cells in PBS on the right hind leg. Tumor size was monitored three times a week by caliper measurements. Tumor-bearing mice were included in the imaging experiments about 4 weeks post-tumor cell injection, when tumors reached a volume of at least 400–700 mm^3^.

#### PET Imaging in Tumor-Bearing Mice

Small animal positron emission tomography (PET) using the nanoScan PET/CT scanner (Mediso Medical Imaging Systems) was performed as previously described.^175^ In brief, U87 tumor-bearing mice were anesthetized using desflurane, positioned and immobilized prone with their medial axis parallel to the axis of the scanner. PET acquisition was started 20 s before the intravenous injection of the radiotracer. Mice received 8–12 MBq of radiotracer delivered in 0.2 mL of 0.9% NaCl + 2% DMSO v/v through a tail vein catheter, corresponding to 100–140 pmol for [^64^Cu]­Cu-**71**, [^64^Cu]­Cu-**73**, and [^64^Cu]­Cu-**74**. Emission data were acquired continuously at the dedicated time points (0–60 min p.i. dynamic PET scan, 4/24 h p.i. static PET scans). With each PET scan, a corresponding CT image was documented and used for anatomical referencing and attenuation correction. PET data were reconstructed using Mediso Tera-Tomo 3D iterative reconstruction. Images were postprocessed and analyzed using ROVER (ABX) and displayed as maximum intensity projections (MIPs) at the indicated time points and scaling. For PET data quantification, 3D volumes of interest (VOI) were created by applying a fixed threshold (10% for tumor and muscle) for delineation of the organs of interest and subsequent determination of standardized uptake values (SUV_mean_) and time-activity curves (TACs).

To prove the involvement of cathepsin B in pharmacokinetics, in particular tumor uptake, PET imaging with [^64^Cu]­Cu-**71** was performed in the presence of the cathepsin B inhibitor CA074 (10 mg/mL in 0.9% NaCl + 2% DMSO, 10 mg/kg body weight; *n* = 4) in comparison to the control, i.e., the absence of CA074 (injection of vehicle solution, 0.9% NaCl + 2% DMSO; *n* = 5).

#### Biodistribution Experiments

Organ distribution of [^64^Cu]­Cu-**71** and [^64^Cu]­Cu-**73** in U87 tumor-bearing mice was performed as described elsewhere with some modifications.^176^ In brief, mice were i.v. injected with 0.1–1.1 MBq of [^64^Cu]­Cu-**71**, [^64^Cu]­Cu-**72**, [^64^Cu]­Cu-**73**, or [^64^Cu]­Cu-**74**, corresponding to about 16 pmol. At dedicated time points (1/4/24 h p.i., each *n* = 4), narcotized mice were sacrificed via cardiac puncture and cervical dislocation. In addition to blood, major organs (e.g., liver, kidney, spleen, lung, muscle, femur) as well as tumors were taken from the sacrificed mice and analyzed for weight and radioactivity concentration. SUV_mean_ values were calculated as a percentage of recorded radioactivity at *t*_*x*_ with respect to the initially injected radioactivity *t*_0_ and the body weight of individual mice.

To prove the involvement of cathepsin B in organ distribution, particularly tumor uptake, the biodistribution of [^64^Cu]­Cu-**71** was investigated in the presence of the cathepsin B inhibitor CA074 (10 mg/mL in 0.9% NaCl + 2% DMSO, 10 mg/kg body weight; *n* = 4 for each time point), in comparison to the control, i.e., the absence of CA074 (injection of vehicle solution only, 0.9% NaCl + 2% DMSO; *n* = 4 for each time point).

#### Metabolic Stability

In accordance to the investigation of metabolic stability in healthy Wistar rats, the metabolic fate of [^64^Cu]­Cu-**71** and [^64^Cu]­Cu-**73** was studied also in U87MG tumor-bearing mice to get insights, in particular, into the proteolytic conversion of [^64^Cu]­Cu-**71** to [^64^Cu]­Cu-**73** in the presence of cathepsin B. The experimental procedure is the same as for rats with the following minor modifications. Mice were injected i.v. with at least 50 MBq and sacrificed at 1 h or 18 to 24 h p.i. Blood and organs of interest were obtained and subjected to metabolite analysis by radio-HPLC as noted above and previously reported.^161^

## Computational Methods

### Modeling of Substrate Compounds

The crystallographic structure of the propeptide (i.e., residues 39-KRLCGTFL-46, PDB ID 3PBH, 2.5 Å)^72^ was used as a template to model the 3D structure of substrates **4** and **11** in MOE.^177^ This template structure was manually reoriented along the *y*-axis toward the substrate binding site of cathepsin B (PDB ID 3PBH), with residue 39 of the propeptide template occupying the spatial position corresponding to residue 46 in the reference structure. Then, mutations were introduced to model compound **4** (1-GIVRAK­(Dnp)­GG-8) and to incorporate the Abz and amide groups at the N- and C-termini, respectively. Next, dihedral angle scans of the peptide backbone and side chains were performed to maximize the structural alignment of compound **4** side chains at positions P2 (i.e., Val3, referred to in Figure 4 as Val2) and P4′ (i.e., Gly8, referred to in the main text as Gly4′) to the cathepsin B propeptide residues Thr^P^44 (S2 site) and Leu^P^41, respectively (Figure S4A in Supporting Information), and to exclude any potential steric clashes with protein residues at the binding site. Mutation of Gly8 to Val in **4** were introduced to model the 3D structure of compound **11**.

The so-modeled extended conformation of **4** and **11** was confirmed by predictions performed with the AlphaFold3 server.^73^ Of note, the specific functionalizations of compound **4** mentioned above (i.e., Dnp, Abz, and amide groups) are not supported in AlphaFold3. Therefore, predictions with the AF3 server were made with the natural sequence GIVRAKGG.

### Molecular Docking

The protein–protein docking protocol from MOE was used for predicting binding of compounds **4** and **11** to cathepsin B. The cathepsin B crystallographic structures having the occluding loop in closed (PDB ID 1GMY, 1.9 Å)^71^ and open (PDB ID 3PBH, 2.5 Å)^72^ conformations were used. Their cocrystallized ligands were excluded. The binding site was defined along cathepsin B residues Arg21-Ala31, Met66-Ala77, Cys108-Thr125, Gly172-Lys184, Glu194-Ala200, Ala218-Phe230, and Gly241-Val247. Protein and modeled substrate compounds **4** and **11** were considered rigid, and the *hydrophobic patch* potential was used. The rigid body method with default parameters for pose refinement was applied. The top 10 solutions were visualized, and the best-scored poses at the substrate binding site with substrate residue Val3 in pocket S2 or in its close proximity were selected. Figures were created with PyMOL.^178^

## Supplementary Material



## Notes

Supplementary figures and tables for enzyme kinetics, fluorescence microscopy, serum stability, radiopharmacology, supplementary figures for molecular modeling, Western blot and immunohistochemistry, supplementary tables for the experimental section, synthesis and analytics of compound **78**, and analytical documentation of synthesized substrates and probes (PDF)

## #

Department of Nuclear Medicine, University Hospital Carl Gustav Carus, Technische Universität Dresden, Fetscherstraße 74, 01307 Dresden, Germany

## ◆

Lydia Strohbach, née Behring
