# Supplementary material for: Development of Cathepsin B‑Activatable Cell-Penetrating Peptides for Tumor Targeting
Source: ACS Pharmacol Transl Sci. 2026 Feb 18;9(4):966–96. doi: 10.1021/acsptsci.5c00746 (PMC13077498; doi:10.1021/acsptsci.5c00746)
Supplement: Supplementary file 1 [file pt5c00746_si_001.pdf]

SUPPORTING INFORMATION

Development of cathepsin B-activatable cell-penetrating peptides for tumor targeting

Konstantin Kuhne,[1,4] Lydia Strohbach,[1,4,‡] Christin Neuber,[1,4] Robert Wodtke,[1,4] Gloria Ruiz-Gomez,[2] Birgit Belter,[1,4] Florian Brandt,[1,5] Lara Gluhacevic von Krüchten,[3] Max Keller,[3] M. Teresa Pisabarro,[2] Klaus Kopka,[1,4] Jens Pietzsch,[1,4] Reik Löser[1,4,*]

[1] Institute of Radiopharmaceutical Cancer Research, Helmholtz-Zentrum Dresden-Rossendorf, Bautzner Landstrasse 400, 01328 Dresden, Germany

[2] BIOTEC, Technische Universität Dresden, Tatzberg 47-49, 01307 Dresden, Germany

[3] Institute of Pharmacy, Faculty of Chemistry and Pharmacy, Universität Regensburg, Universitätsstraße 31, 93053 Regensburg

[4] Faculty of Chemistry and Food Chemistry, School of Science, Technische Universität Dresden, 01069 Dresden, Germany

[5] present address: Department of Nuclear Medicine, University Hospital Carl Gustav Carus, Technische Universität Dresden, Fetscherstraße 74, 01307 Dresden, Germany

‡ Lydia Strohbach *née* Behring
* Corresponding author, r.loeser@hzdr.de

Content

Evaluation of inner filter effect 2

Determination and analysis of kinetic parameters 5

Supplementary figures for molecular modeling of cathepsin B-substrate complexes.......... 6

Substrate degradation in blood serum structure-kinetics relationships forGFLG-derived octapeptides 7

Synthesis of the carbamoylated arginine building block Fmoc-Arg(ec,Boc)-OH (**78**)............10

Cell lines and antibodies for cathepsin expression analysis 13

Results of Western blot-based expression analysis..15

Immunohistochemical detection of cysteine cathepsins in tumor tissue sections...............18

Proof of specific uptake of TAMRA-ACPP (**68**) 21

Supplementary material for radiopharmacological characterization of [^{64}Cu]Cu-NODAGA-ACPP ([^{64}Cu]Cu-**71**) and analogues 24

Supplementary tables for experimental section..41

Analytical documentation of synthesized substrates and probes 43

References for Supporting Information 121

Evaluation of inner filter effect

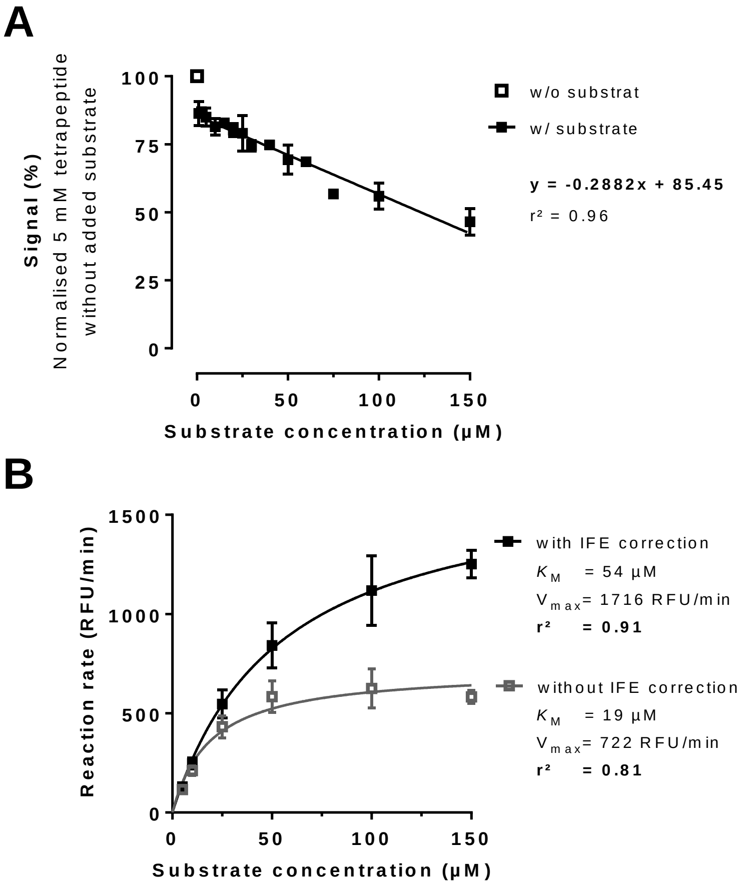

Figure S1: Correction for IFE in FRET-assay-derived data. (**A**) Empiric determination of the IFE correction factor. Signal attenuation for 15 different substrates at various concentrations was determined in comparison to 5 µM Abz-GIVR-COOH in assay buffer A without added substrate. The averaged attenuation values for all substrates were plotted, and the concentration-dependent correction factor was determined by linear regression. (**B**) Effect of IFE correction on kinetic data. While the impact of IFE correction on the initial values and subsequently the reaction rates is minimal at $c < 25$ µM, the effect becomes pronounced at higher concentrations.

Substrate concentration ranges and solubility

Table S1: Concentration ranges (maximum and minimum of 6 different concentrations each) employed for the kinetic characterization of the substrate compounds towards cathepsin B-catalyzed conversion. The determination of the solubility limits were determined by considering the concentration dependence of the absorption in the range around 600 nm as exemplarily shown for compounds **5**, **11** and **37** in Figure S2.

cpd	P3	P2	P1	P1‘	P3‘	P4‘	Substrate concentration (µM)	
							minimum	maximimum
**1**	I	V	R	A	-	-	10	100
**2**	I	V	R	A	-	-	10	150
**3**	I	V	R	A	G	-	5	100
**4**	I	V	R	A	G	G	5	100
**5**	I	V	R	A	G	S	5	150
**6**	I	V	R	A	G	A	5	75
**7**	I	V	R	A	G	2Abu	5	150
**8**	I	V	R	A	G	Nva	2.5	30
**9**	I	V	R	A	G	P	2.5	50
**10**	I	V	R	A	G	Nle	5	50
**11**	I	V	R	A	G	V	5	150
**12**	I	V	R	A	G	I	5	50
**13**	I	V	R	A	G	Tle	5	50
**14**	I	V	R	A	G	L	5	50
**15**	I	V	R	A	G	Cprg	5	50
**16**	I	V	R	A	G	Cbg	2.5	50
**17**	I	V	R	A	G	Cpeg	2.5	30
**18**	I	V	R	A	G	Phg	2.5	30
**19**	I	V	R	A	G	F	2.5	30
**20**	I	V	R	A	G	Y	2.5	40
**21**	I	V	R	A	G	H	5	150
**22**	I	V	R	A	G	W	2.5	20
**23**	I	V	R	A	G	T	5	150
**24**	I	V	R	A	G	Hse	5	100
**25**	I	V	R	A	G	C	2.5	50
**26**	I	V	R	A	G	M	2.5	50
**27**	I	V	R	A	G	D	10	200
**28**	I	V	R	A	G	E	5	150
**29**	I	V	R	A	G	N	10	200
**30**	I	V	R	A	G	Q	5	150
**31**	I	V	R	A	G	Orn	5	150
**32**	I	V	R	A	G	K	5	150
**33**	I	V	R	A	G	R	5	150
**Abz-GIV-P1-RAK(Dnp)-GS-CONH$_2$**								
**34**	I	V	hArg	A	G	S	5	150
**35**	I	V	nArg	A	G	S	5	150
**36**	I	V	r	A	G	S	5	150
**37**	I	V	Cit	A	G	S	2.5	25
**Abz-GFLGAK(Dnp)GS-CONH$_2$**								
**38**	F	L	G	A	G	S	10	100
**Abz- G-P3-P2-P1-P1‘-Lys(Dnp)-Sar-P4‘-CONH$_2$**								
**39**	I	V	R	A	Sar	S	5	150
**40**	I	V	R(ec)	A	Sar	S	5	100
**41**	F	L	G	A	Sar	S	2.5	25
**42**	F	L	G	A	Sar	V	0.5	10
**43**	F	L	G	A	Sar	G	2.5	25
**44**	F	L	G	A	Sar	A	2.5	25
**45**	F	L	G	A	Sar	L	2.5	25
**46**	F	L	G	A	Sar	F	2.5	25
**47**	F	L	G	A	Sar	Y	2.5	25
**48**	F	L	G	A	Sar	E	2.5	25
**49**	F	L	G	A	Sar	Q	2.5	25
**50**	F	L	G	A	Sar	Orn	2.5	50
**51**	F	L	G	A	Sar	K	2.5	50
**52**	F	ACBC	G	A	Sar	V	2.0	12.5
**53**	L	F	G	A	Sar	V	2.0	12.5

**54**	L	F(3-Me)	G	A	Sar	V	2.0	12.5
**55**	L	F(3-I)	G	A	Sar	V	2.0	12.5
**56**	F	L	Cit	A	Sar	V	2.0	12.5
**57**	F	F	Cit	A	Sar	V	2.0	12.5
**58**	F	V	Cit	A	Sar	V	2.0	12.5
**59**	F	ACBC	Cit	A	Sar	V	2.0	12.5
**60**	L	F(3-Me)	Cit	A	Sar	V	2.0	12.5
**61**	I	V	Cit	A	Sar	V	2.0	12.5
**62**	I	ACBC	Cit	A	Sar	V	2.0	12.5
**63**	I	V	R(ec)	A	Sar	V	2.0	12.5
**64**	I	F(3-Me)	R(ec)	A	Sar	V	2.0	12.5
**65**	I	F(3-I)	R(ec)	A	Sar	V	2	8
**66**	L	F	G	F	Sar	V	2	10
**67**	L	F	Cit	F	Sar	V	2	10

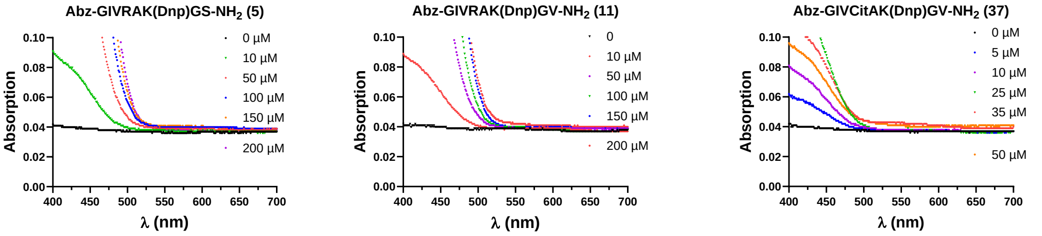

Figure S2: Determination of substrate solubility in cathepsin B assay buffer (1 % DMSO, 37°C) via absorption measurement as exemplarily shown for compounds **5**, **11**, **37**. The absorption values are not corrected for background absorption. Measurements were started after a mixing time of 15 min at 37°C each.

Determination and analysis of kinetic parameters

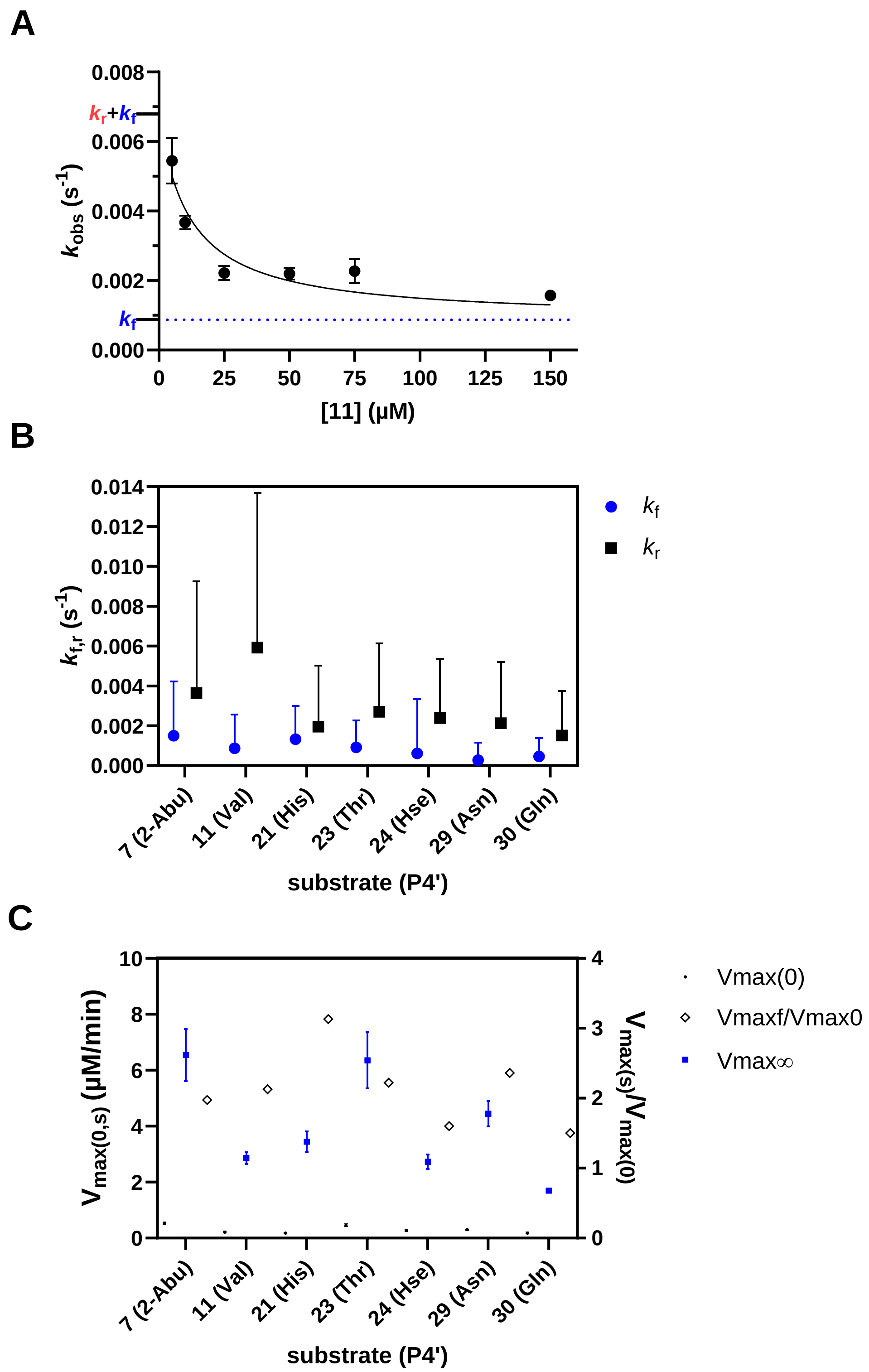

Figure S3: Determination and analysis of kinetic parameters derived from the hysteretic conversion of selected GIVRAK-derived octapeptides by cathepsin B. (**A**) Replot of pseudo-first order rate constants k_{obs} against substrate concentration ([S]) and regression curve for determination of first-order rate constant k_r and k_f exemplarily shown for compound **11**. Their locations on the y axis are each highlighted. (**B**) Plot of k_r and k_f for different P4' residues indicate no systematic variation with side chain structure. (**C**) Plot of maximum velocities for initial ($V_{max(0)}$) and final ($V_{max(s)}$) rates and derived ratios $V_{max(s)}/V_{max(0)}$. As the first-order rate constants, the ratios do not systematically vary for different side chains. Except for $V_{max(s)}/V_{max(0)}$ all data are plotted ± SEM. In (**B**), lower error bars are omitted for clarity.

Supplementary figures for molecular modeling of cathepsin B-substrate complexes

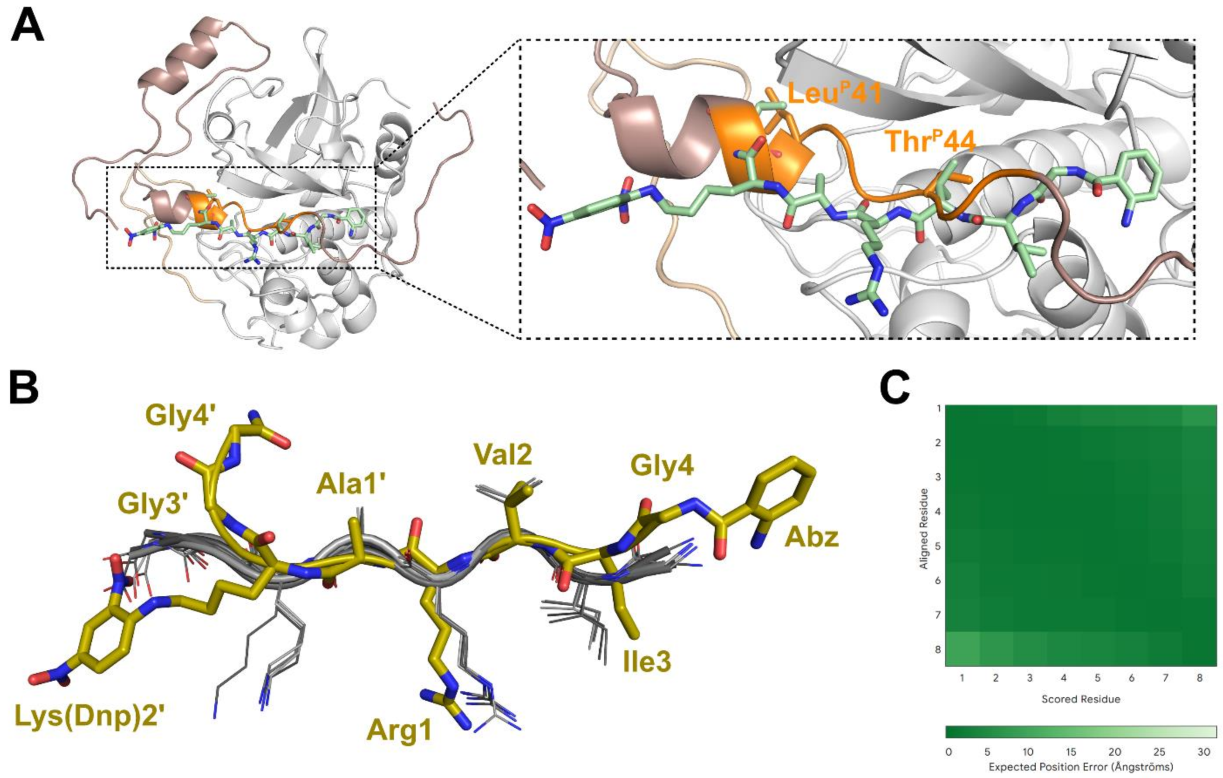

Figure S4: Molecular modeling of substrates **4** and **11**. (**A**) Superimposition of the model obtained with MOE for substrate **11** (green sticks) and the propeptide (brown; template region highlighted in orange) in complex with cathepsin B (gray) (PDB ID 3PBH, 2.5 Å). (**B**) Superimposition of the model obtained with MOE for substrate **4** (olive sticks) and top 5 models predicted for the unmodified octapeptide GIVRAKGV with AlphaFold3 (gray gradient lines from darkest (top1) to lightest (top5)). Figure created with PyMOL v.2.4.1. (**C**) Predicted aligned error score of substrate **4** modeled with AlphaFold3.

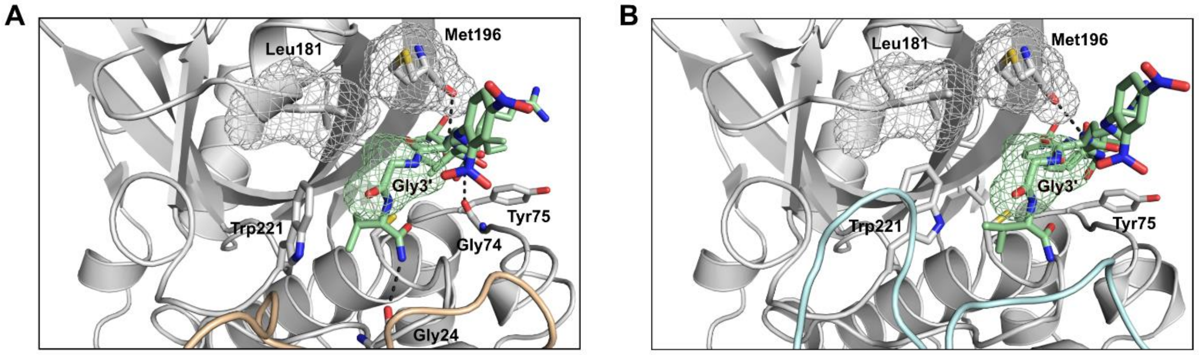

Figure S5: Modelling of cathepsin B in complex with substrate **11**. Cathepsin B is shown as a grey cartoon with the occluding loop in (**A**) open (PDB ID 3PBH, 2.5 Å) and (**B**) closed (PDB ID 1GMY, 1.9 Å) conformations in pale and cyan, respectively. Interacting residues are shown as sticks, colored by atom-type and labeled. Substrate **11** is shown in green sticks, colored by atom-type and labeled. The surface mesh denotes van der Waals contacts of cathepsin B Leu181 and Met196 with substrate residue Gly3'. H-bonds are depicted with black dashed lines. Figure created with PyMOL v.2.4.1.

Substrate degradation in blood serum and structure-kinetics relationships for GFLG-derived octapeptides

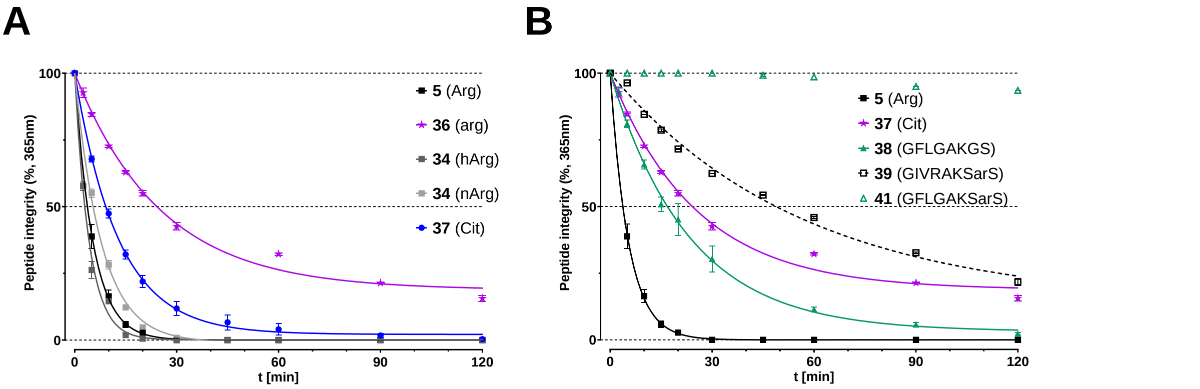

Figure S6: Time course for the degradation of octapeptidic cathepsin B substrates in human serum. (**A**) Attempts for stabilization by replacing the Arg residue in P1. (**B**) *N*-methylation of the P2'-P3' peptide bond and/or exchange of the N-terminal half by the GFLG motif. For comparison, compounds **5** and **37** were included, too.

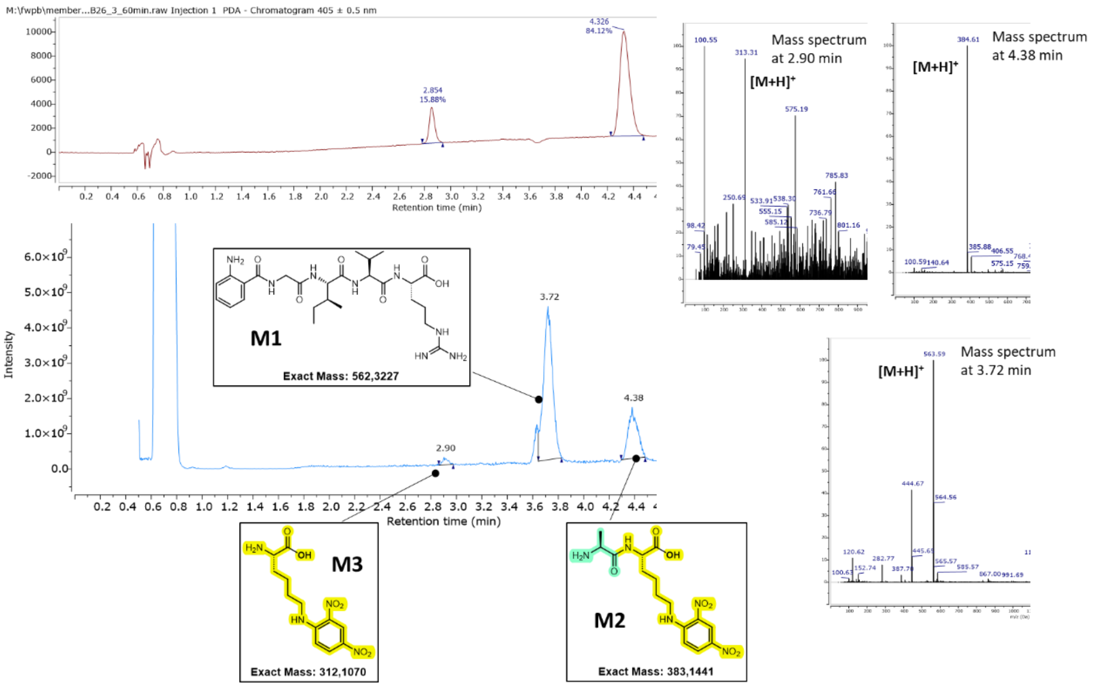

Figure S7: Assessment of stability of compound **5** (**Abz-GIVRAK(Dnp)GS-NH$_2$**) in human serum. Exemplary LC-DAD-MS analysis after incubation of **5** in human serum for 60 min. UV (405 nm) and TIC traces indicate the formation of three metabolites. No residual intact **5** is left after 60 min. The metabolites were assigned, based on the mass spectra, to compounds H-Lys(Dnp)-OH (**M3**) and H-Ala-Lys(Dnp)-OH (**M2**) and to the compound formed by cleavage after Arg leading to the aminobenzoylated tetrapeptide **M1**. As a common fragment of compounds bearing Abz, -119 amu ((6-iminocyclohexa-2,4-dien-1-ylidene)methanone is cleaved off) is visible, which corresponds to the $[M+H-Abz]^+$ signal.

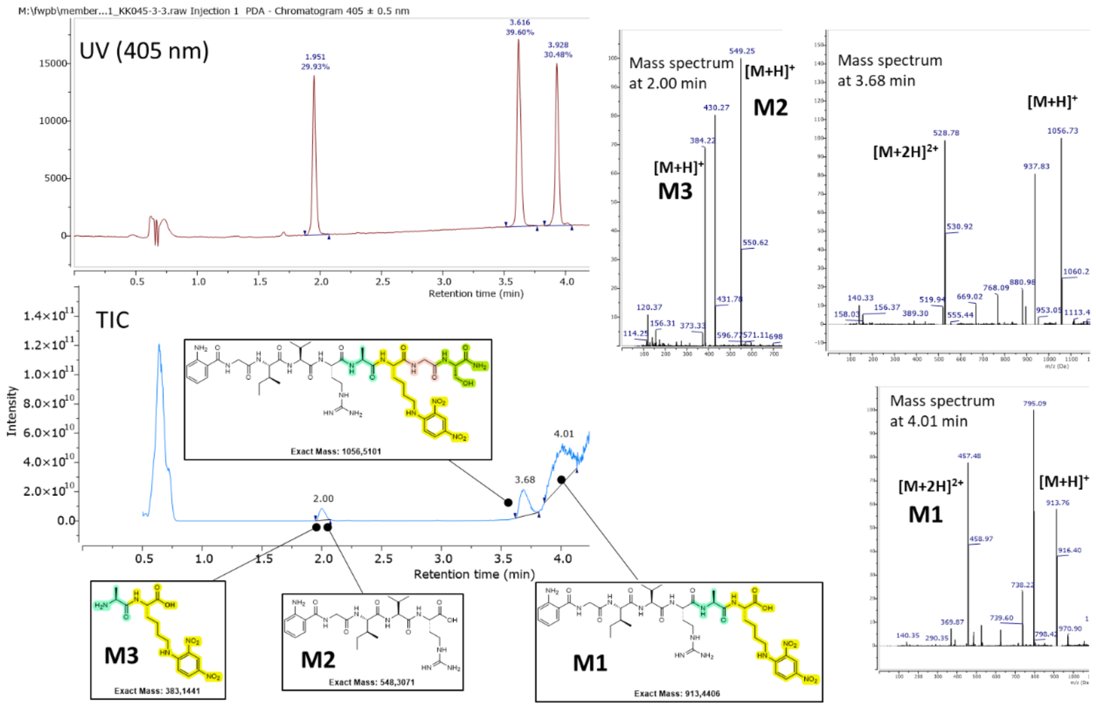

Figure S8: Assessment of stability of compound **35** (**Abz-GIV-norArg-AK(Dnp)GS-NH$_2$**) in human serum. Exemplary LC-DAD-MS analysis after incubation of **35** in human serum for 10 min. UV (405 nm) and TIC traces indicate the formation of three metabolites in addition to residual intact **35**, which were assigned, based on the mass spectra, to compounds formed by cleavage of the C-terminal glycine-serine dipeptide (**M1**), cleavage between nor-arginine and alanine (**M2**), or cleavage at both aforementioned sites (**M3**) of **35**. Of note, **M2** and **M3** elute at the same retention time. As a common fragment of compounds bearing Abz, -119 amu ((6-iminocyclohexa-2,4-dien-1-ylidene)methanone is cleaved off) is visible, which corresponds to the [M+H-Abz]$^+$ signal.

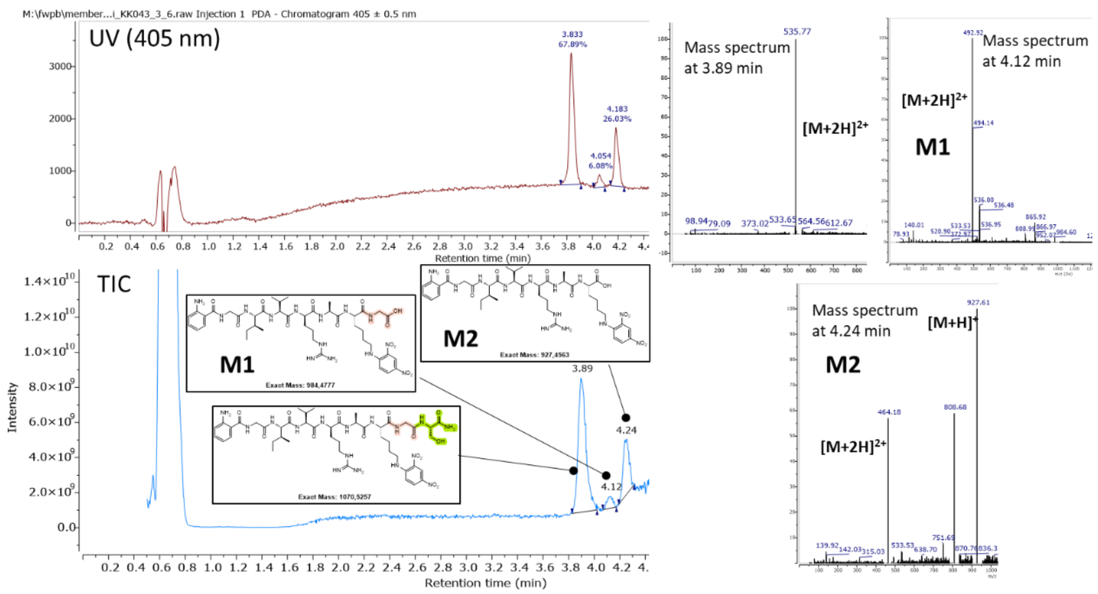

Figure S9: Assessment of stability of compound **36** (**Abz-GIVrAK(Dnp)GS-NH$_2$**) in human serum. Exemplary LC-DAD-MS analysis after incubation of X in human serum for 30 min. UV (405 nm) and TIC traces indicate the formation of two metabolites in addition to residual intact **36**, which were assigned, based on the mass spectra, to compounds formed by cleavage of the C-terminal serine (**M1**) or glycine-serine dipeptide (**M2**) from **36**. As a common fragment of compounds bearing Abz, -119 amu ((6-iminocyclohexa-2,4-dien-1-ylidene)methanone is cleaved off) is visible, which corresponds to the [M+H-Abz]$^+$ signal. Due to the D-configured arginine residue, cleavage between arginine and alanine was not detectable (compared to compound **5**, see **Figure S7**).

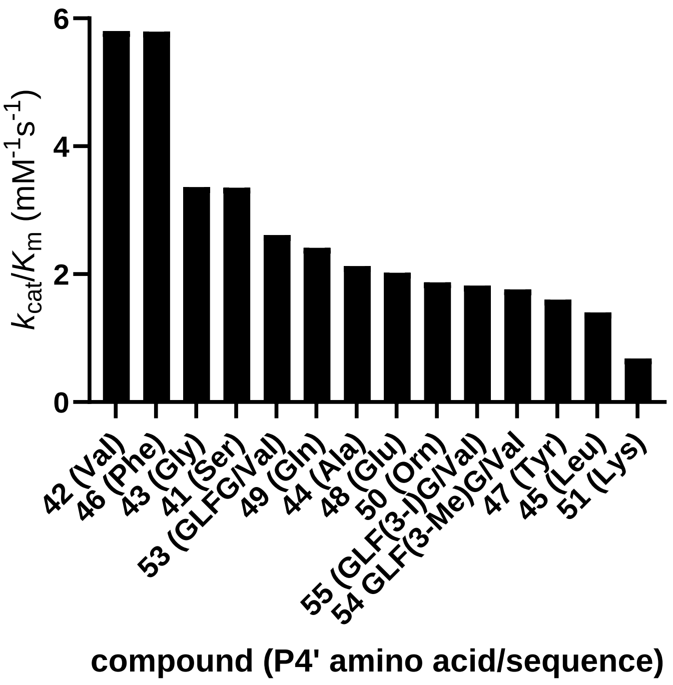

Figure S10: Influence of variation of P4' residue in the octapeptide sequence Abz-GFLG-AK(Dnp)(NMe)G-X-NH$_2$. Data points were arranged in the order of declining kinetic efficiency.

Synthesis of the carbamoylated arginine building block Fmoc-Arg(ec,Boc)-OH (78)

For the synthesis of arginine building block **78**, amine **75** [1, 2] was treated with the guanidinylating reagent **76** [3] in the presence of mercury(II) chloride to give arginine derivative **77** (Scheme S1). Subsequently, the benzyl group in **77** was removed by hydrogenolysis to afforded compound **78** that can be incorporated into peptides by SPPS.

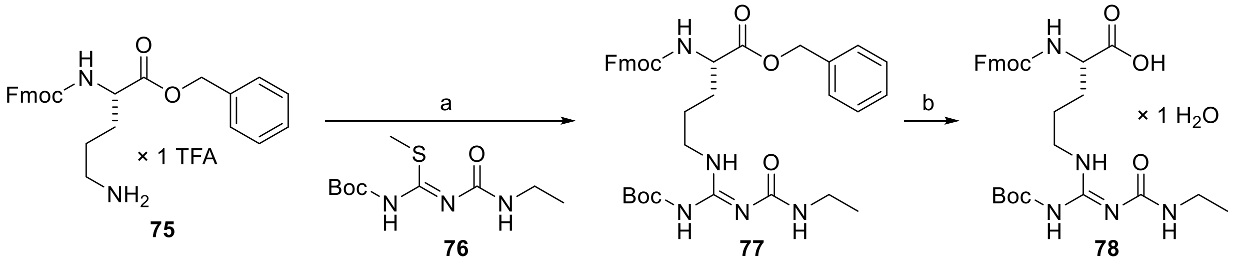

Scheme S1. Synthesis of the Fmoc-Arg(ec)-OH (**78**). Reagents and conditions: a) $HgCl_2$, DIPEA, CH_2Cl_2, rt, 1.5 h, 83%; c) H_2, 10% Pd/C, 2-propanol, 35 °C, 4 h, 66%.

General materials and synthesis for solution phase synthesis

Chemicals. Mercury(II) chloride, *N*,*N*-diisoproyplethylamine (DIPEA), the palladium-on-charcoal catalyst (10% palladium) and dichloromethane (CH_2Cl_2) were of analytical grade, purchased from commercial suppliers. Gradient grade 2-propanol was from Carl Roth (Karlsruhe, Germany) and gradient grade acetonitrile for HPLC was obtained from Sigma-Aldrich (Taufkirchen, Germany). Deuterated DMSO (DMSO-d_6) was purchased from Deutero (Kastellaun, Germany). Compound **75** was prepared according to a reported procedure [2]. The synthesis of **76** was reported elsewhere [4].

Thin layer and column chromatography (TLC). TLC was performed on Merck silica gel 60 F_{254} TLC aluminium plates. Spots were detected by irradiation with UV light (254 nm). Silica Gel 60 (63–200 µm, Merck) was used for column chromatography.

Melting point determination. Melting points were determined with a Büchi 510 apparatus (Büchi, Essen, Germany) and are uncorrected.

Elemental analysis. Elemental analysis was performed with a Vario MICRO Cube elemental analyzer (Elementar Analysensysteme, Hanau, Germany). At least two determinations were carried out per sample, and average values were calculated. Data fulfilled the requirement of less than ±0.4% deviation.

Mass spectrometry. High resolution mass spectrometry (HRMS) analysis was performed on an Agilent 6540 UHD Accurate-Mass Q-TOF LC/MS system (Agilent Technologies, Santa Clara, CA) using an ESI source.

NMR spectroscopy. NMR spectra were recorded on a Bruker Avance 400 instrument (^{1}H: 400 MHz, ^{13}C: 101 MHz) or a Bruker Avance 600 instrument with cryogenic probe (^{1}H: 600 MHz, ^{13}C: 151 MHz) (Bruker, Karlsruhe, Germany) at 300 K. The spectra were calibrated based on the solvent residual peaks (^{1}H-NMR: DMSO-d_6: δ = 2.50 ppm; ^{13}C-NMR: DMSO-d_6: δ = 39.50 ppm) and are reported as follows: ^{1}H-NMR: chemical shift δ in ppm (multiplicity (s = singlet, d = doublet, t = triplet, q = quartet, m = multiplet, br s = broad singlet), integral, coupling constant *J* in Hz); ^{13}C-NMR: chemical shift δ in ppm. To note: in the NMR spectra of the arginine derivatives **77** and **78** two major rotamers (with respect to the

orientation of the *tert*-butyloxycarbonyl group) with a ratio of approximately 1:1 were evident in the ^{1}H- and ^{13}C-NMR spectra.

Reversed-phase HPLC. Analytical RP-HPLC was performed with a system from Agilent Technologies composed of a 1290 Infinity binary pump equipped with a degasser, a 1290 Infinity Autosampler, a 1290 Infinity Thermostated Column Compartment, a 1260 Infinity Diode Array Detector and a 1260 Infinity Fluorescence Detector. A Kinetex XB-C18, 2.5 µm, 100 × 3 mm (Phenomenex) served as stationary phase at a flow rate of 0.6 mL/min. Mixtures of 0.04% aq. TFA (A) and acetonitrile (B) were used as mobile phase. The following linear gradient was applied: 0–16 min: A/B 80:20–5:95, 16–20 min: 5:95. The oven temperature was 25 °C, the injection volume was 20 µL and detection was performed at 220 nm. The retention (capacity) factor was calculated from the retention time (t_R) according to $k = (t_R - t_0)/t_0$ (t_0 = dead time).

N^{ω}-*tert*-Butoxycarbonyl-$N^{\omega'}$-(ethylaminocarbonyl)-N^{α}-((fluoren-9-yl)methoxy)carbonyl-L-arginine benzyl ester (77)

$HgCl_2$ (1.26 g, 4.65 mmol) and DIPEA (1.9 mL, 10.9 mmol) were added to a stirred solution of **75** (1.73 g, 3.10 mmol) and **76** (0.850 g, 3.25 mmol) in anhydrous CH_2Cl_2 (30 mL), and stirring was continued at rt for 1.5 h. Solid material was removed by centrifugation and the supernatant was decanted. The residue was resuspended in CH_2Cl_2 (15 mL) followed by centrifugation and decantation of the supernatant. This washing step was repeated twice. The supernatants were filtered, and the combined filtrates were concentrated under reduced pressure. CH_2Cl_2 (15 mL) was added and the volatiles were evaporated. The oily residue was dried *in vacuo* and subjected to column chromatography (CH_2Cl_2/ethyl acetate 100:1 to 2:1). The solvent of the eluate was removed under reduced pressure, and subsequent drying *in vacuo* yielded product **77** as a white solid (1.70 g, 83%). Mp 102-104 °C. TLC (light petroleum/ethyl acetate 1:1 v/v): R_f = 0.8. ^{1}H-NMR (400 MHz, DMSO-d_6, two major rotamers (ca. 1:1) were evident): δ 0.95-1.08 (m, 3H), 1.40 (s, 4.5H), 1.44 (s, 4.5H), 1.48−1.59 (m, 2H), 1.59-1.69 (m, 2H), 1.69−1.81 (m, 1H), 2.96-3.04 (m, 1H), 3.04-3.10 (m, 1H), 3.19-3.29 (m, 2H), 4.07−4.19 (m, 1H), 4.19-4.26 (m, 1H), 4.26-4.37 (m, 2H), 5.07-5.17 (m, 2H), 6.90 (t, *J*=5.4 Hz, 0.5H), 7.24-7.36 (m, 6.5 H), 7.41 (t, *J*=7.4 Hz, 2H), 7.71 (d, *J*=7.4 Hz, 2H), 7.81-7.92 (m, 3H), 8.15 (br s, 0.5H), 9.16 (br s, 0.5H), 11.08 (s, 0.5H), 12.45-12.74 (m, 0.5H). ^{13}C-NMR (100 MHz, DMSO-d_6, two major rotamers (ca. 1:1) were evident): δ 14.6, 15.0, 25.3, 25.4, 27.7, 27.98, 28.02, 28.1, 33.9, 39.4, 46.6, 53.7, 53.8, 65.7, 65.9, 81.9, 120.1, 125.2, 127.0, 127.6, 127.7, 127.96, 127.98, 128.4, 135.9, 140.7, 143.7, 143.8, 152.4, 153.0, 154.4, 156.13, 156.18, 156.5, 162.6, 164.1, 172.1, 172.2. HRMS (ESI): *m/z* $[M+H]^+$ calcd. for $[C_{36}H_{44}N_5O_7]^+$ 658.3235, found 658.3251. $C_{36}H_{43}N_5O_7$ (657.77).

N^{ω}-*tert*-Butoxycarbonyl-$N^{\omega'}$-(ethylaminocarbonyl)-N^{α}- ((fluoren-9-yl)methoxy)carbonyl-L-arginine hydrate (78)

In a two-necked 100-mL round-bottom flask, under an atmosphere of argon, a 10% Pd/C catalyst (350 mg) was added to a solution of **77** (1.68 g, 2.56 mmol) and acetic acid (7 µL) in 2-propanol (25 mL), and a slow stream of hydrogen was passed through a glass tube into the vigorously stirred suspension for 4.5 h (additional catalyst was added after 1 h (250 mg) and 3 h (150 mg)). The catalyst was removed by centrifugation and the supernatant decanted. The residue was resuspended in 2-propanol (8 mL) followed by centrifugation and decantation of the supernatant. This washing step was repeated twice. The combined supernatants were filtered using a 25 mm syringe filter (Nylon, 0.22 µm, polypropylene housing). Water (600 mL) was added to the filtrate, and the mixture was lyophilized. The crude product was subjected to column chromatography (acetonitrile/EtOH 40:1 to 20:1; upon appearance of the

product in the eluate, the eluent was changed to acetonitrile/EtOH/AcOH 1000:125:1). After completion of the elution of the product, the eluate was immediately diluted with water (twice the amount of organic solvent), immediately followed by lyophilization affording **78** as a white solid (961 mg, 64%). Mp 130-132 °C. TLC (acetonitrile/EtOH/AcOH 100:10:1 v/v/v): R_f = 0.7. ^{1}H-NMR (600 MHz, DMSO-d$_6$, two major rotamers (ca. 1:1) were evident): δ 0.94-1.09 (m, 3H), 1.41 (s, 4.5H), 1.46 (s, 4.5 H), 1.49–1.66 (m, 3H), 1.66–1.79 (m, 1H), 2.93-3.04 (m, 1H), 3.04-3.10 (m, 1H), 3.19-3.31 (m, 2H), 3.90-4.04 (m, 1H), 4.14–4.37 (m, 3H), 6.77-7.04 (br s, 0.5H), 7.27-7.37 (m, 2H), 7.37-7.47 (m, 2H), 7.60-7.76 (m, 3H), 7.79-8.00 (m, 2.5H), 8.14 (br s, 0.5H), 9.18 (s, 0.5H), 11.07 (s, 0.5H), 12.31-12.85 (m, 1.5H). ^{13}C-NMR (150 MHz, DMSO-d$_6$, two major rotamers (ca. 1:1) were evident): δ 14.6, 15.0, 15.7, 25.3, 25.6, 33.96, 34.01, 39.9, 46.7, 53.6, 65.6, 82.0, 120.1, 125.3, 127.1, 127.6, 140.7, 143.8, 143.9, 152.4, 153.8, 154.4, 156.1, 156.2, 156.4, 162.5, 164.1, 173.7, 173.8. IR (KBr): 3333, 3066, 2978, 2935, 2875, 1717, 1638, 1601, 1513, 1451, 1413, 1370, 1233, 1149 cm^{-1}. HRMS (ESI): m/z $[M+H]^+$ calcd for $[C_{29}H_{38}N_5O_7]^+$ 568.2766, found 568.2774. Anal. Calcd. for $C_{29}H_{37}N_5O_7 \cdot H_2O$: C 59.43, H 6.71, N 11.96; found: C 59.65, H 6.47, N 11.94. RP-HPLC (220 nm): >99% (t_R = 7.2 min, k = 8.0; Figure S11). $C_{29}H_{37}N_5O_7 \cdot H_2O$ (567.64 + 18.02).

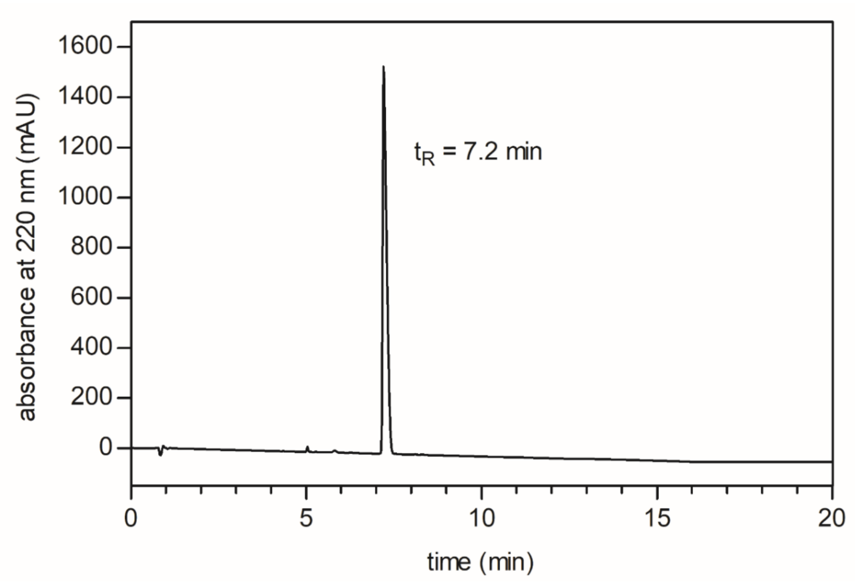

Figure S11: Chromatogram of the RP-HPLC analysis of compound **78**

Cell lines and antibodies for cathepsin expression analysis

Table S2: Cell lines used for cathepsin expression analysis

Cell line	Origin	Source	Order number	Culture medium
**U87-MG**	human, glioblastoma	University hospital Dresden, Clinic and polyclinic for Neurosurgery (Kirsch group, Dr. O. Uckermann)	ATCC® HTB-14™	DMEM<br>10% FKS
**U251-MG**	human, glioblastoma (astrocytoma)	ECACC (Sigma Aldrich)	09063001	DMEM<br>10% FKS
**A431**	human, epidermoid carcinoma	University hospital Dresden, Clinic and polyclinic for Radiation Therapy and Radio-Oncology (Mrs. Pfitzmann)	ATCC® CRL-1555™	DMEM<br>10% FKS
**Mel-Juso**	human, melanoma	DSMZ	ACC-74	DMEM<br>10% FKS
**SW403**	human colorectal adenocarcinoma (Dukes' type C, grade III, female)	DSMZ	ACC 294	DMEM<br>10% FKS<br>1% P/S
**SW480**	human colorectal adenocarcinoma (Dukes' type B, male)	DSMZ	ACC 313	RPMI<br>10% FKS<br>1% P/S
**SW620**	human colorectal adenocarcinoma (Dukes' type C, male); from lymph node metastasis	ATCC	CCL227	DMEM<br>10% FKS<br>1% P/S

Table S3: Antibodies used for cathepsin expression analysis

[a] in 0.05% Tween 20 (v/v) in TBS, PM: powdered milk

Antibody	Manufacturer/Distributor	Order number	Dilution	Buffer[a]
**anti-Cathepsin B**	ABCAM	ab#92955	1:500	2% BSA
**anti-Cathepsin K**	ABCAM	ab#207086	1:5000	5% PM
**anti-Cathepsin L**	ABCAM	#ab6314	1:2000	1% BSA
**anti-Cathepsin S**	ABCAM	#ab134157	1:5000	5% BSA
**anti-Cystatin B**	SANTA CRUZ BIOTECHNOLOGIES	sc-33274	1:600	2% BSA
**anti-Cystatin C**	ABCAM	ab#109508	1:1000	5% PM
**anti-β-Actin mouse monoclonal**	SIGMA-ALDRICH	A5316	1:1000	5% PM
**anti-GAPDH mouse monoclonal**	SIGMA-ALDRICH	G8795	1:5000	5% PM

Results of Western blot-based expression analysis

A

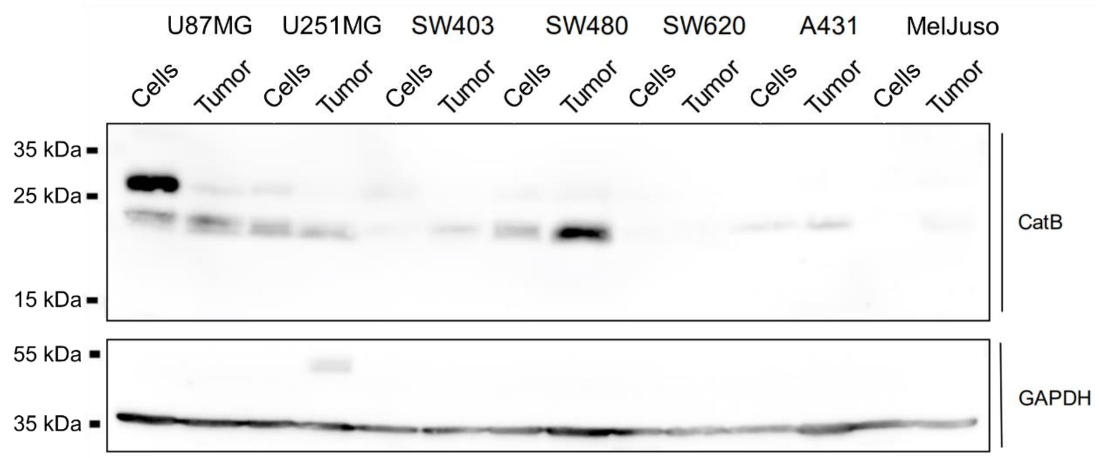

B

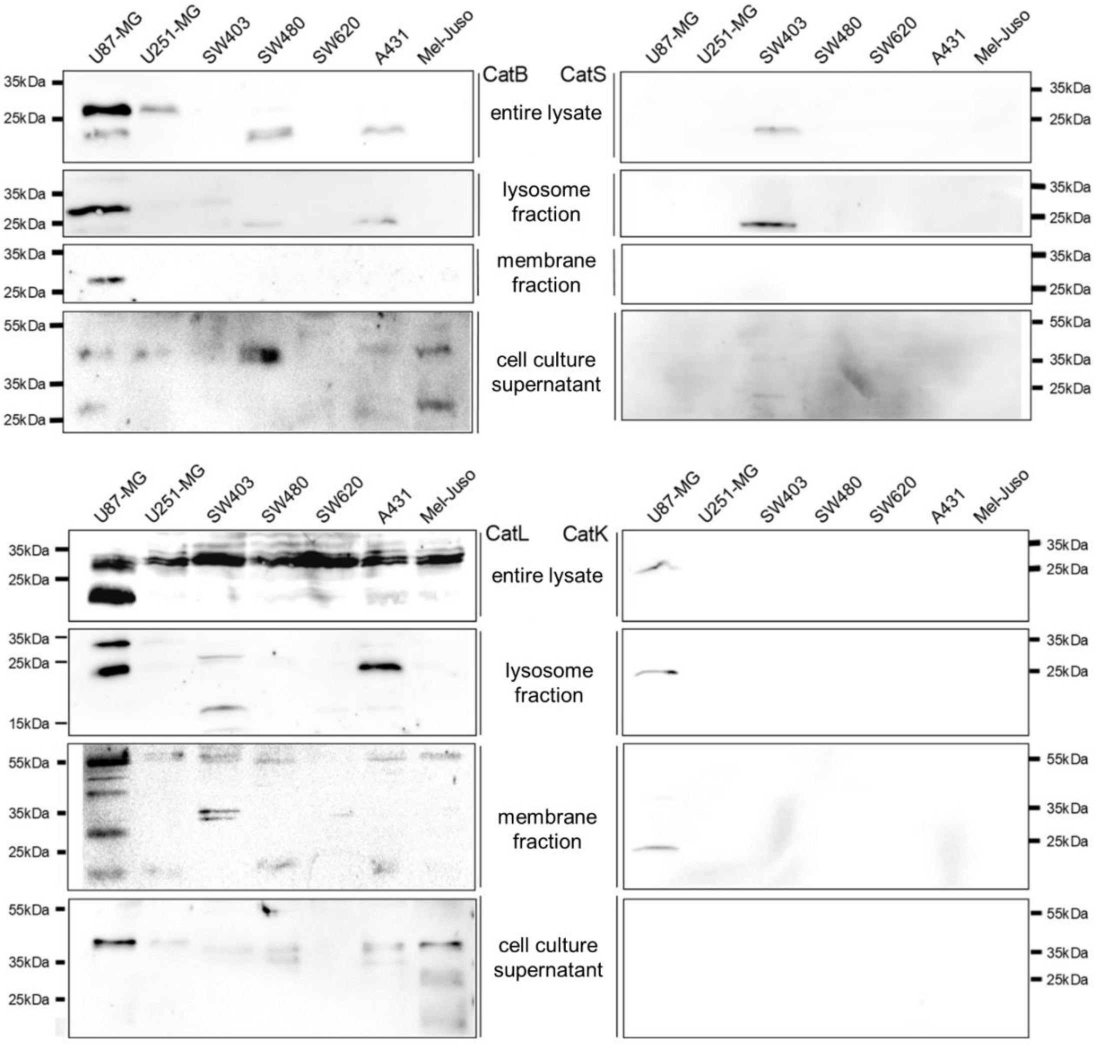

Figure S12: (**A**) Western blot analysis of the expression of the cathepsin B in 2D-cell culture (top) and in mouse model xenograft tumours (bottom) from various cell lines. Primary antibodies: mouse anti-cathepsin B (abcam, ab#92955), mouse anti-β-actin (Sigma-Aldrich, A5316). Secondary antibody: anti-mouse IgG POD (Sigma Aldrich, A9044). Blocking with either 5% (w/w) bovine serum albumin (BSA) in 0.1% Tween/TBS (v/v) or 5% (w/w) skim milk powder (SMP) in 0.1% Tween/TBS (v/v). (**B**) Western blot analysis for localising of cathepsins B, S, L and K in the lysosomal and membrane fraction and cell supernatant.

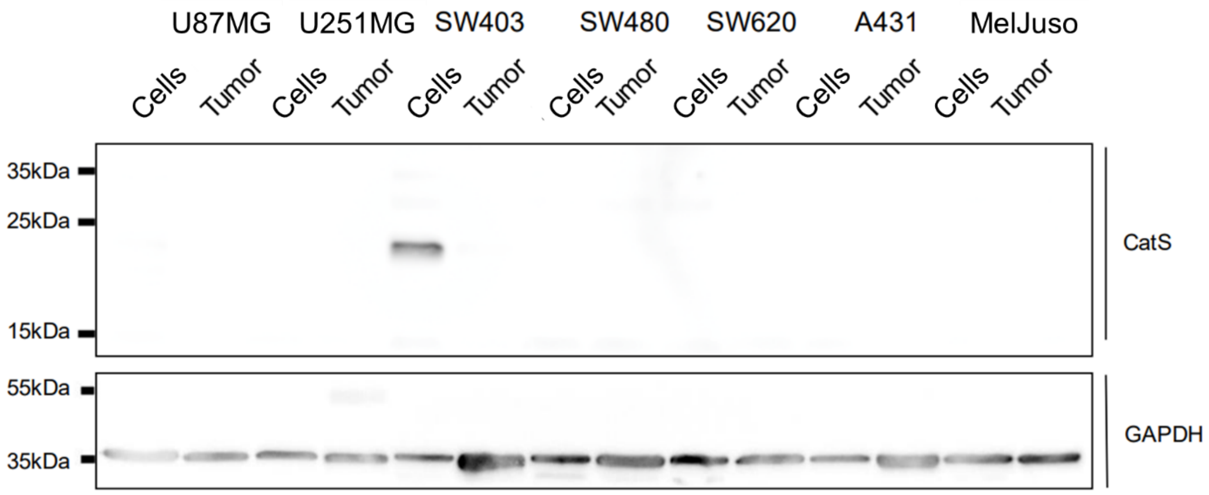

Figure S13: Expression of cathepsin S as analyzed in lysates of tumor cells and derived murine xenograft tissue.

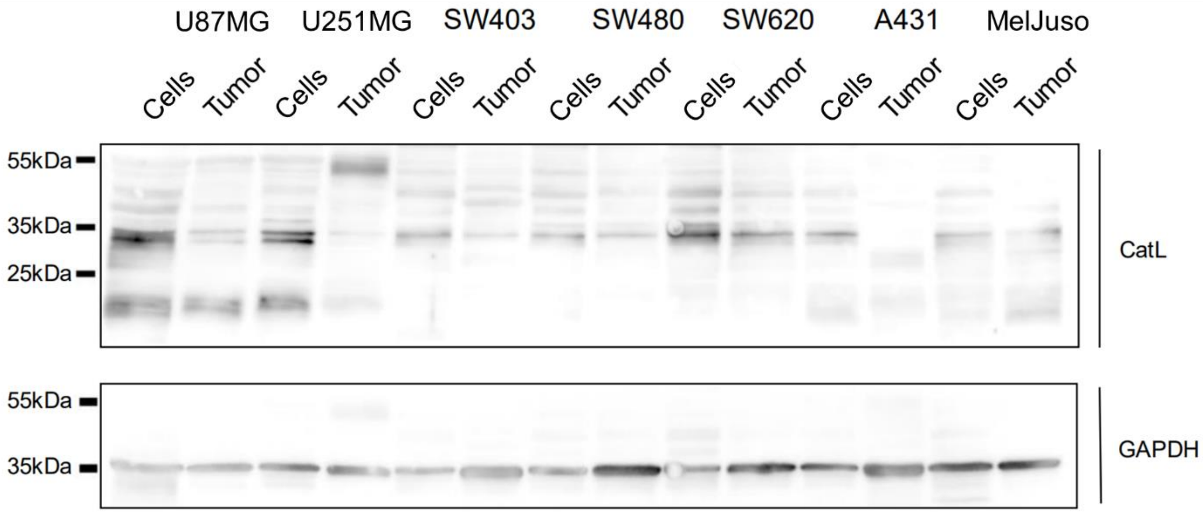

Figure S14: Expression of cathepsin L as analyzed in lysates of tumor cells and derived murine xenograft tissue.

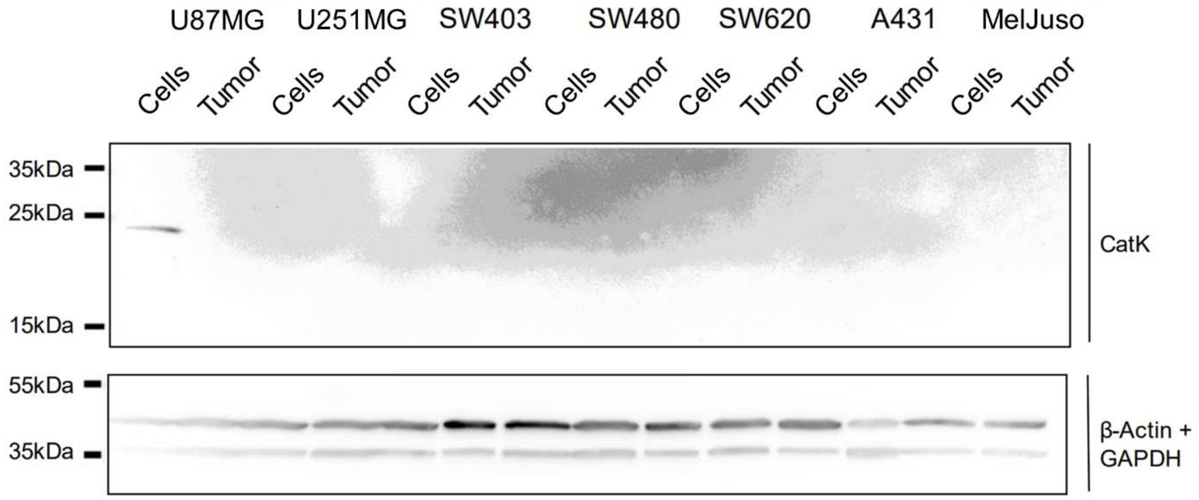

Figure S15: Expression of cathepsin K as analyzed in lysates of tumor cells and derived murine xenograft tissue.

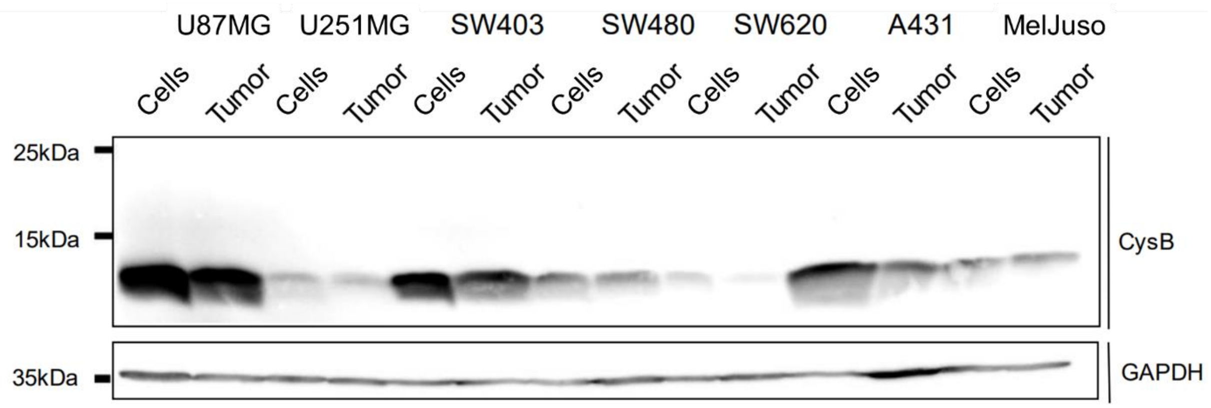

Figure S16: Expression of cystatin B as analyzed in lysates of tumor cells and derived murine xenograft tissue.

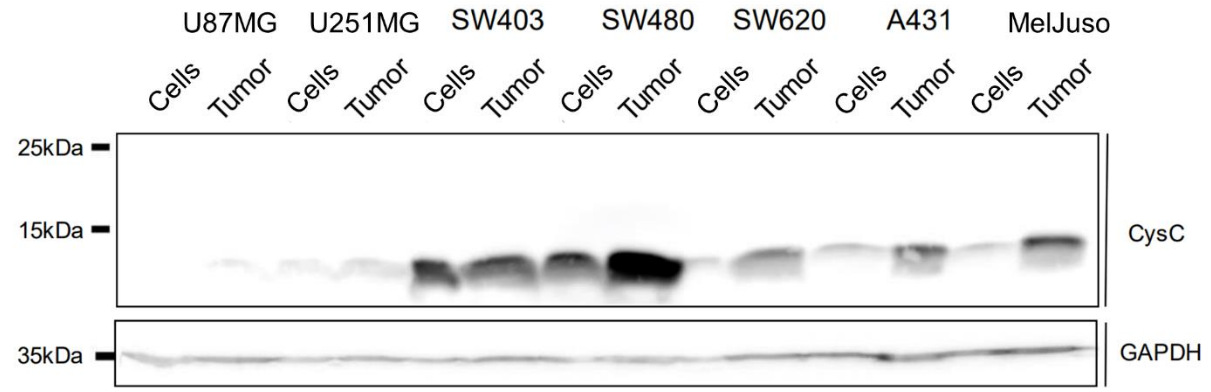

Figure S17: Expression of cystatin C as analyzed in lysates of tumor cells and derived murine xenograft tissue.

Immunohistochemical detection of cysteine cathepsins in tumor tissue sections

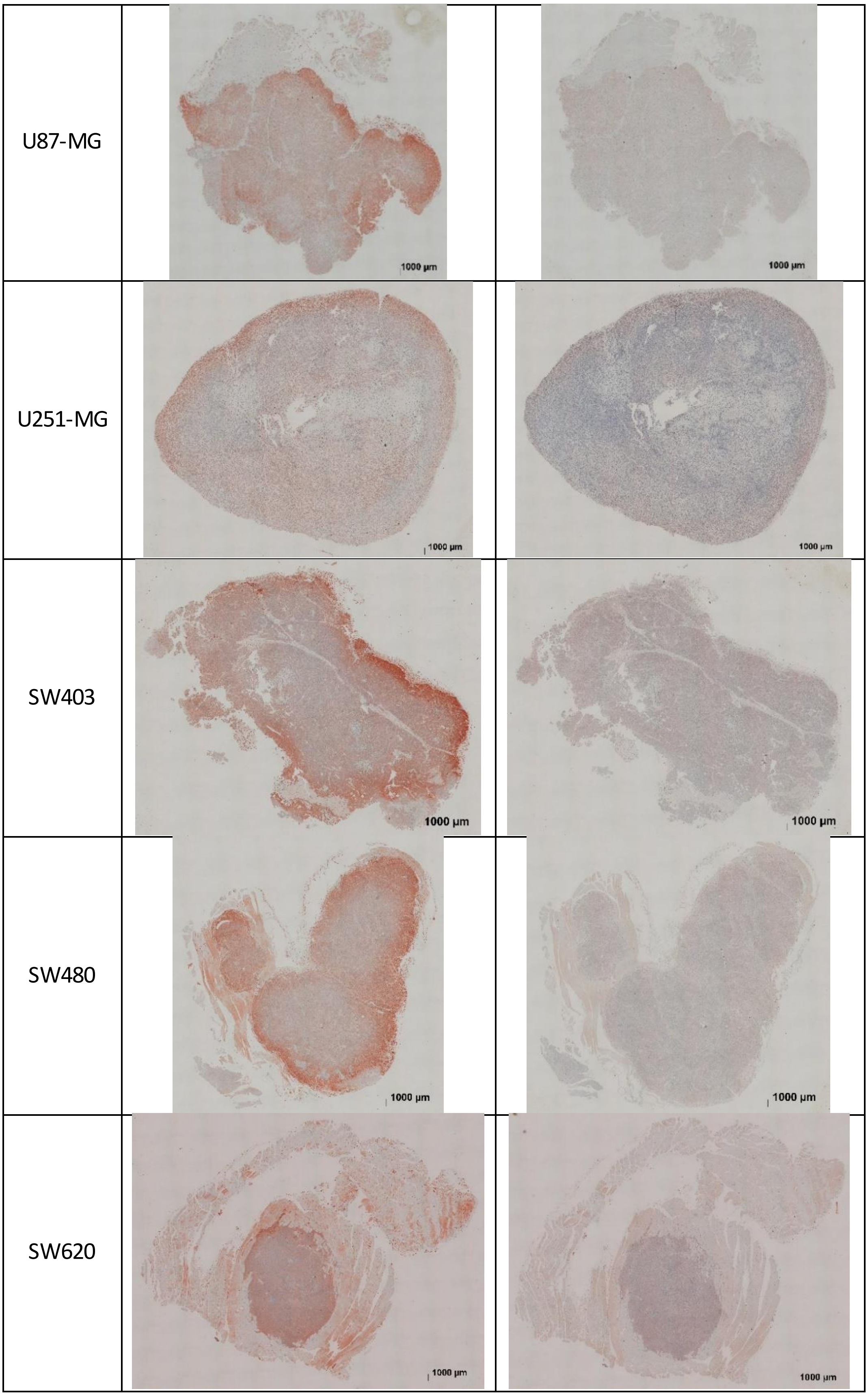

Figure S18: Immunohistochemical detection of cathepsin B in tumor xenograft tissue sections.

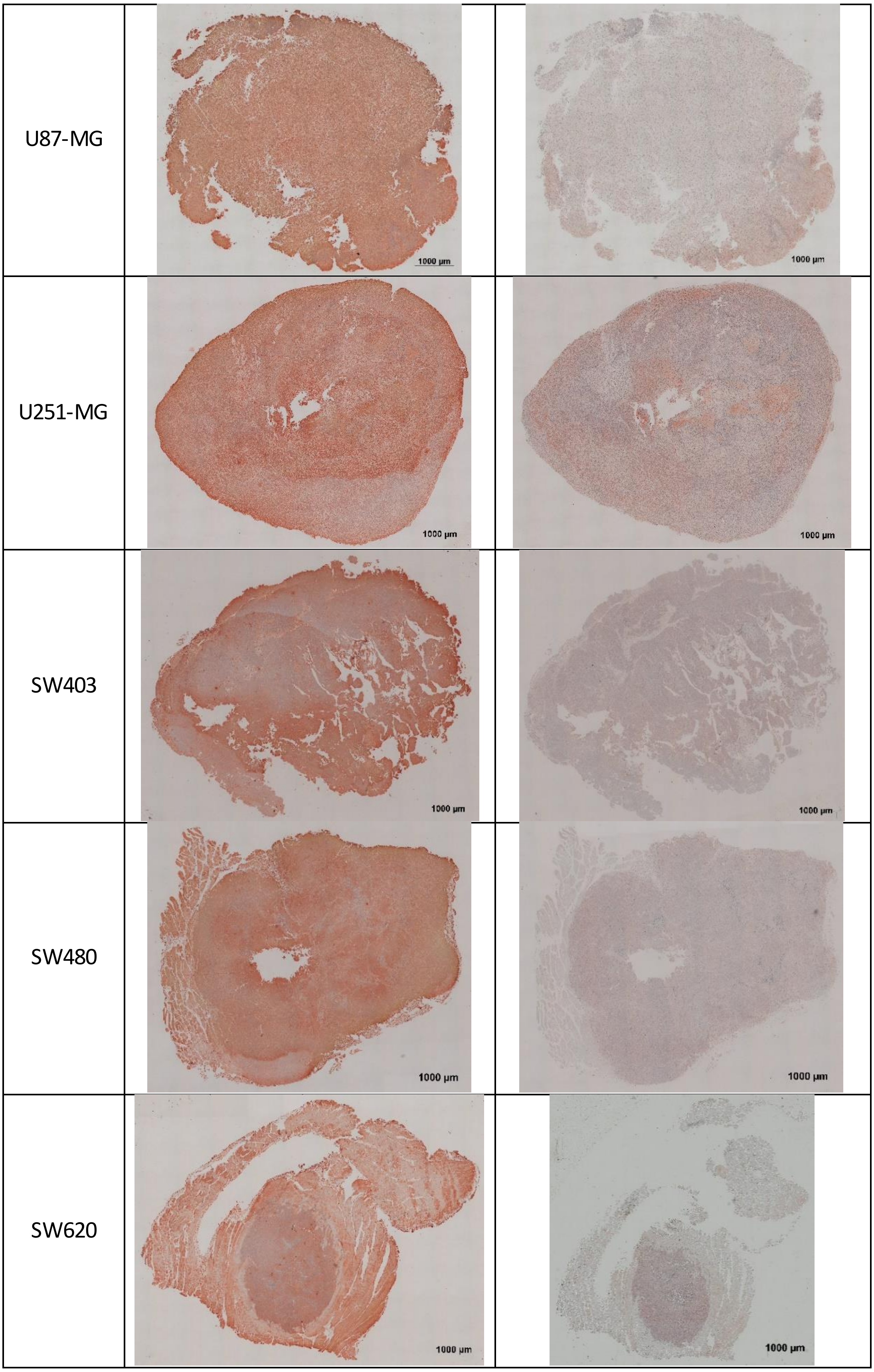

Figure S19: Immunohistochemical detection of cathepsin L in tumor xenograft tissue sections.

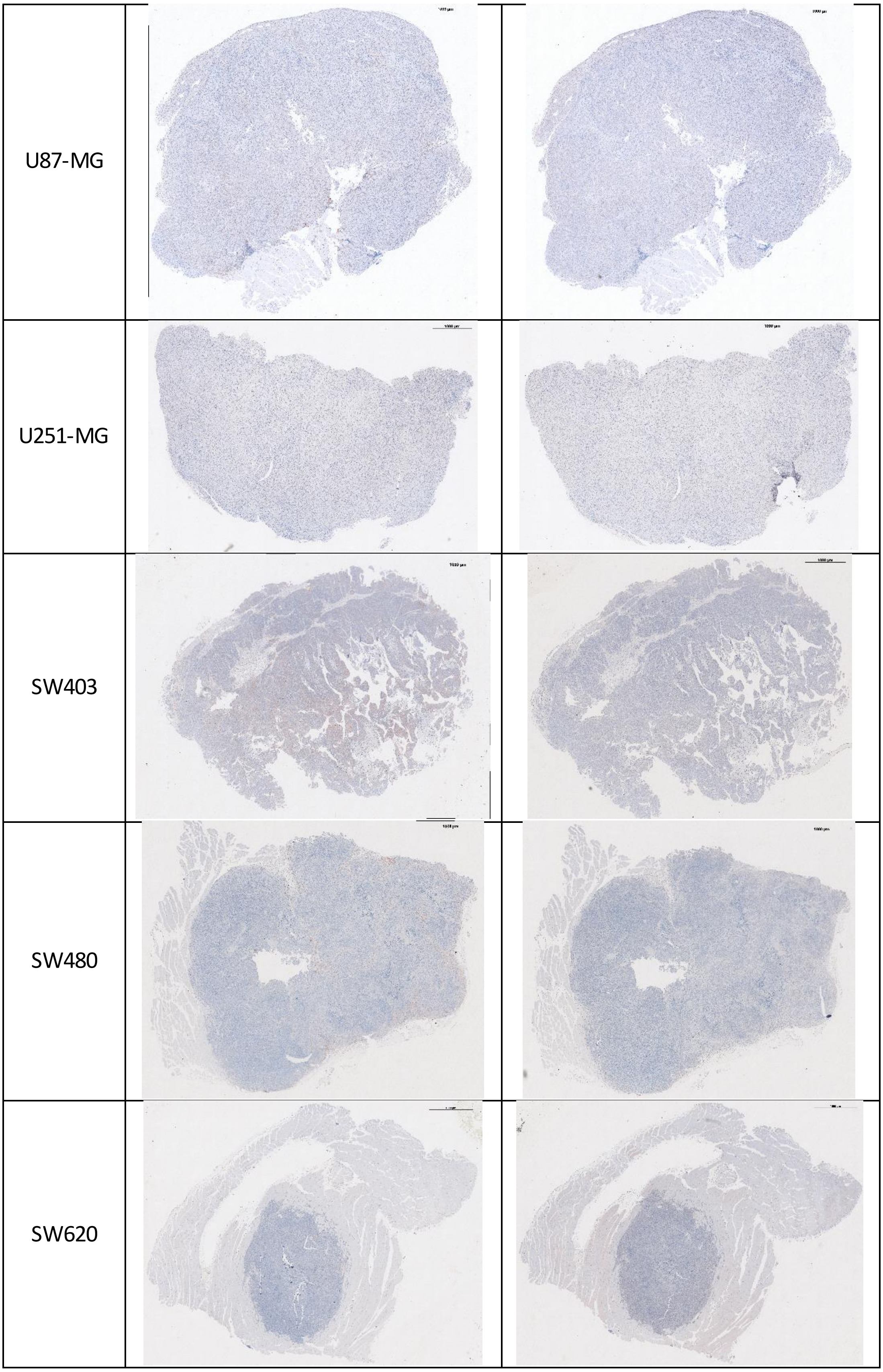

Figure S20: Immunohistochemical detection of cathepsin S in tumor xenograft tissue sections.

Proof of specific uptake of TAMRA-ACPP (68)

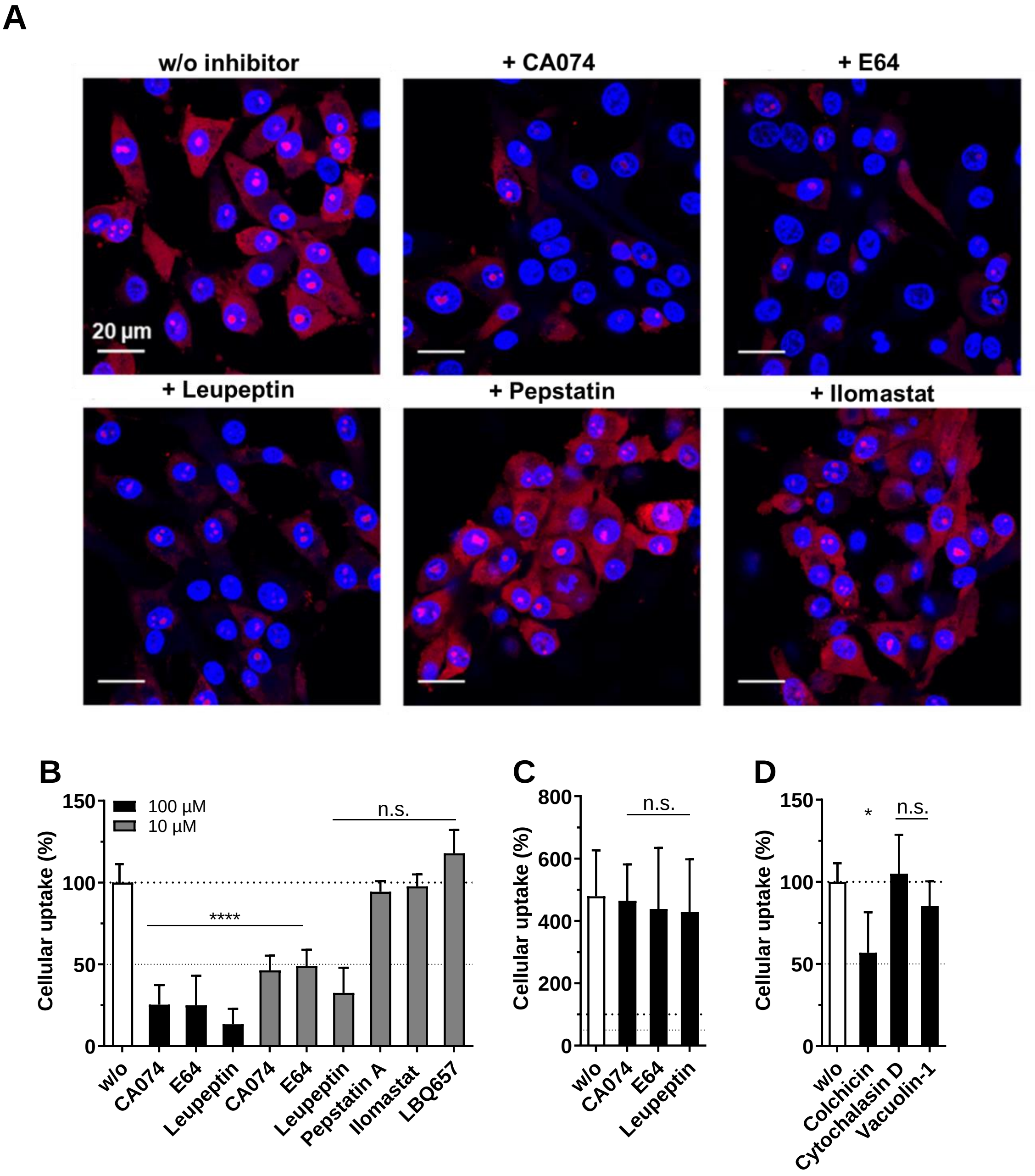

Figure S21: Proof of specificity for the uptake of TAMRA-ACPP (**68**) in human U87MG glioma cells. (**A**) Fluorescence microscopy images in the absence and presence of various protease inhibitors ([inhibitor]= 10 µM each). After pre-incubation with 10 µM inhibitor, the cells were treated with **68** (5 µM). Red: TAMRA-ACPP (**68**), blue: Nuclei stained by Hoechst 33342. n ≥ 4. (**B**) Effect of different protease inhibitors on the uptake of compound **68**. n ≥ 4. (**C**) Uptake of TAMRA-CPP (**70**) in the presence of different protease inhibitors. Inhibitor concentration: 100 µM, n ≥ 4, vehicle control was normalized to vehicle control of **68**. (**D**) Effect of inhibitors of cellular trafficking on the uptake of compound **68**. Uptake values are each normalized to the value of uptake of TAMRA-ACPP (**68**) in U87MG cells after 30 min at 37 °C in the presence of 0.5 mM DTT (100%). Statistics: One-Way ANOVA with Dunnett post-test. Comparison vs. w/o. * = $p < 0.01$, **** = $p < 0.0001$. See Table S4 for inhibitor structures and activity data.

Table S4: Structure and activities of inhibitor compounds applied in the study of the cellular uptake of TAMRA-ACCP (**68**).

**Compound**	**Structure**	**Target protein**	**Activity**	**Ref.**
E64	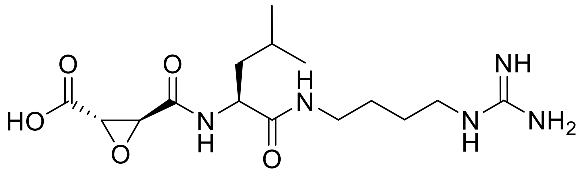	Cat B	k_{inact}/K_I=89400 $M^{-1}s^{-1}$	5
		Cat L	k_{inact}/K_I =96250 $M^{-1}s^{-1}$	
		Cat S	k_{inact}/K_I= 99000 $M^{-1}s^{-1}$	
		Cat K	IC_{50}=4 nM	6
CA074	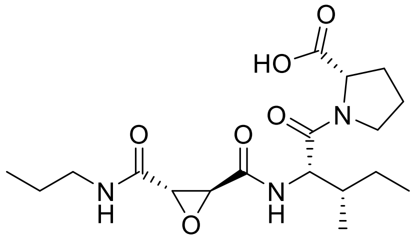	Cat B	IC_{50}=44 nM	7
		Cat L	IC_{50}>16 µM	
		Cat S	IC_{50}=4.8 µM µM	
		Cat K	IC_{50}>16 µM µM	
Leupeptin	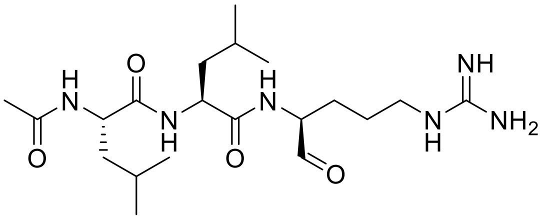	Cat B	IC_{50}=117 nM	8
		Cat L	IC_{50}=70.3 nM	
		CatK	IC_{50}=42.2 nM	9
Ilomastat	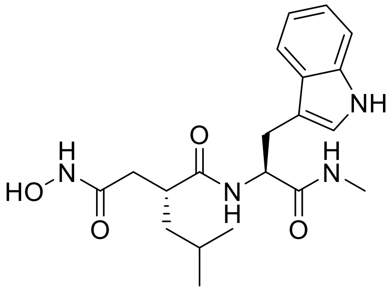	MMP-2	K_i =0.39 nM	10
		MMP-3	K_i =27 nM	
		MMP-8	K_i =0.18 nM	
		MMP-9	K_i =0.2 nM	
		MMP-12	K_i=3.8 nM	11
Pepstatin A	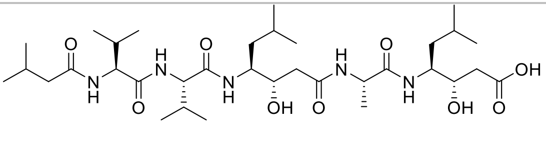	Cat D	IC_{50}=0.55 nM	12
		Cat E	IC_{50}=0.45 nM	13

LBQ657	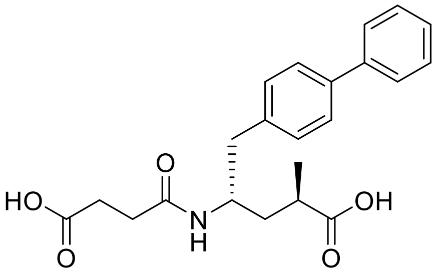	Neprilysin	IC_{50}=20 nM	[14]
Colchicine	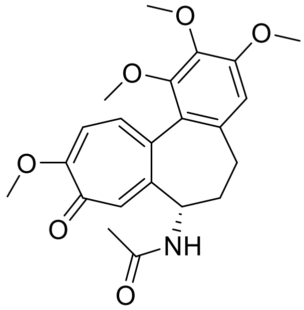	Tubulin	IC_{50}=360 nM	[15]
Cytochalasin D	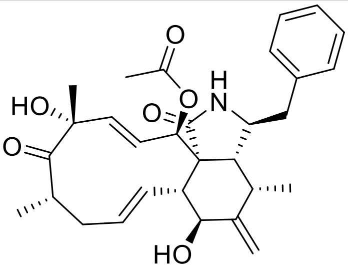	Actin	$IC_{50}\approx$500 nM	[16]
Vacuolin-1	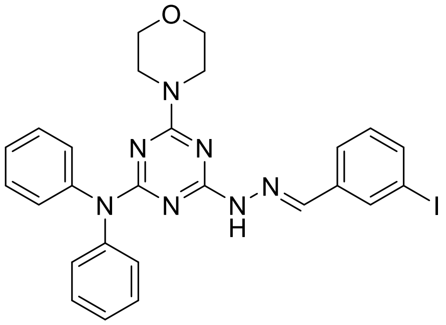	Phosphatidylinositol-3-phosphate-5 kinase (PIKfyve)	K_d= 9 nM	[17]

Supplementary material for radiopharmacological characterization of [^{64}Cu]Cu-NODAGA-ACPP ([^{64}Cu]Cu-**71**) and analogues

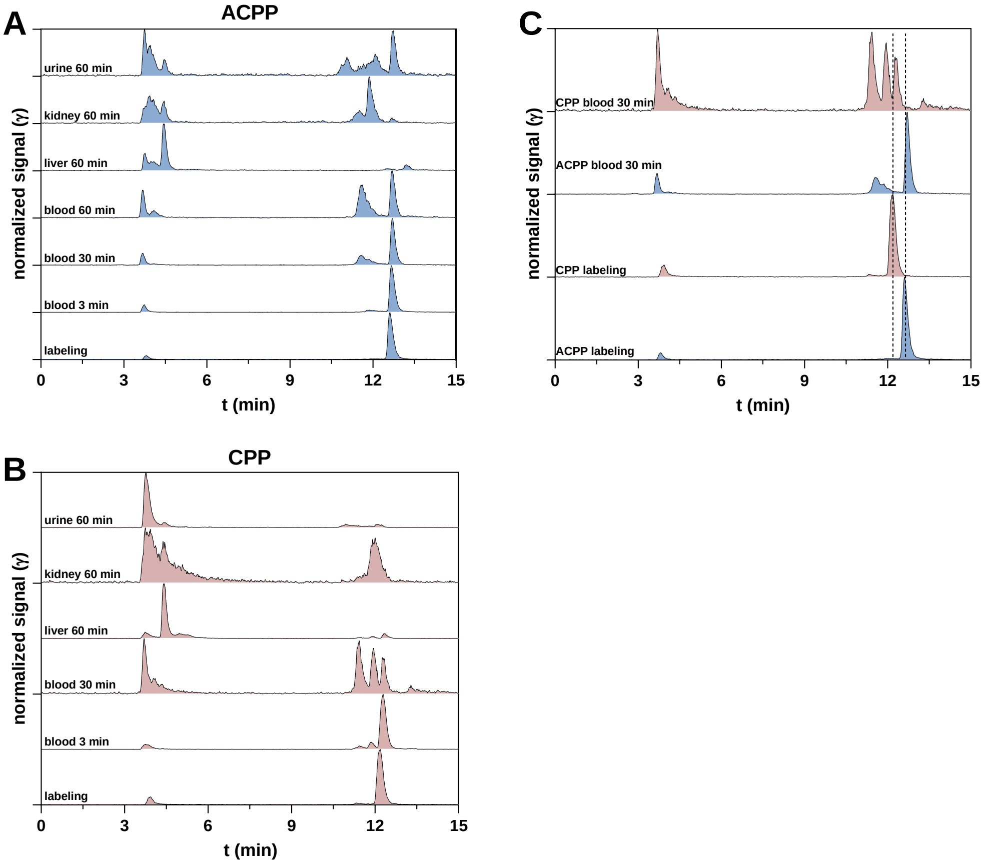

Figure S22: Metabolic stability of [^{64}Cu]Cu-NODAGA-ACPP ([^{64}Cu]Cu-**71** (**A**) and [^{64}Cu]Cu-NODAGA-CPP ([^{64}Cu]Cu-**71** (**B**) as examined in body fluids and selected organs in healthy Wistar rats by ex vivo radio-HPLC analysis. (**C**) Overlayed chromatograms for [^{64}Cu]Cu-**71** and [^{64}Cu]Cu-**73** at 30 min p.i. for comparison.

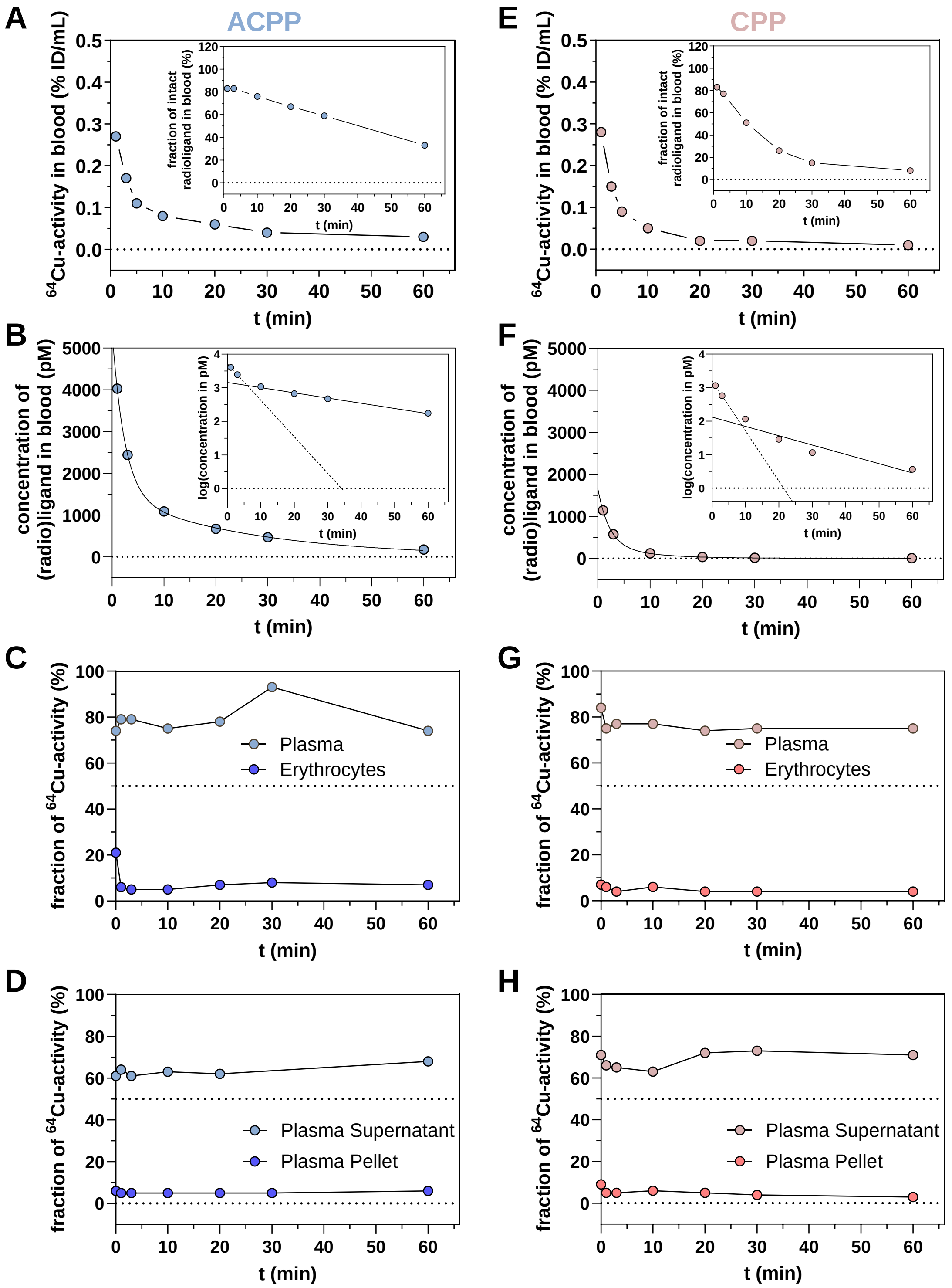

Figure S23: Blood clearance and blood component analysis for [^{64}Cu]NODAGA-ACPP and [^{64}Cu]NODAGA-CPP. (**A**)/(**E**) and (**B**)/(**F**): Time-activity curves as sampled in the blood expressed as %ID/mL ((**A**) and (**E**), respectively) and molar concentration (B and F, respectively). (**C**)/(**D**) and (**G**)/(**H**): Time course of activity distribution among blood components (**C**)/(**D**) and (**G**)/(**H**).

Table S5: Comparison of pharmacokinetic parameters and uptake in selected organs of [^{64}Cu]Cu-NODAGA-ACPP ([^{64}Cu]Cu-**71**) and [^{64}Cu]Cu-NODAGA-CPP (([^{64}Cu]Cu-**73**) in healthy Wistar rats. The corresponding diagrams are shown in **Figure S23** above.

	**[^{64}Cu]Cu-NODAGA-ACPP ([^{64}Cu]Cu-71)**	**[^{64}Cu]Cu-NODAGA-CPP ([^{64}Cu]Cu-73)**
Rat body weight (g)	481	437
Injected activity	104.8	110.2
Molar activity (MBq/nmol)	59	225
Injected amount (nmol)	1.78	0.49
RCP (%)	93	85
C_{02} (nM)	1.44	0.13
k_{Ell} (min^{-1})	0.016	0.028
V_D (mL)	1.232	3.715
V_D (mL/kg)	2561	8501
terminal $t_{1/2}$ (min)	45	25
Plasma CL (mL min^{-1})	19	103
Plasma CL (mL min^{-1} kg^{-1})	40	236
Activity in kidneys (%ID)	24.4	3.7
Activity in liver (%ID)	3.9	15.3
Activity in spleen (%ID)	1.2	3.0
Activity in lung (%ID)	2.2	0.5
Activity in urine (%ID)	1.1	3.4

Table S6: Ex vivo biodistribution of [^{64}Cu]Cu-NODAGA-ACCP ([^{64}Cu]**71**) under baseline conditions (control) and upon treartment with 10 mg/kg CA074 (blocked).

	1 h				4 h				24 h			
	**control**		**blocked**		**control**		**blocked**		**control**		**blocked**	
**SUV**	**MW**	**SD**	**MW**	**SD**	**MW**	**SD**	**MW**	**SD**	**MW**	**SD**	**MW**	**SD**
**Blood**	0.33	0.05	0.30	0.09	0.42	0.33	0.34	0.17	0.53	0.36	0.33	0.14
**BAT**	0.18	0.02	0.21	0.04	0.16	0.05	0.21	0.07	0.19	0.05	0.20	0.05
**Skin & hairs**	0.32	0.01	0.35	0.08	0.17	0.03	0.20	0.04	0.16	0.03	0.20	0.03
**Brain**	0.02	0.00	0.02	0.00	0,01	0.01	0.02	0.00	0.03	0.00	0.04	0.00
**Ovaries**	0.25	0.02	0.24	0.07	0.90	0.99	0.32	0.25	0.11	0.04	0.19	0.05
**Uterus**	0.25	0.03	0.24	0.03	0.82	0.80	0.44	0.43	0.16	0.02	0.19	0.06
**Pancreas**	0.14	0.03	0.13	0.01	2.46	2.79	0.47	0.62	0.09	0.04	0.12	0.02
**Spleen**	2.28	0.19	1.48	0.22	2.81	1.18	1.03	0.36	2.54	0.09	2.02	0.28
**Adrenal glands**	0.75	0.20	0.79	0.40	1.34	1.44	0.95	0.34	0.61	0.13	0.63	0.30
**Kidney**	34.75	3.60	29.46	1.78	22.27	4.03	29.14	3.43	7.37	0.18	10.79	2.00
**Fat**	0.08	0.03	0.22	0.17	2.62	3.18	0.41	0.32	0.07	0.03	0.48	0.83
**Muscle**	0.10	0.01	0.09	0.00	0.26	0.24	0.10	0.08	0.05	0.00	0.06	0.01
**Heart**	0.20	0.01	0.21	0.01	0.15	0.03	0.19	0.03	0.22	0.02	0.26	0.02
**Lung**	1.39	0.31	1.00	0.43	0.37	0.09	0.42	0.04	0.41	0.14	0.51	0.06
**Thyroid**	0.35	0.04	0.29	0.09	0.27	0.04	0.27	0.01	0.23	0.02	0.35	0.04
**Liver**	7.73	1.04	9.25	1.01	7.32	3.39	7.08	1.68	6.94	0.35	6.93	0.75
**Femur**	0.31	0.06	0.30	0.05	0.26	0.07	0.21	0.02	0.16	0.03	0.21	0.04
**Tumor**	0.26	0.01	0.27	0.01	0.23	0.12	0.15	0.05	0.23	0.06	0.30	0.07

Table S6 (continued)												
	**1 h**				**4 h**				**24 h**			
	**control**		**blocked**		**control**		**blocked**		**control**		**blocked**	
**SUV**	**MW**	**SD**	**MW**	**SD**	**MW**	**SD**	**MW**	**SD**	**MW**	**SD**	**MW**	**SD**
**Tu/Mu**	2.63	0.30	2.93	0.21	3.37	3.70	2.25	1.54	4.16	0.73	5.53	1.48
%ID												
**Intestine**	2.67	0.18	2.47	0.12	7.33	5.77	3.44	2.02	3.01	1.06	3.78	0.42
**Stomach**	0.47	0.01	0.41	0.11	0.94	0.75	0.60	0.30	0.27	0.07	0.43	0.09

Table S7: Ex vivo biodistribution of [^{64}Cu]Cu-NODAGA-CCP ([^{64}Cu]**73**).

	1 h		4 h		24 h	
	**control**		**control**		**control**	
**SUV**	**MW**	**SD**	**MW**	**SD**	**MW**	**SD**
**Blood**	0.25	0.25	0.63	0.69	0.18	0.05
**BAT**	0.10	0.05	0.28	0.27	0.18	0.10
**Skin & hairs**	0.19	0.05	0.16	0.05	0.14	0.02
**Brain**	0.02	0.01	0.02	0.01	0.02	0.00
**Ovaries**	0.58	0.69	0.54	0.41	0.41	0.09
**Uterus**	0.73	0.97	0.60	0.90	0.29	0.33
**Pancreas**	1.47	2.71	0.45	0.56	0.80	1.39
**Spleen**	2.15	1.22	2.78	1.95	1.33	0.24
**Adrenal glands**	2.00	0.73	3.43	1.93	1.68	0.54
**Kidney**	1.65	0.15	1.69	0.25	0.97	0.11
**Fat**	0.51	0.88	0.27	0.37	0.16	0.16
**Muscle**	0.07	0.05	0.07	0.04	0.06	0.03
**Heart**	0.22	0.03	0.34	0.12	0.25	0.06
**Lung**	0.84	0.45	1.89	1.45	0.75	0.18
**Thyroid**	0.25	0.08	0.22	0.10	0.22	0.06
**Liver**	10.46	5.58	15.52	6.29	11.85	2.11
**Femur**	0.27	0.07	0.40	0.13	0.38	0.12
**Tumor**	0.16	0.06	0.13	0.04	0.17	0.03
**Tu/Mu**	2.67	1.10	2.06	0.58	3.00	1.01
%ID						
**Intestine**	6.52	8.73	6.37	7.38	4.74	4.23
**Stomach**	1.11	1.23	0.68	0.57	0.57	0.63

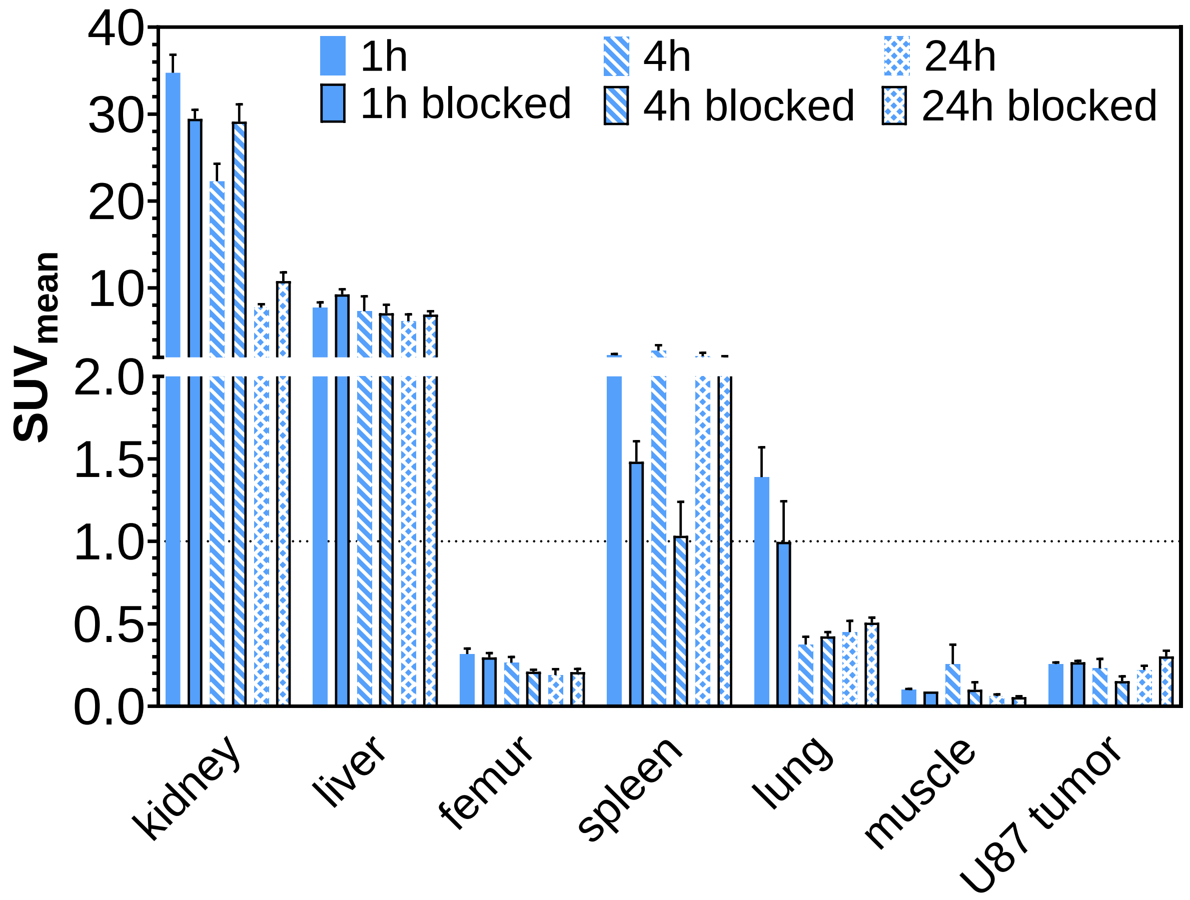

Figure S24: Ex-vivo biodistribution of [^{64}Cu]Cu-NODAGA-ACPP ([^{64}Cu]Cu-**71**) for selected organs in U87MG-tumor-bearing mice in the absence and presence of treatment with CA074 (10 mg/kg) at various time point p.i. (see legend). Uptake vales (SUV) for all organs are included in Table S6 above. Data points in the absence of CA074 are identical to those shown in Figure 11A in the main text.

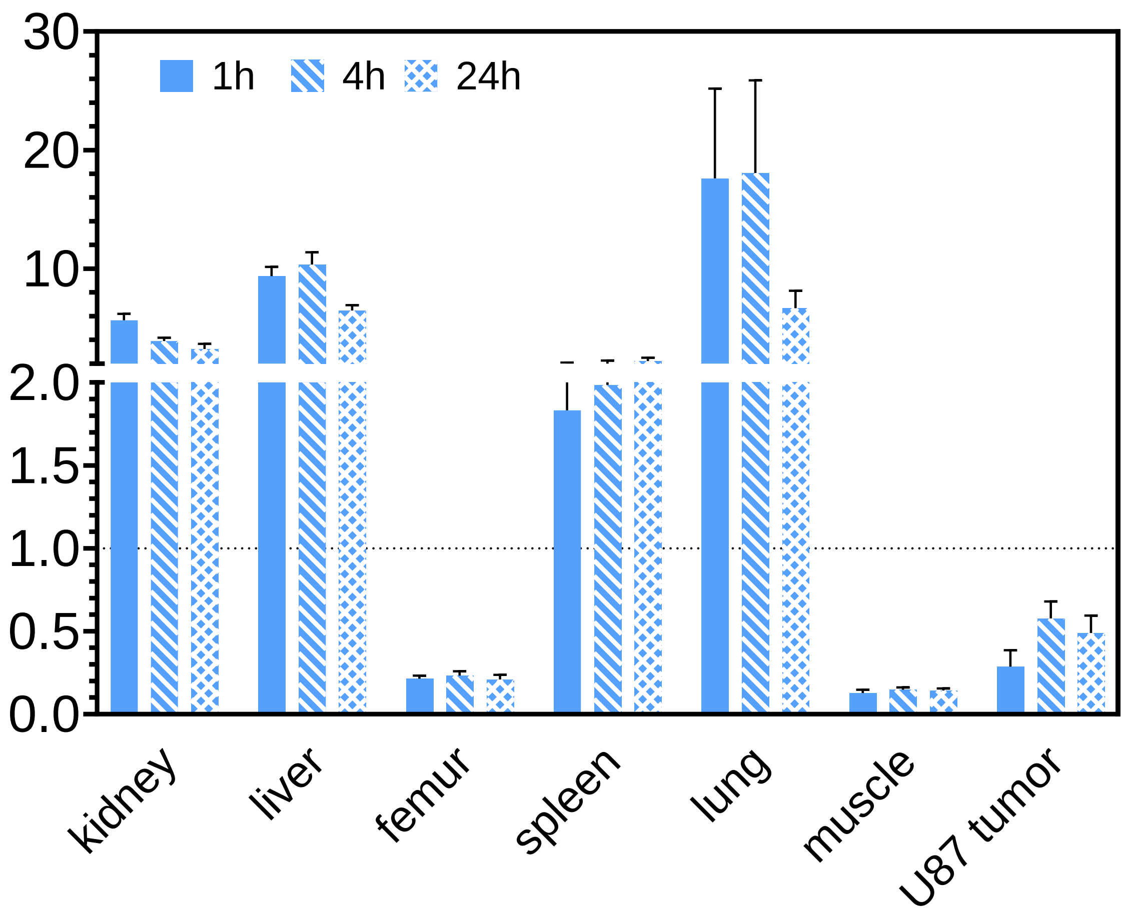

Figure S25: Ex-vivo biodistribution of [^{64}Cu]Cu-NODAGA-nACPP(ala)$_8$ ([^{64}Cu]Cu-**72**) for selected organs in U87MG-tumor-bearing mice at various time points p.i. (see legend). Uptake vales (SUV) for all organs are included in Table S8 below.

Table S8: Ex vivo biodistribution of [^{64}Cu]Cu-NODAGA-nACPP(ala$_8$) ([^{64}Cu]**72**).

	1 h		4 h		24 h	
	**control**		**control**		**control**	
SUV	**MW**	**SD**	**MW**	**SD**	**MW**	**SD**
**Blood**	0.42	0.15	0.56	0.10	0.79	0.06
**BAT**	0.51	0.32	0.37	0.11	0.68	0.31
**Skin & hairs**	0.44	0.04	0.42	0.10	0.35	0.07
**Brain**	0.05	0.01	0.07	0.01	0.11	0.04
**Ovaries**	0.36	0.13	0.45	0.12	0.42	1.90
**Uterus**	0.33	0.04	0.48	0.22	0.43	0.13
**Pancreas**	0.31	0.06	0.40	0.08	0.29	0.03
**Spleen**	1.83	0.54	1.99	0.56	2.23	0.54
**Adrenal glands**	0.87	0.50	0.82	0.35	1.17	0.59
**Kidney**	5.67	1.07	3.90	0.59	3.24	0.87
**Fat**	0.22	0.10	0.14	0.10	0.13	0.09
**Muscle**	0.13	0.04	0.15	0.03	0.14	0.03
**Heart**	0.54	0.10	0.73	0.11	0.86	0.39
**Lung**	17.62	15.15	18.07	15.61	6.68	2.94
**Thyroid**	0.69	0.31	0.59	0.27	0.48	0.12
**Liver**	9.38	1.56	10.36	2.07	6.49	0.88
**Femur**	0.21	0.03	0.23	0.05	0.21	0.06
**Tumor**	0.29	0.19	0.58	0.20	0.49	0.21
**Tu/Mu**	2.56	1.88	4.05	1.53	3.31	0.74
%ID						
**Intestine**	6.11	2.12	10.25	3.68	6.78	0.77
**Stomach**	1.35	0.53	1.39	0.73	0.64	0.08

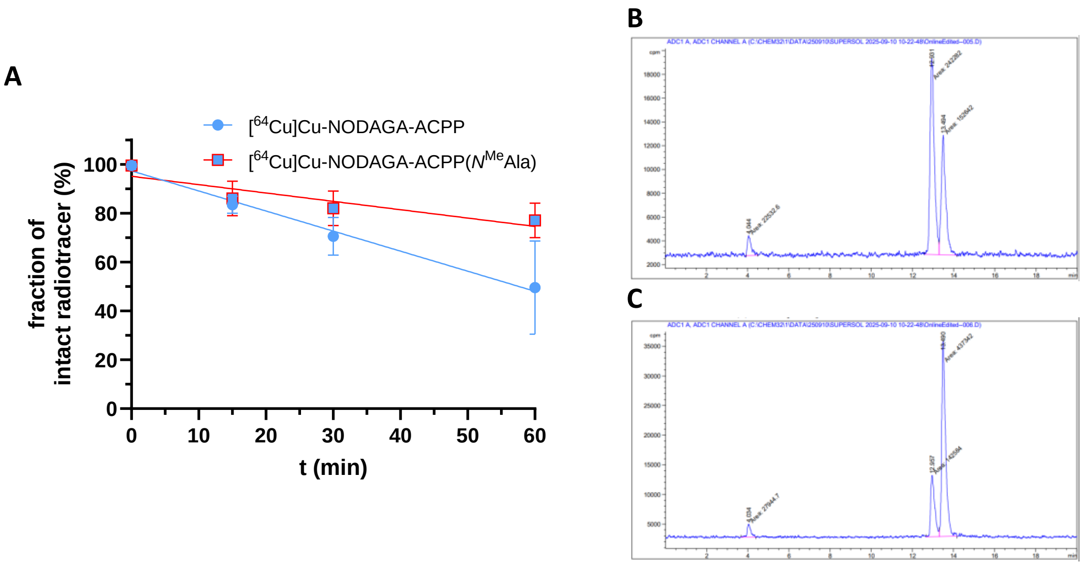

Figure S26: Cathepsin B-catalyzed cleavage of [^{64}Cu]Cu-NODAGA-ACPP ([^{64}Cu]Cu-**71**) and [^{64}Cu]Cu-NODAGA-ACPP(N^{Me}Ala) ([^{64}Cu]Cu-**74**) at pH=6 (buffered aqueous solution as specified in Table S19) and 37 °C). A radiotracer concentration of 1 µM was chosen for each compound and the enzyme concentration was 6.05 nM each. (**A**) Time course of proteolytic cleavage (data points ± SD from two independent experiments) and linear regression lines for calculation of pseudo first-order rate constants k_{obs} obtained by division of the line slopes by the initial concentration (100%). Values of 22.6 mM^{-1}s^{-1} and 9.4 mM^{-1}s^{-1} were calculated as second-order rate constants k_{obs}/[E] [18] for the cathepsin B-catalyzed conversion of [^{64}Cu]Cu-**71** and [^{64}Cu]Cu-**74**, respectively. (**B**) and (**C**) Radio-HPLC chromatograms of the reaction mixtures for [^{64}Cu]Cu-**71** and [^{64}Cu]Cu-**74**, respectively, for each 60 min of incubation time.

For stability of [^{64}Cu]Cu-**71** and [^{64}Cu]Cu-**74** in human plasma see Figure S27.

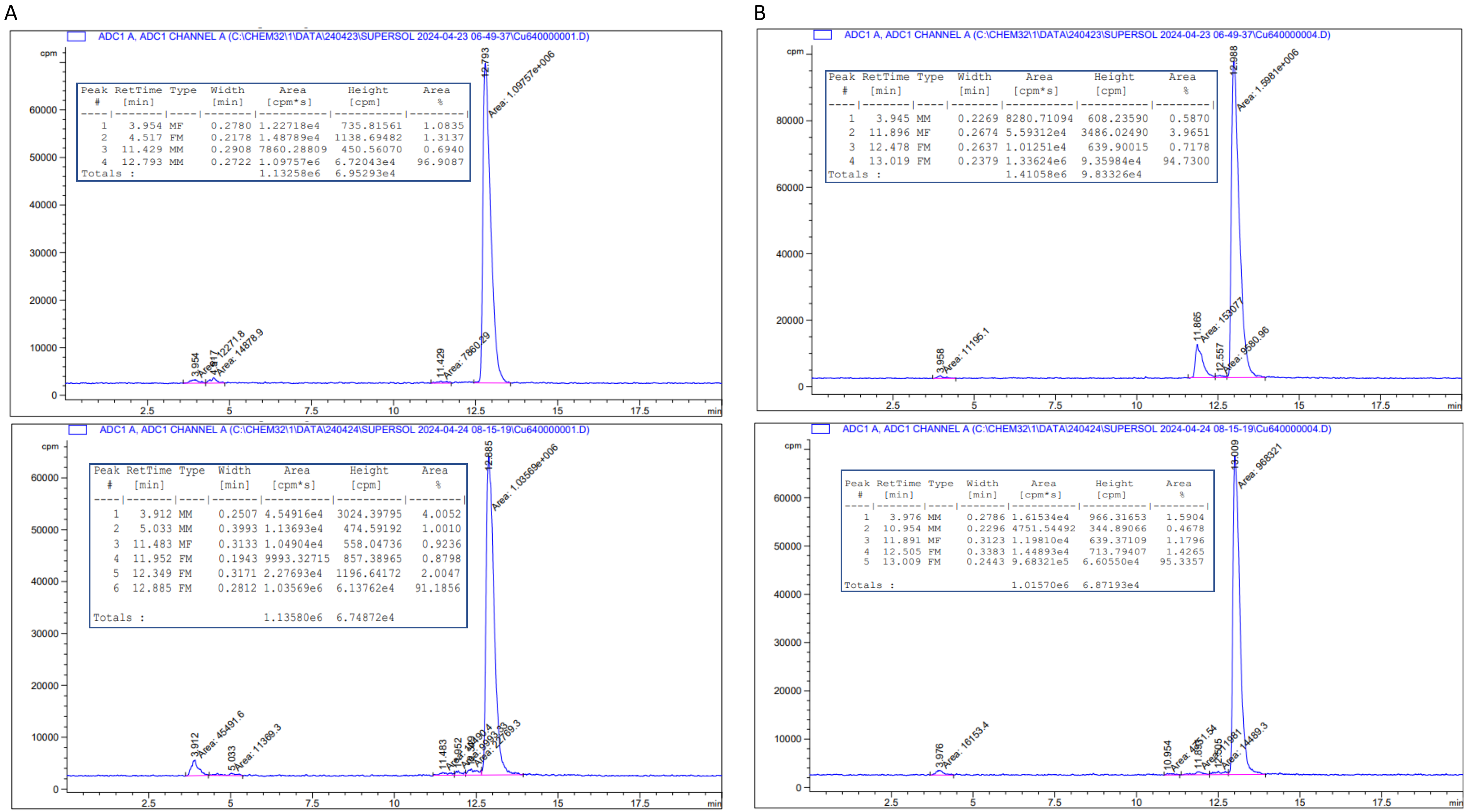

Figure S27: Radio-HPLC chromatograms after radiolabeling (top each) and after incubation for 24 h in human plasma (bottom each) for compound [^{64}Cu]Cu-71 (**A**) and [^{64}Cu]Cu-74 (**B**).

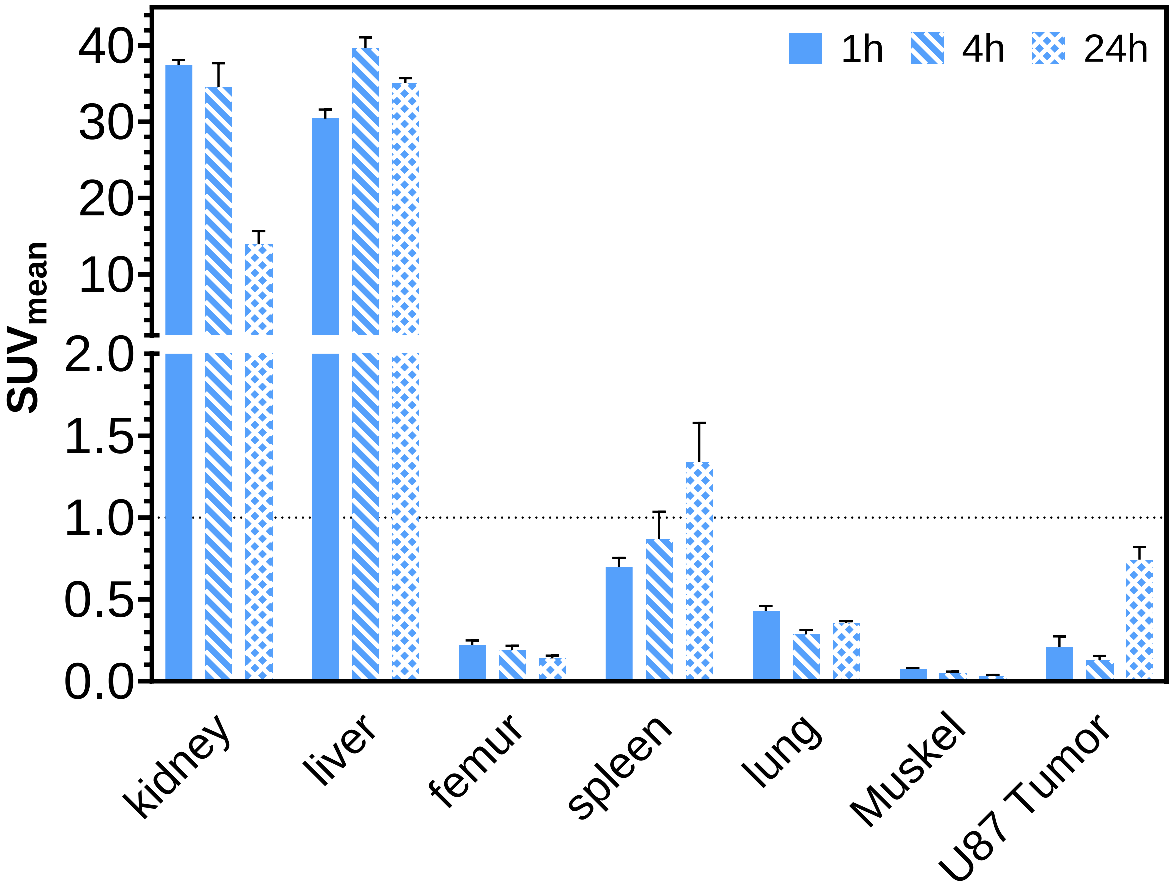

Figure S28: Ex-vivo biodistribution of [^{64}Cu]Cu-NODAGA-nACPP^{NMe}Ala ([^{64}Cu]Cu-**74**) for selected organs in U87MG-tumor-bearing mice at various time points p.i. (see legend). Uptake vales (SUV) for all organs are included in Table S9 below.

Table S9: Ex vivo biodistribution of [^{64}Cu]Cu-NODAGA-nACPP(^{NMe}Ala) ([^{64}Cu]**74**).

	1 h		4 h		24 h	
	**control**		**control**		**Control**	
SUV	**MW**	**SD**	**MW**	**SD**	**MW**	**SD**
**Blood**	0.30	0.08	0.10	0.04	0.20	0.12
**BAT**	0.07	0.02	0.04	0.01	0.07	0.02
**Skin & hairs**	0.49	0.25	0.42	0.13	0.23	0.06
**Brain**	0.02	0.00	0.02	0.01	0.03	0.01
**Ovaries**	0.04	0.01	0.05	0.01	0.04	0.01
**Uterus**	0.25	0.09	0.22	0.12	0.18	0.04
**Pancreas**	0.10	0.01	0.27	0.36	0.11	0.04
**Spleen**	0.70	0.11	0.87	0.33	1.34	0.48
**Adrenal glands**	0.03	0.01	0.03	0.01	0.04	0.02
**Kidney**	37.44	1.32	34.57	6.23	13.94	3.44
**Fat**	0.05	0.04	0.06	0.08	0.05	0.01
**Muscle**	0.08	0.01	0.05	0.02	0.03	0.01
**Heart**	0.08	0.01	0.06	0.01	0.09	0.03
**Lung**	0.43	0.06	0.29	0.05	0.35	0.02
**Thyroid**	0.04	0.01	0.04	0.01	0.09	0.04
**Liver**	30.41	2.37	39.63	2.87	35.06	1.33
**Femur**	0.22	0.05	0.19	0.05	0.14	0.03
**Tumor**	0.21	0.12	0.13	0.05	0.57	0.36
**Tu/Mu**	2.95	1.85	2.82	0.75	20.82	17.62
%ID						
**Intestine**	2.87	0.52	3.24	1.32	n.d.	
**Stomach**	0.30	0.04	0.46	0.50	n.d.	

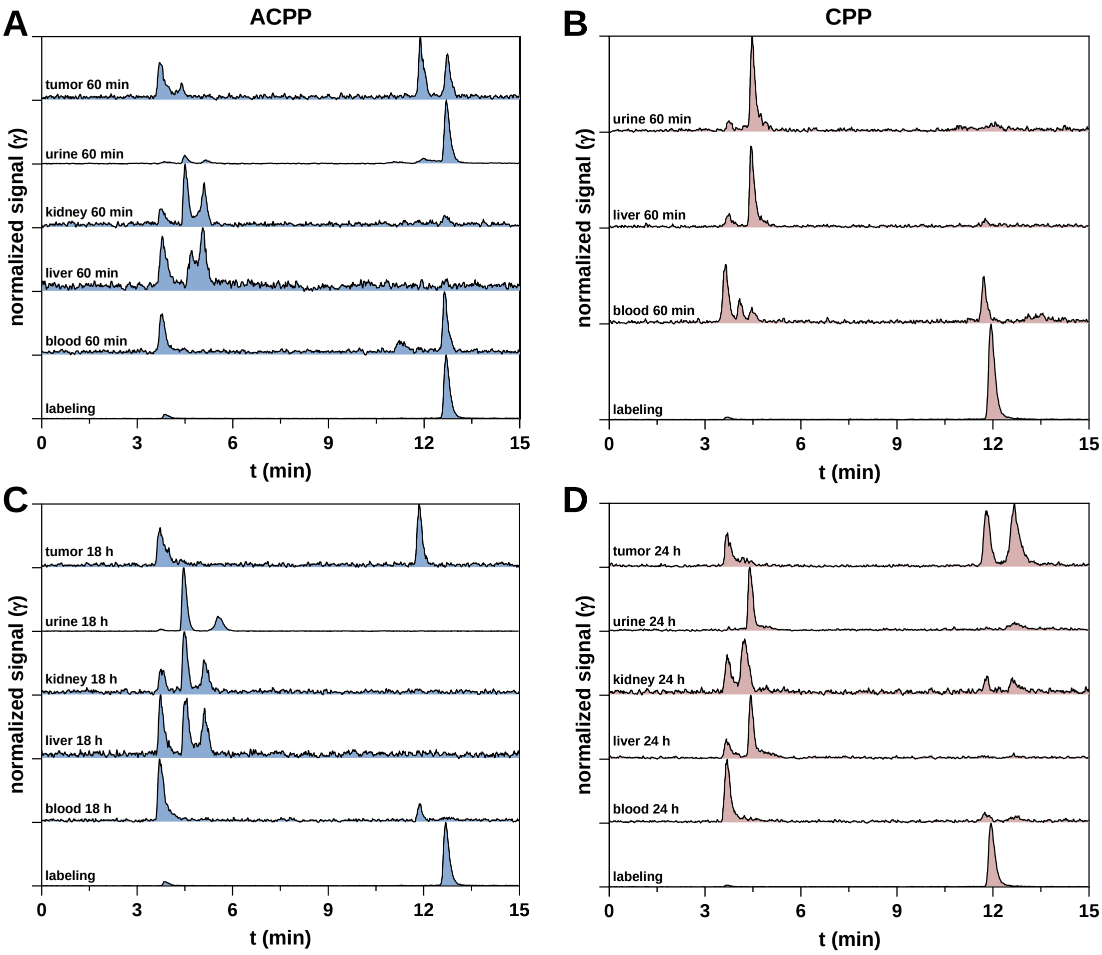

Figure S29: Metabolic stability of [^{64}Cu]Cu-**71** (**A**, **C**) and [^{64}Cu]Cu-**73** (**B**, **D**) as examined in body fluids and selected organs U87MG-bearing mice by ex vivo radio-HPLC analysis at 60 min p.i. (**A** and **B**) and 18 or 24 h p.i. (**C** and **D**, respectively).

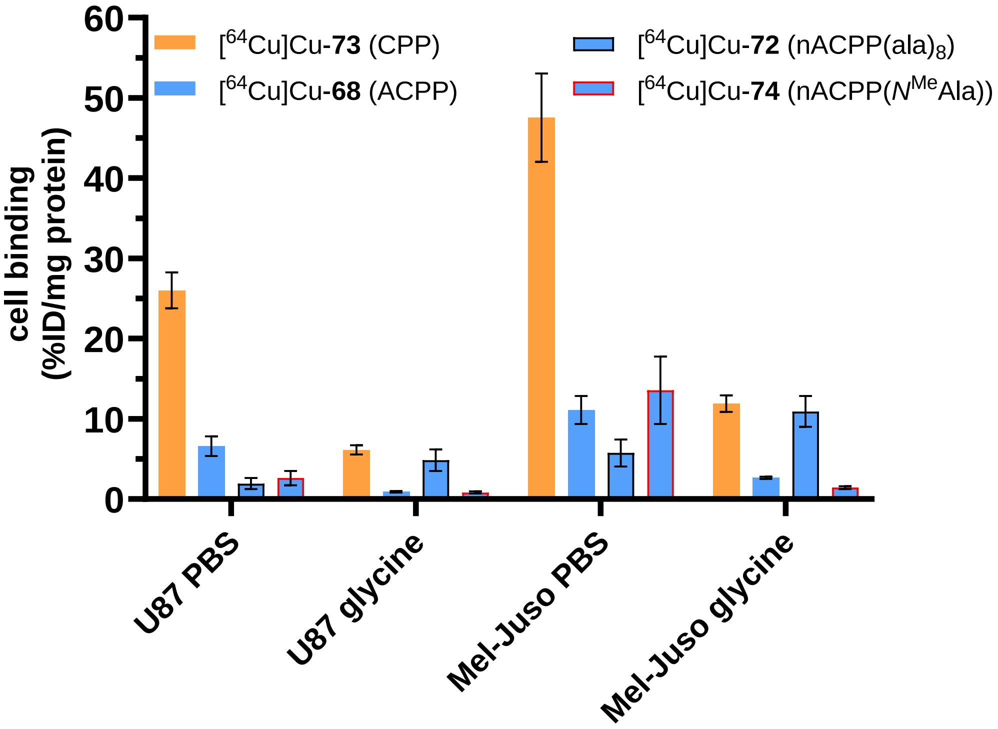

Figure S30: Binding of ^{64}Cu-labeled ACPP and control probes in U87MG and Mel-Juso cells. Data are plotted as mean values ± SEM, n=18 ([^{64}Cu]Cu-**73**), n=9 (other compounds).

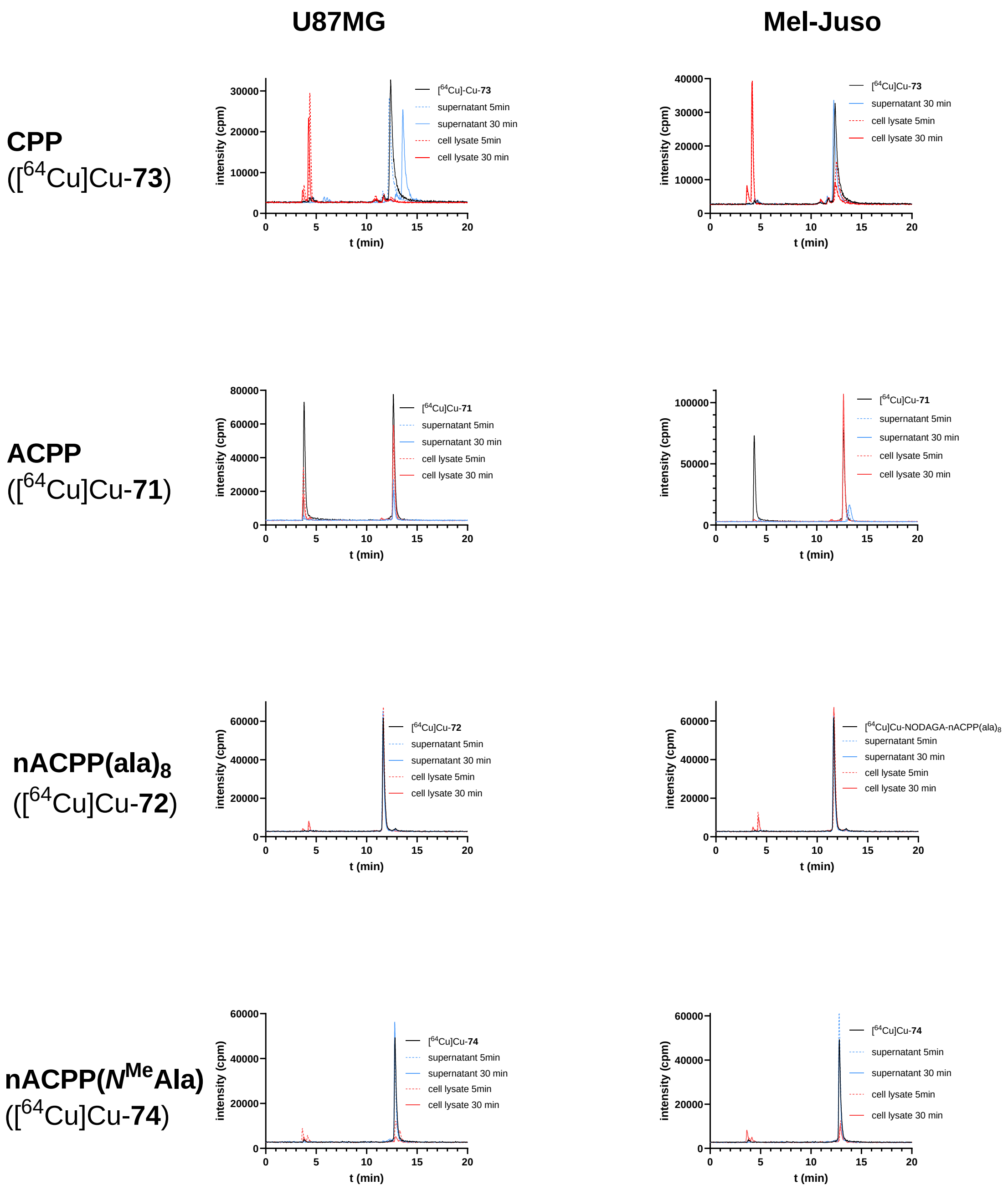

Figure S31: Proof of stability f ^{64}Cu-labeled probes and control compounds during cell binding studies in Figure S30 by radio-HPLC.

A

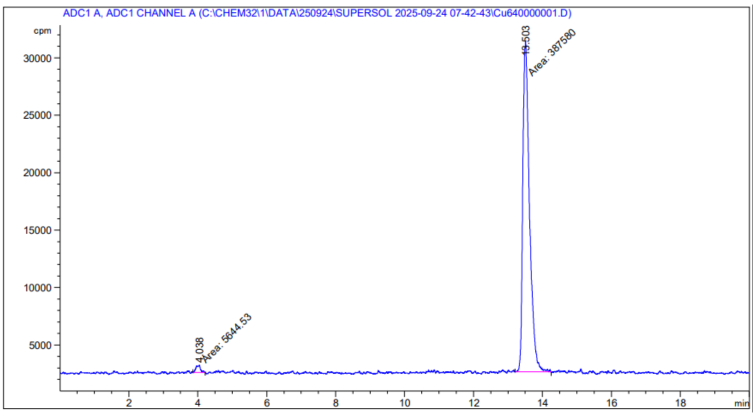

B

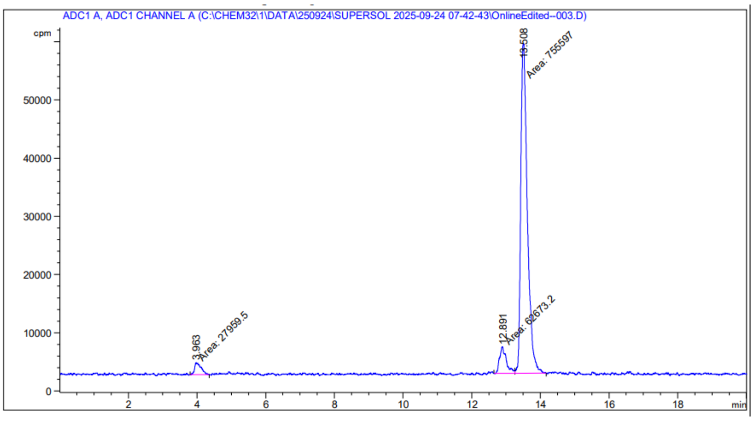

Figure 32: Proteolytic cleavage of ACPP [^{64}Cu]Cu-**71** in the presence U87MG cells cultivated in acidic medium. (**A**) Radio-HPLC of [^{64}Cu]Cu-**71** as obtained after radiolabeling. (**B**) Analysis of cell supernatant after incubation at 15 min by radio-HPLC. Later time points were excluded due to the reduced viability of the cell under these conditions.

Table S10: Comparison of substance amounts for CPPs and ACPPs in imaging experiments in mouse-based disease models reported in the literature.

		Aguilera *et al.*[19]	Duijnhoven *et al.*[20]	Duijnhoven *et al.*[21]	Duijnhoven *et al.*[22]	Kuhne *et al.* (this work)
Substance amount injected per mouse	CPP	10 nmol	60 nmol	10 nmol	10 nmol	100-140 pmol
	ACPP	10 nmol	60 nmol	10 nmol	10 nmol	100-140 pmol

Supplementary tables for experimental section

Eluent gradients for HPLC

Table S11: Analytical gradient 1.

t (min)	*Eluent A (%)*	*Eluent B (%)*
0	5	95
5	5	95
30	55	45
31	95	5
36	95	5
37	5	95
42	5	95

Table S12: Analytical gradient 2.

t (min)	*Eluent A (%)*	*Eluent B (%)*
0	15	85
5	15	85
30	65	35
31	95	5
36	95	5
37	15	85
42	15	85

Table S13: Analytical gradient 3.

t (min)	*Eluent A (%)*	*Eluent B (%)*
0	25	75
5	25	75
30	75	25
31	95	5
36	95	5
37	25	75
42	25	75

Table S14: Analytical gradient 4.

t (min)	*Eluent A (%)*	*Eluent B (%)*
0	45	55
5	45	55
30	95	5
31	95	5
36	95	5
37	45	55
42	45	55

Table S15: Representative gradient for preparative RP-HPLC.

t (min)	*Eluent A (%)*	*Eluent B (%)*
0	45	55
5	45	55
15	55	45
17	95	5
22	95	5
24	45	55
34	45	55

Table S16: UPLC-gradient 1.

t (min)	*Eluent A (%)*	*Eluent B (%)*
0	5	95
0.5	5	95
5.5	55	45
6.0	95	5
7.0	95	5
7.5	5	95
8.5	5	95

Table S17: UPLC-gradient 2.

t (min)	*Eluent A (%)*	*Eluent B (%)*
0	25	75
0.5	25	75
5.5	75	25
6.0	95	5
7.0	95	5
7.5	25	75
8.5	25	75

Table S18: UPLC-gradient 3.

t (min)	*Eluent A (%)*	*Eluent B (%)*
0	45	55
0.5	45	55
5.5	95	55
6.0	95	5
7.0	95	5
7.5	45	55
8.5	45	55

Composition of buffer solution for kinetic enzyme assays

Table S19: Composition of assay buffers for each cysteine cathepsin.

Assay buffer	*Composition*
A	100 mM sodium acetate, 100 mM NaCl, 5 mM EDTA, 0.01 % Brij 35
B	A + 10 % DMSO (V/V)
C	A + 5 mM DTT

Table S20: Composition of enzyme buffer for each cysteine cathepsin.

Enzyme	*Enzyme buffer composition*
CatB	20 mM Sodium acetate buffer (1 mM EDTA, 10 mM DTT, pH 5.0)
CatK	50 mM Citrate buffer (1 mM EDTA, 100 mM NaCl, 0.01 % CHAPS, 10 mM DTT, pH 5.0)
CatL	20 mM Malonate buffer (1 mM EDTA, 400 mM NaCl, 10 mM DTT, pH 5.5)
CatS	50 mM Phosphate buffer (2 mM EDTA, 50 mM NaCl, 0.01 % Triton X-100, 5 mM DTT, pH 6.5)

Analytical documentation of synthesized substrates and probes

Abz-Gly-Ile-Val-Arg-Ala-Lys(Dnp)-OH (1)

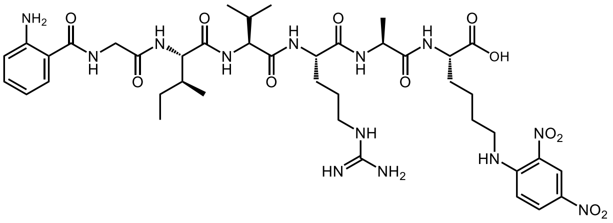

Chemical formula	$C_{41}H_{62}N_{13}O_{12}$
Molecular weight	928.02 g/mol
Exact mass	927.46 g/mol
Identified molecular ions (HR-ESI)	*m/z 928.4636 for [M+H]$^+$ (calcd. 928.4635)*<br>*m/z 809.4273 for[M-Abz+H]$^+$ (calcd. 809.4264)*
Yield	53.9 mg (52 %)
t_R	19.6 min
Purity (HPLC, 214 nm)	99.3 %
Chromatogram	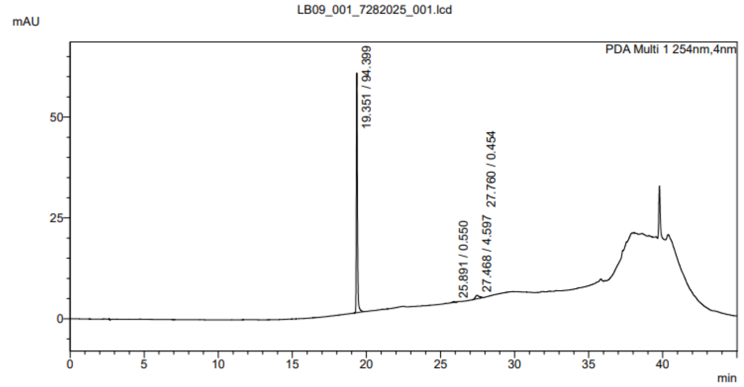
Mass spectrum (HR-ESI)	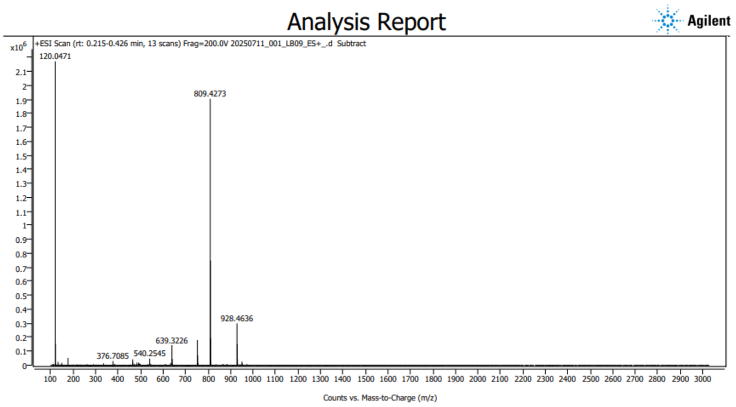

Abz-Gly-Ile-Val-Arg-OH (1a)

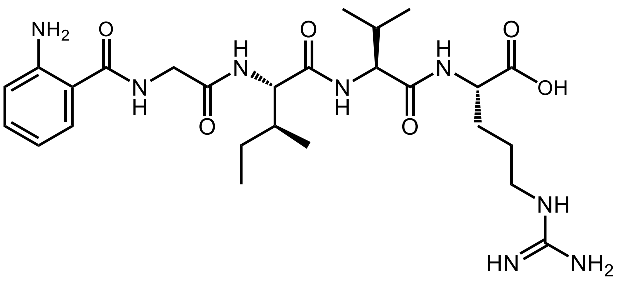

Chemical formula	$C_{26}H_{42}N_8O_6$
Molecular weight	562.67 g/mol
Exact mass	562.32 g/mol
Identified molecular ions (ESI)	*m/z 563.12 for $[M+H]^+$ (calcd. 563.33)*
Yield	45.3 mg (67%)
t_R	t_R = 15.4 min
Purity (HPLC, 214 nm)	>96%

Abz-Gly-Ile-Val-Arg-Ala-Lys(Dnp)-NH$_2$ (2)

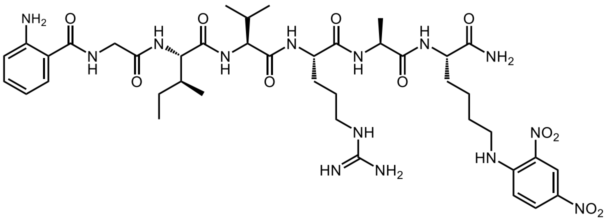

Chemical formula	$C_{41}H_{62}N_{14}O_{11}$
Molecular weight	927.03 g/mol
Exact mass	926.47 g/mol
Identified molecular ions (HR-ESI)	*m/z 927.4796 for [M+H]$^+$ (calcd. 927.4795)*<br>*m/z 808.4430 for [M-Abz+H]$^+$ (calcd. 808.4424)*
Yield	48.1 mg (46 %)
t_R	19.6 min
Purity (HPLC, 214 nm)	99.3 %
Chromatogram	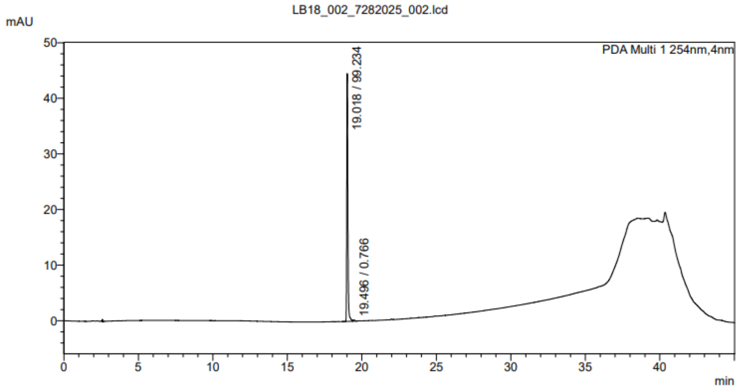
Mass spectrum (HR-ESI)	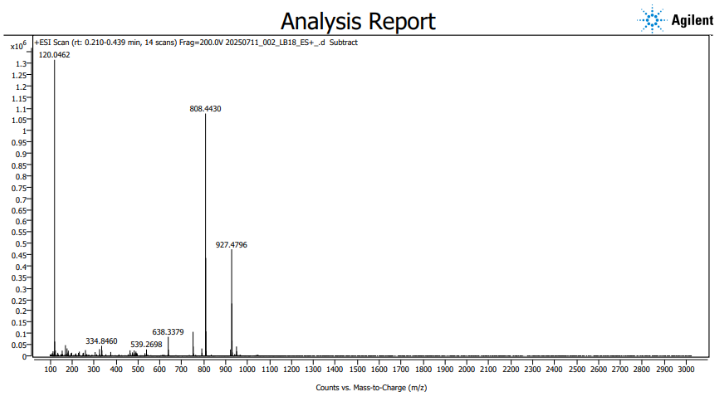

Abz-Gly-Ile-Val-Arg-Ala-Lys(Dnp)-Gly-NH$_2$ (3)

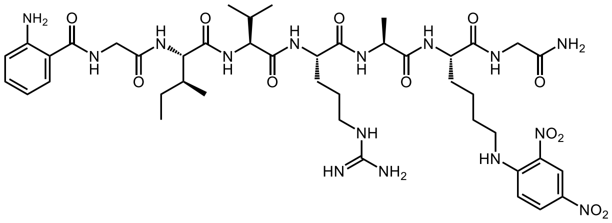

Chemical formula	$C_{43}H_{65}N_{15}O_{12}$
Molecular weight	984.09 g/mol
Exact mass	983.49 g/mol
Identified molecular ions (HR-ESI)	*m/z 984.5008 for [M+H]$^+$ (calcd. 984.5010)*<br>*m/z 865.4644 for [M-Abz+H]$^+$ (calcd. 865.4639)*
Yield	75.4 mg (77 %)
t_R	19.8 min
Purity (HPLC, 214 nm)	98.6 %
	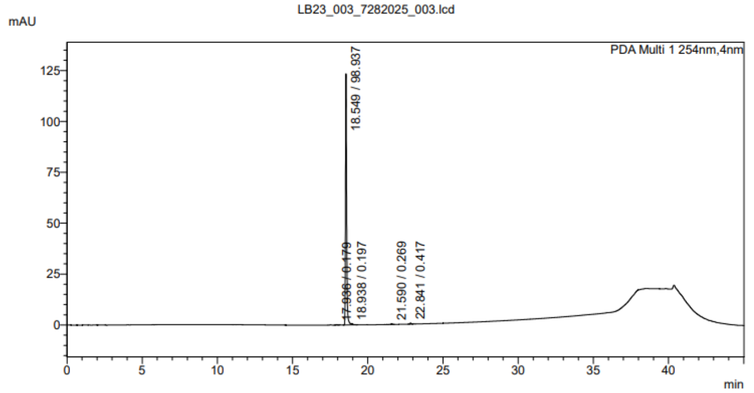
Mass spectrum (HR-ESI)	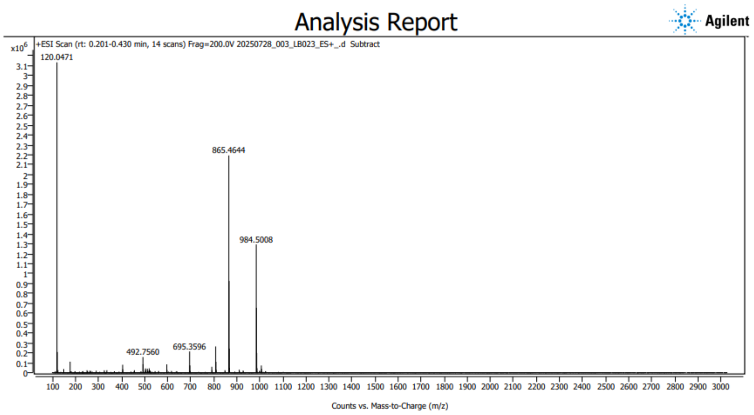

Abz-Gly-Ile-Val-Arg-Ala-Lys(Dnp)-Gly-Gly-NH_2 (4)

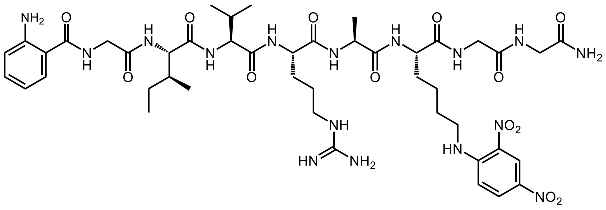

Chemical formula	$C_{45}H_{68}N_{16}O_{13}$
Molecular weight	1041,14 g/mol
Exact mass	1040.52 g/mol
Identified molecular ions (HR-ESI)	*m/z 1041.5220 for $[M+H]^+$ (calcd. 1041.5225)*<br>*m/z 922.4856 for $[M\text{-}Abz+H]^+$ (calcd. 922.4853)*
Yield	88.1 mg (69 %)
t_R	20.3 min
Purity (HPLC, 214 nm)	92.0 %
Chromatogram	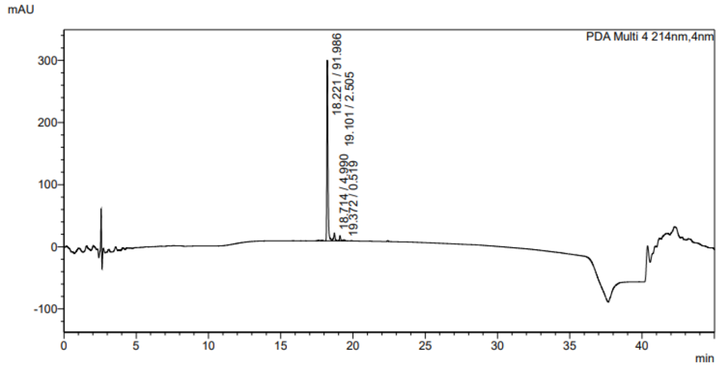
Mass spectrum (HR-ESI)	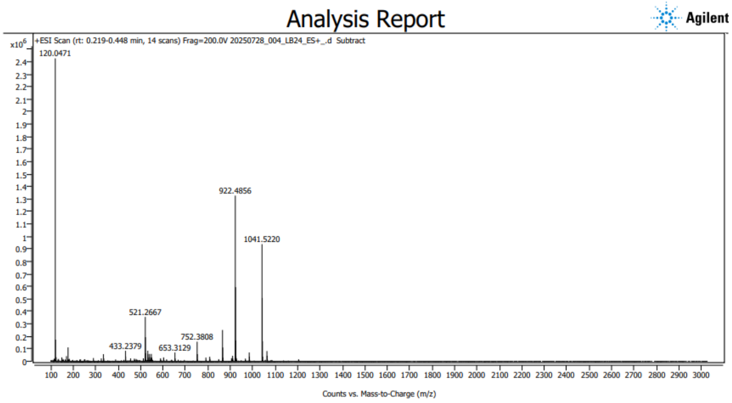

Abz-Gly-Ile-Val-Arg-Ala-Lys(Dnp)-Gly-Ser-NH$_2$ (5)

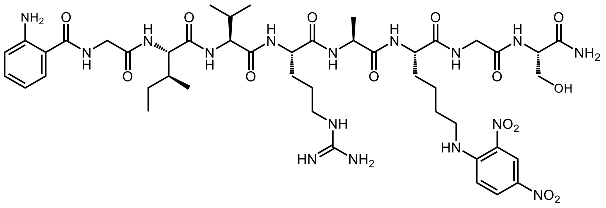

Chemical formula	$C_{46}H_{70}N_{16}O_{14}$
Molecular weight	1071.16
Exact mass	1070.53 g/mol
Identified molecular ions (HR-ESI)	*m/z 1071.5324 for [M+H]$^+$ (calcd. 1071.5330)*<br>*m/z 952.4960 for [M-Abz+H]$^+$ (calcd. 952.4959)*
Yield	67.0 mg (63 %)
t_R	17.5 min
Purity (HPLC, 214 nm)	96.9 %
Chromatogram	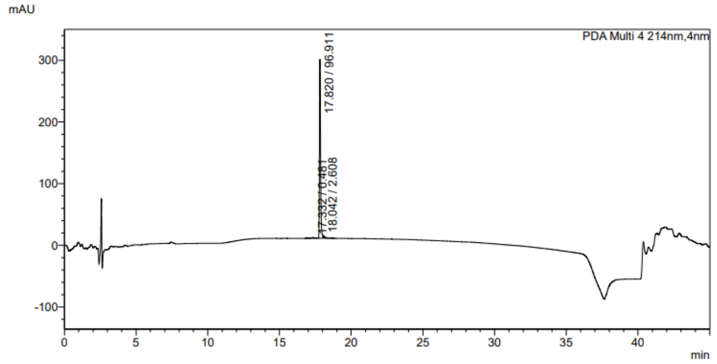
Mass spectrum (ESI)	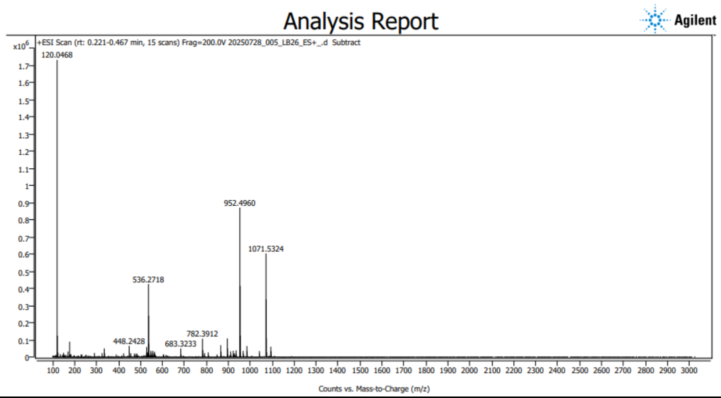

Abz-Gly-Ile-Val-Arg-Ala-Lys(Dnp)-Gly-Ala-NH$_2$ (6)

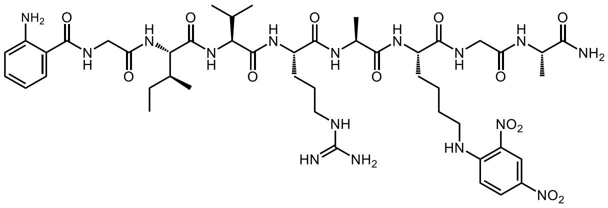

Chemical formula	$C_{46}H_{70}N_{16}O_{13}$
Molecular weight	1055.13 g/mol
Exact mass	1054.53 g/mol
Identified molecular ions (ESI)	*m/z 1055.45 for [M+H]$^+$*<br>*m/z 936.48 for [M-Abz+H]$^+$*<br>*m/z 528.39 for [M+2H]$^{2+}$*
Identified molecular ions (MALDI)	*m/z 1056.6 for [M+H]$^+$*<br>*m/z 1078.5 for [M+Na]$^+$*<br>*m/z 1094.5 for [M+K]$^+$*
Yield	40.8 mg (32 %)
Purity (HPLC, 214 nm)	> 98%
Chromatogram	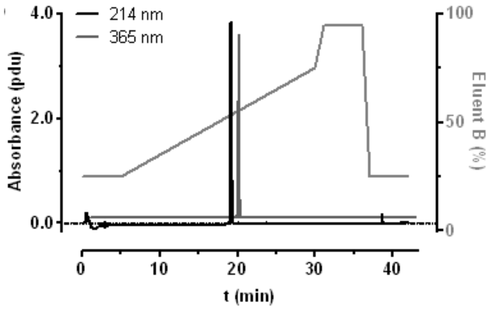
Mass spectrum (ESI)	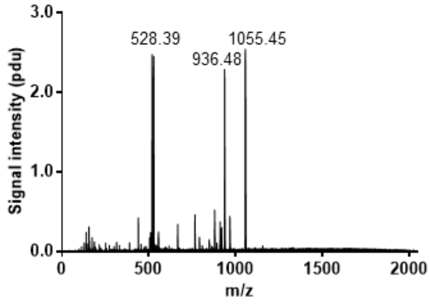
Mass spectrum (MALDI)	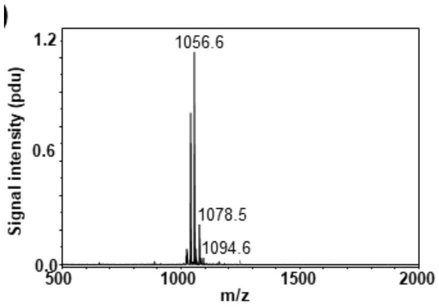

Abz-Gly-Ile-Val-Arg-Ala-Lys(Dnp)-Gly-2Abu-NH$_2$ (7)

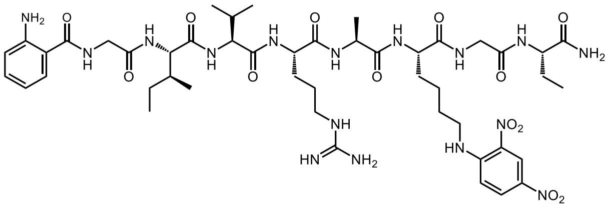

Chemical formula	$C_{47}H_{72}N_{16}O_{13}$
Molecular weight	1069.17 g/mol
Exact mass	1068.55 g/mol
Identified molecular ions (ESI)	*m/z 1069.76 for [M+H]$^+$*<br>*m/z 950.40 for [M-Abz+H]$^+$*<br>*m/z 535.39 for [M+2H]$^{2+}$*
Identified molecular ions (MALDI)	*m/z 1070.5 for [M+H]$^+$*<br>*m/z 1092.4 for [M+Na]$^+$*<br>*m/z 1108.4 for [M+K]$^+$*<br>97.6 %
Yield	57.4 mg (34 %)
Purity (HPLC, 214 nm)	97.6 %
Chromatogram	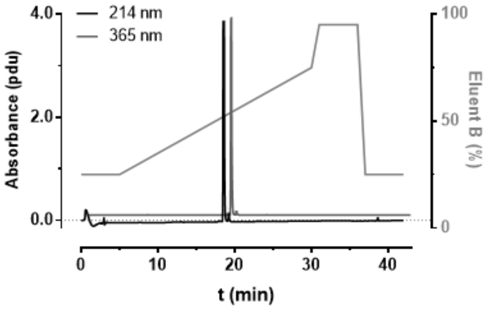
Mass spectrum (ESI)	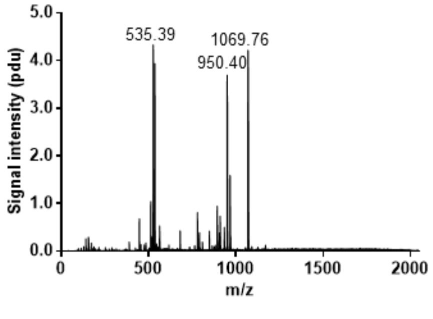
Mass spectrum (MALDI)	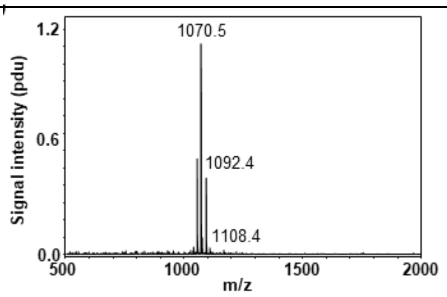

Abz-Gly-Ile-Val-Arg-Ala-Lys(Dnp)-Gly-Nva-NH$_2$ (8)

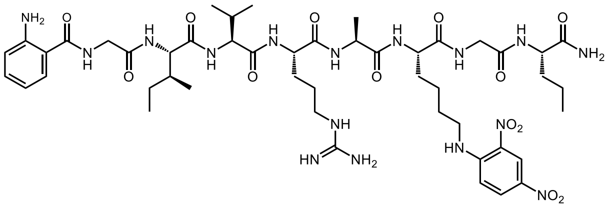

Chemical formula	$C_{48}H_{74}N_{16}O_{13}$
Molecular weight	1083.20 g/mol
Exact mass	1082.56 g/mol
Identified molecular ions (ESI)	*m/z 1083.88 for [M+H]$^+$*<br>*m/z 964.52 for [M-Abz+H]$^+$*<br>*m/z 542.40 for [M+2H]$^{2+}$*
Identified molecular ions (MALDI)	*m/z 1084.6 for [M+H]$^+$*<br>*m/z 1106.5 for [M+Na]$^+$*<br>*m/z 1122.5 for [M+K]$^+$*
Yield	50.0 mg (38 %)
Purity (HPLC, 214 nm)	97.1 %
Chromatogram	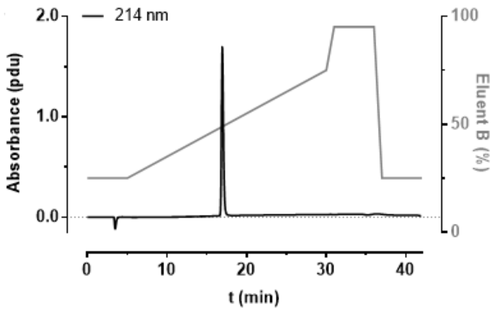
Mass spectrum (ESI)	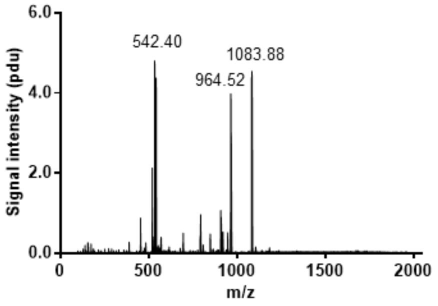
Mass spectrum (MALDI)	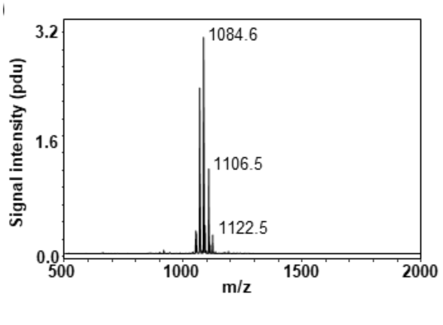

Abz-Gly-Ile-Val-Arg-Ala-Lys(Dnp)-Gly-Pro-NH$_2$ (9)

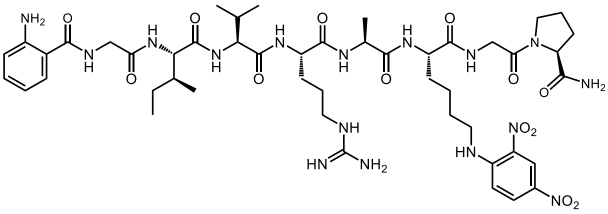

Chemical formula	$C_{48}H_{72}N_{16}O_{13}$
Molecular weight	1081.18 g/mol
Exact mass	1080.55 g/mol
Identified molecular ions (MALDI)	*m/z 1082.5 for [M+H]$^+$*<br>*m/z 1104-4 for [M+Na]$^+$*
Yield	24.4 mg (84 % from 29.2 mg crude peptide)
Purity (HPLC, 214 nm)	> 98 %
Chromatogram	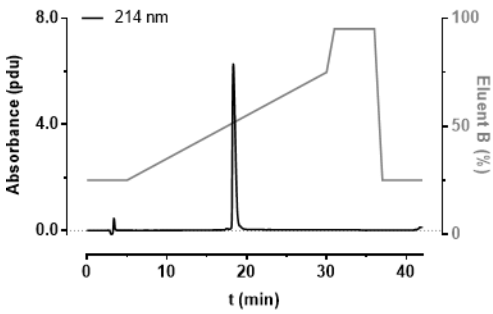
Mass spectrum (MALDI)	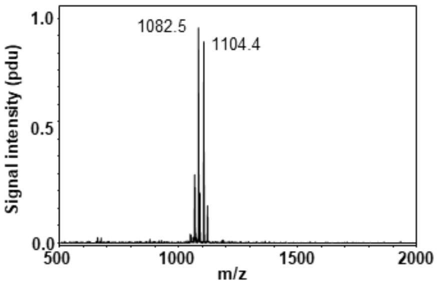

Abz-Gly-Ile-Val-Arg-Ala-Lys(Dnp)-Gly-Nle-NH_2 (10)

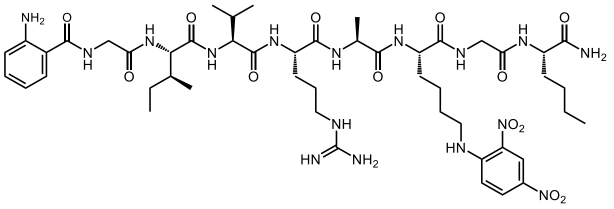

Chemical formula	$C_{49}H_{76}N_{16}O_{13}$
Molecular weight	1097.23 g/mol
Exact mass	1096.58 g/mol
Identified molecular ions (ESI)	*m/z 1097.80 for $[M+H]^+$*<br>*m/z 978.44 for $[M\text{-}Abz+H]^+$*<br>*m/z 549.40 for $[M+2H]^{2+}$*
Identified molecular ions (MALDI)	*m/z 1098.6 for $[M+H]^+$*<br>*m/z 1120.5 for $[M+Na]^+$*<br>*m/z 1136.6 for $[M+K]^+$*
Yield	71.6 mg (54 %)
Purity (HPLC, 214 nm)	> 98 %
Chromatogram	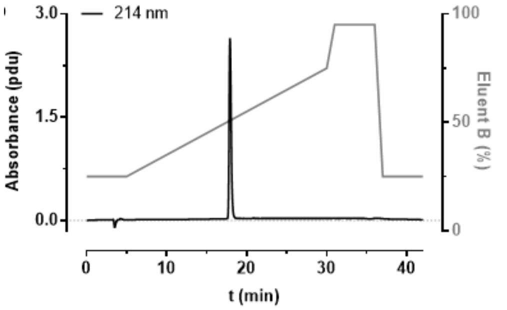
Mass spectrum (ESI)	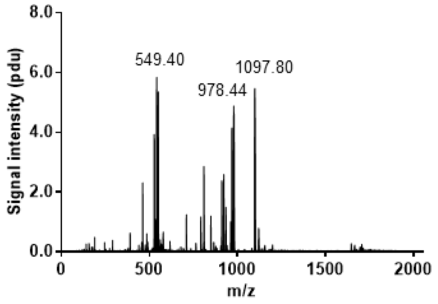
Mass spectrum (MALDI)	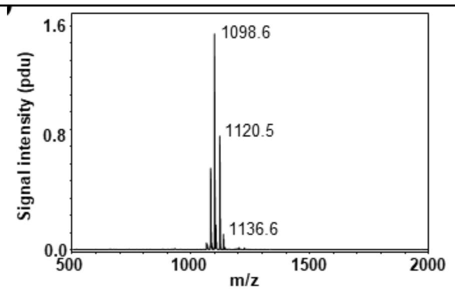

Abz-Gly-Ile-Val-Arg-Ala-Lys(Dnp)-Gly-Val-NH2 (11)

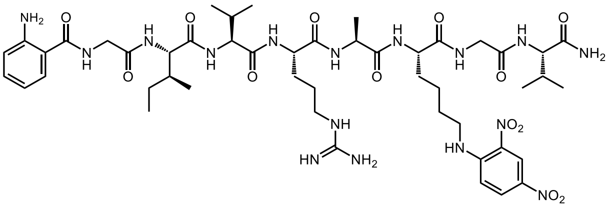

Chemical formula	$C_{48}H_{74}N_{16}O_{13}$
Molecular weight	1083.22 g/mol
Exact mass	1082.56 g/mol
Identified molecular ions (ESI)	*m/z 1083.78 for [M+H]*$^{+}$<br>*m/z 964.52 for [M-Abz+H]*$^{+}$<br>*m/z 542.40 for [M+2H]*$^{2+}$
Identified molecular ions (MALDI)	*m/z 1084.4 for [M+H]*$^{+}$<br>*m/z 1106.3 for [M+Na]*$^{+}$<br>*m/z 1122.2 for [M+K]*$^{+}$
Yield	15.9 mg (12 %)
Purity (HPLC, 214 nm)	96.4 %
Chromatogram	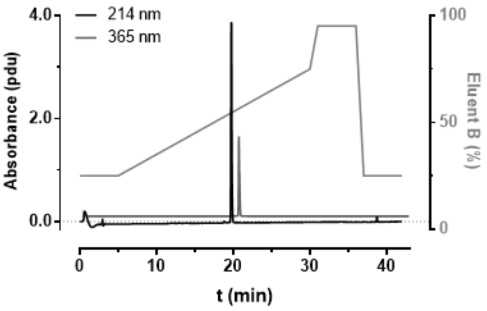
Mass spectrum (ESI)	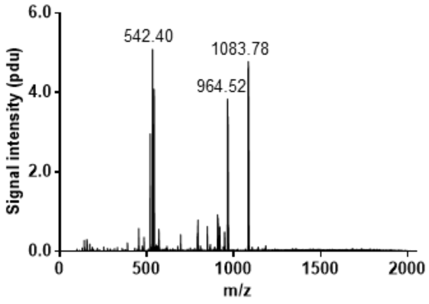
Mass spectrum (MALDI)	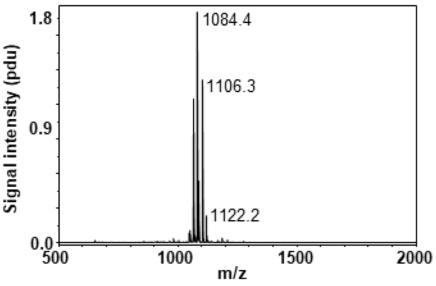

Abz-Gly-Ile-Val-Arg-Ala-Lys(Dnp)-Gly-Ile-NH_2 (12)

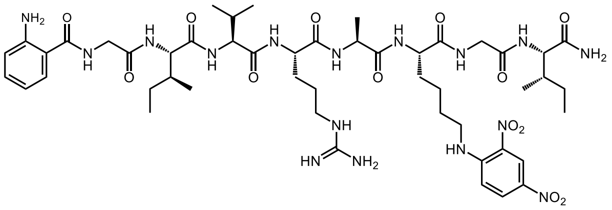

Chemical formula	$C_{49}H_{76}N_{16}O_{13}$
Molecular weight	1097.25 g/mol
Exact mass	1096.58 g/mol
Identified molecular ions (ESI)	*m/z 1097.90 for [M+H]$^+$*<br>*m/z 978.84 for [M-Abz+H]$^+$*<br>*m/z 549.40 for [M+2H]$^{2+}$*
Identified molecular ions (MALDI)	*m/z 1098.6 for [M+H]$^+$*<br>*m/z 1120.4 for [M+Na]$^+$*<br>*m/z 1136.5 for [M+K]$^+$*
Yield	40.9 mg (31 %)
Purity (HPLC, 214 nm)	> 98 %
Chromatogram	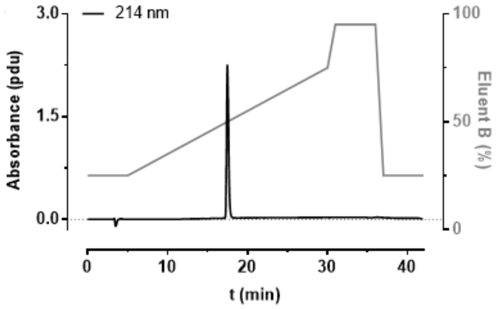
Mass spectrum (ESI)	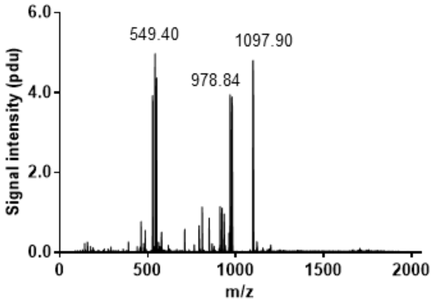
Mass spectrum (MALDI)	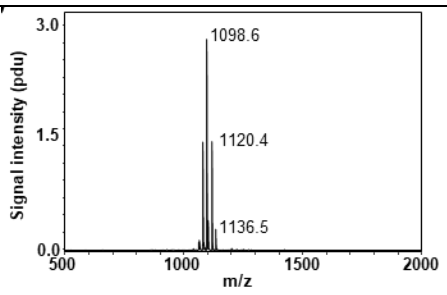

Abz-Gly-Ile-Val-Arg-Ala-Lys(Dnp)-Gly-Tle-NH₂ (13)

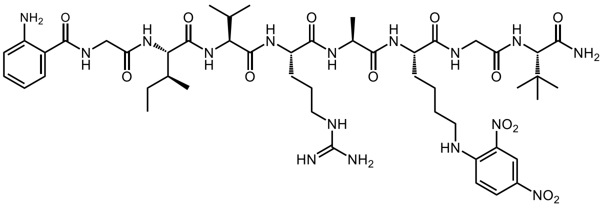

Chemical formula	$C_{49}H_{76}N_{16}O_{13}$
Molecular weight	1097.25 g/mol
Exact mass	1096.58 g/mol
Identified molecular ions (ESI)	*m/z 1097.90 for [M+H]$^+$*<br>*m/z 967.42 for [M-Abz+H]$^+$*<br>*m/z 549.40 for [M+2H]$^{2+}$*<br>1097.23 g/mol
Identified molecular ions (MALDI)	*m/z 1098.5 for [M+H]$^+$*<br>*m/z 1120.5 for [M+Na]$^+$*<br>*m/z 1136.7 for [M+K]$^+$*
Yield	43.5 mg (33 %)
Purity (HPLC, 214 nm)	> 98 %
Chromatogram	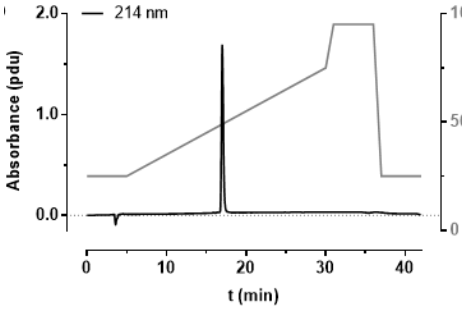
Mass spectrum (ESI)	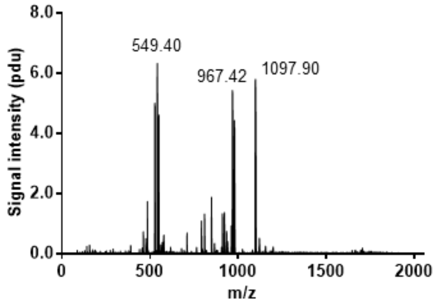
Mass spectrum (MALDI)	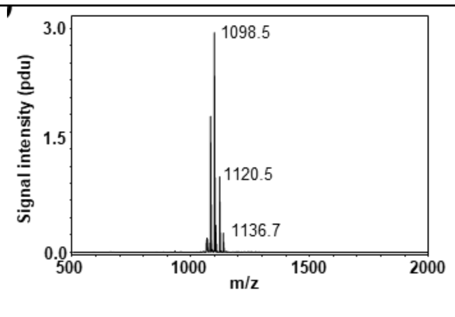

Abz-Gly-Ile-Val-Arg-Ala-Lys(Dnp)-Gly-Leu-NH_2 (14)

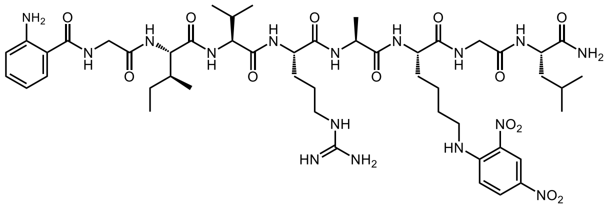

Chemical formula	$C_{49}H_{76}N_{16}O_{13}$
Molecular weight	1097.25 g/mol
Exact mass	1096.58 g/mol
Identified molecular ions (ESI)	*m/z 1097.80 for [M+H]$^+$*<br>*m/z 978.54 for [M-Abz+H]$^+$*<br>*m/z 549.40 for [M+2H]$^{2+}$*
Identified molecular ions (MALDI)	*m/z 1098.6 for [M+H]$^+$*<br>*m/z 1120.7 for [M+Na]$^+$*<br>*m/z 1136.6 for [M+K]$^+$*
Yield	44.7 mg (34 %)
Purity (HPLC, 214 nm)	> 98 %
Chromatogram	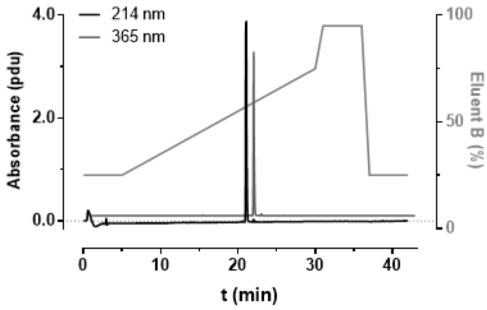
Mass spectrum (ESI)	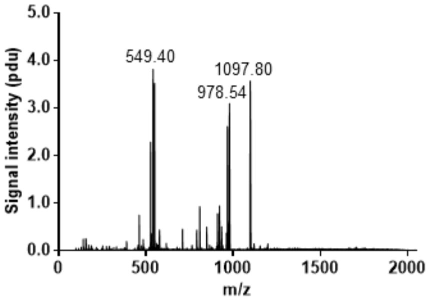
Mass spectrum (MALDI)	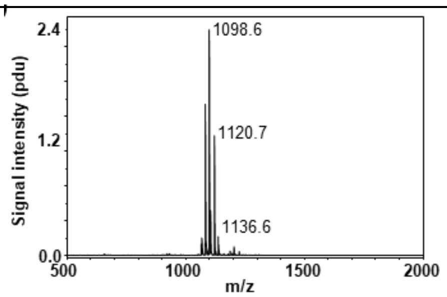

Abz-Gly-Ile-Val-Arg-Ala-Lys(Dnp)-Gly-Cprg-NH_2 (15)

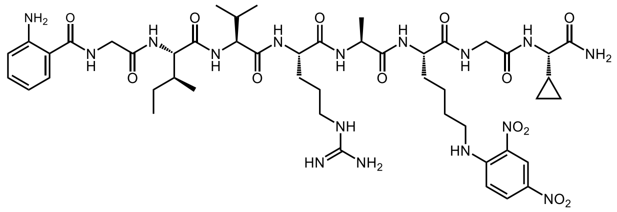

Chemical formula	$C_{48}H_{72}N_{16}O_{13}$
Molecular weight	1081.18 g/mol
Exact mass	1080.55 g/mol
Identified molecular ions (ESI)	*m/z 1081.88 for $[M+H]^+$*<br>*m/z 962.52 for $[M\text{-}Abz+H]^+$*<br>*m/z 541.40 for $[M+2H]^{2+}$*
Identified molecular ions (MALDI)	*m/z 1082.3 for $[M+H]^+$*<br>*m/z 1104.6 for $[M+Na]^+$*<br>*m/z 1120.4 for $[M+K]^+$*
Yield	65.6 mg (50 %)
Purity (HPLC, 214 nm)	> 98 %
Chromatogram	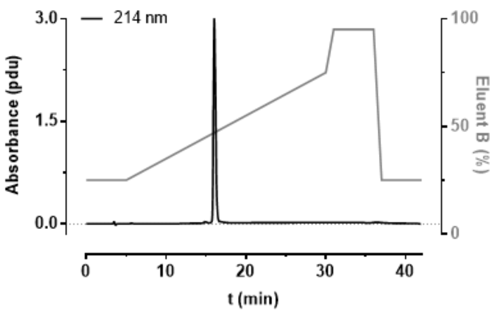
Mass spectrum (ESI)	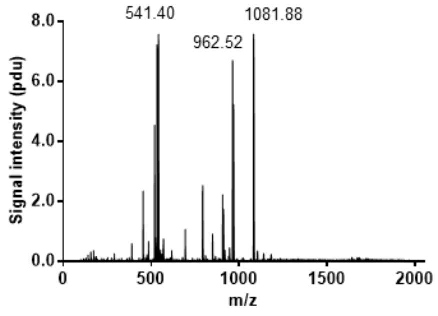
Mass spectrum (MALDI)	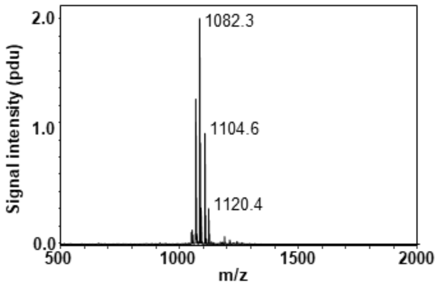

Abz-Gly-Ile-Val-Arg-Ala-Lys(Dnp)-Gly-Cbg-NH$_2$ (16)

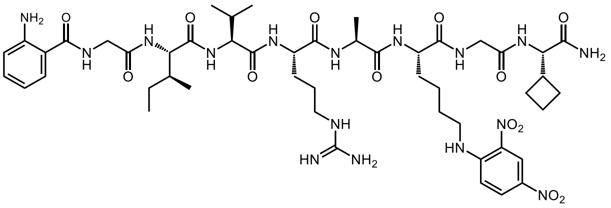

Chemical formula	$C_{49}H_{74}N_{16}O_{13}$
Molecular weight	1095.21 g/mol
Exact mass	1094.56
Identified molecular ions (ESI)	*m/z 1095.90 for [M+H]$^+$*<br>*m/z 976.44 for [M-Abz+H]$^+$*<br>*m/z 548.30 for [M+2H]$^{2+}$*
Identified molecular ions (MALDI)	*m/z 1096.5 for [M+H]$^+$*<br>*m/z 1118.4 for [M+Na]$^+$*<br>*m/z 1134.5 for [M+K]$^+$*
Yield	62.6 mg (47 %)
Purity (HPLC, 214 nm)	95.3 %
Chromatogram	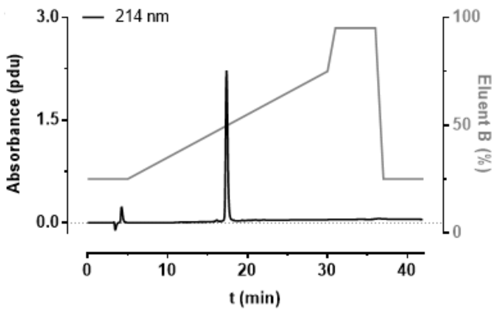
Mass spectrum (ESI)	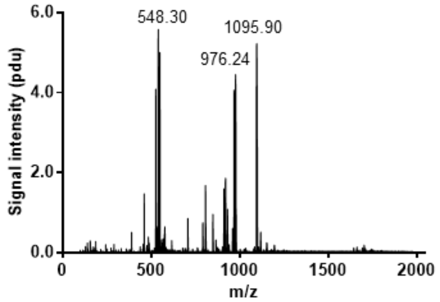
Mass spectrum (MALDI)	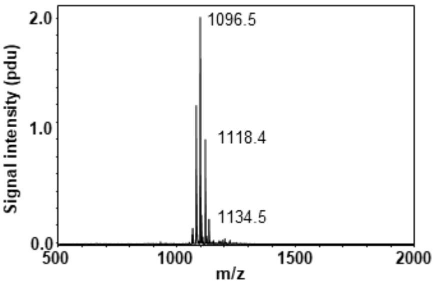

Abz-Gly-Ile-Val-Arg-Ala-Lys(Dnp)-Gly-Cpeg-NH_2 (17)

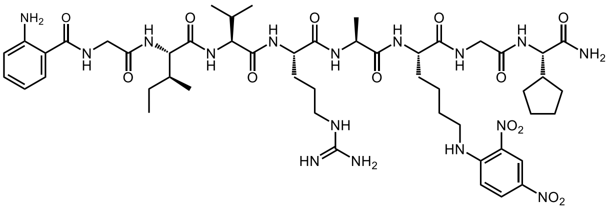

Chemical formula	$C_{50}H_{76}N_{16}O_{13}$
Molecular weight	1109.24 g/mol
Exact mass	1108.58 g/mol
Identified molecular ions (ESI)	*m/z 1109.72 for $[M+H]^+$*<br>*m/z 990.46 for $[M\text{-}Abz+H]^+$*<br>*m/z 555.41 for $[M+2H]^{2+}$*
Identified molecular ions (MALDI)	*m/z 1110.2 for $[M+H]^+$*<br>*m/z 1132.4 for $[M+Na]^+$*<br>*m/z 1149.2 for $[M+K]^+$*
Yield	28.6 mg (21 %)
Purity (HPLC, 214 nm)	> 98 %
Chromatogram	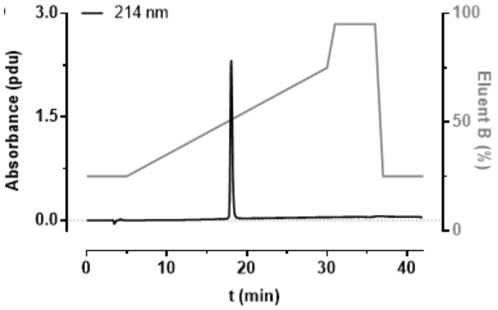
Mass spectrum (ESI)	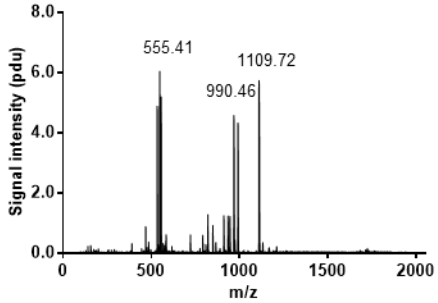
Mass spectrum (MALDI)	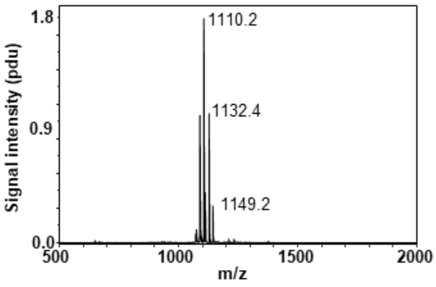

Abz-Gly-Ile-Val-Arg-Ala-Lys(Dnp)-Gly-Phg-NH$_2$ (18)

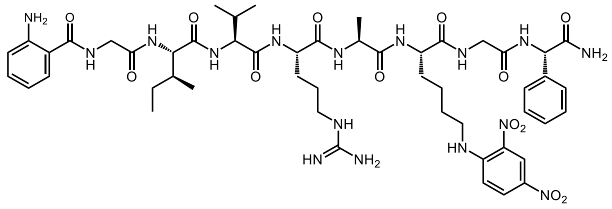

Chemical formula	$C_{51}H_{72}N_{16}O_{13}$
Molecular weight	1117.22 g/mol
Exact mass	1116.55 g/mol
Identified molecular ions (ESI)	*m/z 1117.43 for [M+H]$^+$*<br>*m/z 998.47 for [M-Abz+H]$^+$*<br>*m/z 559.31 for [M+2H]$^{2+}$*
Identified molecular ions (MALDI)	*m/z 1118.5 for [M+H]$^+$*<br>*m/z 1140.6 for [M+Na]$^+$*<br>*m/z 1156.6 for [M+K]$^+$*
Yield	16.8 mg (13 %)
Purity (HPLC, 214 nm)	95.9 %
Chromatogram	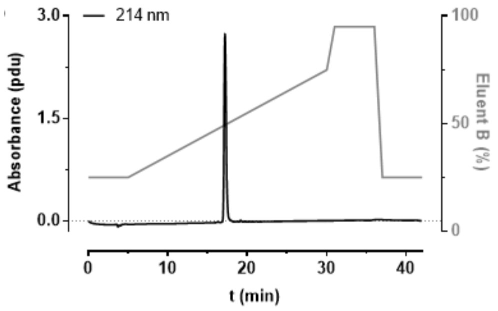
Mass spectrum (ESI)	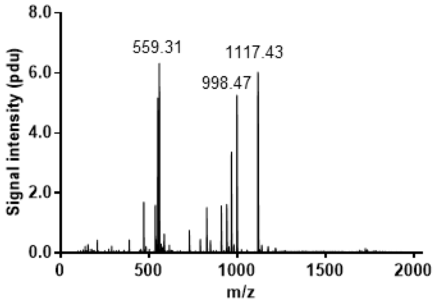
Mass spectrum (MALDI)	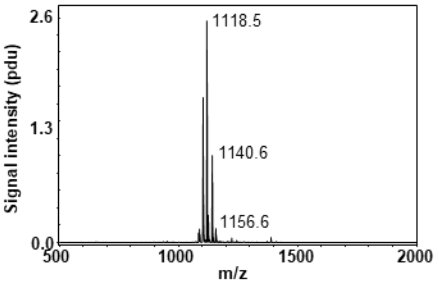

Abz-Gly-Ile-Val-Arg-Ala-Lys(Dnp)-Gly-Phe-NH$_2$ (19)

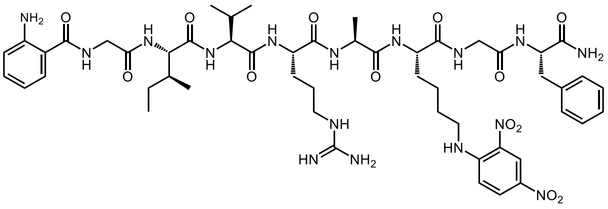

Chemical formula	$C_{52}H_{74}N_{16}O_{13}$
Molecular weight	1131.24 g/mol
Exact mass	1130.56 g/mol
Identified molecular ions (ESI)	*m/z 1132.15 for [M+H]$^+$*<br>*m/z 1012.49 for [M-Abz+H]$^+$*<br>*m/z 566.32 for [M+2H]$^{2+}$*
Identified molecular ions (MALDI)	*m/z 1132.6 for [M+H]$^+$*<br>*m/z 1154.5 for [M+Na]$^+$*<br>*m/z 1170.3 for [M+K]$^+$*
Yield	38.5 mg (28 %)
Purity (HPLC, 214 nm)	98.0 %
Chromatogram	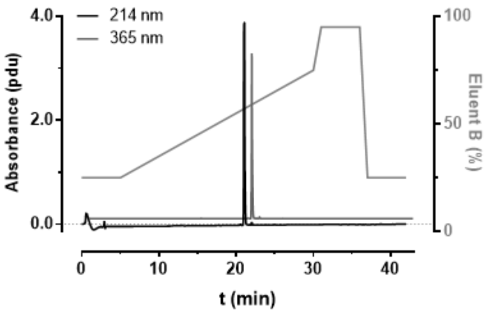
Mass spectrum (ESI)	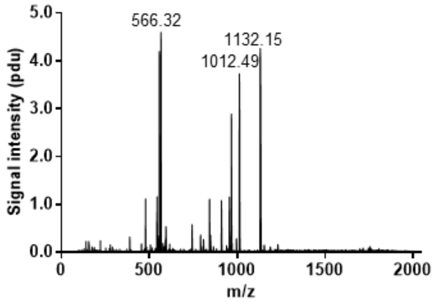
Mass spectrum (MALDI)	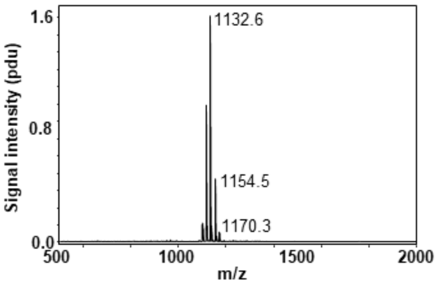

Abz-Gly-Ile-Val-Arg-Ala-Lys(Dnp)-Gly-Tyr-NH₂ (20)

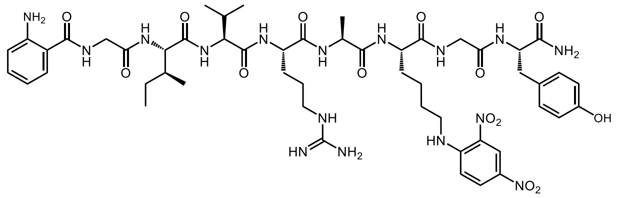

Chemical formula	$C_{52}H_{74}N_{16}O_{14}$
Molecular weight	1147.24 g/mol
Exact mass	1146.56 g/mol
Identified molecular ions (ESI)	*m/z 1147.87 for [M+H]⁺*<br>*m/z 1028.81 for [M-Abz+H]⁺*<br>*m/z 574.32 for [M+2H]²⁺*
Identified molecular ions (MALDI)	*m/z 1148.6 for [M+H]⁺*<br>*m/z 1170.5 for [M+Na]⁺*<br>*m/z 1186.6 for [M+K]⁺*
Yield	57.2 mg (42 %)
Purity (HPLC, 214 nm)	97.0 %
Chromatogram	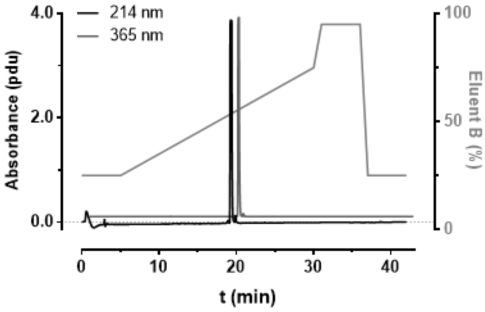
Mass spectrum (ESI)	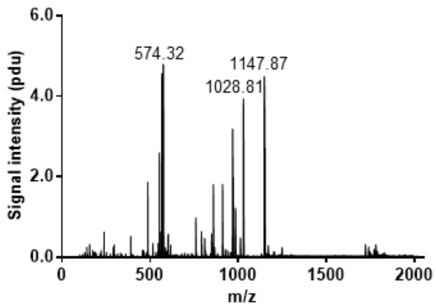
Mass spectrum (MALDI)	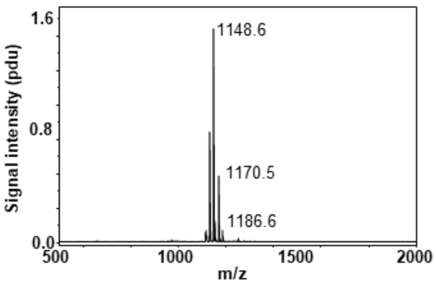

Abz-Gly-Ile-Val-Arg-Ala-Lys(Dnp)-Gly-His-NH_2 (21)

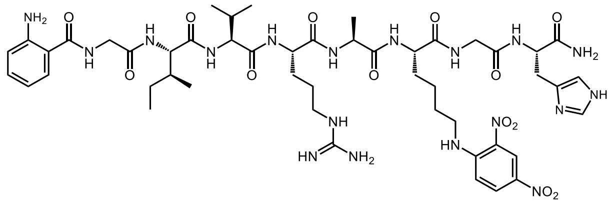

Chemical formula	$C_{49}H_{72}N_{18}O_{13}$
Molecular weight	1121.21 g/mol
Exact mass	1120.55 g/mol
Identified molecular ions (ESI)	*m/z 1121.73 for $[M+H]^+$*<br>*m/z 1002.77 for $[M\text{-}Abz+H]^+$*<br>*m/z 561.41 for $[M+2H]^{2+}$*
Identified molecular ions (MALDI)	*m/z 1122.5 for $[M+H]^+$*<br>*m/z 1144.6 for $[M+Na]^+$*<br>*m/z 1160.5 for $[M+K]^+$*
Yield	45.8 mg (31 %)
Purity (HPLC, 214 nm)	> 98 %
Chromatogram	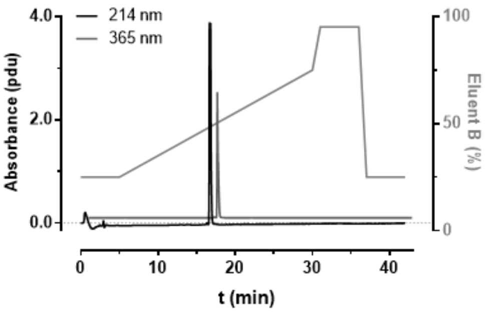
Mass spectrum (ESI)	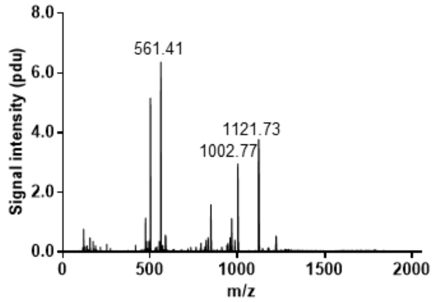
Mass spectrum (MALDI)	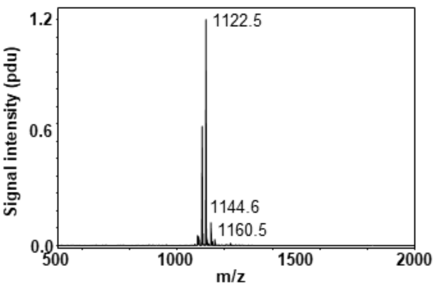

Abz-Gly-Ile-Val-Arg-Ala-Lys(Dnp)-Gly-Trp-NH$_2$ (22)

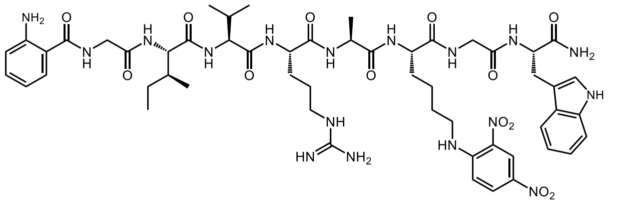

Chemical formula	$C_{54}H_{75}N_{17}O_{13}$
Molecular weight	1170.28 g/mol
Exact mass	1169.57 g/mol
Identified molecular ions (ESI)	*m/z 1170.89 for [M+H]$^+$*<br>*m/z 1051.84 for [M-Abz+H]$^+$*<br>*m/z 585.83 for [M+2H]$^{2+}$*
Identified molecular ions (MALDI)	*m/z 1171.6 for [M+H]$^+$*<br>*m/z 1193.5 for [M+Na]$^+$*
Yield	25.3 mg (18 %)
Purity (HPLC, 214 nm)	> 98 %
Chromatogram	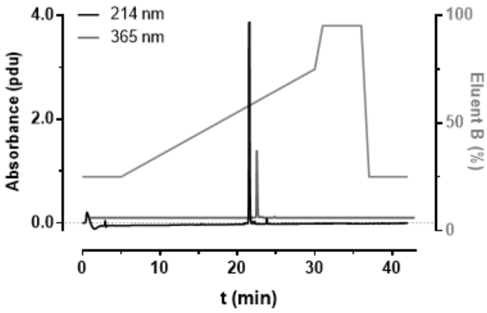
Mass spectrum (ESI)	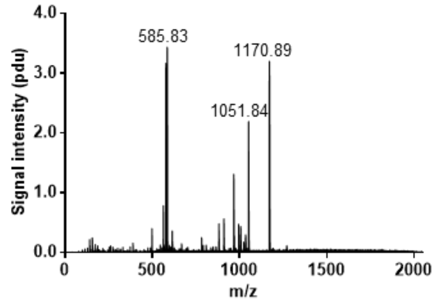
Mass spectrum (MALDI)	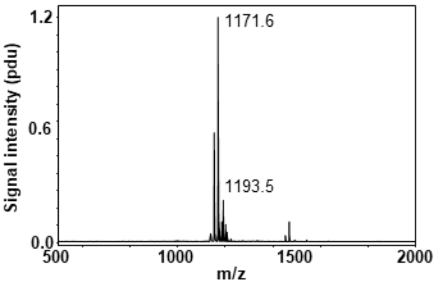

Abz-Gly-Ile-Val-Arg-Ala-Lys(Dnp)-Gly-Thr-NH$_2$ (23)

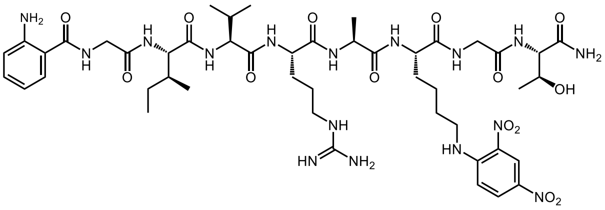

Chemical formula	$C_{47}H_{72}N_{16}O_{14}$
Molecular weight	1085.17 g/mol
Exact mass	1084.54 g/mol
Identified molecular ions (ESI)	*m/z 1085.79 for [M+H]$^+$*<br>*m/z 966.52 for [M-Abz+H]$^+$*<br>*m/z 543.30 for [M+2H]$^{2+}$*
Identified molecular ions (MALDI)	*m/z 1086.2 for [M+H]$^+$*<br>*m/z 1108.3 for [M+Na]$^+$*<br>*m/z 1124.2 for [M+K]$^+$*
Yield	47.7 mg (36 %)
Purity (HPLC, 214 nm)	> 96 %
Chromatogram	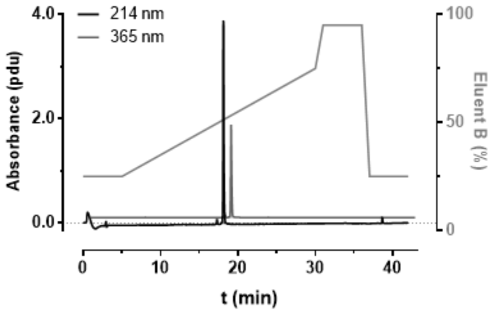
Mass spectrum (ESI)	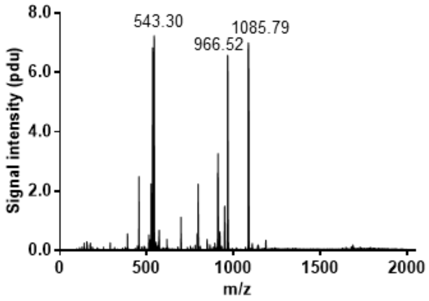
Mass spectrum (MALDI)	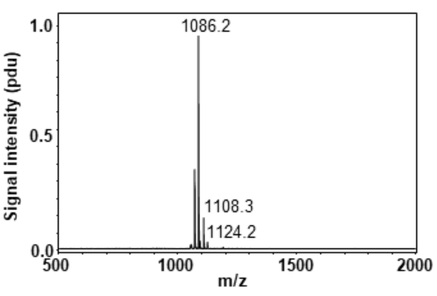

Abz-Gly-Ile-Val-Arg-Ala-Lys(Dnp)-Gly-Hse-NH$_2$ (24)

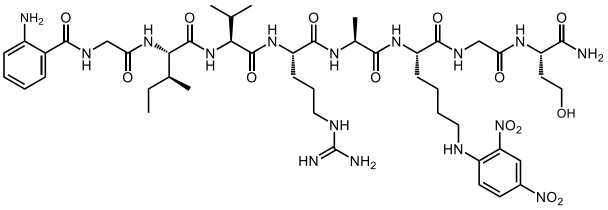

Chemical formula	$C_{47}H_{72}N_{16}O_{14}$
Molecular weight	1085.17 g/mol
Exact mass	1084.54 g/mol
Identified molecular ions (ESI)	*m/z 1085.79 for [M+H]$^+$*<br>*m/z 966.52 for [M-Abz+H]$^+$*<br>*m/z 543.30 for [M+2H]$^{2+}$*
Identified molecular ions (MALDI)	*m/z 1086.2 for [M+H]$^+$*<br>*m/z 1108.3 for [M+Na]$^+$*<br>*m/z 1124.2 for [M+K]$^+$*
Yield	47.7 mg (36 %)
Purity (HPLC, 214 nm)	> 96 %
Chromatogram	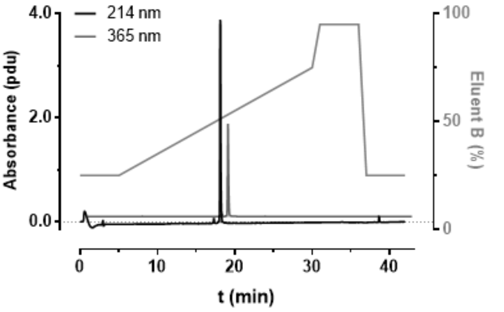
Mass spectrum (ESI)	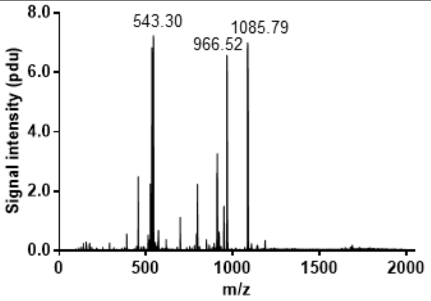
Mass spectrum (MALDI)	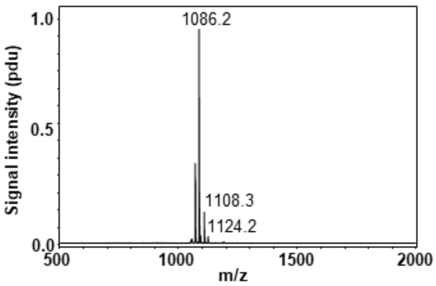

Abz-Gly-Ile-Val-Arg-Ala-Lys(Dnp)-Gly-Cys-NH₂ (25)

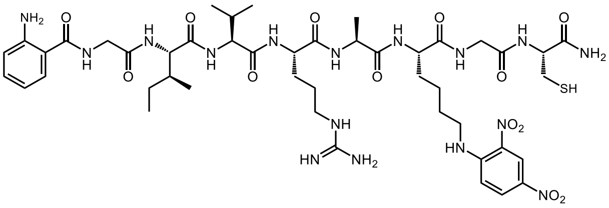

Chemical formula	$C_{46}H_{70}N_{16}O_{13}S$
Molecular weight	1087.21 g/mol
Exact mass	1086.50 g/mol
Identified molecular ions (MALDI)	*m/z 1088.6 for for [M+H]+*<br>*m/z 1110.4 for [M+Na]+*
Yield	18.6 mg (59 % from 31.6 mg crude peptide)
Purity (HPLC, 214 nm)	> 98 %
Chromatogram	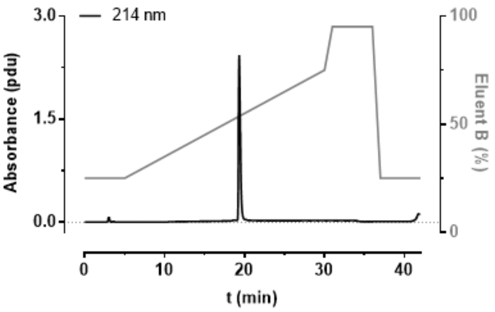
Mass spectrum (MALDI)	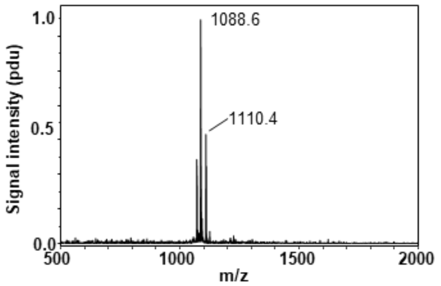

Abz-Gly-Ile-Val-Arg-Ala-Lys(Dnp)-Gly-Met-NH_2 (26)

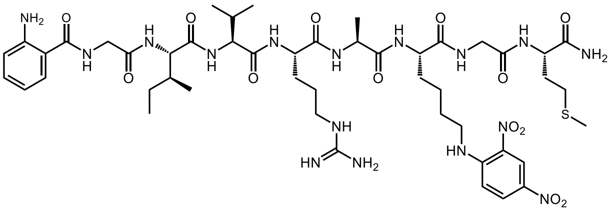

Chemical formula	$C_{48}H_{74}N_{16}O_{13}S$
Molecular weight	1115.27 g/mol
Exact mass	1114.53 g/mol
Identified molecular ions (ESI)	*m/z 1116.13 for $[M+H]^+$*<br>*m/z 996.46 for $[M\text{-}Abz+H]^+$*<br>*m/z 558.31 for $[M+2H]^{2+}$*
Identified molecular ions (MALDI)	*m/z 1116.6 for $[M+H]^+$*<br>*m/z 1138.7 for $[M+Na]^+$*<br>*m/z 1154.7 for $[M+K]^+$*
Yield	68.7 mg (51 %)
Purity (HPLC, 214 nm)	> 98 %
Chromatogram	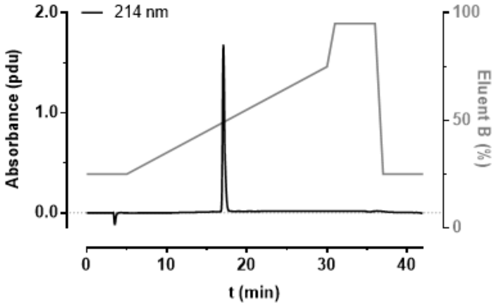
Mass spectrum (ESI)	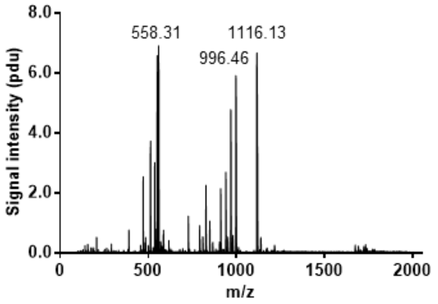
Mass spectrum (MALDI)	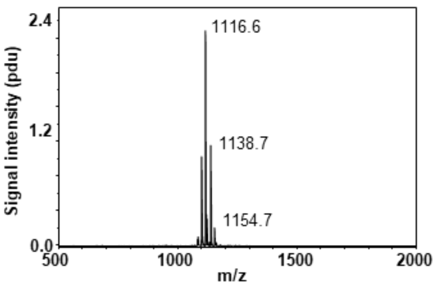

Abz-Gly-Ile-Val-Arg-Ala-Lys(Dnp)-Gly-Asp-NH_2 (27)

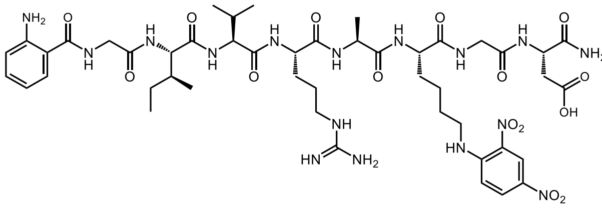

Chemical formula	$C_{47}H_{70}N_{16}O_{15}$
Molecular weight	1099.16 g/mol
Exact mass	1098.52 g/mol
Identified molecular ions (ESI)	*m/z 1099.80 for [M+H]$^+$*<br>*m/z 980.44 for [M-Abz+H]$^+$*<br>*m/z 550.30 for [M+2H]$^{2+}$*
Identified molecular ions (MALDI)	*m/z 1100.6 for [M+H]$^+$*<br>*m/z 1122.5 for [M+Na]$^+$*<br>*m/z 1138.6 for [M+K]$^+$*
Yield	62.4 mg (47 %)
Purity (HPLC, 214 nm)	96.0 %
Chromatogram	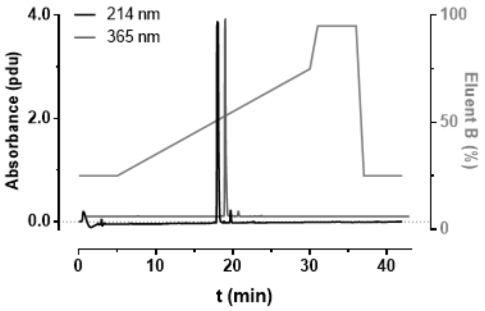
Mass spectrum (ESI)	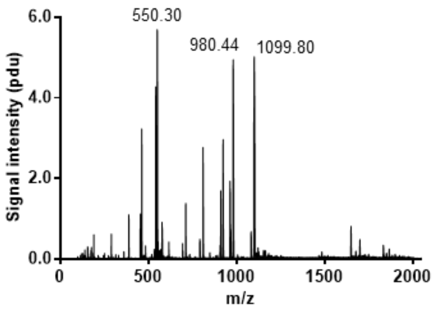
Mass spectrum (MALDI)	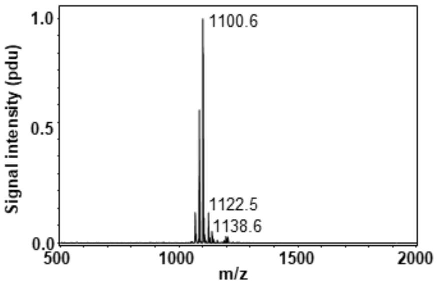

Abz-Gly-Ile-Val-Arg-Ala-Lys(Dnp)-Gly-Glu-NH_2 (28)

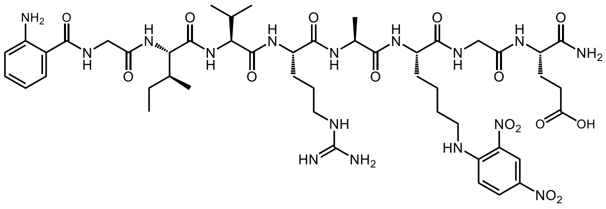

Chemical formula	$C_{48}H_{72}N_{16}O_{15}$
Molecular weight	1113.18 g/mol
Exact mass	1112.54 g/mol
Identified molecular ions (ESI)	*m/z 1114.12 for [M+H]$^+$*<br>*m/z 994.76 for [M-Abz+H]$^+$*<br>*m/z 557.31 for [M+2H]$^{2+}$*
Identified molecular ions (MALDI)	*m/z 1114.6 for [M+H]$^+$*<br>*m/z 1136.5 for [M+Na]$^+$*<br>*m/z 1152.6 for [M+K]$^+$*
Yield	25.6 mg (19 %)
Purity (HPLC, 214 nm)	97.8 %
Chromatogram	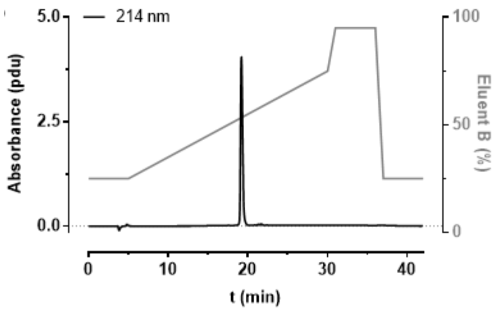
Mass spectrum (ESI)	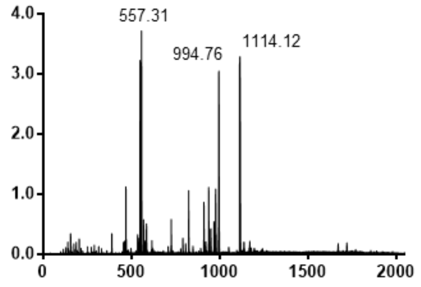
Mass spectrum (MALDI)	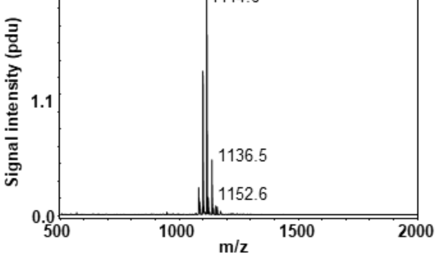

Abz-Gly-Ile-Val-Arg-Ala-Lys(Dnp)-Gly-Asn-NH₂ (29)

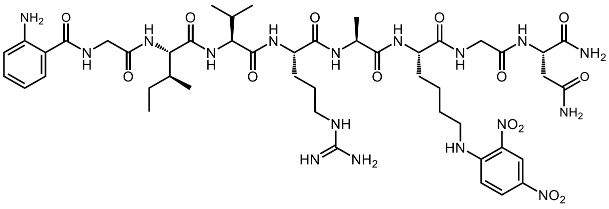

Chemical formula	$C_{47}H_{71}N_{17}O_{14}$
Molecular weight	1098.17 g/mol
Exact mass	1097.54 g/mol
Identified molecular ions (ESI)	*m/z 1098.40 for $[M+H]^+$*<br>*m/z 979.44 for $[M\text{-}Abz+H]^+$*<br>*m/z 549.80 for $[M+2H]^{2+}$*
Identified molecular ions (MALDI)	*m/z 1099.5 for $[M+H]^+$*<br>*m/z 1121.4 for $[M+Na]^+$*<br>*m/z 1137.5 for $[M+K]^+$*
Yield	35.1 mg (27 %)
Purity (HPLC, 214 nm)	> 98 %
Chromatogram	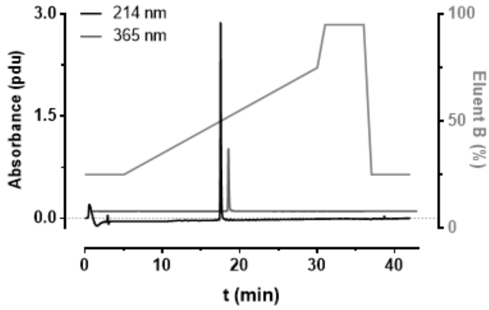
Mass spectrum (ESI)	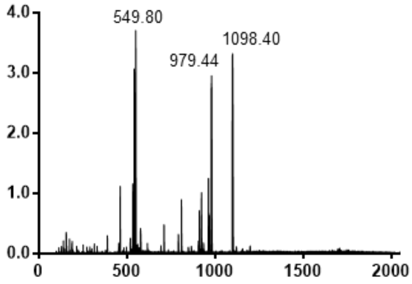
Mass spectrum (MALDI)	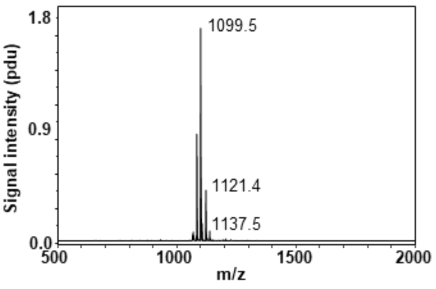

Abz-Gly-Ile-Val-Arg-Ala-Lys(Dnp)-Gly-Gln-NH_2 (30)

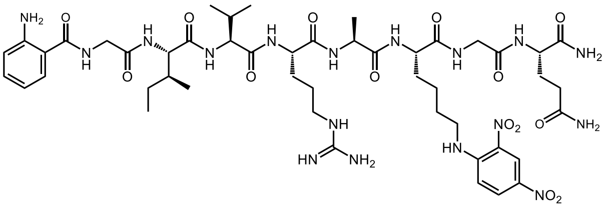

Chemical formula	$C_{48}H_{73}N_{17}O_{14}$
Molecular weight	1112.20 g/mol
Exact mass	1111.55 g/mol
Identified molecular ions (ESI)	*m/z 1112.82 for [M+H]⁺*<br>*m/z 993.46 for [M-Abz+H]⁺*<br>*m/z 556.91 for [M+2H]²⁺*
Identified molecular ions (MALDI)	*m/z 1113.6 for [M+H]⁺*<br>*m/z 1135.6 for [M+Na]⁺*<br>*m/z 1151.7 for [M+K]⁺*
Yield	43.4 mg (32 %)
Purity (HPLC, 214 nm)	> 98 %
Chromatogram	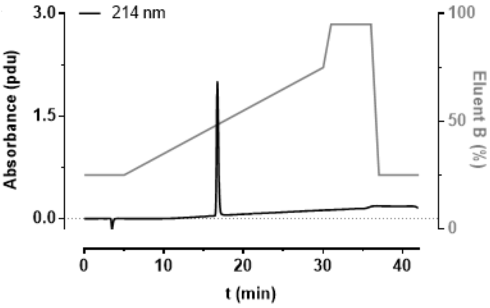
Mass spectrum (ESI)	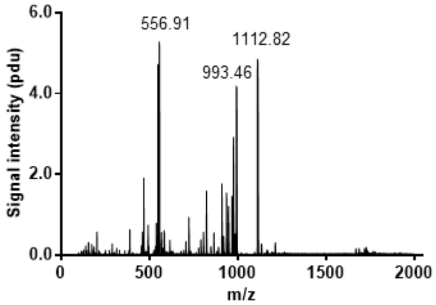
Mass spectrum (MALDI)	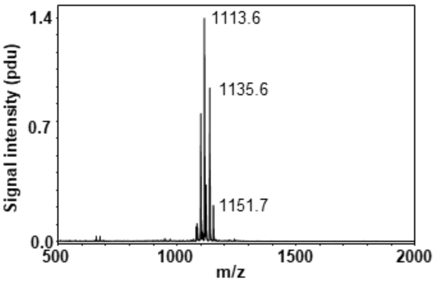

Abz-Gly-Ile-Val-Arg-Ala-Lys(Dnp)-Gly-Orn-NH_2 (31)

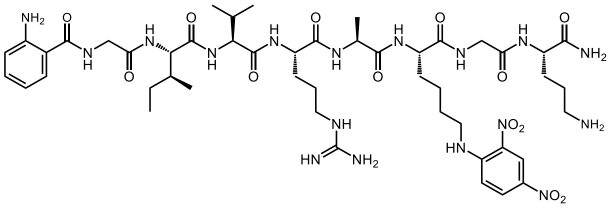

Chemical formula	$C_{48}H_{75}N_{17}O_{13}$
Molecular weight	1098.22 g/mol
Exact mass	1097.57 g/mol
Identified molecular ions (ESI)	*m/z 1099.10 for [M+H]$^+$*<br>*m/z 979.74 for [M-Abz+H]$^+$*<br>*m/z 549.90 for [M+2H]$^{2+}$*
Identified molecular ions (MALDI)	*m/z 1099.6 for [M+H]$^+$*<br>*m/z 1121.5 for [M+Na]$^+$*<br>*m/z 1137.6 for [M+K]$^+$*
Yield	26.8 mg (19 %)
Purity (HPLC, 214 nm)	97.8 %
Chromatogram	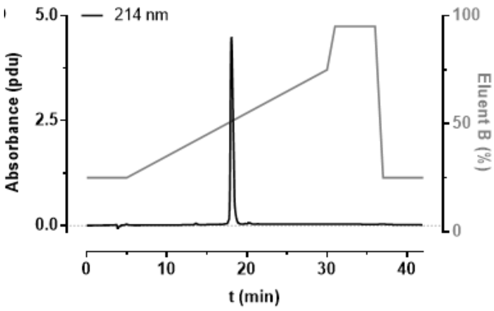
Mass spectrum (ESI)	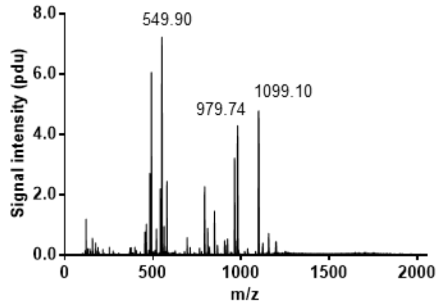
Mass spectrum (MALDI)	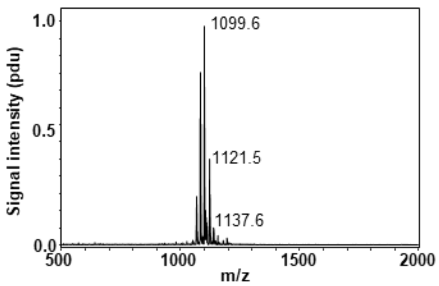

Abz-Gly-Ile-Val-Arg-Ala-Lys(Dnp)-Gly-Lys-NH₂ (32)

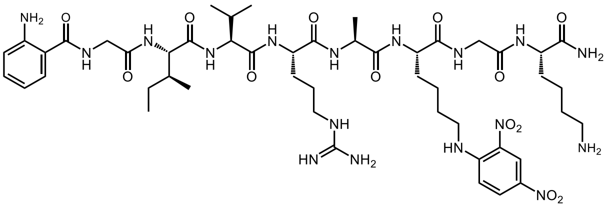

Chemical formula	$C_{49}H_{77}N_{17}O_{13}$
Molecular weight	1112.24 g/mol
Exact mass	1111.59 g/mol
Identified molecular ions (ESI)	*m/z 1112.82 for [M+H]⁺*<br>*m/z 993.46 for [M-Abz+H]⁺*<br>*m/z 556.91 for [M+2H]²⁺*
Identified molecular ions (MALDI)	*m/z 1113.7 for [M+H]⁺*<br>*m/z 1135.8 for [M+Na]⁺*<br>*m/z 1151.7 for [M+K]⁺*
Yield	24.7 mg (17 %)
Purity (HPLC, 214 nm)	96.9 %
Chromatogram	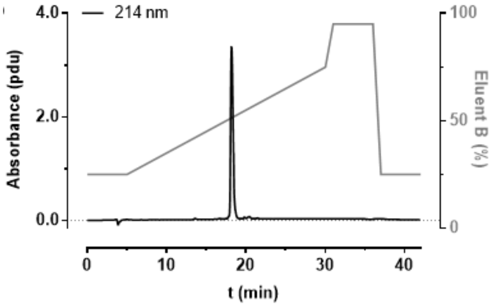
Mass spectrum (ESI)	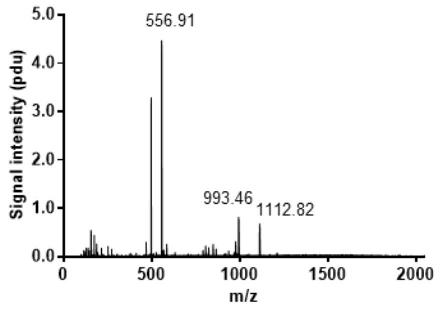
Mass spectrum (MALDI)	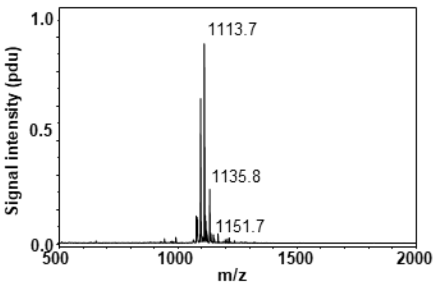

Abz-Gly-Ile-Val-Arg-Ala-Lys(Dnp)-Gly-Arg-NH_2 (33)

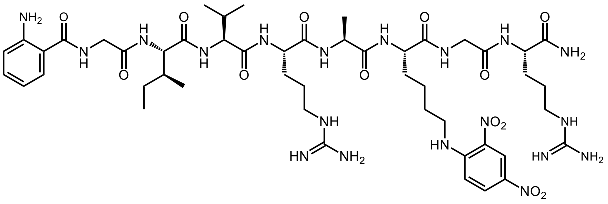

Chemical formula	$C_{49}H_{77}N_{19}O_{13}$
Molecular weight	1140.26 g/mol
Exact mass	1139.59 g/mol
Identified molecular ions (ESI)	*m/z 1140.86 for $[M+H]^+$*<br>*m/z 570.82 for $[M+2H]^{2+}$*
Identified molecular ions (MALDI)	*m/z 1141.7 for $[M+H]^+$*<br>*m/z 1163.6 for $[M+Na]^+$*<br>*m/z 1189.8 for $[M+K]^+$*
Yield	11.5 mg (8 %)
Purity (HPLC, 214 nm)	96.1 %
Chromatogram	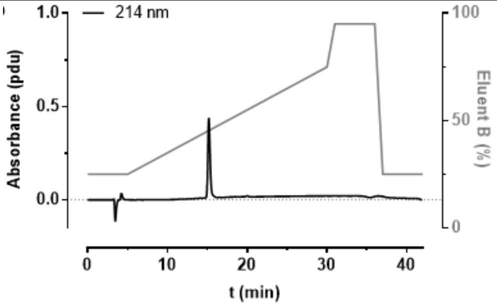
Mass spectrum (ESI)	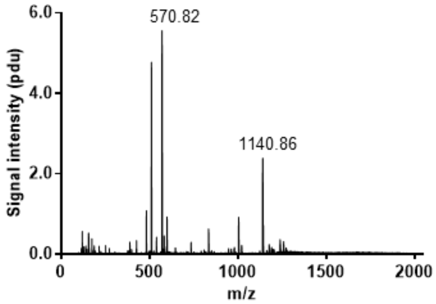
Mass spectrum (MALDI)	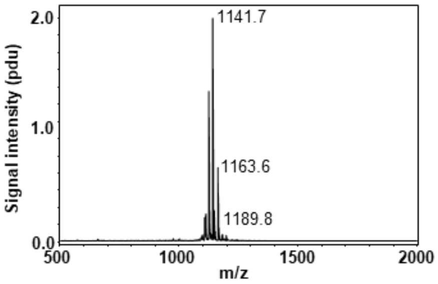

Abz-Gly-Ile-Val-*h*Arg-Ala-Lys(Dnp)-Gly-Ser-NH_2 (34)

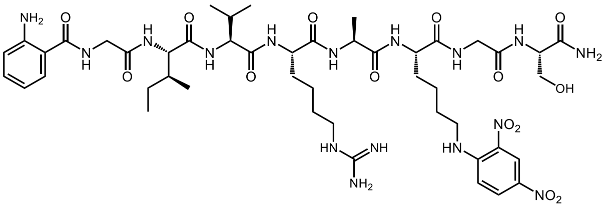

Chemical formula	$C_{47}H_{72}N_{16}O_{14}$
Molecular weight	1085.17 g/mol
Exact mass	1084.54 g/mol
Identified molecular ions (ESI)	*m/z 1085.79 for $[M+H]^+$*<br>*m/z 966.52 for $[M\text{-}Abz+H]^+$*<br>*m/z 543.40 for $[M+2H]^{2+}$*
Identified molecular ions (MALDI)	*m/z 1086.5 for $[M+H]^+$*<br>*m/z 1108.6 for $[M+Na]^+$*<br>*m/z 1124.5 for $[M+K]^+$*
Yield	79.0 mg (60 %)
Purity (HPLC, 214 nm)	> 98 %
Chromatogram	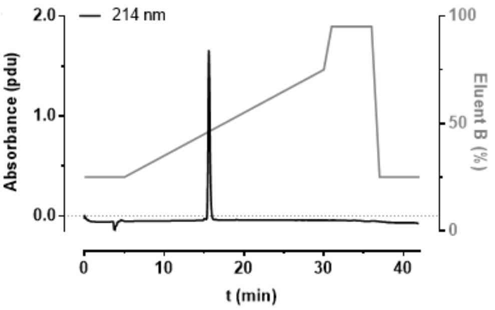
Mass spectrum (ESI)	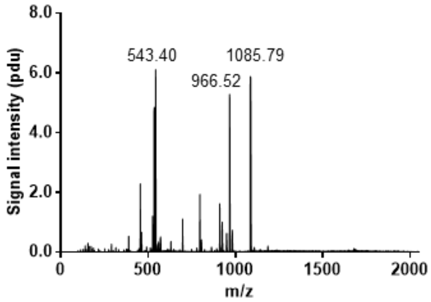
Mass spectrum (MALDI)	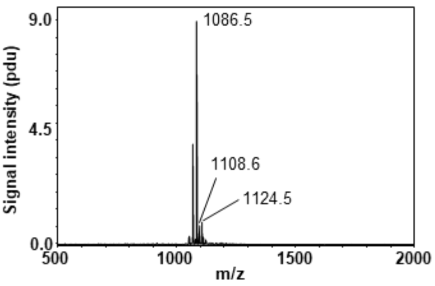

Abz-Gly-Ile-Val-*n*Arg-Ala-Lys(Dnp)-Gly-Ser-NH_2 (35)

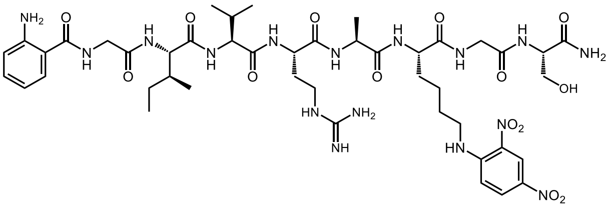

Chemical formula	$C_{45}H_{68}N_{16}O_{14}$
Molecular weight	1057.12 g/mol
Exact mass	1056.51 g/mol
Identified molecular ions (ESI)	*m/z 1057.85 for $[M+H]^+$*<br>*m/z 938.38 for $[M\text{-}Abz+H]^+$*<br>*m/z 529.39 for $[M+2H]^{2+}$*
Identified molecular ions (MALDI)	*m/z 1058.5 for $[M+H]^+$*<br>*m/z 1080.6 for $[M+Na]^+$*<br>*m/z 1096.6 for $[M+K]^+$*
Yield	89.5 mg (70 %)
Purity (HPLC, 214 nm)	> 98 %
Chromatogram	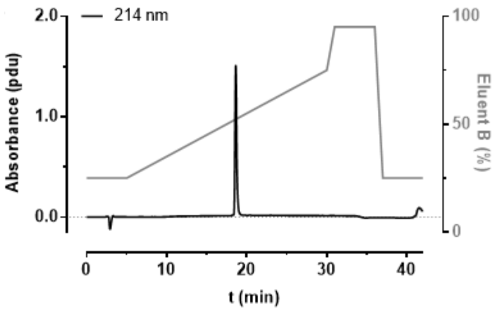
Mass spectrum (ESI)	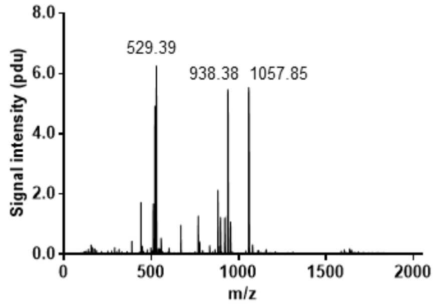
Mass spectrum (MALDI)	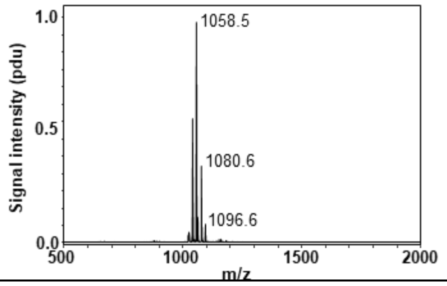

Abz-Gly-Ile-Val-arg-Ala-Lys(Dnp)-Gly-Ser-NH$_2$ (36)

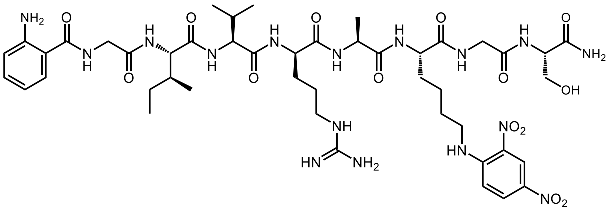

Chemical formula	$C_{47}H_{72}N_{16}O_{14}$
Molecular weight	1085.17 g/mol
Exact mass	1084.54 g/mol
Identified molecular ions (ESI)	*m/z 1085.79 for [M+H]$^+$*<br>*m/z 966.52 for [M-Abz+H]$^+$*<br>*m/z 543.40 for [M+2H]$^{2+}$*
Identified molecular ions (MALDI)	*m/z 1086.5 for [M+H]$^+$*<br>*m/z 1108.6 for [M+Na]$^+$*<br>*m/z 1124.5 for [M+K]$^+$*
Yield	79.0 mg (60 %)
Purity (HPLC, 214 nm)	> 98 %
Chromatogram	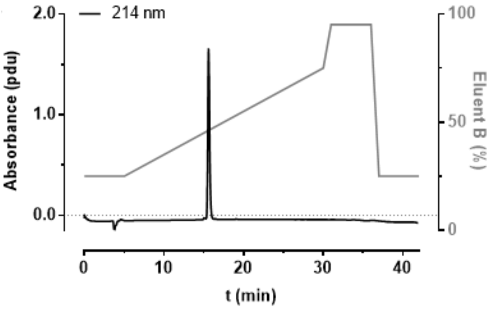
Mass spectrum (ESI)	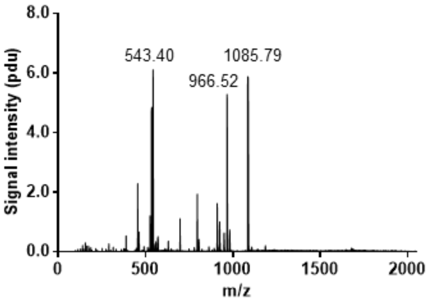
Mass spectrum (MALDI)	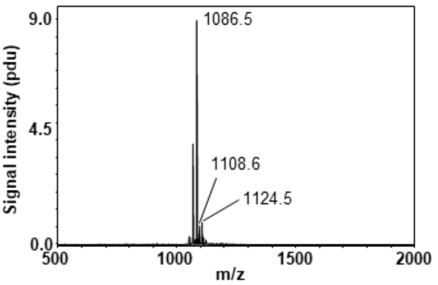

Abz-Gly-Ile-Val-Cit-Ala-Lys(Dnp)-Gly-Ser-NH$_2$ (37)

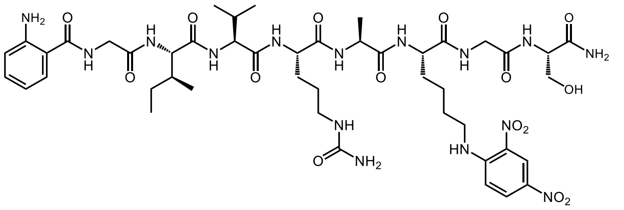

Chemical formula	$C_{46}H_{69}N_{15}O_{15}$
Exact mass	1071.51 g/mol
Identified molecular ions (ESI)	*m/z 1094.41 for [M+Na]$^+$*
Yield	26.0 mg (20 %)
t_R	21.0 min
Purity (HPLC, 214 nm)	>98 %

Abz-Gly-Phe-Leu-Gly-Ala-Lys(Dnp)-Gly-Ser-NH_2 (38)

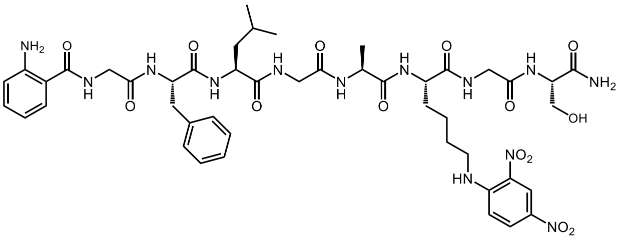

Chemical formula	$C_{46}H_{74}N_{17}O_{14}$
Molecular weight	1020.06 g/mol
Exact mass	1019.45 g/mol
Identified molecular ions (ESI)	*m/z 2039.97 for $[2M+H]^+$*<br>*m/z 1020.40 for $[M+H]^+$*<br>*m/z 901.33 for $[M-Abz+H]^+$*
Identified molecular ions (MALDI)	*m/z 1020.2 for $[M+H]^+$*<br>*m/z 1043.4 for $[M+Na]^+$*<br>*m/z 1059.5 for $[M+K]^+$*
Yield	12.9 mg (11 %)
Purity (HPLC, 214 nm)	> 98 %
Chromatogram	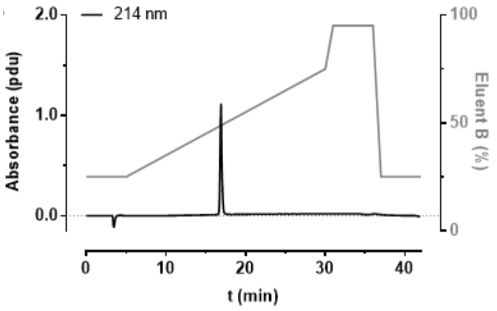
Mass spectrum (ESI)	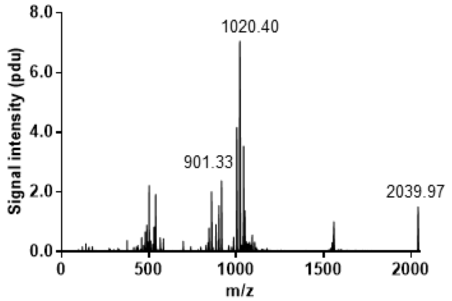
Mass spectrum (MALDI)	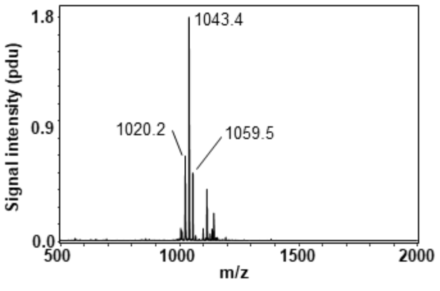

Abz-Gly-Ile-Val-Arg-Ala-Lys(Dnp)-Sar-Ser-NH$_2$ (39)

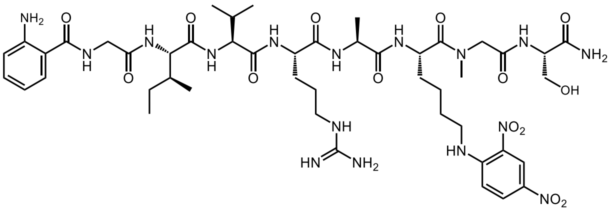

Chemical formula	$C_{47}H_{72}N_{16}O_{14}$
Molecular weight	1085.17 g/mol
Exact mass	1084.54 g/mol
Identified molecular ions (ESI)	*m/z 1086.19 for [M+H]$^+$*<br>*m/z 966.42 for [M-Abz+H]$^+$*<br>*m/z 543.40 for [M+2H]$^{2+}$*
Identified molecular ions (MALDI)	*m/z 1086.4 for [M+H]$^+$*<br>*m/z 1108.5 for [M+Na]$^+$*
Yield	55.6 mg (42 %)
Purity (HPLC, 214 nm)	> 98 %
Chromatogram	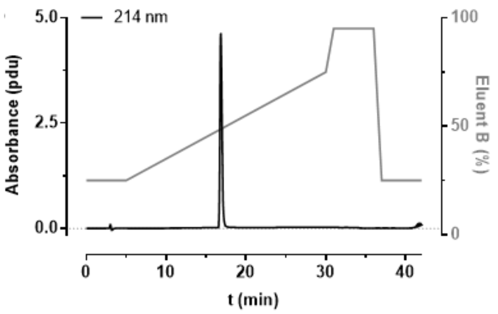
Mass spectrum (ESI)	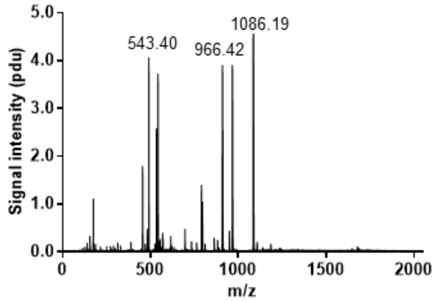
Mass spectrum (MALDI)	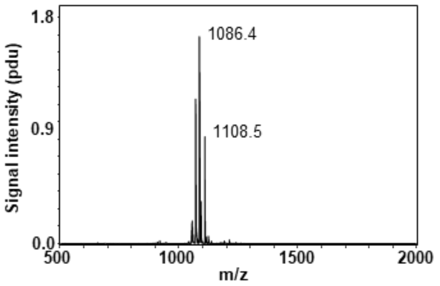

Abz-Gly-Ile-Val-Arg(ec)-Ala-Lys(Dnp)-Sar-Ser-NH_2 (40)

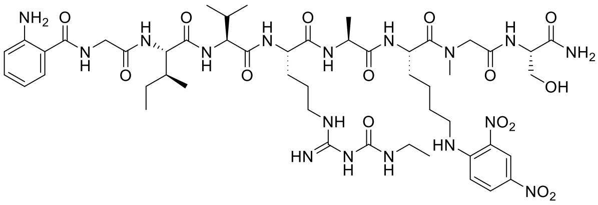

Chemical formula	$C_{50}H_{77}N_{17}O_{15}$
Molecular weight	1156.25 g/mol
Exact mass	1155.58 g/mol
Identified molecular ions (ESI)	*m/z 1178.57 for [M+Na]$^+$*<br>*m/z 1156.38 for [M+H]$^+$*<br>*m/z 1037.32 for [M-Abz+H]$^+$*<br>*m/z 578.93 for [M+2H]$^{2+}$*
Yield	24.5 mg (35 %)
Purity (HPLC, 214 nm)	97.3 %
Chromatogram	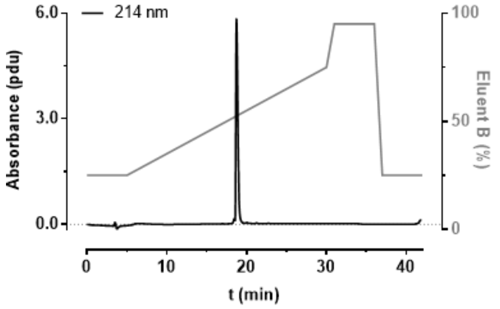
Mass spectrum (ESI)	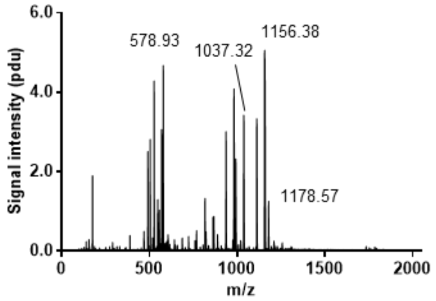

Abz-Gly-Phe-Leu-Gly-Ala-Lys(Dnp)-Sar-Ser-NH_2 (41)

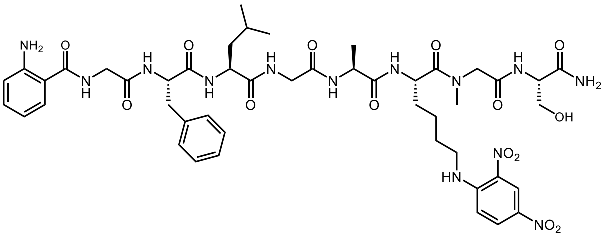

Chemical formula	$C_{47}H_{63}N_{13}O_{14}$
Molecular weight	1034.08 g/mol
Exact mass	1033.46 g/mol
Identified molecular ions (ESI)	*m/z 1056.53 for [M+Na]⁺*<br>*m/z 1034.42 for [M+H]⁺*<br>*m/z 912.25 for [M-Abz+H]⁺*
Identified molecular ions (MALDI)	*m/z 1035.2 for [M+H]⁺*<br>*m/z 1057.1 for [M+Na]⁺*<br>*m/z 1073.2 for [M+K]⁺*
Yield	79.3 mg (69 %)
Purity (HPLC, 214 nm)	> 98 %
Chromatogram	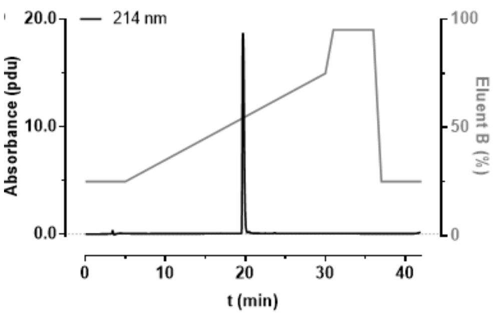
Mass spectrum (ESI)	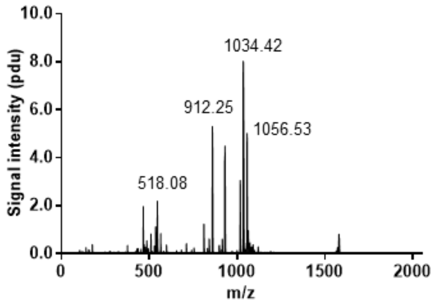
Mass spectrum (MALDI)	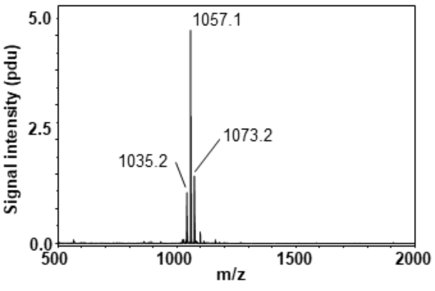

Abz-Gly-Phe-Leu-Gly-Ala-Lys(Dnp)-Sar-Val-NH$_2$ (42)

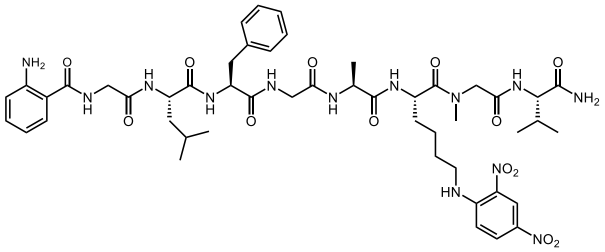

Chemical formula	$C_{49}H_{67}N_{13}O_{13}$
Molecular weight	1046.14 g/mol
Exact mass	1045.50 g/mol
Identified molecular ions (ESI)	*m/z 1068.74 for [M+Na]$^+$*<br>*m/z 1046.63 for [M+H]$^+$*
Identified molecular ions (MALDI)	*m/z 1047.4 fr [M+H]$^+$*<br>*m/z 1069.5 for [M+Na]$^+$*<br>*m/z 1085.4 for [M+K]$^+$*
Yield	23.3 mg (20 %)
Purity (HPLC, 214 nm)	> 98 %
Chromatogram	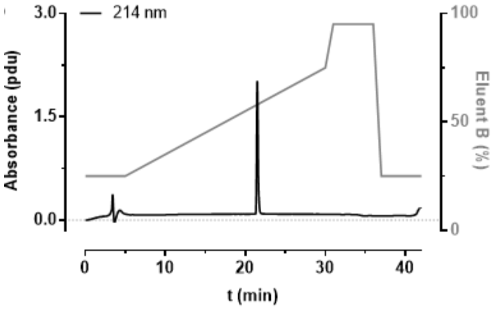
Mass spectrum (ESI)	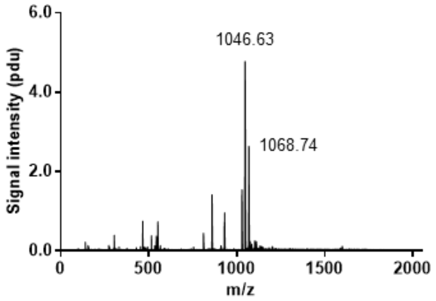
Mass spectrum (MALDI)	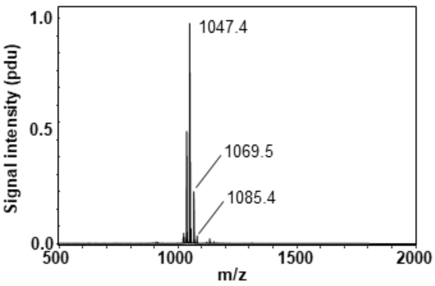

Abz-Gly-Phe-Leu-Gly-Ala-Lys(Dnp)-Sar-Gly-NH_2 (43)

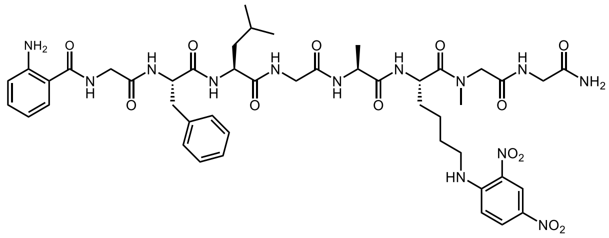

Chemical formula	$C_{46}H_{61}N_{13}O_{13}$
Molecular weight	1004.06 g/mol
Exact mass	1003.45 g/mol
Identified molecular ions (ESI)	*m/z 2007.89 for $[2M+H]^+$*<br>*m/z 1026.71 for $[M+Na]^+$*<br>*m/z 1004.48 for $[M+H]^+$*<br>*m/z 885.41 for $[M\text{-}Abz+H]^+$*<br>*m/z 502.77 for $[M+2H]^{2+}$*
Identified molecular ions (MALDI)	*m/z 1005.3 for $[M+H]^+$*<br>*m/z 1027.2 for $[M+Na]^+$*<br>*m/z 1043.2 for $[M+K]^+$*
Yield	27.0 mg (89 % from 30.5 mg crude peptide)
Purity (HPLC, 214 nm)	> 98 %
Chromatogram	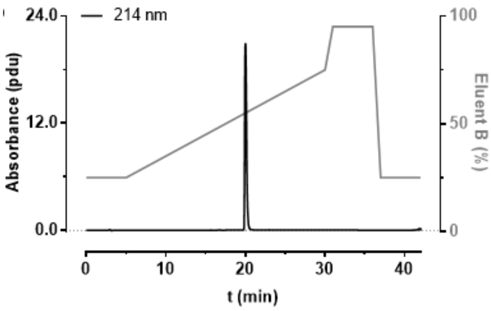
Mass spectrum (ESI)	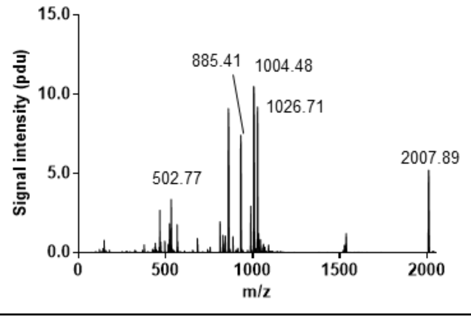
Mass spectrum (MALDI)	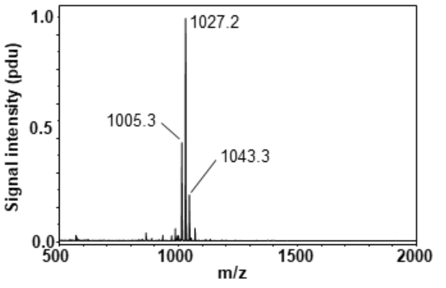

Abz-Gly-Phe-Leu-Gly-Ala-Lys(Dnp)-Sar-Ala-NH$_2$ (44)

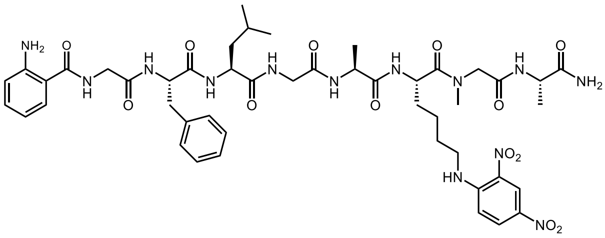

Chemical formula	$C_{47}H_{63}N_{13}O_{13}$
Molecular weight	1018.08 g/mol
Exact mass	1017.47 g/mol
Identified molecular ions (ESI)	*m/z 2035.66 for [2M+H]$^+$*<br>*m/z 1040.32 for [M+Na]$^+$*<br>*m/z 1018.29 for [M+H]$^+$*<br>*m/z 899.33 for [M-Abz+H]$^+$*
Identified molecular ions (MALDI)	*m/z 1019.2 for [M+H]$^+$*<br>*m/z 1040.9 for [M+Na]$^+$*<br>*m/z 1057.3 for [M+K]$^+$*
Yield	27.0 mg (91 % from 29.7 mg crude peptide)
Purity (HPLC, 214 nm)	> 98 %
Chromatogram	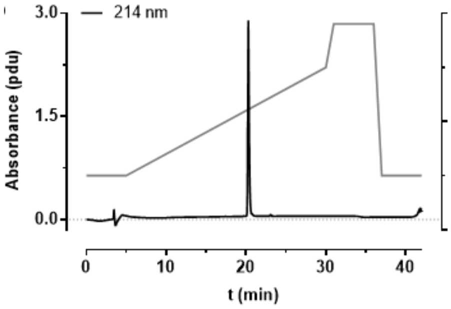
Mass spectrum (ESI)	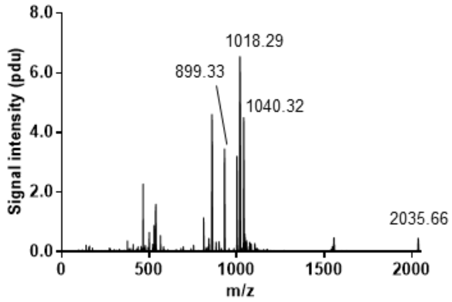
Mass spectrum (MALDI)	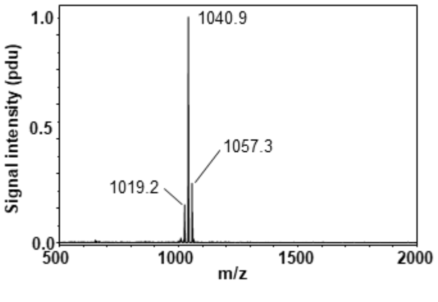

Abz-Gly-Phe-Leu-Gly-Ala-Lys(Dnp)-Sar-Leu-NH$_2$ (45)

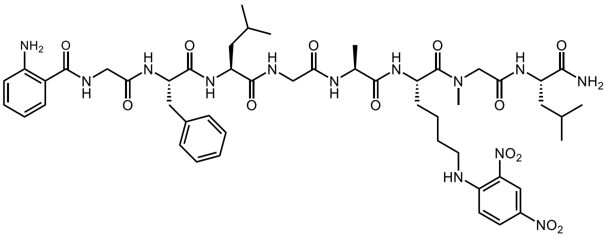

Chemical formula	$C_{50}H_{69}N_{13}O_{13}$
Molecular weight	1060.16 g/mol
Exact mass	1059.61 g/mol
Identified molecular ions (ESI)	*m/z 1082.48 for [M+Na]⁺*<br>*m/z 1060.35 for [M+H]⁺*<br>*m/z 930.17 for [M-Abz+H]⁺*
Identified molecular ions (MALDI)	*m/z 1061.2 for [M+H]⁺*<br>*m/z 1083.6 for [M+Na]⁺*<br>*m/z 1099.4 for [M+K]⁺*
Yield	23.0 mg (82 % from 29.0 mg crude peptide)
Purity (HPLC, 214 nm)	> 98 %
Chromatogram	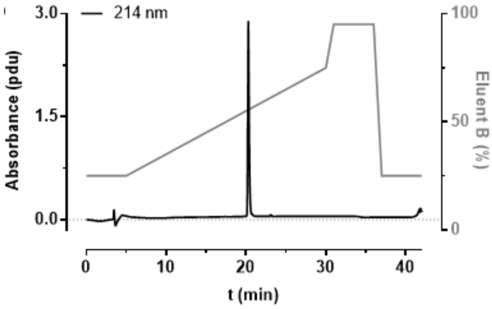
Mass spectrum (ESI)	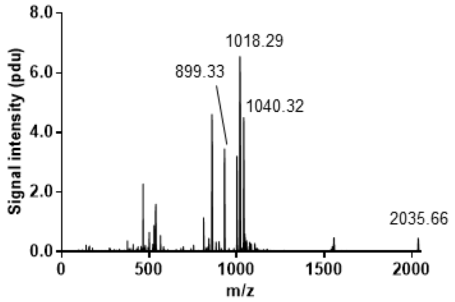
Mass spectrum (MALDI)	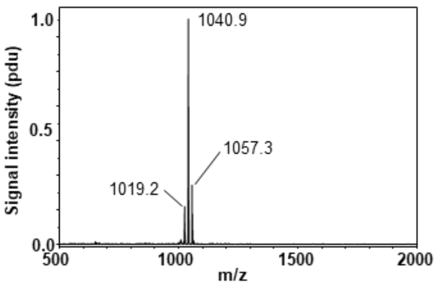

Abz-Gly-Phe-Leu-Gly-Ala-Lys(Dnp)-Sar-Phe-NH_2 (46)

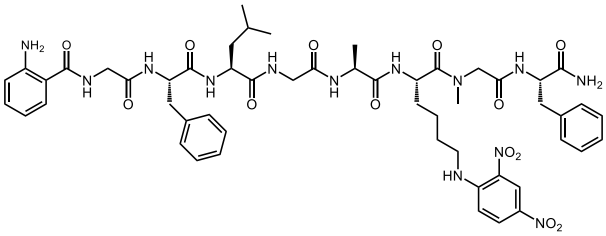

Chemical formula	$C_{53}H_{67}N_{13}O_{13}$
Molecular weight	1094.18 g/mol
Exact mass	1093.50 g/mol
Identified molecular ions (ESI)	*m/ 1116.74 for [M+Na]$^+$*<br>*m/z 1094.30 for [M+H]$^+$*<br>*m/z 975.30 for [M-Abz+H]$^+$*
Identified molecular ions (MALDI)	*m/z 1095.3 for [M+H]$^+$*<br>*m/z 1117.2 for [M+Na]$^+$*<br>*m/z 1133.2 for [M+K]$^+$*
Yield	23.8 mg (82 % from 29.2 mg crude peptide)
Purity (HPLC, 214 nm)	97.8 %
Chromatogram	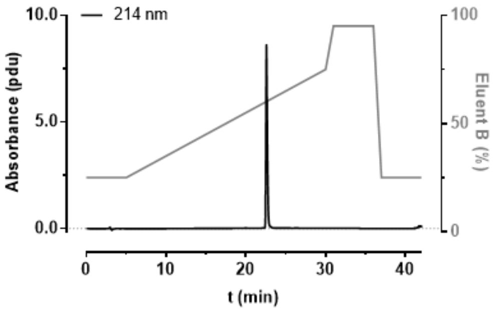
Mass spectrum (ESI)	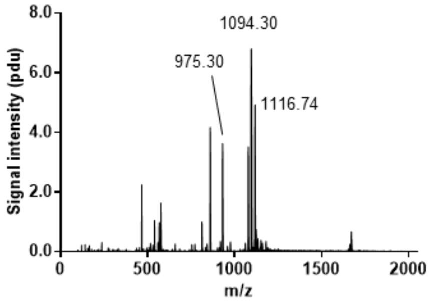
Mass spectrum (MALDI)	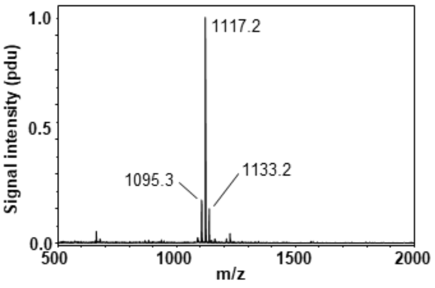

Abz-Gly-Phe-Leu-Gly-Ala-Lys(Dnp)-Sar-Tyr-NH$_2$ (47)

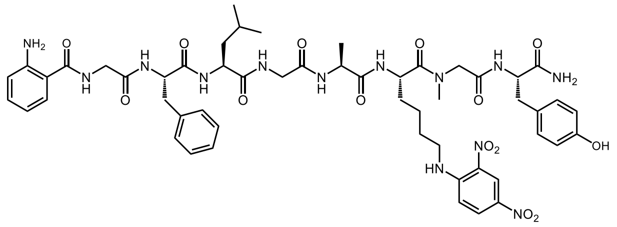

Chemical formula	$C_{53}H_{67}N_{13}O_{14}$
Molecular weight	1110.18 g/mol
Exact mass	1109.49 g/mol
Identified molecular ions (ESI)	*m/z 1133.05 for [M+Na]$^+$*<br>*m/z 1111.12 for [M+H]$^+$*<br>*m/z 991.76 for [M-Abz+H]$^+$*<br>*m/z 555.91 for [M+2H]$^{2+}$*
Identified molecular ions (MALDI)	*m/z 1111.3 for [M+H]$^+$*<br>*m/z 1133.5 for [M+Na]$^+$*<br>*m/z 1149.2 for [M+K]$^+$*
Yield	16.0 mg (83 % from 31.5 mg crude peptide)
Purity (HPLC, 214 nm)	95.4 %
Chromatogram	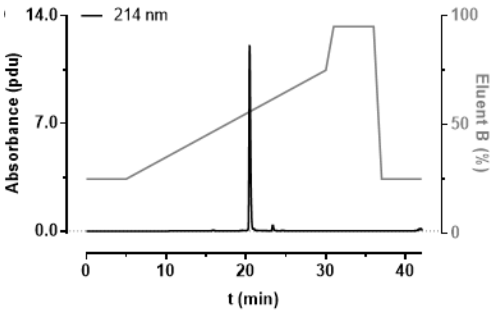
Mass spectrum (ESI)	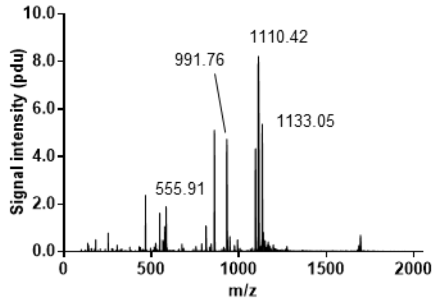
Mass spectrum (MALDI)	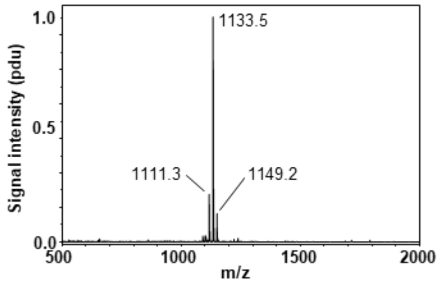

Abz-Gly-Phe-Leu-Gly-Ala-Lys(Dnp)-Sar-Glu-NH$_2$ (48)

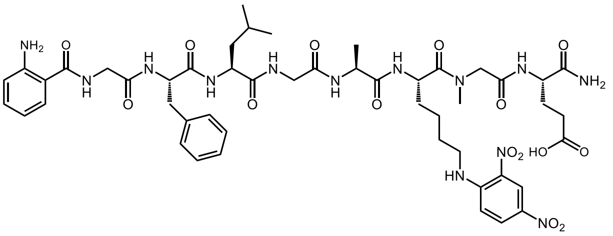

Chemical formula	$C_{49}H_{65}N_{13}O_{15}$
Molecular weight	1076.12 g/mol
Exact mass	1075.47 g/mol
Identified molecular ions (ESI)	*m/z 1098.79 for [M+Na]$^+$*<br>*m/z 1076.77 for [M+H]$^+$*
Yield	24.0 mg (77 % from 31.0 mg crude peptide)
Purity (HPLC, 214 nm)	> 98 %
Chromatogram	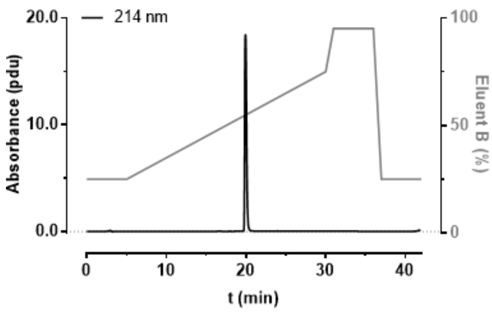
Mass spectrum (ESI)	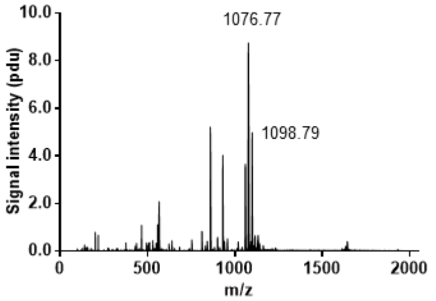

Abz-Gly-Phe-Leu-Gly-Ala-Lys(Dnp)-Sar-Gln-NH$_2$ (49)

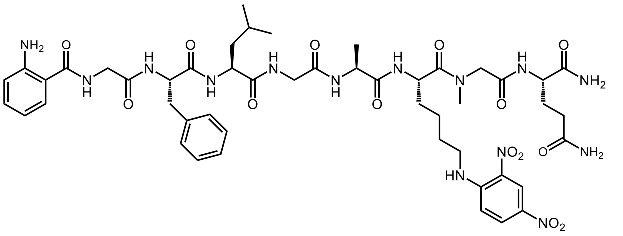

Chemical formula	$C_{49}H_{66}N_{14}O_{14}$
Molecular weight	1075.13 g/mol
Exact mass	1074.49 g/mol
Identified molecular ions (ESI)	*m/z 1097.70 for [M+Na]$^+$*<br>*m/z 1075.87 for [M+H]$^+$*<br>*m/z 956.41 for [M-Abz+H]$^+$*<br>*m/z 538.39 for [M+2H]$^{2+}$*
Identified molecular ions (MALDI)	*m/z 1076.3 for [M+H]$^+$*<br>*m/z 1098.6 for [M+Na]$^+$*<br>*m/z 1114.3 for [M+K]$^+$*
Yield	29.1 mg (96 % from 30.3 mg crude peptide)
Purity (HPLC, 214 nm)	> 98 %
Chromatogram	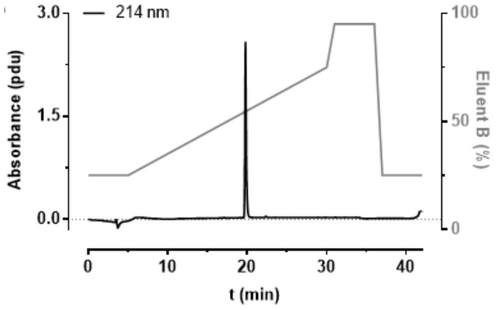
Mass spectrum (ESI)	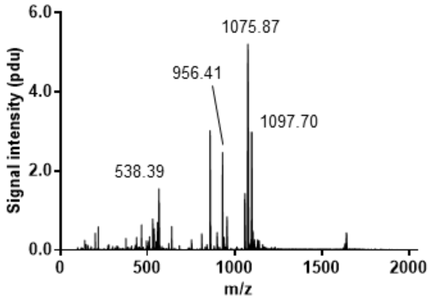
Mass spectrum (MALDI)	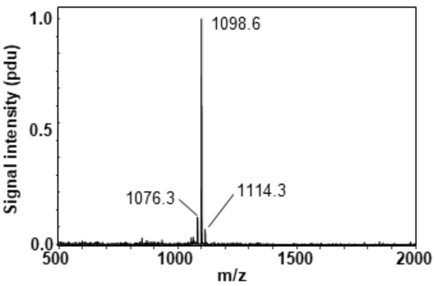

Abz-Gly-Phe-Leu-Gly-Ala-Lys(Dnp)-Sar-Orn-NH$_2$ (50)

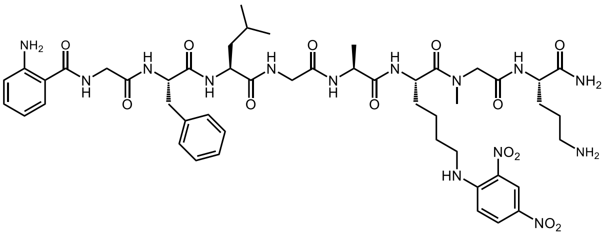

Chemical formula	$C_{49}H_{68}N_{14}O_{13}$
Molecular weight	1061.15 g/mol
Exact mass	1060.51 g/mol
Identified molecular ions (ESI)	*m/z 1083.48 for [M+Na]$^+$*<br>*m/z 1061.45 for [M+H]$^+$*<br>*m/z 942.39 for [M-Abz+H]$^+$*<br>*m/z 531.39 for [M+2H]$^{2+}$*
Identified molecular ions (MALDI)	*m/z 1062.2 for [M+H]$^+$*<br>*m/z 1084.5 for [M+Na]$^+$*<br>*m/z 1100.2 for [M+K]$^+$*
Yield	27.3 mg (80 % from 34.0 mg crude peptide)
Purity (HPLC, 214 nm)	96.3 %
Chromatogram	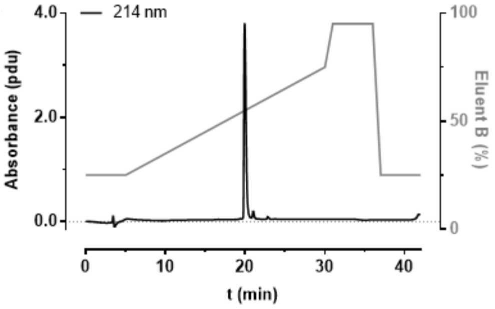
Mass spectrum (ESI)	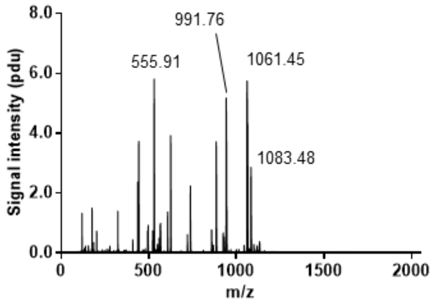
Mass spectrum (MALDI)	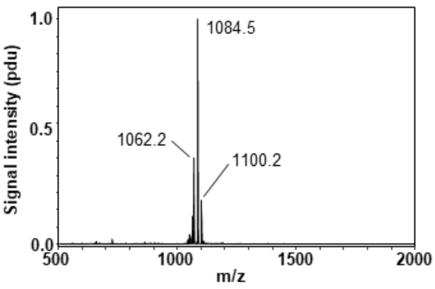

Abz-Gly-Phe-Leu-Gly-Ala-Lys(Dnp)-Sar-Lys-NH_2 (51)

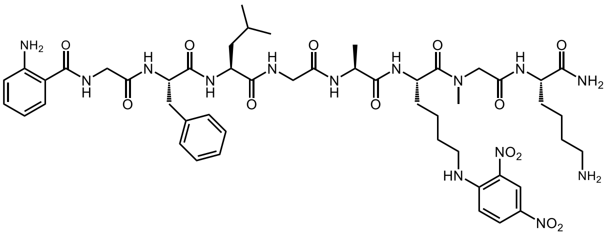

<table>
<tr><td>Chemical formula</td><td>$C_{50}H_{70}N_{14}O_{13}$</td></tr>
<tr><td>Molecular weight</td><td>1075.18 g/mol</td></tr>
<tr><td>Exact mass</td><td>1074.52 g/mol</td></tr>
<tr><td>Identified molecular ions (ESI)</td><td>m/z 1097.84 for [M+Na]$^+$
m/z 1075.77 for [M+H]$^+$
m/z 956.27 for [M-Abz+H]$^+$
m/z 538.39 for [M+2H]$^{2+}$</td></tr>
<tr><td>Identified molecular ions (MALDI)</td><td>m/z 1076.3 for [M+H]$^+$
m/z 1098.6 for [M+Na]$^+$
m/z 1114.3 for [M+K]$^+$</td></tr>
<tr><td>Yield</td><td>23.9 mg (81 % from 29.7 mg crude peptide)</td></tr>
<tr><td>Purity (HPLC, 214 nm)</td><td>> 98 %</td></tr>
<tr><td>Chromatogram</td><td>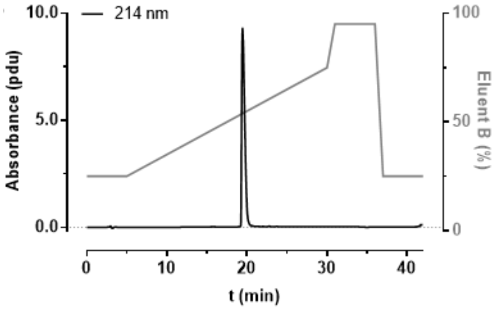
</td></tr>
<tr><td>Mass spectrum (ESI)</td><td>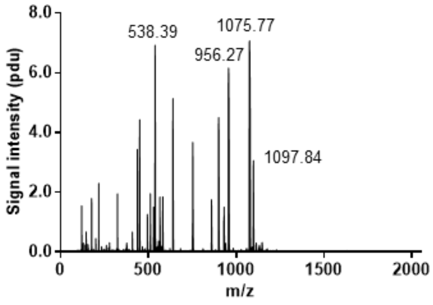
</td></tr>
<tr><td>Mass spectrum (MALDI)</td><td>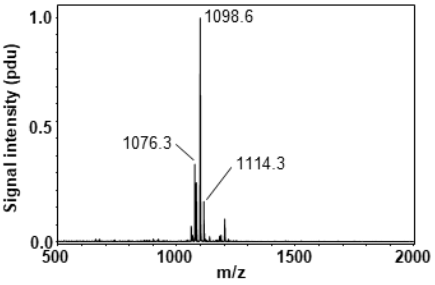
</td></tr>
</table>

Abz-Gly-Phe-ACBC-Gly-Ala-Lys(Dnp)-Sar-Val-NH$_2$ (52)

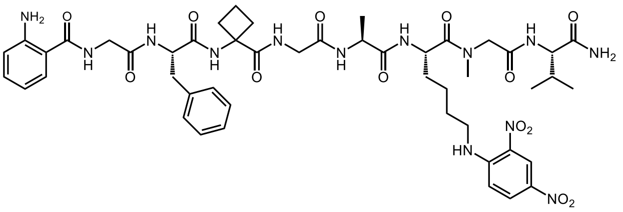

Chemical formula	$C_{48}H_{63}N_{13}O_{13}$
Molecular weight	1030.11
Exact mass	1029.4668
Identified molecular ions (HR-ESI)	*1030.4738 for [M+H]$^+$ (calcd. 1030.4741)*<br>*1052.4553 for [M+Na]$^+$ (calcd. 1052.4560)*
Yield	21.8 mg
Purity (HPLC, 214 nm)	97.2 %
Chromatogram	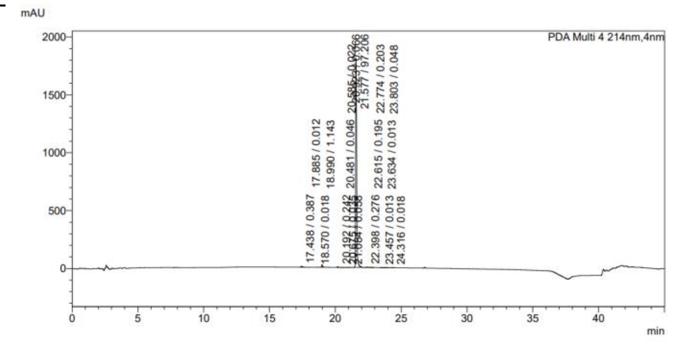
Mass spectrum (HR-ESI)	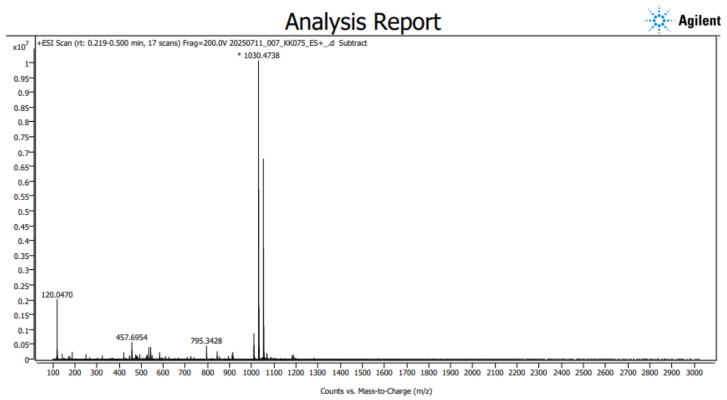

Abz-Gly-Leu-Phe-Gly-Ala-Lys(Dnp)-Sar-Val-NH$_2$ (53)

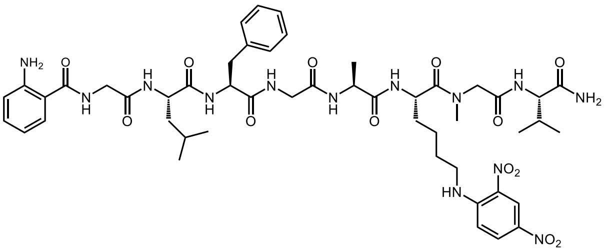

Chemical formula	$C_{49}H_{67}N_{13}O_{13}$
Molecular weight	1046.14 g/mol
Exact mass	1045.50 g/mol
Identified molecular ions (ESI)	*m/z 1068.76 for [M+Na]⁺*<br>*m/z 1046.53 for [M+H]⁺*
Identified molecular ions (MALDI)	*m/z 1047.3 for [M+H]⁺*<br>*m/z 1069.2 for [M+Na]⁺*<br>*m/z 1085.2 for [M+K]⁺*
Yield	22.9 mg (79 % from 29.0 mg crude peptide)
Purity (HPLC, 214 nm)	> 98 %
Chromatogram	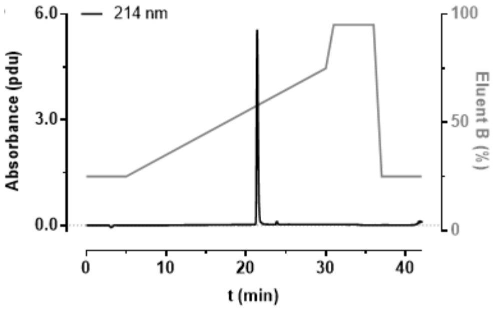
Mass spectrum (ESI)	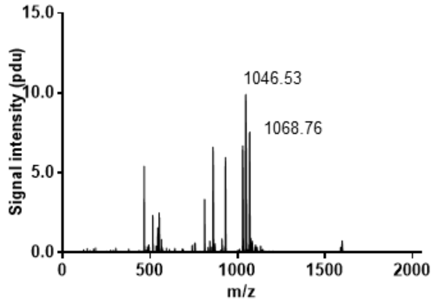
Mass spectrum (MALDI)	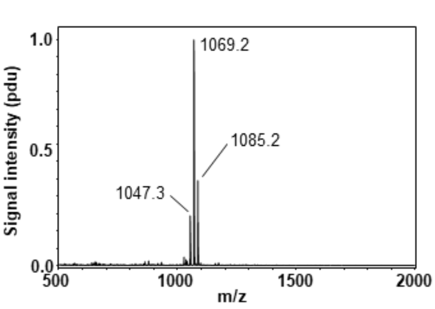

Abz-Gly-Leu-Phe(3-Me)-Gly-Ala-Lys(Dnp)-Sar-Val-NH_2 (54)

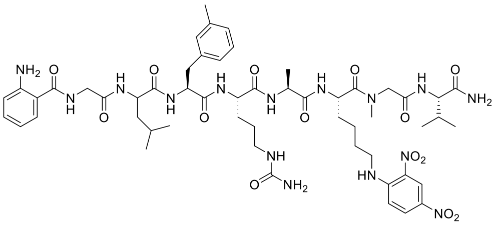

Chemical formula	$C_{50}H_{69}N_{13}O_{13}$<br>23.3 mg (79 % from 30.2 mg crude peptide)<br>> 98 %
Molecular weight	1060.16 g/mol
Exact mass	1059.51 g/mol
Identified molecular ions (ESI)	*m/z 1082.78 for [M+Na]$^+$*<br>*m/z 1060.75 for [M+H]$^+$*
Identified molecular ions (MALDI)	*m/z 1061.2 for [M+H]$^+$*<br>*m/z 1083.6 for [M+Na]$^+$*<br>*m/z 1099.3 for [M+K]$^+$*
Yield	23.3 mg (79 % from 30.2 mg crude peptide)
Purity (HPLC, 214 nm)	> 98 %
Chromatogram	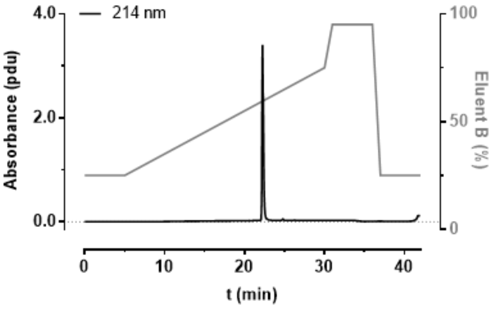
Mass spectrum (ESI)	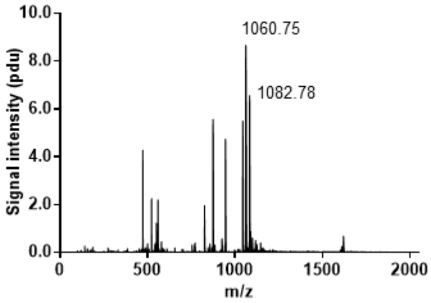
Mass spectrum (MALDI)	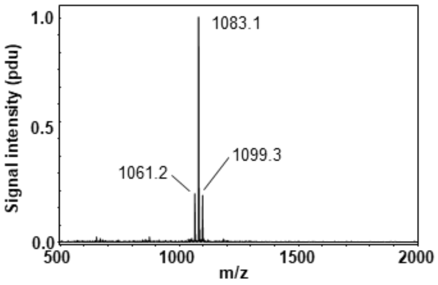

Abz-Gly-Leu-Phe(3-I)-Gly-Ala-Lys(Dnp)-Sar-Val-NH$_2$ (55)

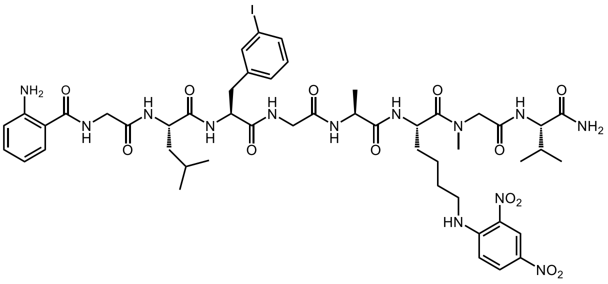

Chemical formula	$C_{49}H_{66}IN_{13}O_{13}$
Molecular weight	1172.03 g/mol
Exact mass	1171.39 g/mol
Identified molecular ions (ESI)	*m/z 1194.64 for [M+Na]$^+$*<br>*m/z 1172.70 for [M+H]$^+$*
Yield	2.3 mg (7 % from 31.2 mg crude peptide)
Purity (HPLC, 214 nm)	> 98 %
Chromatogram	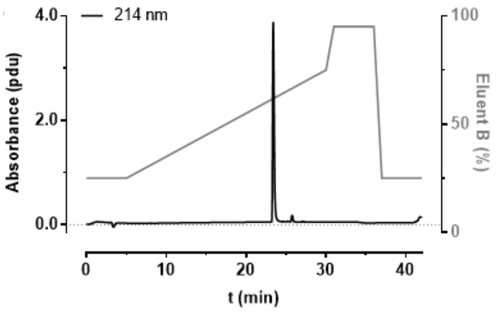
Mass spectrum (ESI)	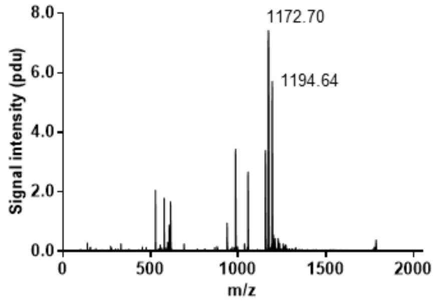

Abz-Gly-Phe-Leu-Cit-Ala-Lys(Dnp)-Sar-Val-NH$_2$ (56)

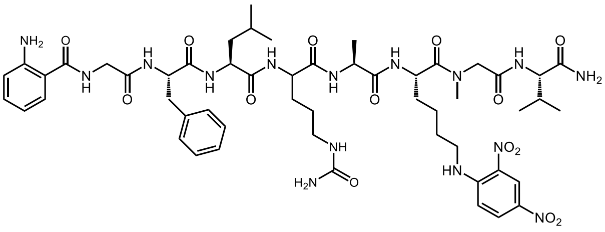

Chemical formula	$C_{53}H_{75}N_{15}O_{14}$
Molecular weight	1146.25 g/mol
Exact mass	1145.56 g/mol
Identified molecular ions (ESI)	*m/z 1168.79 for [M+Na]$^+$*<br>*m/z 1146.86 for [M+H]$^+$*<br>*m/z 1027.81 for [M-Abz+H]$^+$*<br>*m/z 573.92 for [M+2H]$^{2+}$*
Identified molecular ions (MALDI)	*m/z 1147.3 for [M+H]$^+$*<br>*m/z 1169.5 for [M+Na]$^+$*<br>*m/z 1185.3 for [M+K]$^+$*
Yield	15.9 mg (50 % from 32.1 mg crude peptide)
Purity (HPLC, 214 nm)	> 98 %
Chromatogram	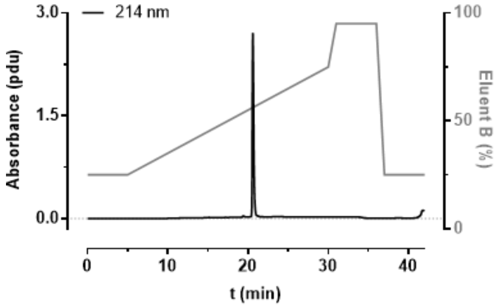
Mass spectrum (ESI)	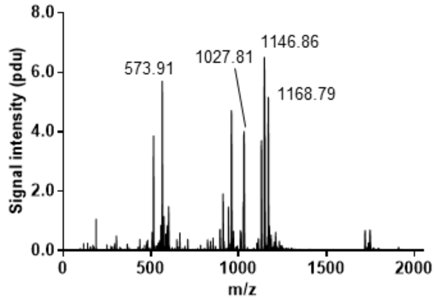
Mass spectrum (MALDI)	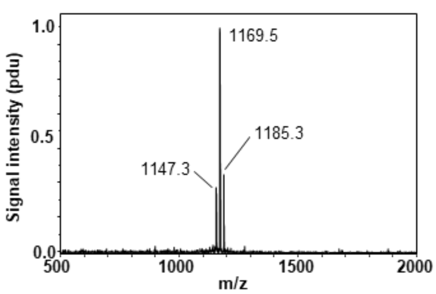

Abz-Gly-Phe-Phe-Cit-Ala-Lys(Dnp)-Sar-Val-NH$_2$ (57)

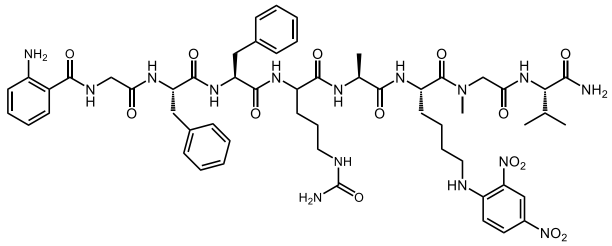

Chemical formula	$C_{56}H_{73}N_{15}O_{14}$
Molecular weight	1180.27 g/mol
Exact mass	1179.55 g/mol
Identified molecular ions (ESI)	*m/z 1202.83 for [M+Na]$^+$*<br>*m/z 1181.11 for [M+H]$^+$*
Identified molecular ions (MALDI)	*m/z 1181.4 for [M+H]$^+$*<br>*m/z 1203.6 for [M+Na]$^+$*<br>*m/z 1219.3 for [M+K]$^+$*
Yield	12.9 mg (41 % from 31.0 mg crude peptide)
Purity (HPLC, 214 nm)	> 98 %
Chromatogram	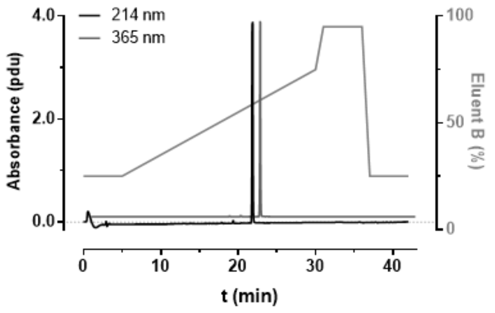
Mass spectrum (ESI)	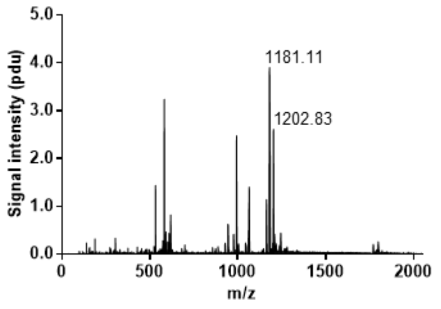
Mass spectrum (MALDI)	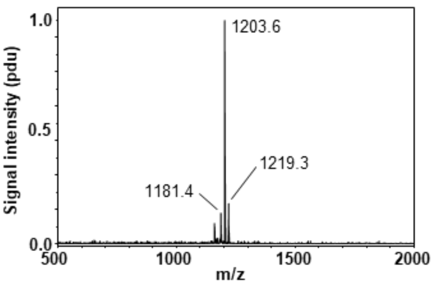

Abz-Gly-Phe-Val-Cit-Ala-Lys(Dnp)-Sar-Val-NH$_2$ (58)

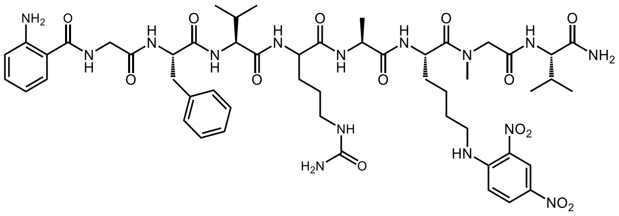

Chemical formula	$C_{52}H_{73}N_{15}O_{14}$
Molecular weight	1132.25
Exact mass	1131.5461
Identified molecular ions (HR-ESI)	*1154.5348 for [M+Na]$^+$ (calcd. 1154.5354)*<br>*1132.5529 for [M+H]$^+$ (calcd. 1132.5534)*
Yield	11.3 mg
Purity (HPLC, 214 nm)	96.3 %
Chromatogram	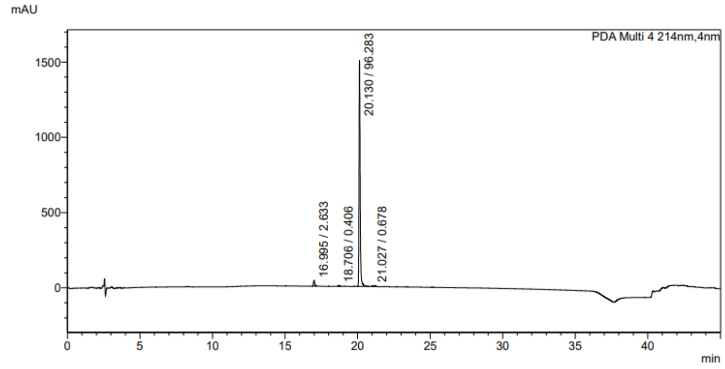
Mass spectrum (HR-ESI)	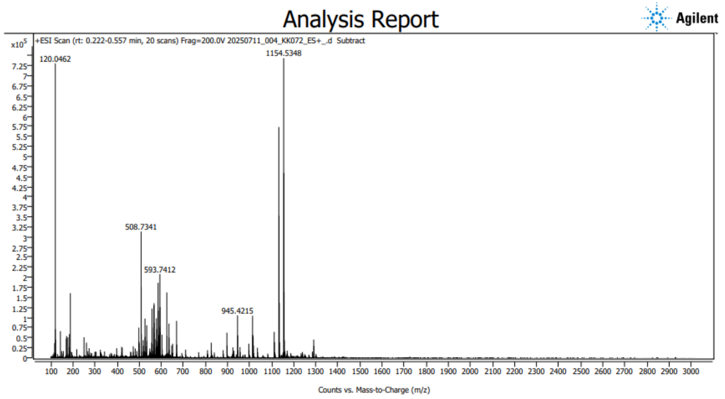

Abz-Gly-Phe-ACBC-Cit-Ala-Lys(Dnp)-Sar-Val-NH_2 (59)

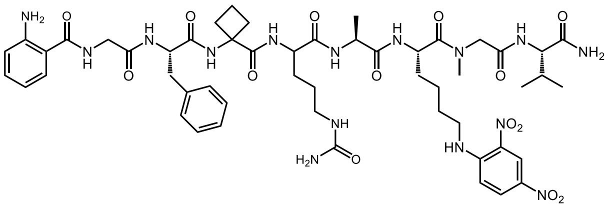

Chemical formula	$C_{52}H_{71}N_{15}O_{14}$
Molecular weight	1130.23
Exact mass	1129.5305
Identified molecular ions (HR-ESI)	*1130.5370 for $[M+H]^+$ (calcd. 1130.5378)*<br>*1152.5186 for $[M+Na]^+$ (calcd. 1152.5197)*
Yield	13.4 mg
Purity (HPLC, 214 nm)	98.1 %
Chromatogram	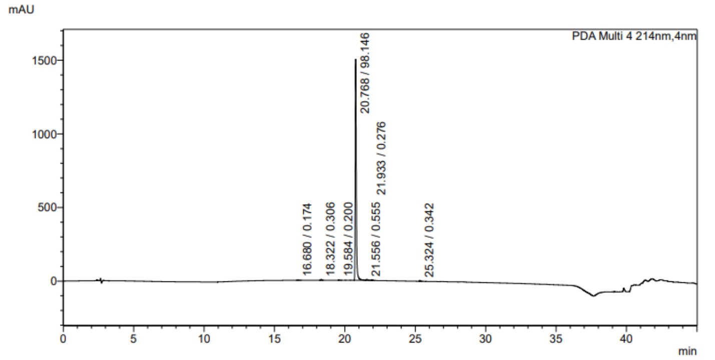
Mass spectrum (HR-ESI)	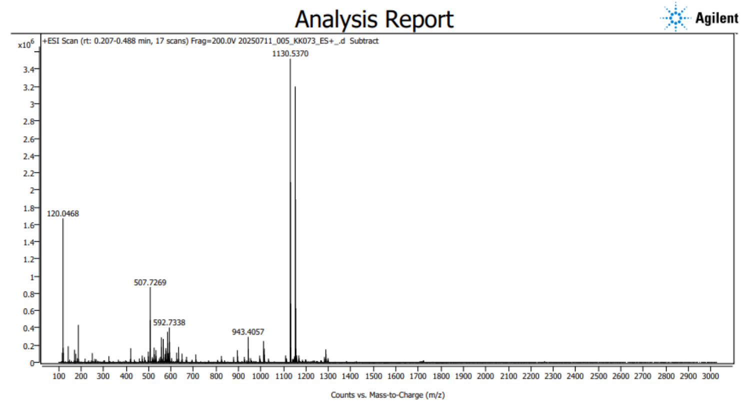

Abz-Gly-Leu-Phe(3-Me)-Cit-Ala-Lys(Dnp)-Sar-Val-NH$_2$ (60)

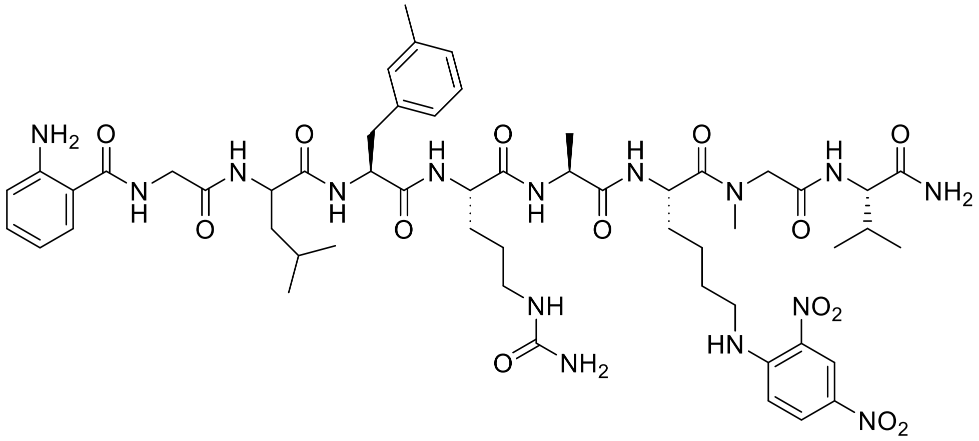

Chemical formula	$C_{54}H_{77}N_{15}O_{14}$
Molecular weight	1160.28 g/mol
Exact mass	1159.58 g/mol
Identified molecular ions (MALDI)	*1161.3 for [M+H]$^+$*<br>*1183.5 for [M+Na]$^+$*
Yield	29.4 mg (46 %)
Purity (HPLC, 214 nm)	> 98 %
Chromatogram	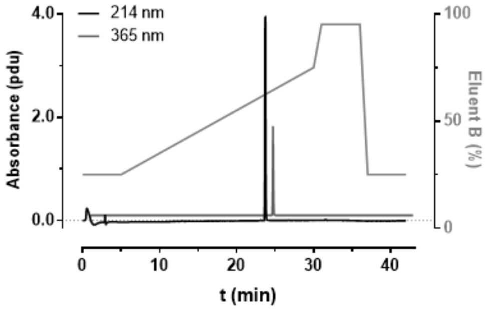
Mass spectrum (MALDI)	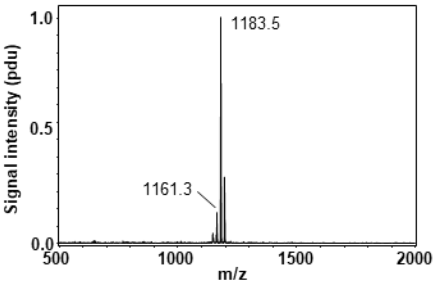

Abz-Gly-Ile-Val-Cit-Ala-Lys(Dnp)-Sar-Val-NH$_2$ (61)

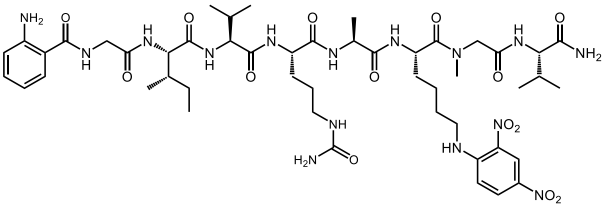

Chemical formula	$C_{59}H_{79}N_{15}O_{14}$
Molecular weight	1098.26 g/mol
Exact mass	1097.56 g/mol
Identified molecular ions (ESI)	*m/z 1132.15 for [M+H]$^+$*<br>*m/z 1012.49 for [M-Abz+H]$^+$*<br>*m/z 566.32 for [M+2H]$^{2+}$*
Identified molecular ions (MALDI)	*m/z 1099.4 for [M+H]$^+$*<br>*m/z 1121.3 for [M+Na]$^+$*<br>*m/z 1137.3 for [M+K]$^+$*
Yield	6.5 mg (20 % from 32.0 mg crude peptide)
Purity (HPLC, 214 nm)	> 98 %
Chromatogram	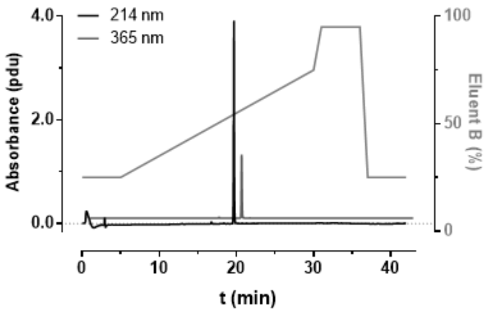
Mass spectrum (ESI)	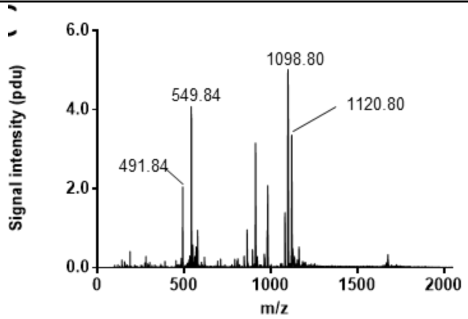
Mass spectrum (MALDI)	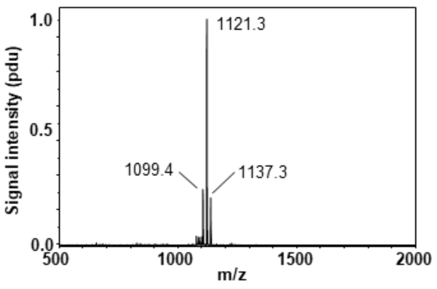

Abz-Gly-Ile-ACBC-Cit-Ala-Lys(Dnp)-Sar-Val-NH_2 (62)

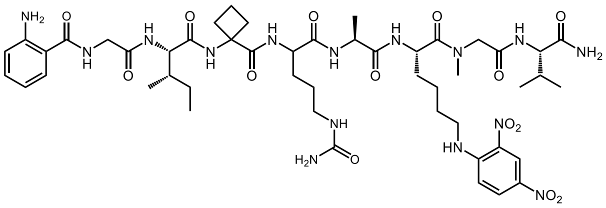

Chemical formula	$C_{49}H_{73}N_{15}O_{14}$
Molecular weight	1096.20 g/mol
Exact mass	1095.55 g/mol
Identified molecular ions (ESI)	*m/z 1118.43 for $[M+Na]^+$*<br>*m/z 1096.40 for $[M+H]^+$*<br>*m/z 977.74for $[M-Abz+H]^+$*<br>*m/z 548.80 for $[M+2H]^{2+}$*
Identified molecular ions (MALDI)	*m/z 1097.3 for $[M+H]^+$*<br>*m/z 1119.5 for $[M+Na]^+$*<br>*m/z 1135.3 for $[M+K]^+$*
Yield	14.6 mg (47 % from 31.3 mg crude peptide)
Purity (HPLC, 214 nm)	> 98 %
Chromatogram	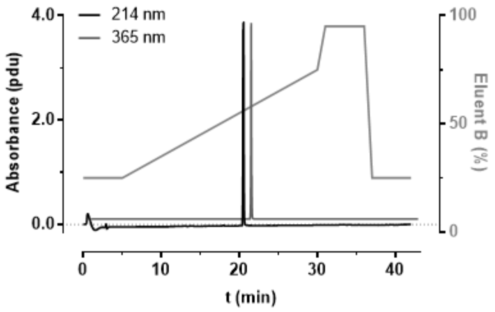
Mass spectrum (ESI)	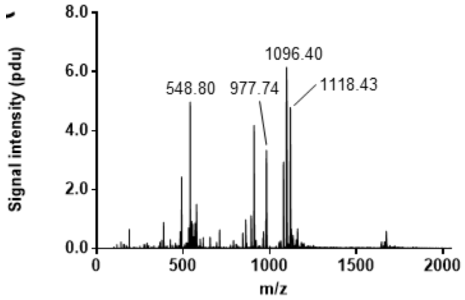
Mass spectrum (MALDI)	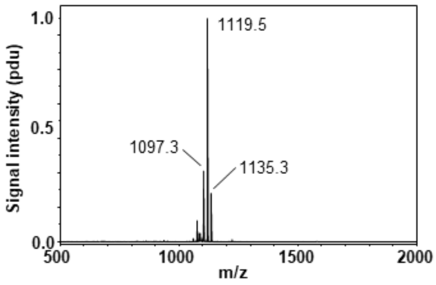

Abz-Gly-Ile-Val-Arg(ec)-Ala-Lys(Dnp)-Sar-Val-NH_2 (63)

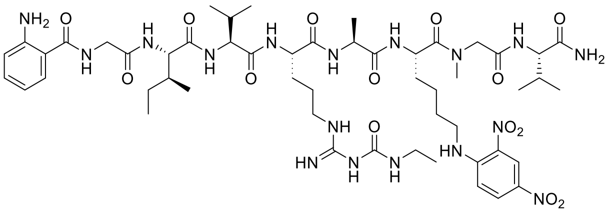

Chemical formula	$C_{52}H_{81}N_{17}O_{14}$
Molecular weight	1168.31 g/mol
Exact mass	1167.6149 g/mol
Identified molecular ions (HR-ESI)	*1168.6207 for $[M+H]^+$ (calcd. 1168.6222)*<br>*584.8160 for $[M+2H]^+$ (calcd. 584.8147)*
Yield	18.2 mg (26 %)
Purity (HPLC, 214 nm)	90.7 %
Chromatogram	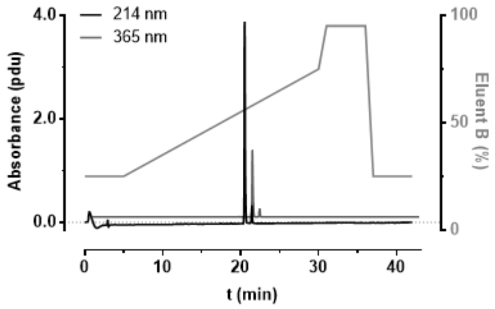
Mass spectrum (HR-ESI)	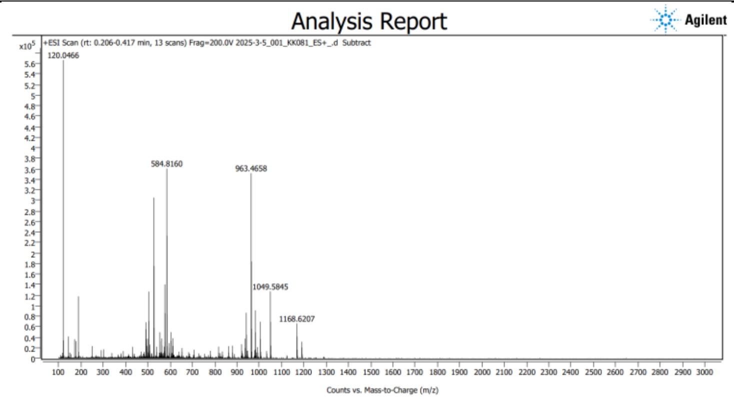

Abz-Gly-Ile-Phe(3-Me)-Arg(ec)-Ala-Lys(Dnp)-Sar-Val-NH_2 (64)

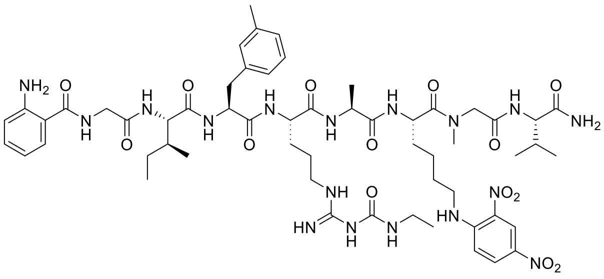

Chemical formula	$C_{57}H_{83}N_{17}O_{14}$
Molecular weight	1230.37 g/mol
Exact mass	1229.63 g/mol
Identified molecular ions (HR-ESI)	*1230.6373 for $[M+H]^+$ (calcd. 1230.6378)*<br>*1252.6190 for $[M+Na]^+$ (calcd. 1252.6198)*<br>*615.8231 for $[M+2H]^{2+}$ (calcd. 615.8226)*
Yield	17.7 mg (24 %)
Purity (HPLC, 214 nm)	> 98 %
Chromatogram	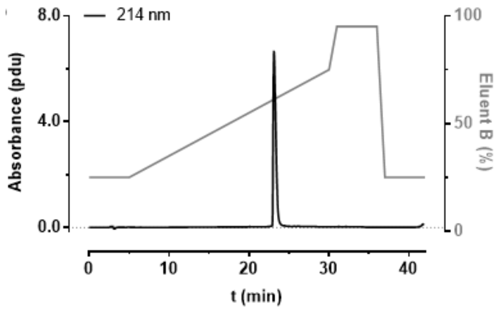
Mass spectrum (HR-ESI)	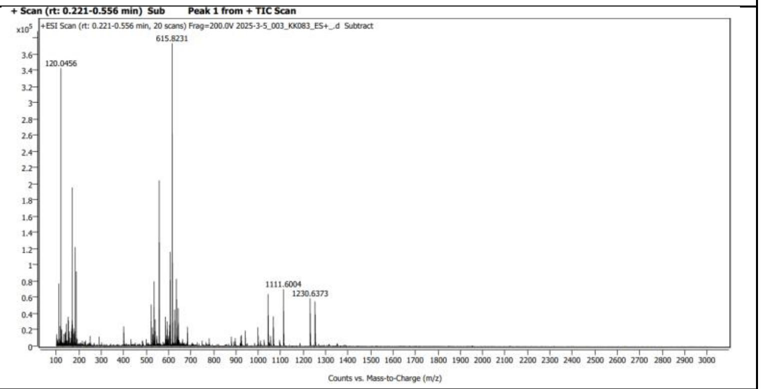

Abz-Gly-Ile-Phe(3-I)-Arg(ec)-Ala-Lys(Dnp)-Sar-Val-NH_2 (65)

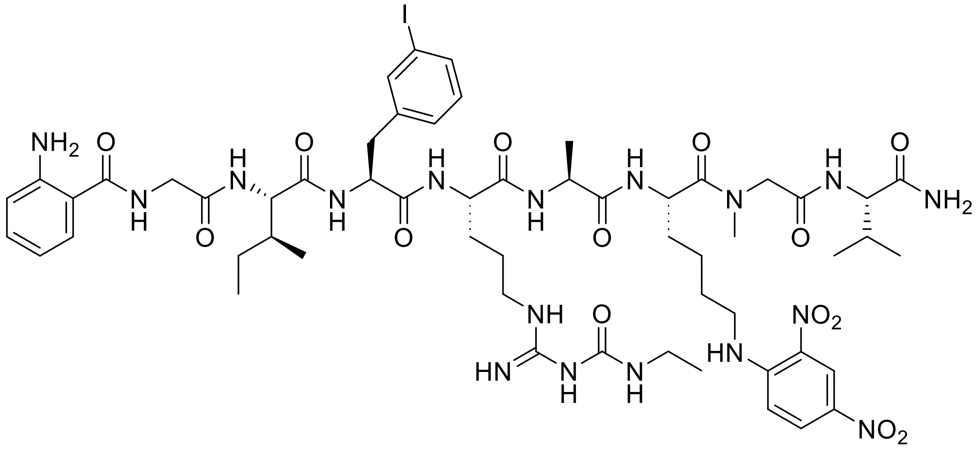

Chemical formula	$C_{56}H_{80}IN_{17}O_{14}$
Molecular weight	1342.24 g/mol
Exact mass	1341.51 g/mol
Identified molecular ions (HR-ESI)	*1364.4993 for [M+Na]$^+$ (calcd. 1364.5008)*<br>*1342.5175 for [M+H]$^+$ (calcd. 1342.5189)*<br>*671.7634 for [M+2H]$^{2+}$ (calcd. 671.7631)*
Yield	4.3 mg (5 %)
Purity (HPLC, 214 nm)	> 98 %
Chromatogram	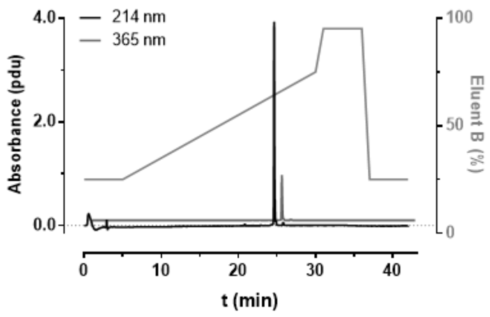
Mass spectrum (HR-ESI)	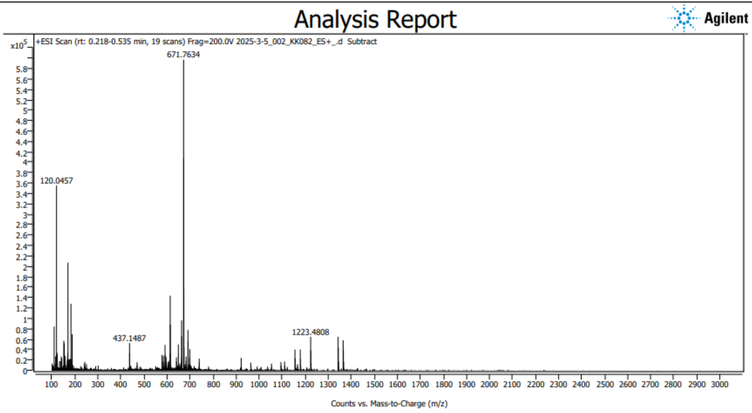

Abz-Gly-Leu-Phe-Gly-Phe-Lys(Dnp)-Sar-Val-NH$_2$ (66)

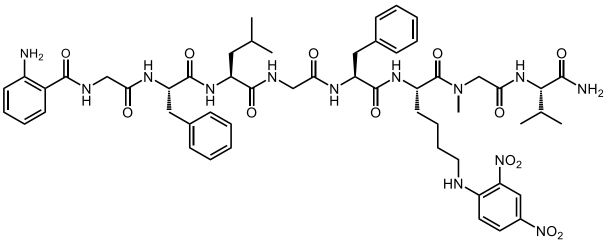

Chemical formula	$C_{55}H_{71}N_{13}O_{13}$
Molecular weight	1122.23 g/mol
Exact mass	1121.73 g/mol
Identified molecular ions (ESI)	*m/z 1122.83 for [M+H]$^+$*<br>*m/z 1145.16 for [M+Na]$^+$*
Identified molecular ions (MALDI)	*m/z 1121.3 for [M+H]+*<br>*m/z 1144.4 for [M+Na]+*<br>*m/z 1161.3 for [M+K]+*
Yield	13.3 mg (32 % from 31.0 mg crude peptide)
Purity (HPLC, 214 nm)	> 98 %
Chromatogram	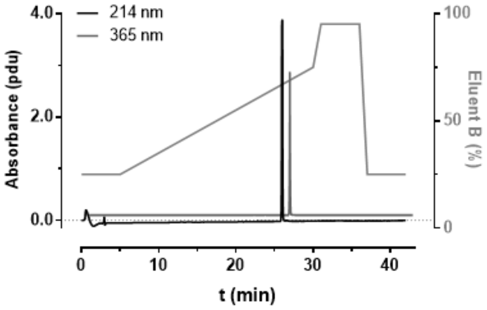
Mass spectrum (ESI)	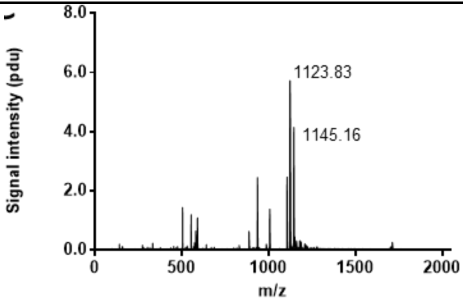
Mass spectrum (MALDI)	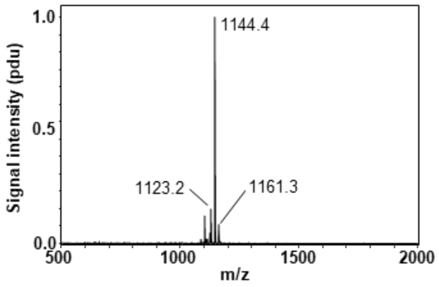

Abz-Gly-Phe-Leu-Cit-Phe-Lys(Dnp)-Sar-Val-NH$_2$ (67)

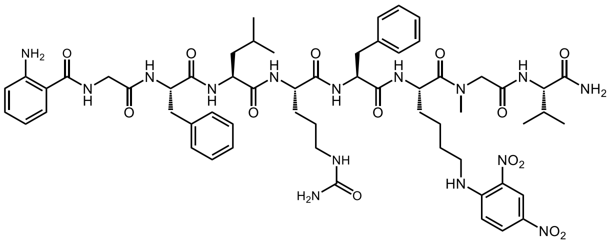

Chemical formula	$C_{59}H_{79}N_{15}O_{14}$
Molecular weight	1222.35 g/mol
Exact mass	1221.59 g/mol
Identified molecular ions (HR-ESI)	*m/z 1244.5808 for [M+Na]$^+$ (calcd. 1244.5823)*<br>*m/z 1222.5989 for [M+H]$^+$ (calcd. 1222.6004)*<br>*m/z 611.8049 for [M+2H]$^{2+}$ (calcd. 611.8038)*
Yield	6.5 mg (20 % from 32.0 mg crude peptide)
Purity (HPLC, 214 nm)	99.4 %
Chromatogram	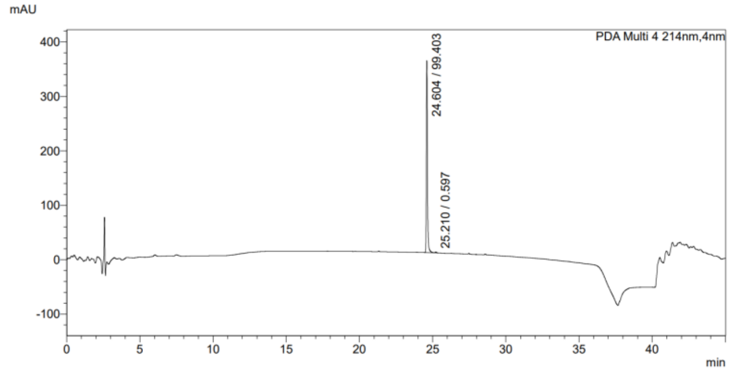
Mass spectrum (ESI)	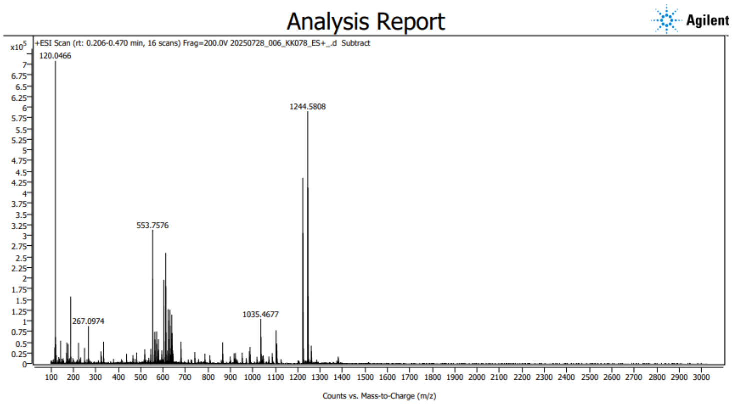

Ac-(glu)$_9$-Ahx-Gly-Phe-Leu-Gly-Ala-Lys(Dnp)-Sar-Val-Ahx-(arg)$_9$-Ahx-K(TAM)-NH$_2$ (68)

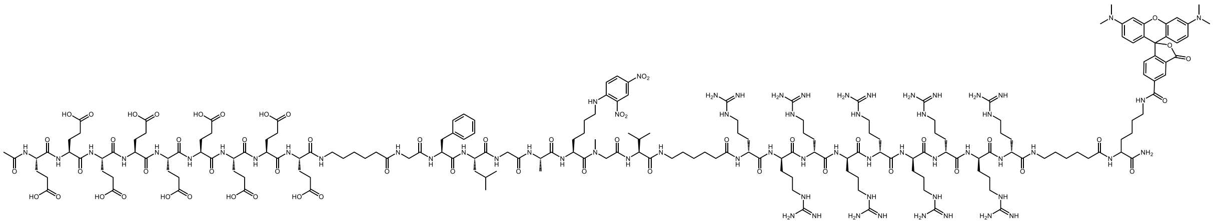

Chemical formula	$C_{192}H_{300}N_{64}O_{57}$
Molecular weight	4416.90 g/mol
Exact mass	4414.25 g/mol
Identified molecular ions (HR-ESI)	*m/z 884.2580 for $[M+5H]^{5+}$ (calcd. 884.2593)*<br>*m/z 737.0495 for $[M+6H]^{6+}$ (calcd. 737.0506)*
Yield	42.8 mg
Purity (HPLC, 214 nm)	96.5 %
Chromatogram	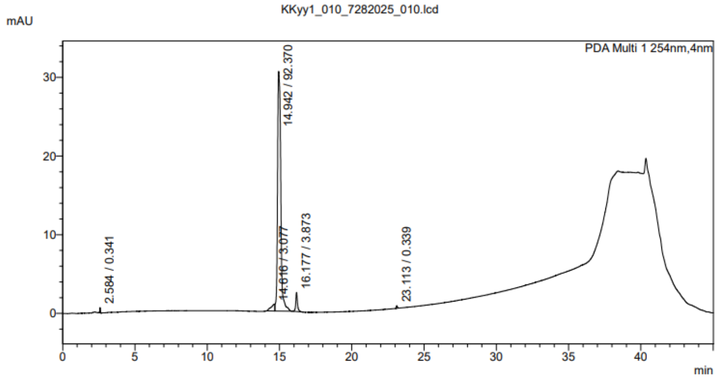
Mass spectrum (ESI)	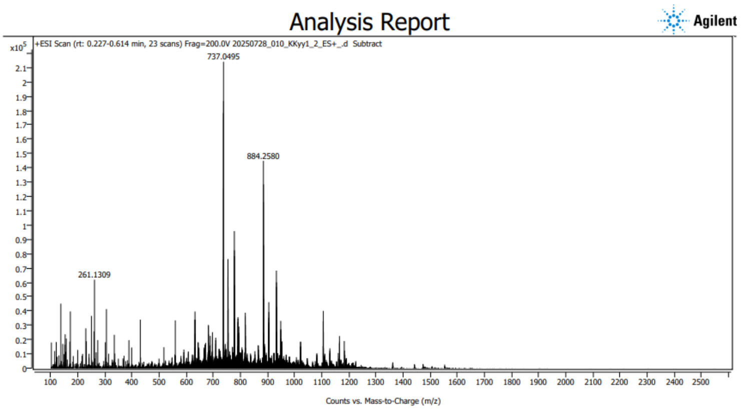

Ac-(glu)$_9$-Ahx-(ala)$_8$-(arg)$_9$-Ahx-K(NODA-GA)-NH$_2$ (69)

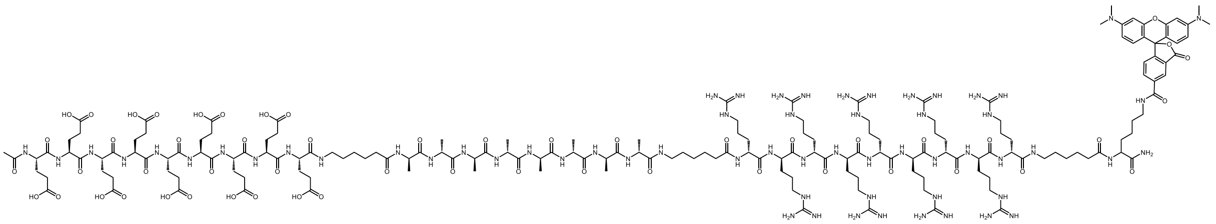

Chemical formula	$C_{174}H_{281}N_{61}O_{53}$
Molecular weight	4075.54 g/mol
Exact mass	4073.12 g/mol
Identified molecular ions (HR-ESI)	*m/z 1019.7867 for $[M+4H]^{4+}$ (calcd. 1019.7878)*<br>*m/z 816.0310 for $[M+5H]^{5+}$ (calcd. 816.0317)*<br>*m/z 680.1937 for $[M+6H]^{6+}$ (calcd. 680.1943)*
Yield	16.4 mg
Purity (HPLC, 214 nm)	97.4%
Chromatogram	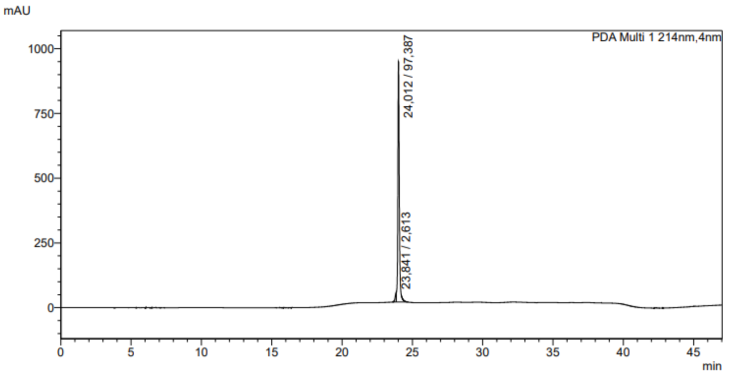
Mass spectrum (ESI)	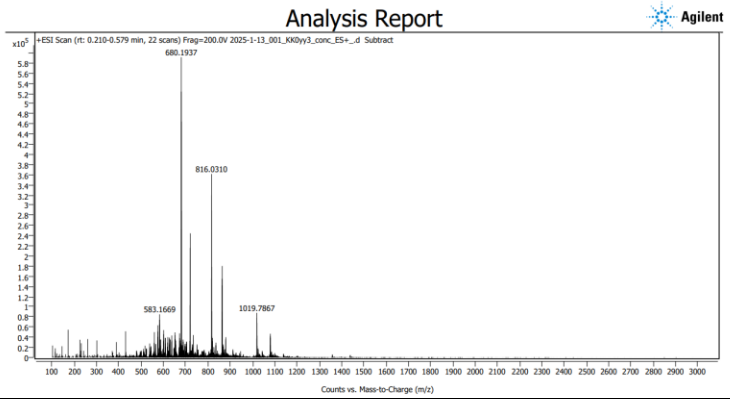

H-Ala-Lys(Dnp)-Sar-Val-Ahx-(arg)$_9$-Ahx-K(TAM)-NH$_2$ (70)

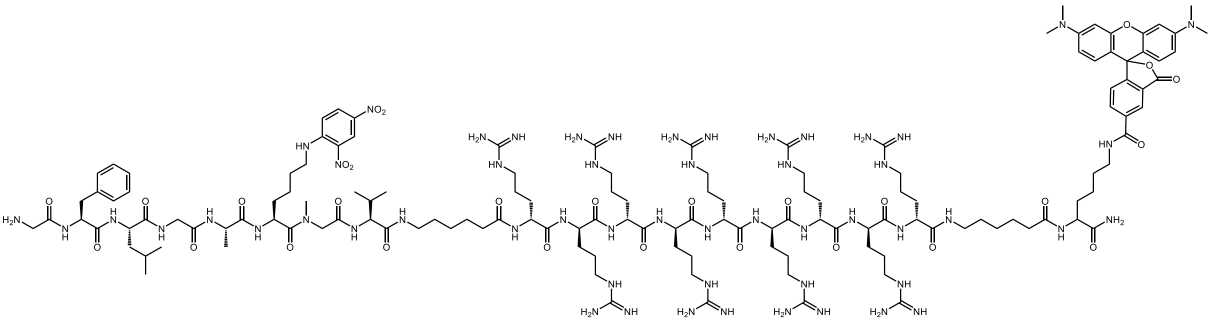

Chemical formula	$C_{120}H_{198}N_{50}O_{24}$
Molecular weight	2725.23 g/mol
Exact mass	2723.58 g/mol
Purity (HPLC, 214 nm)	>99.9%
Chromatogram	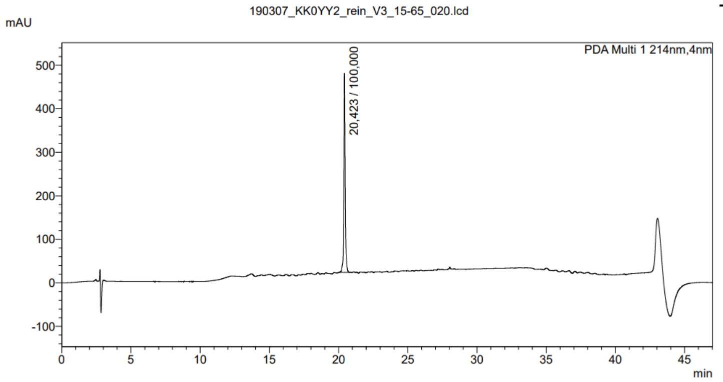

Ac-(glu)$_9$-Ahx-Gly-Phe-Leu-Gly-Ala-Lys(Dnp)-Sar-Val-Ahx-(arg)$_9$-Ahx-K(NODA-GA)-NH$_2$ (71)

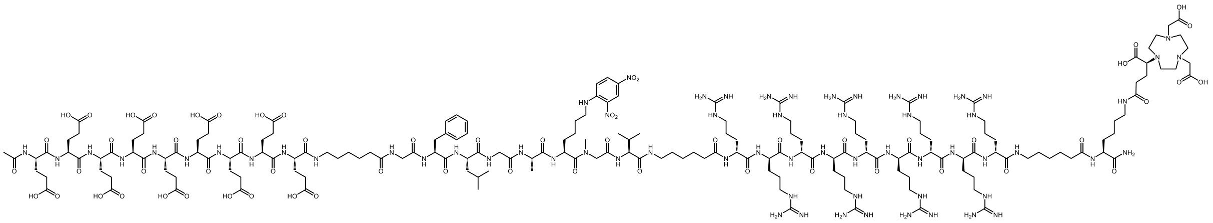

Chemical formula	$C_{182}H_{303}N_{65}O_{60}$
Molecular weight	4361.82 g/mol
Exact mass	4359.27 g/mol
Identified molecular ions (HR-ESI)	*m/z 1454.7630 for [M+3H]$^{3+}$ (calcd. 1454.7643)*<br>*m/z 1091.3240 for [M+4H]$^{4+}$ (calcd. 1091.3250)*<br>*m/z 873.2607 for [M+5H]$^{5+}$ (calcd. 873.2615)*<br>*m/z 727.8849 for [M+6H]$^{6+}$ (calcd. 727.8858)*
Yield	69.3 mg
Purity (HPLC, 214 nm)	95.5 %
Chromatogram	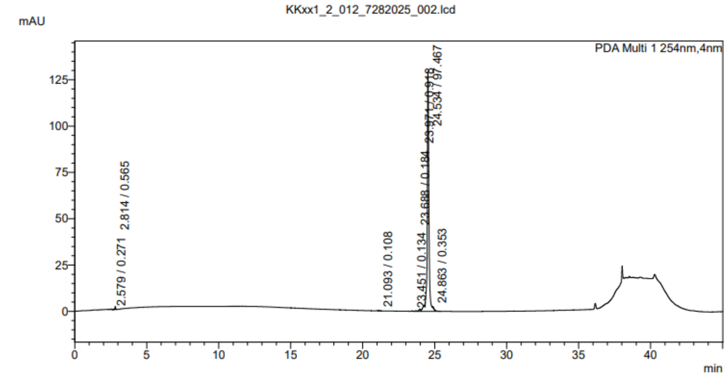
Mass spectrum (ESI)	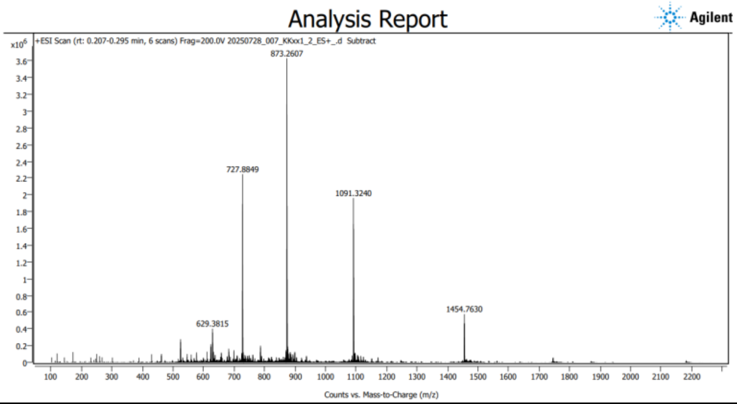

Ac-(glu)$_9$-Ahx-(ala)$_8$-(arg)$_9$-Ahx-K(NODA-GA)-NH$_2$ (72)

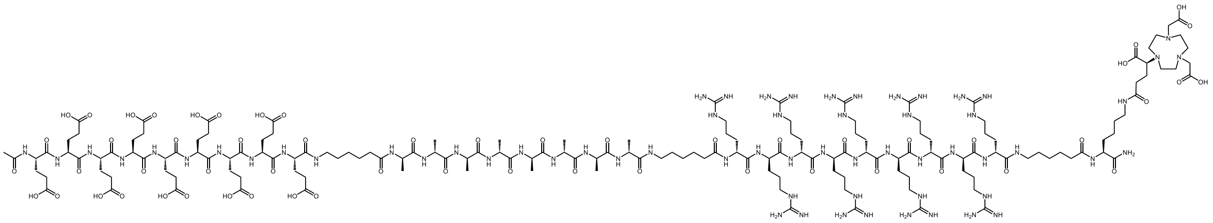

Chemical formula	$C_{164}H_{284}N_{62}O_{56}$
Molecular weight	4020.45 g/mol
Exact mass	4018.13 g/mol
Identified molecular ions (HR-ESI)	*m/z 1341.0505 for $[M+3H]^{3+}$ (calcd. 1341.0518)*<br>*m/z 1006.0397 for $[M+4H]^{4+}$ (calcd. 1006.0406)*<br>*m/z 805.0334 for $[M+5H]^{5+}$ (calcd. 805.0340)*
Yield	18.7 mg
Purity (HPLC, 214 nm)	99.5 %
Chromatogram	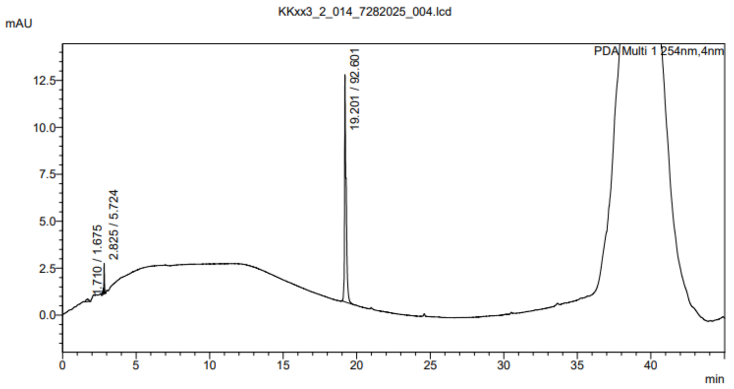
Mass spectrum (ESI)	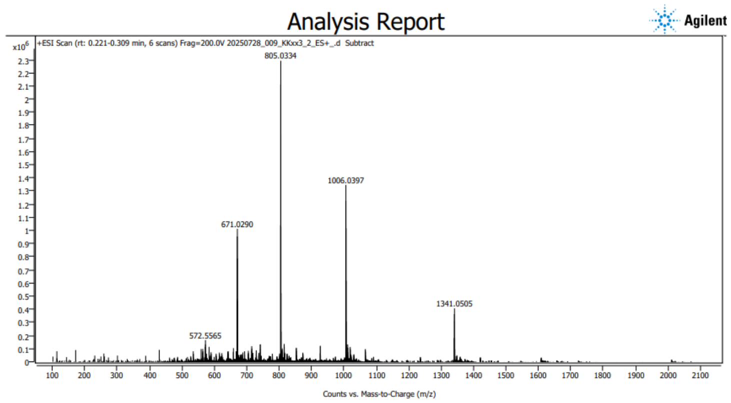

H-Ala-Lys(Dnp)-Sar-Val-Ahx-(arg)$_9$-Ahx-K(NODA-GA)-NH$_2$ (73)

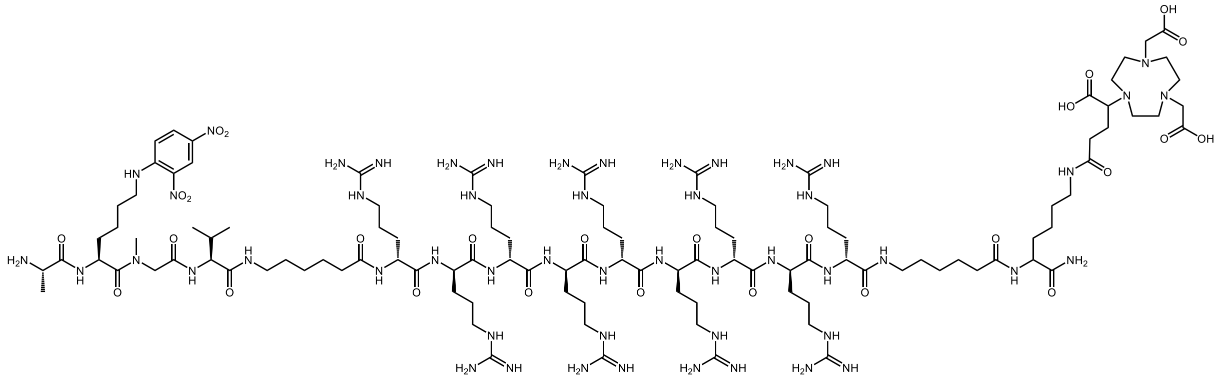

Chemical formula	$C_{110}H_{201}N_{53}O_{27}$
Molecular weight	2670.15 g/mol
Exact mass	2668.15 g/mol
Identified molecular ions (HR-ESI)	*m/z 890.8714 for $[M+3H]^{3+}$ (calcd. 890.8725)*<br>*m/z 668.4054 for $[M+4H]^{4+}$ (calcd. 668.4062)*<br>*m/z 534.9258 for $[M+5H]^{5+}$ (calcd. 534.9264)*
Yield	67.1 mg
Purity (HPLC, 214 nm)	97.1 %
Chromatogram	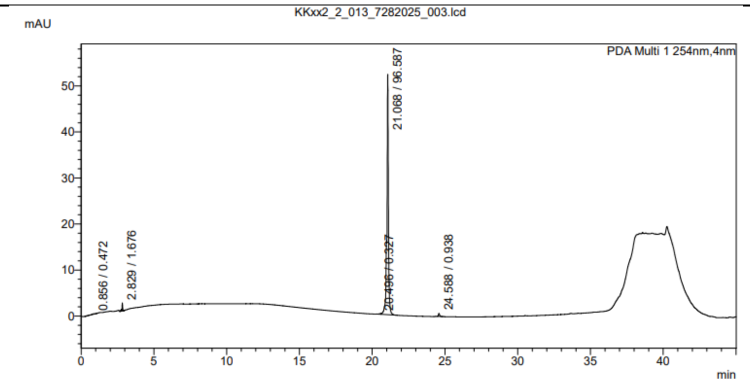
Mass spectrum (ESI)	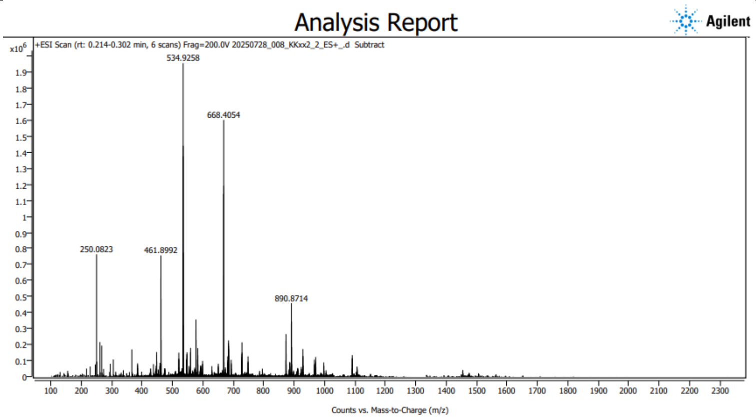

Ac-(glu)$_9$-Ahx-Gly-Phe-Leu-Gly-^{N}MeAla-Lys(Dnp)-Sar-Val-Ahx-(arg)$_9$-Ahx-K(NODA-GA)-NH$_2$ (74)

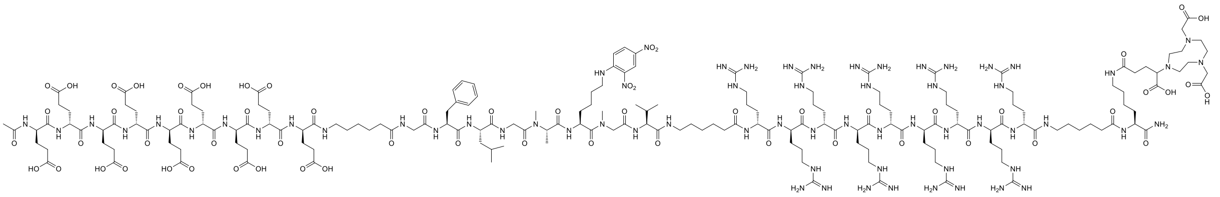

Chemical formula	$C_{183}H_{305}N_{65}O_{60}$
Molecular weight	4375.85 g/mol
Exact mass	4373.28 g/mol
Identified molecular ions (HR-ESI)	*m/z 1094.8301 for $[M+4H]^{4+}$ (calcd. 1094.8290)*<br>*m/z 730.2234 for $[M+6H]^{6+}$ (calcd. 730.2217)*
Purity (HPLC, 254 nm)	100%
Chromatogram	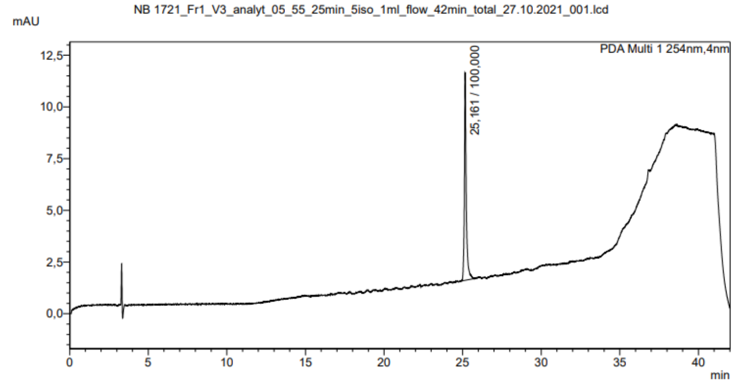
Mass spectrum (ESI)	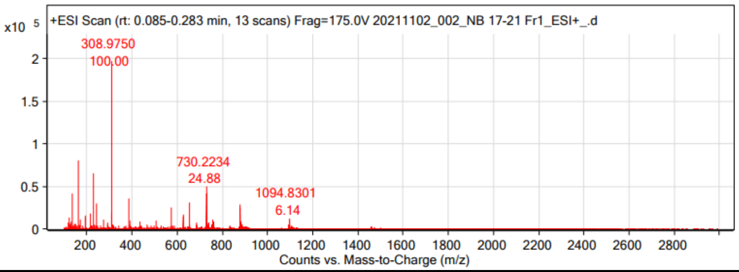

Radio-HPLC chromatograms for radiolabeling of compounds **71**-**74** with copper-64

A

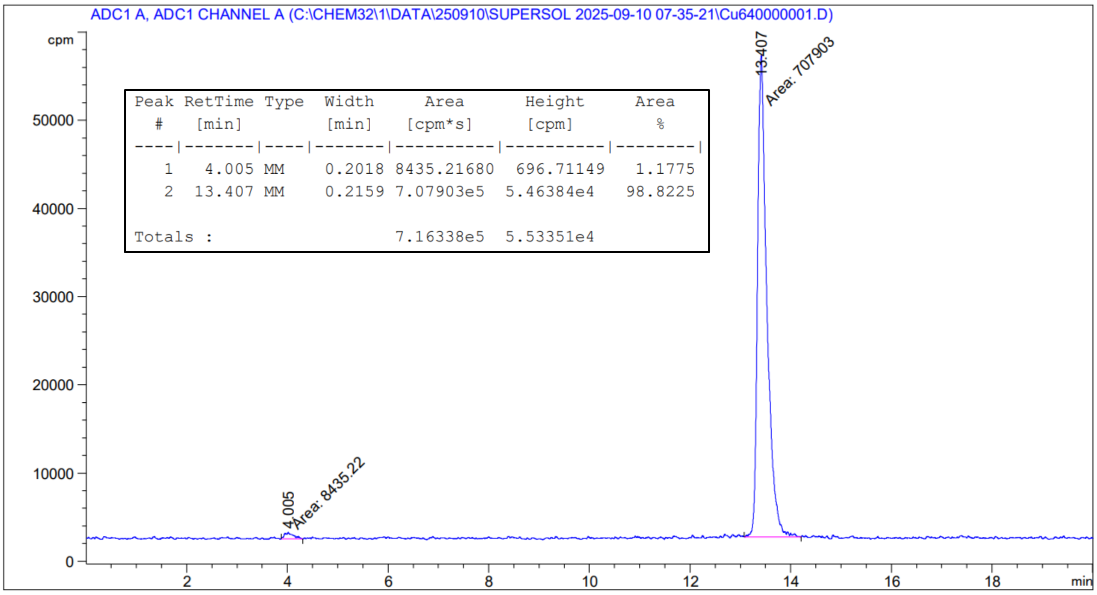

B

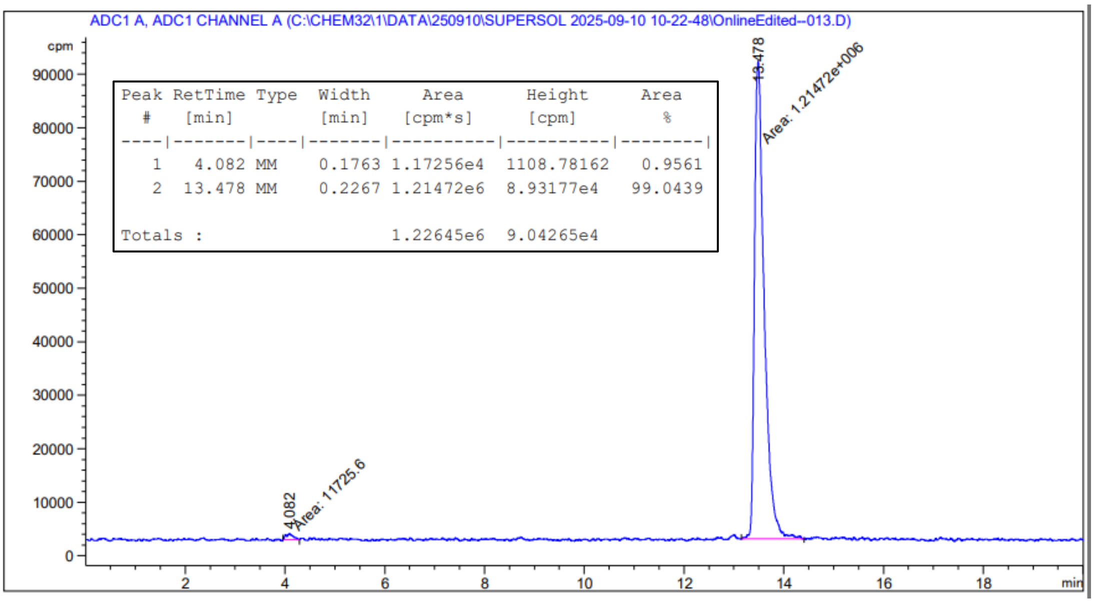

Figure S33: Radio-HPLC chromatograms with integration table (inset) of compound [^{64}Cu]Cu-**71** immediately after radiolabeling (**A**) and after 8 h at room temperature in aqueous solution (**B**).

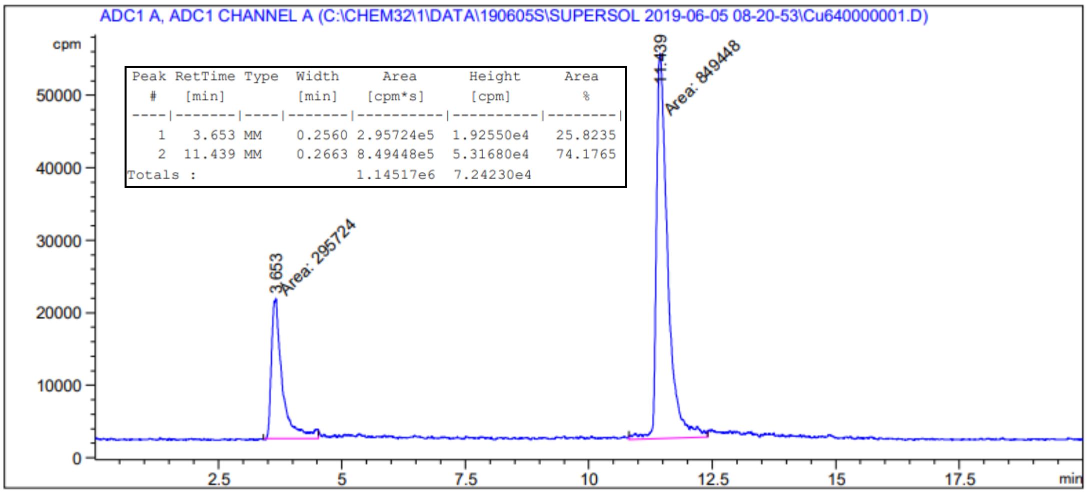

Figure S34: Radio-HPLC chromatogram with integration table (inset) of compound [^{64}Cu]Cu-**72** immediately after radiolabeling.

A

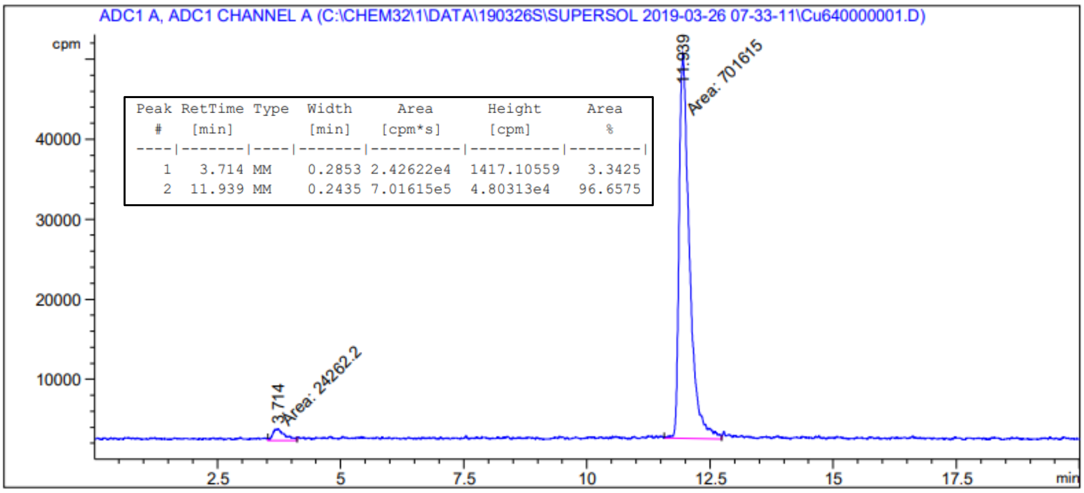

B

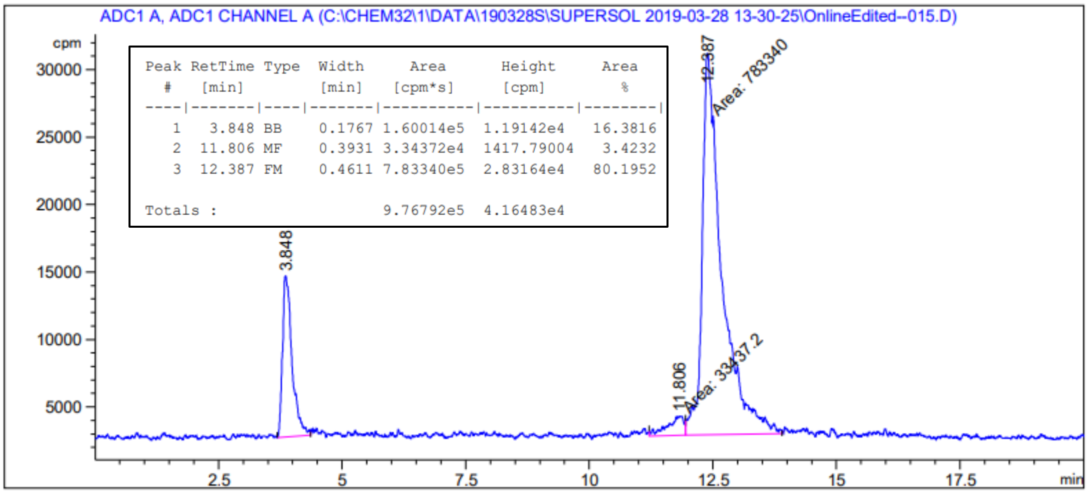

Figure S35: Radio-HPLC chromatograms with integration table (inset) of compound [^{64}Cu]Cu-**73** immediately after radiolabeling (**A**) and after 9 h at room temperature in aqueous solution (**B**).

A

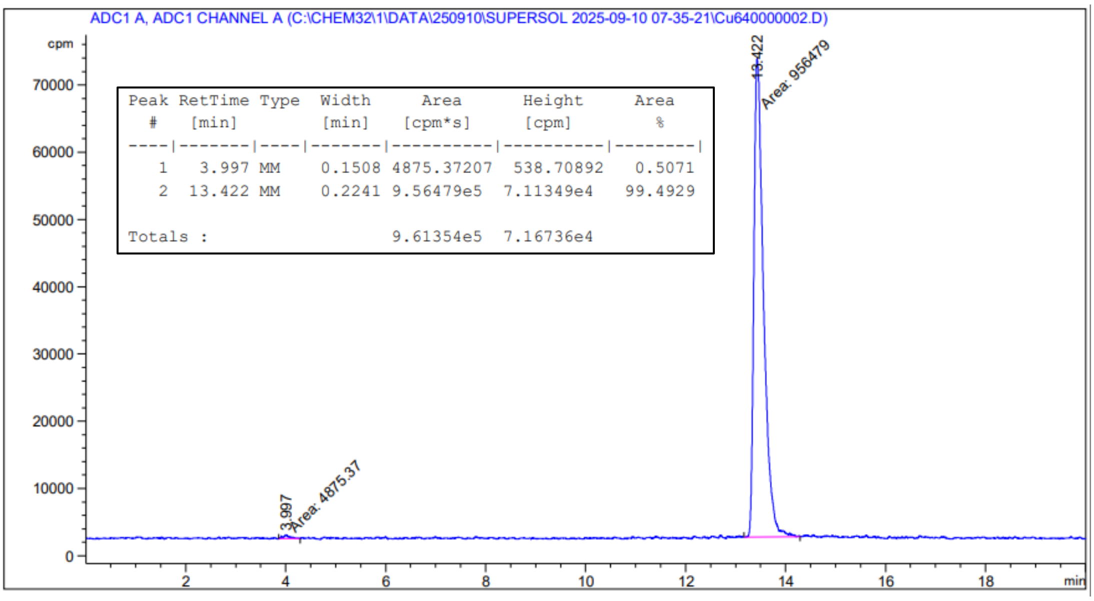

Peak #	RetTime [min]	Type	Width [min]	Area [cpm*s]	Height [cpm]	Area %
1	3.997	MM	0.1508	4875.37207	538.70892	0.5071
2	13.422	MM	0.2241	9.56479e5	7.11349e4	99.4929
Totals :				9.61354e5	7.16736e4	

B

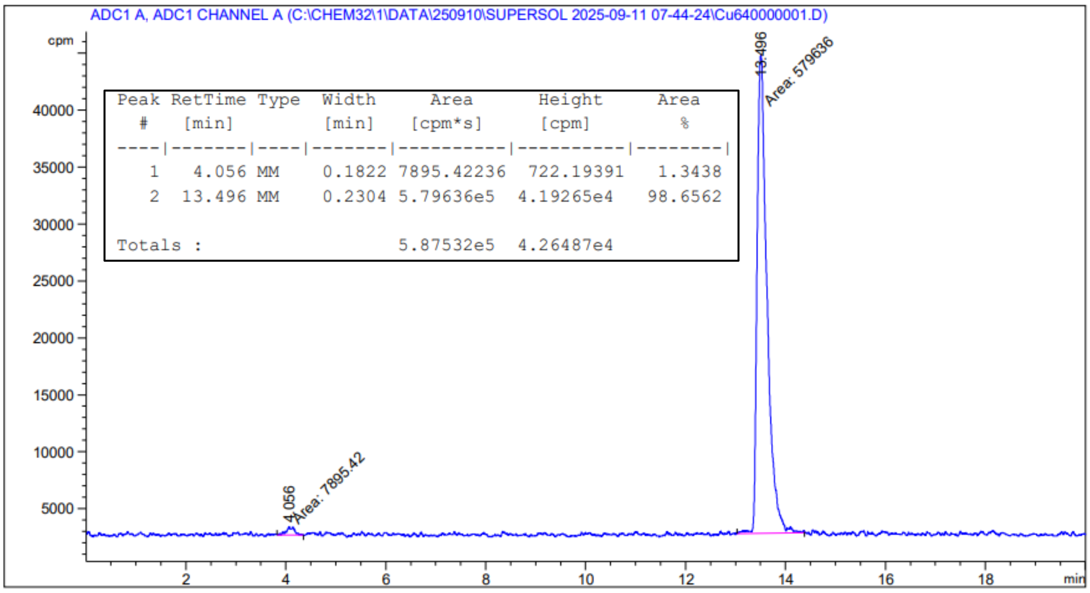

Peak #	RetTime [min]	Type	Width [min]	Area [cpm*s]	Height [cpm]	Area %
1	4.056	MM	0.1822	7895.42236	722.19391	1.3438
2	13.496	MM	0.2304	5.79636e5	4.19265e4	98.6562
Totals :				5.87532e5	4.26487e4	

Figure S36: Radio-HPLC chromatograms with integration table (inset) of compound [^{64}Cu]Cu-**74** immediately after radiolabeling (**A**) and after 8 h at room temperature in aqueous solution (**B**).

References for Supporting Information

(1) Cherkupally, P., Ramesh, S., Govender, T., Kruger, H. G., de la Torre, B. G., and Albericio, F. (**2015**) An efficient solid-phase strategy for total synthesis of naturally occurring amphiphilic marine siderophores: amphibactin-T and moanachelin ala-B, *Org. Biomol. Chem. 13*, 4760-4768, doi: 10.1039/c5ob00100e.

(2) Keller, M., Kuhn, K. K., Einsiedel, J., Hubner, H., Biselli, S., Mollereau, C., Wifling, D., Svobodova, J., Bernhardt, G., Cabrele, C., Vanderheyden, P. M., Gmeiner, P., and Buschauer, A. (**2016**) Mimicking of Arginine by Functionalized N(omega)-Carbamoylated Arginine As a New Broadly Applicable Approach to Labeled Bioactive Peptides: High Affinity Angiotensin, Neuropeptide Y, Neuropeptide FF, and Neurotensin Receptor Ligands As Examples, *J. Med. Chem. 59*, 1925-1945, doi: 10.1021/acs.jmedchem.5b01495.

(3) Pluym, N., Brennauer, A., Keller, M., Ziemek, R., Pop, N., Bernhardt, G., and Buschauer, A. (**2011**) Application of the guanidine-acylguanidine bioisosteric approach to argininamide-type NPY Y(2) receptor antagonists, *ChemMedChem 6*, 1727-1738, doi: 10.1002/cmdc.201100241.

(4) Keller, M., Weiss, S., Hutzler, C., Kuhn, K. K., Mollereau, C., Dukorn, S., Schindler, L., Bernhardt, G., König, B., and Buschauer, A. (**2015**) N(omega)-Carbamoylation of the Argininamide Moiety: An Avenue to Insurmountable NPY Y1 Receptor Antagonists and a Radiolabeled Selective High-Affinity Molecular Tool ([(3)H]UR-MK299) with Extended Residence Time, *J. Med. Chem. 58*, 8834-8849, doi: 10.1021/acs.jmedchem.5b00925.

(5) Powers, J. C., Asgian, J. L., Ekici, O. D., and James, K. E. (**2002**) Irreversible inhibitors of serine, cysteine, and threonine proteases, *Chem. Rev. 102*, 4639-4750, doi: 10.1021/cr010182v.

(6) Shah, F., Wu, Y., Gut, J., Pedduri, Y., Legac, J., Rosenthal, P. J., and Avery, M. A. (**2011**) Design, synthesis and biological evaluation of novel benzothiazole and triazole analogs as falcipain inhibitors, *MedChemComm 2*, doi: 10.1039/c1md00129a.

(7) Yoon, M. C., Christy, M. P., Phan, V. V., Gerwick, W. H., Hook, G., O'Donoghue, A. J., and Hook, V. (**2022**) Molecular Features of CA-074 pH-Dependent Inhibition of Cathepsin B, *Biochemistry 61*, 228-238, doi: 10.1021/acs.biochem.1c00684.

(8) Woo, J.-T., Sigeizumi, S., Yamaguchi, K., Sugimoto, K., Kobori, T., Tsuji, T., and Kondo, K. (**1995**) Peptidyl aldehyde derivatives as potent and selective inhibitors of cathepsin L, *Bioorg. Med. Chem. Lett. 5*, 1501-1504, doi: 10.1016/0960-894x(95)00236-m.

(9) Zeng, G. Z., Tan, N. H., Hao, X. J., Mu, Q. Z., and Li, R. T. (**2006**) Natural inhibitors targeting osteoclast-mediated bone resorption, *Bioorg. Med. Chem. Lett. 16*, 6178-6180, doi: 10.1016/j.bmcl.2006.09.042.

(10) Baidya, S. K., Banerjee, S., Adhikari, N., and Jha, T. (**2022**) Selective Inhibitors of Medium-Size S1' Pocket Matrix Metalloproteinases: A Stepping Stone of Future Drug Discovery, *J. Med. Chem. 65*, 10709-10754, doi: 10.1021/acs.jmedchem.1c01855.

(11) Gossas, T., Nordström, H., Xu, M.-H., Sun, Z.-H., Lin, G.-Q., Wallberg, H., and Danielson, U. H. (**2013**) The advantage of biosensor analysis over enzyme inhibition studies for slow dissociating inhibitors – characterization of hydroxamate-based matrix metalloproteinase-12 inhibitors, *MedChemComm 4*, doi: 10.1039/c2md20268a.

(12) Azim, M. K., Ahmed, W., Khan, I. A., Rao, N. A., and Khan, K. M. (**2008**) Identification of acridinyl hydrazides as potent aspartic protease inhibitors, *Bioorg. Med. Chem. Lett. 18*, 3011-3015, doi: 10.1016/j.bmcl.2008.02.060.

(13) Liu, Y., Zhang, W., Li, L., Salvador, L. A., Chen, T., Chen, W., Felsenstein, K. M., Ladd, T. B., Price, A. R., Golde, T. E., He, J., Xu, Y., Li, Y., and Luesch, H. (**2012**) Cyanobacterial peptides as a prototype for the design of potent beta-secretase inhibitors and the development of selective chemical probes for other aspartic proteases, *J Med Chem 55*, 10749-10765, doi: 10.1021/jm301630s.

(14) Sharma, U., Cozier, G. E., Sturrock, E. D., and Acharya, K. R. (2020) Molecular Basis for Omapatrilat and Sampatrilat Binding to Neprilysin-Implications for Dual Inhibitor Design with Angiotensin-Converting Enzyme, *J. Med. Chem. 63*, 5488-5500, doi: 10.1021/acs.jmedchem.0c00441.
(15) Mai, L. H., Chabot, G. G., Grellier, P., Quentin, L., Dumontet, V., Poulain, C., Espindola, L. S., Michel, S., Vo, H. T., Deguin, B., and Grougnet, R. (2015) Antivascular and anti-parasite activities of natural and hemisynthetic flavonoids from New Caledonian Gardenia species (Rubiaceae), *Eur. J. Med. Chem. 93*, 93-100, doi: 10.1016/j.ejmech.2015.01.012.
(16) Casella, J. F., Flanagan, M. D., and Lin, S. (1981) Cytochalasin D inhibits actin polymerization and induces depolymerization of actin filaments formed during platelet shape change, *Nature 293*, 302-305, doi: 10.1038/293302a0.
(17) Garcia-Carceles, J., Caballero, E., Gil, C., and Martinez, A. (2022) Kinase Inhibitors as Underexplored Antiviral Agents, *J. Med. Chem. 65*, 935-954, doi: 10.1021/acs.jmedchem.1c00302.
(18) Brandt, F., Ullrich, M., Wodtke, J., Kopka, K., Bachmann, M., Löser, R., Pietzsch, J., Pietzsch, H. J., and Wodtke, R. (2023) Enzymological Characterization of (64)Cu-Labeled Neprilysin Substrates and Their Application for Modulating the Renal Clearance of Targeted Radiopharmaceuticals, *J. Med. Chem. 66*, 516-537, doi: 10.1021/acs.jmedchem.2c01472.
(19) Aguilera, T. A., Olson, E. S., Timmers, M. M., Jiang, T., and Tsien, R. Y. (2009) Systemic in vivo distribution of activatable cell penetrating peptides is superior to that of cell penetrating peptides, *Integr. Biol. (Camb.) 1*, 371-381, doi: 10.1039/b904878b.
(20) van Duijnhoven, S. M., Robillard, M. S., Nicolay, K., and Grüll, H. (2011) Tumor targeting of MMP-2/9 activatable cell-penetrating imaging probes is caused by tumor-independent activation, *J. Nucl. Med. 52*, 279-286, doi: 10.2967/jnumed.110.082503.
(21) van Duijnhoven, S. M., Robillard, M. S., Nicolay, K., and Grüll, H. (2015) Development of Radiolabeled Membrane Type-1 Matrix Metalloproteinase Activatable Cell Penetrating Peptide Imaging Probes, *Molecules 20*, 12076-12092, doi: 10.3390/molecules200712076.
(22) van Duijnhoven, S. M., Robillard, M. S., Hermann, S., Kuhlmann, M. T., Schäfers, M., Nicolay, K., and Grüll, H. (2014) Imaging of MMP activity in postischemic cardiac remodeling using radiolabeled MMP-2/9 activatable peptide probes, *Mol. Pharm. 11*, 1415-1423, doi: 10.1021/mp400569k.
